# Synthesis and reactivity of a six-membered heterocyclic 1,3-diphosphaallene†

**DOI:** 10.1039/d4sc06371f

**Published:** 2024-11-29

**Authors:** Mahendra K. Sharma, Christoph Wölper, Hannah Siera, Gebhard Haberhauer, Stephan Schulz

**Affiliations:** a Institute of Inorganic Chemistry, University of Duisburg-Essen Universitätsstraße 5-7 D-45141 Essen Germany stephan.schulz@uni-due.de https://www.uni-due.de/ak_schulz/index_en.php; b Institute of Organic Chemistry, University of Duisburg-Essen Universitätsstraße 5-7 D-45141 Essen Germany; c Center for Nanointegration Duisburg-Essen (CENIDE), University of Duisburg-Essen Carl-Benz-Straße 199 47057 Duisburg Germany

## Abstract

1,3-Diphosphaallenes are a new class of heavier heteroallenes and show a fascinating chemical behavior and reactivity. Herein we report on the room temperature transformation of gallaphosphene LGa(OCP)PGaL 1 (L = HC[C(Me)N(Ar)]_2_, Ar = 2,6-i-Pr_2_C_6_H_3_) to the six-membered metallaheterocycle LGa(PCP)OGaL 2 featuring a LGa-substituted 1,3-diphosphaallene unit. The possible mechanism of formation of 2 is supported by quantum chemical calculations, which revealed that the formation of 2 is energetically more favorable (*ca.* 2 kcal mol^−1^) than the formation of 1 at ambient temperature. Remarkably, 2 reacts with singlet carbenes selectively to new five-membered metallaheterocycles LGa(PC)OGaL(P)NHC (NHC = [CMeN(R)]_2_C; R = Me 3, ^i^Pr 4; C{(NAr)CMe_2_CH_2_CMe_2_ = cAAC (5) featuring a 1,3-diphospha-1,3-butadiene unit. In stark contrast, its reaction with trimethylsilyldiazomethane yields (LGa)_2_O(P_2_C_2_H)SiMe_3_6 featuring a 1,3-diphosphacyclobutene unit. Compounds 2–6 were characterized by heteronuclear NMR (^1^H, ^13^C, ^31^P), UV-vis, and IR spectroscopy. Compounds 2–4 and 6 were also characterized by single crystal X-ray diffraction (sc-XRD) and their bonding nature was investigated by quantum chemical calculations.

## Abstract 2

The structure of 1,3-diphosphaallene 2 and reactions with carbenes to compounds 3–5 featuring a 1,3-diphospha-1,3-butadiene unit and with trimethylsilyldiazomethane to the metallaheterocycle 6 featuring a diphosphacyclobutene ring is reported.

## Graphical abstract

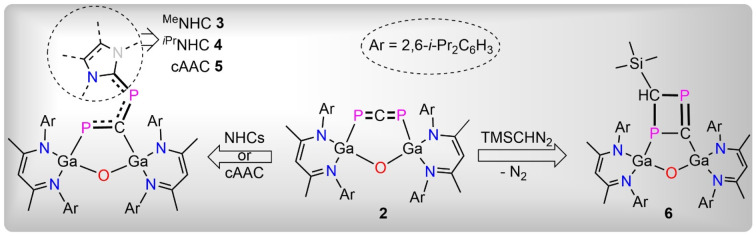

## Introduction

Allenes with two contiguous C–C double bonds represent a fascinating class of unsaturated hydrocarbons due to the unique orthogonal arrangement of the cumulated bonds, which localizes and directs electron distribution, imparting allenes with distinctive chemical properties and reactivity.^1^ For instance, they were reported to undergo cycloaddition and nucleophilic addition reactions, oxidation reactions as well as insertion and bond activation reactions.^1^ Embedding heteroatoms into these allenic systems is particularly attractive in order to modify their electronic structures, reactivity patterns, and photophysical properties, making them valuable reagents in synthetic organic chemistry.^2,3^ In particular, the incorporation of a phosphorus atom into allenes has received significant interest because of its diagonal relationship with carbon in the periodic table that offers a distinctive influence on the allene framework by varying its electron distribution.^4^ The calculated weaker P–C (43 kcal mol^−1^) compared to the C–C double bond energy (65 kcal mol^−1^) provides notable advantages over all-carbon allenes by altering the energies of their frontier molecular orbitals, expanding their utility to small molecule activation, catalysis, and material science.^5^

**Scheme 1:**
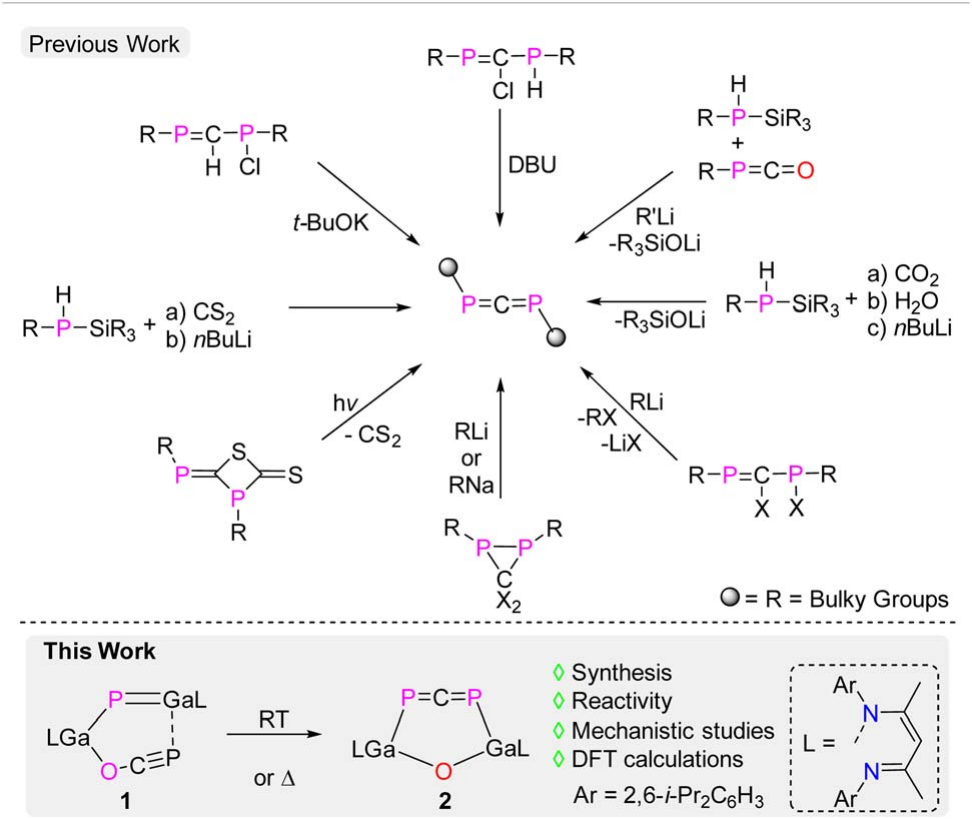
Previously reported synthetic routes to 1,3-diphosphaallenes and synthesis of compound 2 (this work).

In 1984, Yoshifuji,^6^ Appel,^7,8^ and Karsch *et al.*^9,10^ independently prepared the first stable 1,3-diphosphaallene, ArPCPAr (Ar = 2,4,6-*t*-BuC_6_H_2_). Since then, additional kinetically stabilized 1,3-diphosphaallenes have been reported (Scheme 1).^2,3,6–19^ However, their chemical reactivity has been relatively less explored, particularly when compared to intensely studied allenes R_2_CCCR_2_ as well as lighter heteroallenes.^1–3,20^ Quantum chemical calculations on the parent 1,3-diphosphaallene revealed that the LUMO of these compounds typically consists of the low-lying π* orbitals of the P–C double bonds, while the HOMO comprises, more or less similar, two sets of quasi-degenerate n and π orbitals of the PCP unit.^21^ In addition, the calculated charges indicate a negatively charged carbon and positively charged phosphorus atoms.^21^ As a consequence, 1,3-diphosphaallenes were found to react with Lewis acidic transition metals *via* η^1^ or η^2^-coordination. In addition, they underwent protonation as well as dimerization reactions.^2,3^ Furthermore, one-electron redox reactions, reduction reactions, and reactions with organo-lithium reagents have also been reported,^2,3^ whereas reactions of phosphorus-substituted 1,3-diphosphaallenes with Lewis basic N-heterocyclic carbenes (NHCs) remain scarce, in marked contrast to well known reactions of heteroallenes, *i.e.* CO_2_, carbodiimides, isocyanates, and isothiocyanates, with NHCs.

We recently prepared L(X)Ga-substituted phosphaketenes, LGa(X)PCO (X = Cl, PCO; L = HC[C(Me)N(Ar)]_2_; Ar = 2,6-i-Pr_2_C_6_H_3_), which reacted with LGa to gallaphosphenes LGa(X)PGaL (X = Cl, OCP).^22,23^ These were found to undergo cycloaddition reactions with heteroallenes,^24^ X–H bond activation reactions,^25^ and phosphinidene transfer-type reactions with NHCs.^26^ In addition, the photolysis of LGa(Cl)PCO resulted in the formation of the L(Cl)Ga-substituted diphosphene [L(Cl)GaP]_2_, which reacted with an NHC followed by one-electron oxidation to the corresponding radical cation.^27^ Treatment of the bisphosphaketene LGa(PCO)_2_ with a cyclic alkyl(amino)carbene (cAAC) furthermore yielded the 1,2-diphospha-1,3-butadiene LGa(P_2_OC)cAAC containing a π-conjugated P=P–C=C framework, which by one-electron reduction reaction with potassium graphite (KC_8_) gave the corresponding radical anion.^28^ These remarkable findings encouraged us to investigate the reactivity of gallaphosphenes in more detail, and we herein report on the isomerization of LGa(OCP)PGaL 1 to the six-membered metallaheterocycle LGa(PCP)OGaL 2 featuring a 1,3-diphosphaallene unit (Scheme 1). Subsequent reactions with singlet carbenes and trimethylsilyldiazomethane (TMSCHN_2_) yielded unprecedented five-membered metallaheterocycles featuring 1,3-diphospha-1,3-butadienes LGa(PC)OGaL(P)^R^NHC (^R^NHC = [CMeN(R)]_2_C; R = Me 3, i-Pr 4; C{(NAr)CMe_2_CH_2_CMe_2_5) and a fused-ring metallaheterocycle (LGa)_2_O(P_2_C_2_H)SiMe_3_6 featuring a 1,3-diphosphacyclobutene ring.

## Results and discussion

**Scheme 2:**
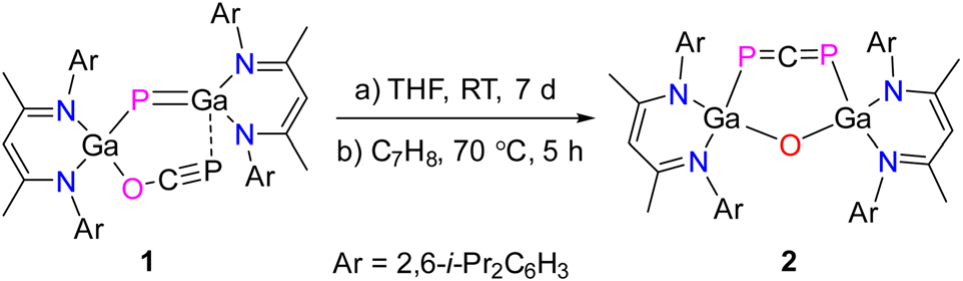
Synthesis of 1,3-diphosphaallene 2.

Gallaphosphene LGa(OCP)PGaL 1 undergoes gradual isomerization to the six-membered metallacycle LGa(PCP)OGaL 2 featuring a 1,3-diphosphaallene unit upon storage in solution (toluene, THF) at ambient temperature (Scheme 2). However, heating a toluene solution of 1 to 70 °C was found to significantly accelerate the rate of this isomerization reaction, resulting in quantitative conversion of 1 to 2 within 5 hours. Upon evaporation of the solvent, 2 was isolated as an olive colored solid in 98% yield. Compound 2 is soluble in THF, toluene, fluorobenzene, and *n*-hexane and stable both in solution and in solid state under inert gas atmosphere at room temperature, whereas it immediately decomposes upon exposure to air and moisture.

Its ^1^H NMR spectrum shows two sets of resonances for the aryl groups of the β-diketiminate ligand, indicating their asymmetric nature in solution (Fig. S1†). This was confirmed by variable-temperature (VT) ^1^H NMR studies, which showed no dynamic behaviour over the temperature range of −60 °C to +75 °C (Fig. S25 and S26†). The ^13^C{^1^H} NMR spectrum of 2 (272.1 ppm, ^1^*J*_CP_ = 73.3 Hz) shows the expected triplet for the phosphorus bound carbon atom of the 1,3-diphosphaallene unit, which is in the range of known 1,3-diphosphaallenes (270 ppm to 280 ppm).^2,3^ However, the ^1^*J*_PC_ coupling constant (73.3 Hz) is rather large in comparison to those of other 1,3-diphosphaallenes (^1^*J*_PC_ = ∼58 Hz),^2,3^ which most likely results from the introduction of electropositive LGa-substituents. The ^31^P{^1^H} NMR spectrum of 2 (–30.9 ppm) displays a sharp singlet for the phosphorus atoms, which is shifted to higher field compared to known 1,3-diphosphaallenes (140 to 169 ppm), which again reflects the influence of the electropositive LGa-substituents.^2,3^

**Fig. 1:**
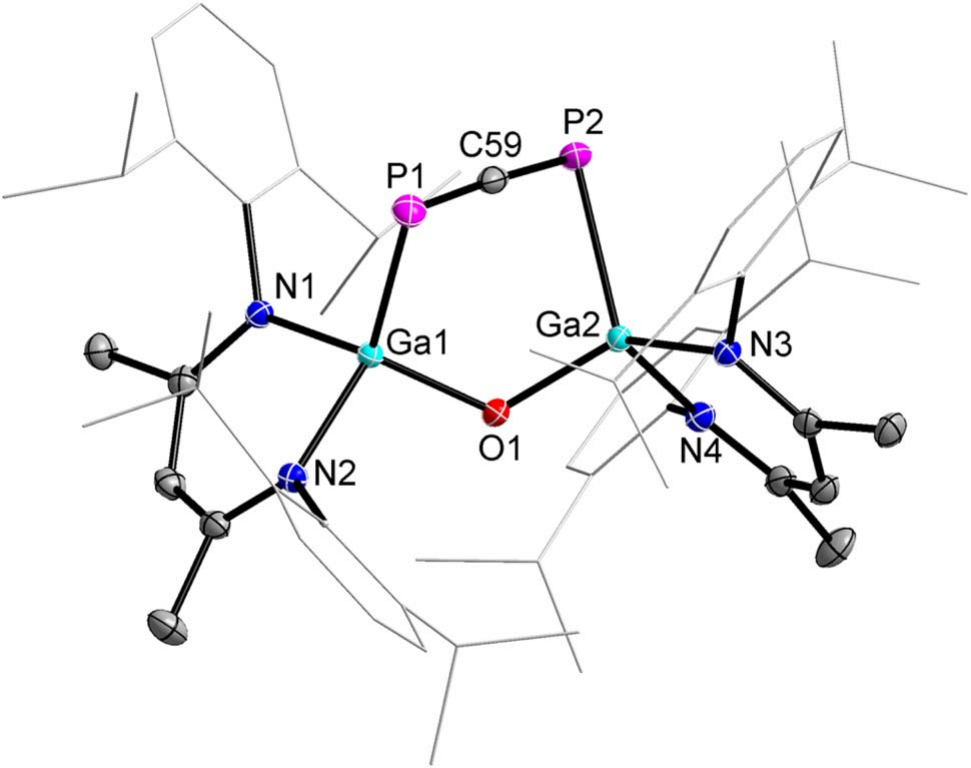
Molecular structure of 1,3-diphosphaallene 2. Ellipsoids set at 50% probability; hydrogen atoms and alternate positions of the disordered parts are omitted for clarity. Selected bond length (Å) and angles (°): P(1)–C(59) 1.6516(13), P(2)–C(59) 1.6506(13), Ga(1)–P(1) 2.3767(3), Ga(2)–P(2) 2.3845(3), Ga(1)–O(1) 1.8082(8), Ga(2)–O(1) 1.8046(8); P(2)–C(59)–P(1) 176.48(8), O(1)–Ga(1)–P(1) 107.78(3), O(1)–Ga(2)–P(2) 107.62(3), Ga(2)–O(1)–Ga(1) 123.63(4).

2 crystallizes in the monoclinic space group *P*2_1_/*c* containing one molecule per asymmetric unit cell or four molecules per unit cell as shown by single-crystal X-ray diffraction (sc-XRD, Fig. 1).^29^ To the best of our knowlegde, 2 represents only the second structurally characterized 1,3-diphosphaallene with cumulated P–C and C–P double bonds.

The solid-state molecular structure of compound 2 revealed a linear P–C–P unit with a bond angle of 176.48(8)°, which is slightly larger than that of the 1,3-diphosphaallene ArPCPAr (Ar = 2,4,6-*t*-BuC_6_H_2_, 172.6(5)°).^10^ The P atoms are twofold- and the Ga atoms fourfold-coordinated. The P1–C59 (1.6516(13) Å) and P2–C59 (1.6506(13) Å) bond lengths are equidistant and in the typical range of phosphaalkenes (1.65–1.67 Å).^30^ They agree with the sum of the calculated P–C double bond radii (P 1.02 Å; C 0.67 Å)^31^ and are comparable to P–C double bond lengths reported for phosphaallenes.^2,3^ The Ga1–P1 (2.3767(3) Å) and Ga2–P2 (2.3845(3) Å) bond lengths are almost identical but slightly longer than the Ga–P single bonds of gallaphosphene 1 (2.2943(16) Å),^22^ phosphaalkenes L(Cl)GaP(^R^NHC) (R = Me, 2.2441(3) Å; R = i-Pr, 2.2538(3) Å),^26^ L(Cl)GaP(cAAC) (2.289 Å),^32^ diphosphene [L(Cl)GaP]_2_ (2.313(3) Å),^27^ and 1,2-diphospha-1,3-butadiene (2.2844(4) Å),^28^ respectively, but agree with the sum of the calculated Ga–P single-bond radii (Ga 1.24 Å; P 1.11 Å).^33^ The IR spectrum of 2 was simulated by DFT calculations, showing strong P–C asymmetric stretching frequencies at 1347 (PBE0) or 1297 (PBE) cm^−1^ (Fig. S5 and S6†). Unfortunately, C–C and C–H stretching vibrations typically appear in this regime, hence we were unable to assign the P–C asymmetric stretching band in the experimental IR spectrum.

**Fig. 2:**
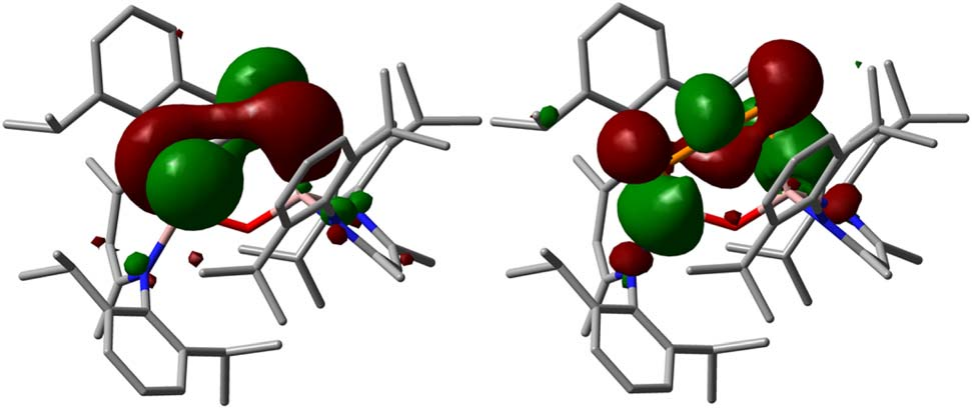
HOMO and LUMO of 1,3-diphosphaallene 2 calculated at PBE0-D3BJ level of theory (isovalue 0.03 a.u.). Hydrogen atoms are omitted for clarity.

DFT calculations were performed to get a deeper insight into the electronic structure of compound 2 and the reaction mechanism of the transformation of 1 into 2 (for details see ESI†). The DFT-optimized geometry of 2 is in good agreement with the solid-state molecular structure. Mayer and Wiberg bond orders analyses calculated at PBE0-D3BJ level of theory show bond orders for the PCP unit of 1.77 to 1.78, which is in accordance with a phosphaallene (Fig. S41†). The HOMO and LUMO of 2 range over the PCP unit, with their spatial orientation being almost perpendicular to each other (Fig. 2). The HOMO essentially corresponds to the antibonding linear combination of the lone pairs on the phosphorus atoms, while the LUMO is composed of the p orbitals of the three centers of the phosphaallene unit, whereby it exhibits two nodes. The p-orbitals are located almost in one plane, whereas organic allenes typically show p orbitals of the outer centers perpendicular to each other. This is probably due to the fact that the PCP unit is part of a six-membered ring.

**Fig. 3:**
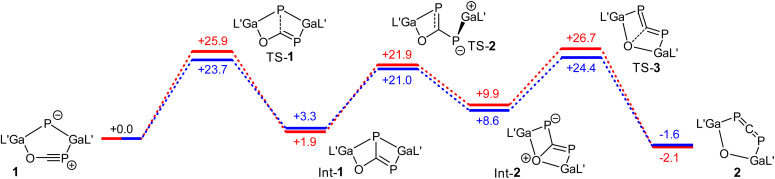
Gibbs energies (*G*) for the rearrangement from 1 to 2 calculated by means of PBE0-D3BJ (red) and PBE-D3BJ (blue). L′ = HC[C(Me)NDipp]_2_, Dipp = 2,6-i-Pr_2_C_6_H_3_. The values are given in kcal mol^−1^.

The mechanistical calculations reveal that the transformation of 1 into 2 takes place in three steps. In the first one, a P–C bond formation occurs, whereby the bicyclic intermediate Int-1 is formed (Fig. 3). In the next step, the Ga–P bond is broken along with a concerted rotation around the C–P bond and a further Ga–O bond is formed. The final step of the rearrangement represents the opening of the bicycle yielding phosphaallene 2, whereby the C–O bond is broken. The formation of the intermediates Int-1 and Int-2 are endergonic reactions, whereas the generation of product 2 is slightly exergonic. The energies of the transition states of the three steps are in a similar range, with the last step being the rate-determining one. The activation energies calculated using PBE-D3BJ (blue in Fig. 3) and PBE0-D3BJ (red) for the third step amounts to 24.4 and 26.7 kcal mol^−1^, respectively. From this, half-lives at 70 °C of 0.1 and 2.7 h can be calculated. Within the error limits of DFT calculations, this agrees very well with the experimental observation, which shows a complete conversion of 1 into 2 within 5 h. Calculations for the rearrangement of an analogous system to 1 with sterically less demanding ligands at the gallium atoms reveal that the size of the ligands should not have a significant impact on the reaction rate (Fig. S42†).

Encouraged by the results from DFT calculations, we sought to probe the chemical reactivity of the 1,3-diphosphaallene 2 in more detail. Heteroallenes such as CO_2_, carbodiimides, isocyanates, and isothiocyanates were shown to react with N-heterocyclic carbenes (NHCs) to the corresponding betaine adducts,^34^ whose electronic properties can be fine-tuned by variation of the carbene. Interestingly, analogous reactions of 1,3-diphosphaallenes with N-heterocyclic carbenes are virtually unknown. To the best of our knowledge, only the 1,3-diphosphaallene ArPCPAr (Ar = 2,4,6-*t*-BuC_6_H_2_)^6–10^ was reported to react with an *in situ* generated dichlorocarbene, however this reaction rather yielded a methylenediphosphirane than the corresponding betaine adduct.^35^ This attracted our interest to explore reactions of 2 with isolable carbenes in more detail. Since numerous singlet carbenes with different stereoelectronic properties are known,^36^ we initially chose to react compounds 2 with small N-heterocyclic carbenes, ^R^NHC (^R^NHC = [CMeN(R)]_2_C; R = Me, i-Pr).

**Scheme 3:**
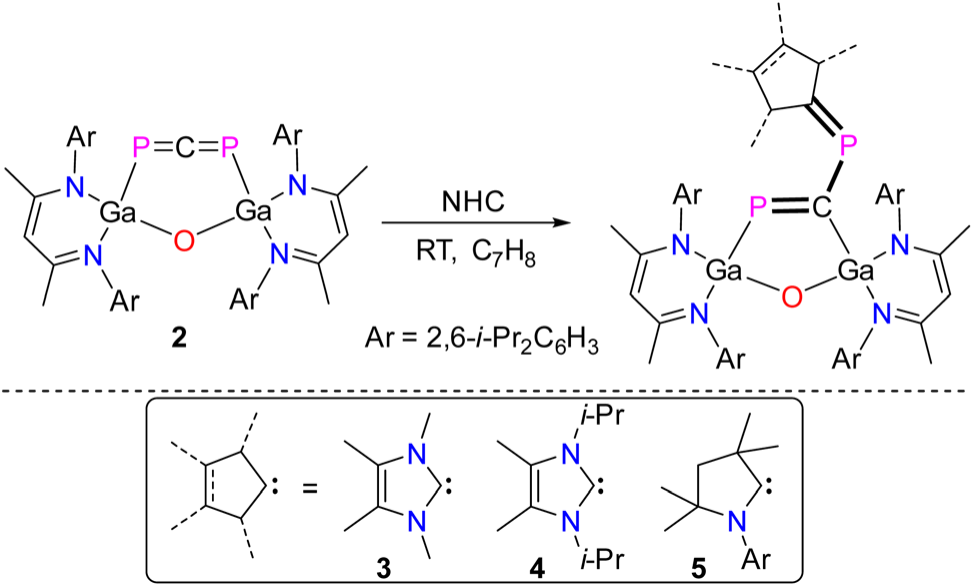
Reactions of 2 with singlet carbenes to 1,3-diphospha-1,3-butadienes 3–5.

Treatment of 2 with equimolar amounts of ^R^NHC at ambient temperature proceeded with an immediate color change from olive to red and formation of the metallaheterocycles LGa(PC)OGaL(P)^R^NHC (R = Me 3, i-Pr 4) featuring a 1,3-diphospha-1,3-butadiene unit (Scheme 3). The analogous reaction of 2 with a sterically more demanding cyclic (alkyl)(amino)-carbene (cAAC) to the metallaheterocycle LGa(PC)OGaL(P)cAAC 5 took 6 hours for completion (93%), whereas the sterically even more hindered IPr carbene (IPr = [CHN(Ar)]_2_C; Ar = 2,6-i-Pr_2_-C_6_H_3_) failed to react, clearly reflecting the effect of steric bulk on the reaction process.

Compounds 3–5 are soluble in common organic solvents and stable under argon atmosphere at ambient temperature, but rapidly decompose upon exposure to air. The ^1^H and ^13^C{^1^H} NMR spectra of compounds 3–5 exhibit two distinct sets of resonances for the β-diketiminate ligand as was observed for L(X)Ga-substituted dipnictenes,^37^ gallapnictenes,^22,38^ and other complexes.^39^ The ^31^P{^1^H} NMR spectra of 3 (168.59 ppm, ^2^*J*_PP_ = 18.0 Hz; 74.73 ppm, ^2^*J*_PP_ = 18.0 Hz), 4 (153.34 ppm, ^2^*J*_PP_ = 22.1 Hz; 73.94 ppm, ^2^*J*_PP_ = 22.1 Hz), and 5 (434.01 ppm, ^2^*J*_PP_ = 53.5 Hz; 143.46 ppm, ^2^*J*_PP_ = 52.7 Hz) each display two doublets for the electronically nonequivalent phosphorus atoms, which are downfield shifted with respect to starting 1,3-diphosphaallene 2 (–30.9 ppm). This downfield shift in the ^31^P signals of compound 5 is more pronounced due to the increased π-acidity of the cyclic alkylamino carbene (cAAC) compared to the NHCs in compounds 3 and 4.^36^ Interestingly, the ^2^*J*_PP_ coupling constants in compounds 3–5 (18.0 Hz to 53.4 Hz) are rather small in comparison to the ^2^*J*_PP_ coupling constants of the previously reported acyclic 1,3-diphospha-1,3-butadienes, which range from 112 Hz to 122 Hz.^40^

**Fig. 4:**
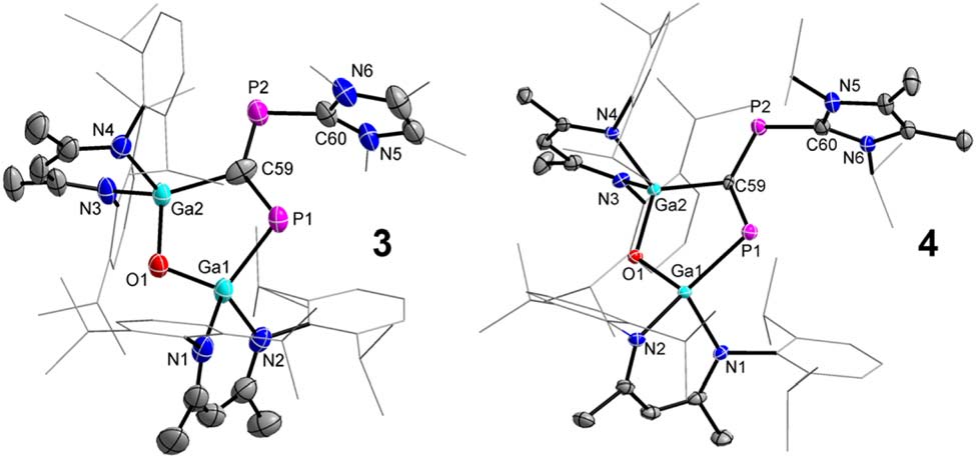
Molecular structures of compounds 3 and 4. Ellipsoids set at 50% probability; hydrogen atoms, solvent molecules (*n*-hexane 3, benzene 4), alternate positions of the disordered parts (3) are omitted for clarity. Selected bond length (Å) and angles (°): 3: Ga(1)–P(1) 2.2926(11), Ga(2)–C(59) 2.092(8), P(1)–C(59) 1.801(6), P(2)–C(59) 1.623(7), P(2)–C(60) 1.847(3); P(2)–C(59)–P(1) 128.9(5), C(59)–P(2)–C(60) 97.5(3); 4: Ga(1)–P(1) 2.3351(4), Ga(2)–C(59) 2.0285(14), P(1)–C(59) 1.7716(15), P(2)–C(59) 1.6990(15), P(2)–C(60) 1.8571(17); P(2)–C(59)–P(1) 129.91(8), C(59)–P(2)–C(60) 105.29(7).

The molecular structures of compounds 3 and 4 were determined by sc-XRD (Fig. 4). Single crystals were obtained by either storing a saturated *n*-hexane solution of 3 to −30 °C or by diffusing *n*-hexane into a saturated benzene solution of 4 at ambient temperature. Compounds 3 and 4 crystallize in the monoclinic space group *C*2/*c*.^29^ The solid-state structures confirmed the formation of the five-membered LGa-containing metallaheterocycles and revealed a planar *cis*-1,3-diphospha-1,3-butadiene unit with conjugated P–C bonds. The five-membered Ga_2_OPC metallaheterocycle is twisted, with a dihedral angle (*ϕ* interplanar angle of the best plane of C60, P2, C59, P1, and Ga2, C59, P1, Ga1, O1) of approximately 17° (3, no reliable value can be given due to the disorder) and 10.46(8)° (4), respectivley, whereas the six-membered C_3_N_2_Ga heterocylce is almost perpendicular to this plane.

The P1–C59 bond lengths (Table 1) in 3 (1.801(6) Å) and 4 (1.7716(15) Å) are slightly shorter than the P2–C60 bond lengths (1.847(3) Å 3 and 1.8571(17) Å 4) but almost 10 pm elongated compared to P–C double bonds in phosphaalkenes (1.65–1.67 Å)^30^ as well as the P–C double bonds observed in 2 (1.6516(13) Å, (1.6506(13) Å), respectively. However, they are comparable to the P–C single bonds in LGa-substituted phosphinidenes (1.8099(13) Å)^26^ and the calculated P–C single-bond radii (P 1.11 Å; C 0.75 Å).^33^ In marked contrast, the central C59–P2 bonds in 3 (1.623(7) Å) and 4 (1.6990(15) Å) are significantly shorter than the P1–C59 and the P2–C60 bonds but comparable to P–C double bonds in phosphaalkenes (1.65–1.67 Å)^30^ and the calculated P–C double-bond radii (P 1.02 Å; C 0.67 Å),^31^ respectively. The structural features point to a highly delocalized P_2_C_2_ π-conjugated system.

**Table 1:** Selected bond lengths (Å) of compounds 2–4 and 6

	P1–C59	C59–P2	P2–C60	Ga1–P1	Ga2–C59
2	2.038(5)	2.313(3)	—	2.313(3)	—
3	1.801(6)	1.623(7)	1.847(3)	2.2926(11)	2.092(8)
4	1.7716(15)	1.6990(15)	1.8571(17)	2.3351(4)	2.0285(14)
6	1.827(9)	1.720(10)	1.875(6)	2.503(2)	1.940(10)

The Ga1–P1 bond lengths in 3 (2.2926(11) Å) and 4 (2.3351(4) Å) are slightly shorter compared to 2 (2.3767(3)/2.3845(3) Å) but comparable to the sum of the calculated Ga–P single-bond radii (Ga 1.24 Å; P 1.11 Å)^33^ and Ga–P single bonds in the diphosphene [L(Cl)GaP]_2_ (2.313(3) Å)^27^ and the 1,2-diphospha-1,3-butadiene LGa(P_2_OC)cAAC (2.2844(4) Å), respectively.^28^ Similarly, the Ga2–C59 bond lengths are comparable to Ga–C single bonds in LGa(C_6_F_5_)F (1.991(2) Å),^41^ LGa(C_5_H_4_NO)OH (1.9698(13) Å),^42^ and the sum of the calculated Ga–C single bond radii (Ga 1.24 Å; C 0.75 Å).^33^

**Scheme 4:**
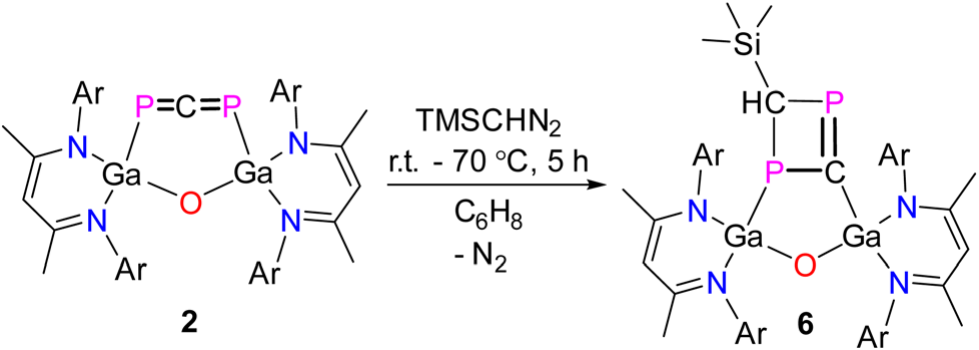
Reaction of 1,3-diphosphallene 2 with trimethylsilyldiazomethane to 6.

To check if 1,3-diphosphaallene 2 only reacts as alkylidene carbene transfer reagent as was observed in reactions with NHCs and cAAC or is also capable for cycloaddition reactions at the P–C double bonds, we reacted 2 with an equimolar amount of trimethylsilyldiazomethane (TMSCHN_2_). This reaction remarkably yielded (LGa)_2_O(P_2_C_2_H)SiMe_3_6 with a 1,3-diphosphacyclobutene unit (Scheme 4).

Compound 6 is a yellow crystalline solid, which is soluble in common organic solvents and stable under an inert gas atmosphere at ambient temperature both in solution and in the solid state, but rapidly decomposes when exposed to air. The ^1^H and ^13^C{^1^H} NMR spectra of compound 6 exhibit the expected sets of resonances for the β-diketiminate ligand, and the ^31^P{^1^H} NMR spectrum showed two doublets at 386.48 ppm (^2^*J*_PP_ = 96.8 Hz) and 89.30 ppm (^2^*J*_PP_ = 96.8 Hz) for the magnetically non-equivalent phosphorus atoms. These signals are downfield shifted compared to the signals observed for the 1,3-diphosphaallene 2 and compounds 3–4, respectively, but they are still at higher field compared to compound 5. Interestingly, the ^2^*J*_PP_ coupling constants of 6 are considerably larger than those observed for compounds 3–5, but they comparable to those of a 1,3-diphospha-1,3-cyclobutene, (Mes*)_2_P_2_H_2_(Me)*t*-Bu (^2^*J*_PP_ = 92 Hz; Mes* = 2,4,6-*t*-Bu_3_C_6_H_2_).^43^

**Fig. 5:**
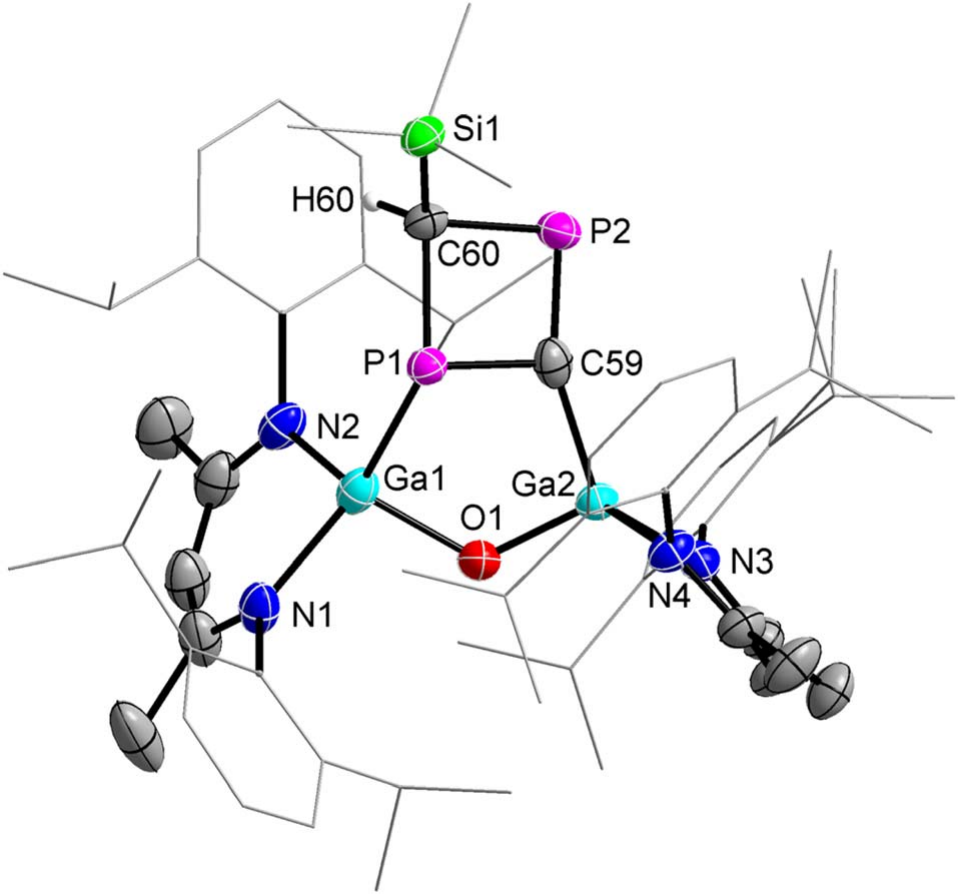
Molecular structure of compound 6. Ellipsoids set at 50% probability; hydrogen atoms, disordered solvent molecules (*n*-hexane), and alternate positions of the disordered parts are omitted for clarity. Selected bond length (Å) and angels (°): Ga(1)–P(1) 2.503(2), Ga(2)–O(1) 1.815(2), Ga(2)–C(59) 1.940(10), P(1)–C(59) 1.827(9), P(1)–C(60) 1.888(6), P(2)–C(59) 1.720(10), P(2)–C(60) 1.875(6).

The molecular structure of compound 6 was determined by sc-XRD (Fig. 5). Compound 6 crystallized in the monoclinic space group *P*2_1_/*c*.^29^ The planar four-membered P_2_C_2_ ring doesn't adopt a co-planar orientation to the five-membered (LGa)_2_OPC ring, most likely to reduced steric interactions. The interplanar angle of the best plane of Ga1, P1, C59, Ga2, O1, and P1, C59, P2, C60 is approximately 50° (no reliable value can be given due to the disorder). The refined diffraction data showed a rotational disorder of the four-membered P_2_CCH(SiMe_3_) ring (52/48) over two positions, with additional disorder in the solvent molecule (*n*-hexane). The P1–C59 (1.827(9) Å), P1–C60 (1.888(6) Å), and P2–C60 (1.875(6) Å) single bond lengths in the four-membered P_2_C_2_ ring are consistent with previously reported P–C single bond lengths^28^ and the calculated P–C single-bond radii (P 1.11 Å; C 0.75 Å).^33^ The P2–C59 (1.720(10) Å) double bond is significantly shorter and comparable to P–C double bonds reported for phosphaalkenes (1.65–1.67 Å)^30^ and the calculated P–C double-bond radii (P 1.02 Å; C 0.67 Å).^31^

**Fig. 6:**
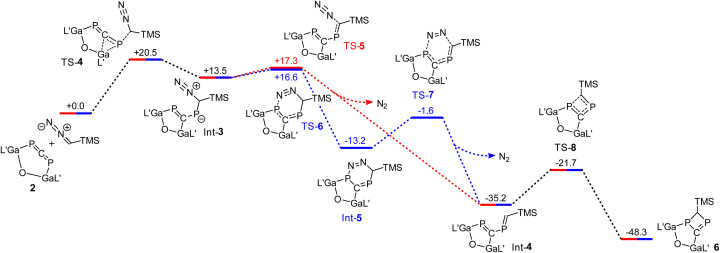
Gibbs energies (*G*) for the reaction of 2 with Me_3_SiCHN_2_ to 6 calculated by means of PBE0-D3BJ. L′ = HC[C(Me)NDipp]_2_, Dipp = 2,6-i-Pr_2_C_6_H_3_. The values are given in kcal mol^−1^.

The reaction mechanism of the formation of 6 was also investigated by quantum chemical calculations (for details see ESI†). DFT calculations show that the reaction of 2 with trimethylsilyldiazomethanes takes place over several steps (Fig. 6). The initial process (TS-4) is the rate-determining one, and involves the attack of the carbon atom from the carbene on a phosphorus atom of the allene in combination with a concerted rearrangement of the gallium center from the phosphorus to the carbon atom. The stabilizing effect of this attack is the interaction of the free electron pair on the carbene with the LUMO of the phosphaallene. The as-formed intermediate Int-3 can be converted into the diene Int-4*via* two different routes: The removal of nitrogen can occur in a single step (TS-5), or in two steps *via* the formation of the bicycle Int-5. The corresponding transition states TS-5 and TS-6 are energetically very similar at 17.3 and 16.6 kcal mol^−1^, so that neither reaction path can be excluded. The final cyclization of the diene Int-4 then yields bicycle 6.

**Fig. 7:**
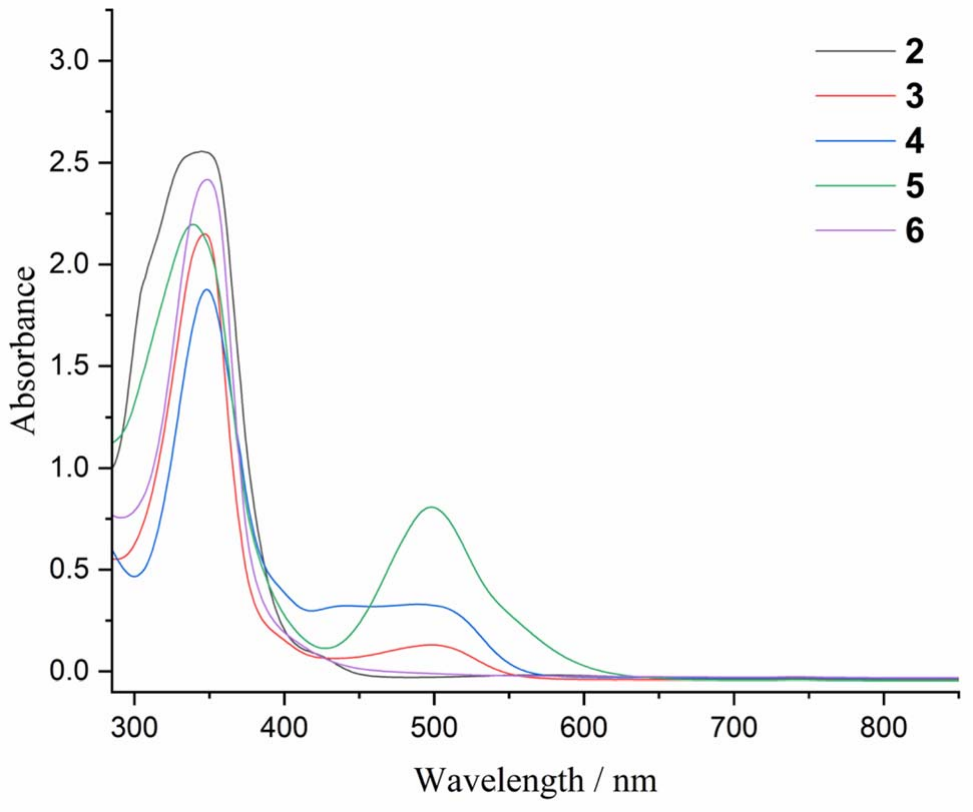
UV-vis spectra of compounds 2–6 in THF.

The UV-vis spectra of the five-membered metallaheterocycles 3 (345, 496 nm), 4 (345, 489 nm), and 5 (345, 498 nm) featuring a 1,3-diphospha-1,3-butadiene unit exhibit two main absorptions (Fig. 7) which based on the TD-DFT calculations at TD-PBE0-D3BJ(SMD,THF)/def2-SVP level of theory, comprise dominant contributions of the π → π* transitions of the β-diketiminate ligand backbone and the 1,3-diphospha-1,3-butadiene π-systems, respectively (Fig. S35–S39 and S46–S51†). In contrast, the UV-vis spectra of compounds 2 and 6 showed only one strong absorption band at 345 nm (Fig. 7) that corresponds to the π → π* transitions of the β-diketiminate ligand backbone π-system (Fig. S33, S34, S40 and S43–S45†).

## Conclusions

We report on a unique and atom economic method for the synthesis of the LGa-substituted 1,3-diphosphaallene 2, which to the best of our knowledge represents only the second structurally characterized 1,3-diphosphaallene. Quantum chemical calculations provided profounds insights into its electronic structure and the energetics of the thermal rearrangement of gallaphosphene 1 to 1,3-diphosphaallene 2. 1,3-Diphosphaallene 2 was found to react as alkylidene carbene transfer reagent in reactions with NHCs and cAAC, yielding the corresponding five-membered metallaheterocycles 3–5 featuring a unique 1,3-diphospha-1,3-butadiene unit, whereas a [3 + 2] cycloaddition reaction to the metallaheterocycle 6 featuring a diphosphacyclobutene unit occurred with trimethylsilyldiazomethane. These interesting results clearly demonstrate the promising potential of gallaphosphene 1 for the synthesis of P-containing 1,3-diphosha-1,3-butadienes with distinct electronic structures, since a variety of singlet carbenes with different stereoelectronic properties are available. Furthermore, the formation of compounds 6 confirms the promising potential of compound 2 in cycloaddition reactions, which may allow for the synthesis of novel P-containing heterocycles.

## Data availability

The data supporting this article have been included as part of the Supplementary Information. Crystallographic data has been deposited at the CCDC under CCDC 2362185 (2), 2362186 (3), 2362187 (4), and 2362188 (6) and can be obtained from https://doi.org/10.1039/d4sc06371f

## Author contributions

M. K. S., conceptualization, experimentation, characterization, writing – original draft; C. W., sc-XRD data acquisition and processing; H. S., quantum chemical calculations; G. H. quantum chemical calculations, supervision; S. S., supervision, final writing, funding acquisition, project administration.

## Conflicts of interest

There are no conflicts to declare.

## Supplementary Material

SC-OLF-D4SC06371F-s001

SC-OLF-D4SC06371F-s002
