# Supplementary material for: Synthesis and reactivity of a six-membered heterocyclic 1,3-diphosphaallene
Source: Chem Sci. 2024 Nov 29;16(3):1189–96. doi: 10.1039/d4sc06371f (PMC11629509; doi:10.1039/d4sc06371f)
Supplement: SC-016-D4SC06371F-s001 [file SC-016-D4SC06371F-s001.pdf]

Supplementary Information (SI) for Chemical Science.
This journal is © The Royal Society of Chemistry 2024

Supporting Information

Content

1. Experimental Section

2. Spectroscopic Characterization

Figure S1-S4. ^{1}H, ^{13}C, ^{31}P NMR and IR spectra of compound **2**.

Figure S5-S6. Simulated IR spectrum of compound **2**.

Figure S7-S10. ^{1}H, ^{13}C, ^{31}P NMR and IR spectra of compound **3**.

Figure S11-S14. ^{1}H, ^{13}C, ^{31}P NMR and IR spectra of compound **4**.

Figure S15. Simulated IR spectrum of compound **4**.

Figure S16-S19. ^{1}H, ^{13}C, ^{31}P NMR and IR spectra of compound **5**.

Figure S20-S23. ^{1}H, ^{13}C, ^{31}P NMR and IR spectra of compound **6**.

Figure S24. Simulated IR spectrum of compound **6**.

Figure S25-S26. Stacked variable temperature (VT) ^{1}H NMR spectra of **2**.

Figure S27-S28. Stacked variable temperature (VT) ^{31}P NMR spectra of **2**.

3. Crystallographic Details

Table S1, S2. Crystal data and structure refinement parameters of compounds **2**–**4** and **6**.

Figure S29-S32. Molecular structures of compounds **2**–**4** and **6**.

4. UV-vis Spectroscopy

Figure S33. UV-visible spectrum of compound **2**.

Figure S34. Simulated UV-visible spectrum of compound **2**.

Figure S35. UV-visible spectrum of compound **3**.

Figure S36. Simulated UV-visible spectrum of compound **3**.

Figure S37. UV-visible spectrum of compound **4**.

Figure S38. Simulated UV-visible spectrum of compound **4**.

Figure S39. UV-visible spectrum of compound **5**.

Figure S40. UV-visible spectrum of compound **6**.

5. Computational Details

Figure S41. Mayer, Wiberg bond orders and NBO-charges of compound **2**.

Figure S42. Gibbs energies for the rearrangement from **S1** to **S2**.

Figure S43-S51. Natural transition orbitals of compounds **2-4**.

6. Cartesian Coordinates and Absolute Energies for the Calculated Compound

Tables S3-5. Absolute energies of the calculated compounds by means of different methods.

7. References

1. Experimental Section

General procedure. All experiments and manipulations were carried out under a dry argon atmosphere using either standard *Schlenk* techniques or an MBraun LABmaster Pro glovebox. THF, toluene, and *n*-hexane were dried using Braun solvent drying system, degassed, and stored over 4Å molecular sieve. Benzene-d_6 was dried by refluxing over NaK, distilled prior to use, and stored over 4Å molecular sieve. Commercial reagents were purchased from Aldrich, Acros, or Alfa-Aesar Chemical Co. and used as received. LGa(OCP)P=GaL **1** (L = HC[C(Me)N(Ar)]$_2$, Ar = 2,6-iPr$_2$C$_6$H$_3$),[1,2] ^{Me}NHC,[3] ^{iPr}NHC,[3] and cAAC·LiOTf[4] were prepared according to literature procedures. Trimethylsilyldiazomethane (0.6 M solution in *n*-hexane) was purchased from sigma-aldrich and used as received. NMR spectra (^{1}H, ^{13}C, and ^{31}P) were recorded on a Bruker Avance III HD 300 (^{1}H 300 MHz, ^{13}C{^{1}H} 75 MHz, ^{31}P{^{1}H} 121 MHz), or a Bruker Avance III HD 400 (^{1}H 400 MHz, ^{13}C{^{1}H} 100 MHz, ^{31}P{^{1}H} 161 MHz), or a Bruker Avance II 500 (^{1}H 500 MHz, ^{13}C{^{1}H} 150 MHz, ^{31}P{^{1}H} 202 MHz) spectrometer and were referenced to internal C$_6$D$_5$H (^{1}H δ = 7.16; ^{13}C δ = 128.06) or external 85% H$_3$PO$_4$ (^{31}P, δ = 0) using chi-values (χ).[5] Elemental analyses were performed at the *Elementaranalyse Labor* of the University of Duisburg-Essen. IR spectra were recorded by a Bruker ALPHA-T FT-IR spectrometer equipped with a single-reflection ATR sampling module.

Synthesis of compound 2.

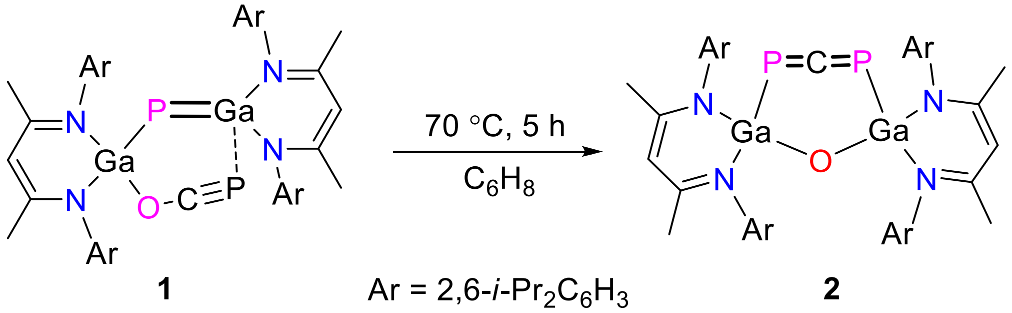

1 **2**

A toluene (20 mL) solution of LGa(OCP)P=GaL **1** (1.0 g, 0.94 mmol) was heated at 70 °C for 5 h, yielding an olive colored solution. All volatiles were removed in *vacuo* to yield a olive colored crystalline powder. Yield: 98% (980 mg). M.p. 121 °C (dec.). Single crystals suitable for X-ray diffraction were grown by diffusing *n*-hexane into a saturated benzene solution of **2** at ambient temperature. Anal. calcd. (%) for $C_{59}H_{82}Ga_2N_4OP_2$ (1064.70): C, 66.56; H, 7.76; N, 5.26. Found: C, 66.63; H, 7.84; N, 5.31. ^{1}H NMR (300 MHz, C$_6$D$_6$, 298 K) δ = 0.84 (d, $^3J_{HH}$ = 6.6 Hz, 6H, CH(CH_3)$_2$), 1.01 (d, $^3J_{HH}$ = 6.6 Hz, 6H, CH(CH_3)$_2$), 1.06 (d, $^3J_{HH}$ = 6.8 Hz, 6H, CH(CH_3)$_2$), 1.09 (d, $^3J_{HH}$ = 6.7 Hz, 6H, CH(CH_3)$_2$), 1.24 (d, $^3J_{HH}$ = 6.6 Hz, 6H, CH(CH_3)$_2$), 1.37 (d, $^3J_{HH}$ = 6.6 Hz, 6H, CH(CH_3)$_2$), 1.41 (d, $^3J_{HH}$ = 6.8 Hz, 6H, CH(CH_3)$_2$), 1.46 (s, 6H, CCH_3), 1.52 (s, 6H, CCH_3), 1.66 (d, $^3J_{HH}$ = 6.7

Hz, 6H, CH(CH_3)$_2$), 3.09 (sept, $^3J_{HH}$ = 6.6 Hz, 2H, CH(CH$_3$)$_2$), 3.21 (sept, $^3J_{HH}$ = 6.8 Hz, 2H, CH(CH$_3$)$_2$), 3.26 (sept, $^3J_{HH}$ = 6.7 Hz, 2H, CH(CH$_3$)$_2$), 4.19 (sept, $^3J_{HH}$ = 6.7 Hz, 2H, CH(CH$_3$)$_2$), 4.77 (s, 2H, CH), 7.06 (d, $^3J_{HH}$ = 6.9 Hz, 2H, C_6H_3), 7.12 (d, $^3J_{HH}$ = 7.2 Hz, 6H, C_6H_3), 7.23 (d, $^3J_{HH}$ = 7.6 Hz, 2H, C_6H_3), 7.29 (d, $^3J_{HH}$ = 7.5 Hz, 2H, C_6H_3). ^{13}C{^{1}H} NMR (75 MHz, C_6D_6, 298 K) δ = 23.5, 23.8, 24.6, 24.7, 25.0, 25.1, 25.3, 26.2 (CH(CH$_3$)$_2$), 27.6, 28.8, 29.9 (CH(CH$_3$)$_2$), 96.2 (CH), 124.0, 124.1, 124.2, 125.2, 127.0, 127.1, 142.5, 142.8, 142.9, 143.4, 144.5, 146.2, 169.0, 170.3 (C_6H$_3$), 272.1 (t, J_{CP} = 73.3 Hz, PCP). ^{31}P{^{1}H} (121 MHz, C_6D_6, 298 K) δ = –30.9 ppm. ATR-IR: ν 2995, 2928, 2868, 1526, 1435, 1386, 1313, 1255, 1176, 1096, 1021, 935, 862, 794, 759, 740, 678, 627, 602, 517 cm^{-1}.

Synthesis of compound 3.

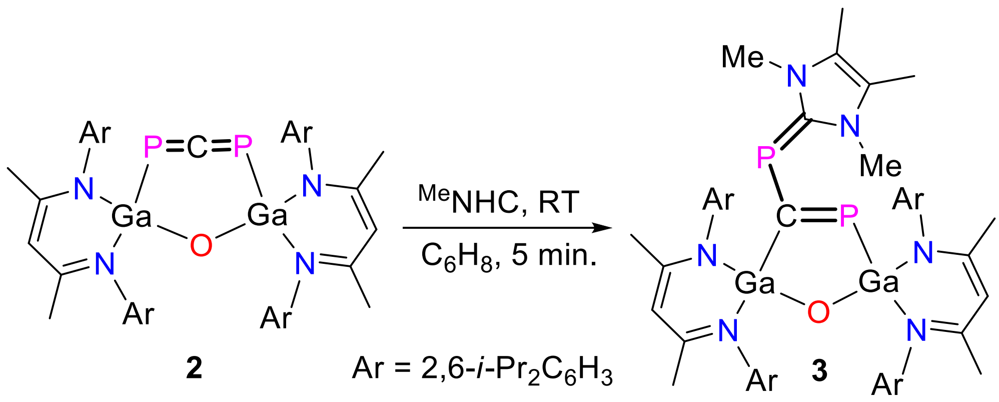

To a toluene (5 mL) solution of **2** (100 mg, 0.094 mmol), ^{Me}NHC (12 mg, 0.094 mmol) was added in one portion at ambient temperature. The olive color immediately turned to red. All volatiles were then removed in *vacuo* to yield **3** as a red crystalline solid. Yield: 97% (108 mg). M.p. 141 °C (dec.). Single crystals suitable for X-ray diffraction were grown by storing a saturated *n*-hexane solution of **3** at –30 °C for 12 hours. Anal. calcd. (%) for $C_{66}H_{94}Ga_2N_6OP_2$ (1188.89): C, 66.68; H, 7.97; N, 7.07. Found: C, 66.53; H, 7.93; N, 7.06. ^{1}H NMR (400 MHz, C_6D_6, 298 K) δ = 0.48 (d, $^3J_{HH}$ = 6.8 Hz, 3H, CH(CH_3)$_2$), 0.90 (pst, $^3J_{HH}$ = 6.8 Hz, 3H, CH(CH_3)$_2$), 0.97 (d, $^3J_{HH}$ = 6.6 Hz, 3H, CH(CH_3)$_2$), 1.03 (d, $^3J_{HH}$ = 6.6 Hz, 3H, CH(CH_3)$_2$), 1.14 (d, $^3J_{HH}$ = 6.8 Hz, 3H, CH(CH_3)$_2$), 1.17 (pst, $^3J_{HH}$ = 6.3 Hz, 6H, CH(CH_3)$_2$), 1.24 (br, 6H, CCH_3), 1.30 (d, $^3J_{HH}$ = 6.8 Hz, 3H, CH(CH_3)$_2$), 1.33 (d, $^3J_{HH}$ = 3.2 Hz, 3H, CH(CH_3)$_2$), 1.34 (d, $^3J_{HH}$ = 3.2 Hz, 3H, CH(CH_3)$_2$), 1.38 (d, $^3J_{HH}$ = 6.8 Hz, 3H, CH(CH_3)$_2$), 1.47 (d, $^3J_{HH}$ = 3.2 Hz, 3H, CH(CH_3)$_2$), 1.49 (d, $^3J_{HH}$ = 3.2 Hz, 3H, CH(CH_3)$_2$), 1.53 (s, 3H, CCH_3), 1.54 (s, 3H, CCH_3), 1.61 (s, 3H, CCH_3), 1.67 (s, 3H, CCH_3), 1.73 (pst, $^3J_{HH}$ = 6.8 Hz, 6H, CH(CH_3)$_2$), 1.80 (d, $^3J_{HH}$ = 6.5 Hz, 3H, CH(CH_3)$_2$), 1.83 (d, $^3J_{HH}$ = 6.7 Hz, 3H, CH(CH_3)$_2$), 2.60 (s, 6H, CCH_3), 3.00 (sept, $^3J_{HH}$ = 6.7 Hz, 1H, CH(CH$_3$)$_2$), 3.05 (sept, $^3J_{HH}$ = 6.7 Hz, 1H, CH(CH$_3$)$_2$), 3.43 (sept, $^3J_{HH}$ = 6.7 Hz, 1H, CH(CH$_3$)$_2$), 3.51 (sept, $^3J_{HH}$ = 6.7 Hz, 1H, CH(CH$_3$)$_2$), 3.61 (sept, $^3J_{HH}$ = 6.7 Hz, 1H,

CH(CH$_3$)$_2$), 4.04 (sept, $^3J_{HH}$ = 6.7 Hz, 1H, CH(CH$_3$)$_2$), 4.60 (sept, $^3J_{HH}$ = 6.7 Hz, 1H, CH(CH$_3$)$_2$), 4.69 (sept, $^3J_{HH}$ = 6.7 Hz, 1H, CH(CH$_3$)$_2$), 4.90 (s, 1H, CH), 5.03 (s, 1H, CH), 6.96–6.99 (m, 3H, C$_6H_3$), 7.04–7.07 (m, 2H, C$_6H_3$), 7.11–7.14 (m, 2H, C$_6H_3$), 7.26 (t, $^3J_{HH}$ = 7.6 Hz, 1H, C$_6H_3$), 7.31–7.36 (m, 3H, C$_6H_3$), 7.38–7.40 (m, 1H, C$_6H_3$). ^{13}C{^{1}H} NMR (100 MHz, C$_6$D$_6$, 298 K) δ = 8.0, 14.4 (CH_3), 23.1, 24.2, 24.5, 24.7, 24.8, 24.9, 25.0, 25.1, 25.3, 25.4, 25.5, 25.6, 25.7, 26.1, 26.5 (CH(CH$_3$)$_2$), 27.0, 27.6, 27.7, 28.1, 28.2, 28.4, 29.0, 29.1, 29.3, 29.4, 29.7 (CH(CH$_3$)$_2$), 32.0 (CC), 97.4, 99.4 (CH), 122.9, 123.2, 123.3, 123.5, 123.6, 123.9, 124.0, 125.4, 125.5, 125.6, 125.7, 126.6, 126.8, 142.7, 143.7, 144.8, 145.5, 146.0, 146.2, 146.5, 146.6, 147.0, 147.1, 147.2, 163.7 (dd, J_{CP} = 9.63 and 124.68 Hz, PCPC), 168.2, 168.7, 169.2, 169.9 (C_6H$_3$), 233.1 (dd, J_{CP} = 94.92 and 110.05 Hz, PCPC). ^{31}P{^{1}H} (121 MHz, C$_6$D$_6$, 298 K) δ = 168.59 (d, $^2J_{PP}$ = 18.0 Hz, PCP), 74.73 (d, $^2J_{PP}$ = 18.0 Hz, PCP). ATR-IR: ν 2955, 2922, 2864, 1621, 1524, 1435, 1394, 1315, 1255, 1175, 1098, 1020, 933, 854, 794, 760, 684, 639, 562, 529 cm^{-1}.

Synthesis of compound 4.

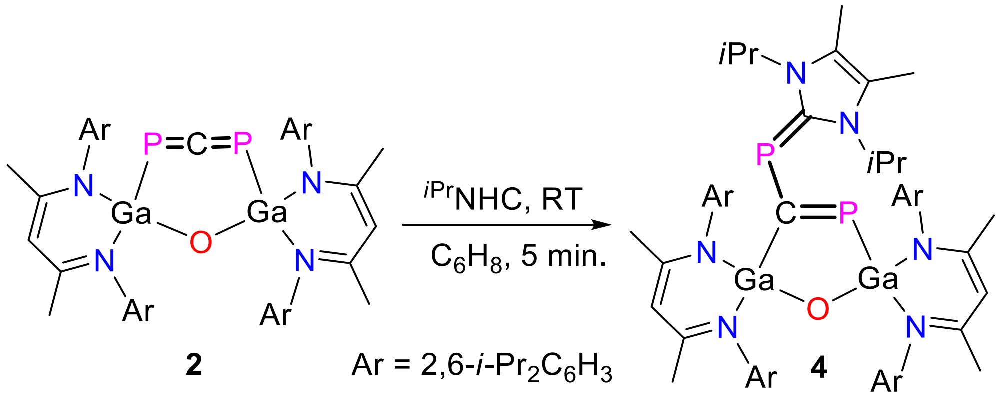

2 Ar = 2,6-i-Pr$_2$C$_6$H$_3$ **4**

To a toluene (5 mL) solution of **2** (100 mg, 0.094 mmol), ^{iPr}NHC (17 mg, 0.094 mmol) was added in one portion at ambient temperature. The olive color immediately turned to red. All volatiles were then removed in *vacuo* to yield **4** as a red crystalline solid. Yield: 98% (114 mg). M.p. 147 °C (dec.). Single crystals suitable for X-ray diffraction were grown by diffusing n-hexane into a saturated benzene solution of **4** at ambient temperature. Anal. calcd. (%) for C$_{70}$H$_{102}$Ga$_2$N$_6$OP$_2$ (1244.99): C, 67.53; H, 8.26; N, 6.75. Found: C, 67.58; H, 8.31; N, 6.81. ^{1}H NMR (300 MHz, C$_6$D$_6$, 298 K) δ = 0.45 (d, $^3J_{HH}$ = 6.8 Hz, 3H, CH(CH_3)$_2$), 0.87 (br, 6H, CH(CH_3)$_2$), 1.02 (d, $^3J_{HH}$ = 6.8 Hz, 3H, CH(CH_3)$_2$), 1.04 (d, $^3J_{HH}$ = 6.8 Hz, 3H, CH(CH_3)$_2$), 1.13 (d, $^3J_{HH}$ = 6.8 Hz, 3H, CH(CH_3)$_2$), 1.18-1.20 (m, 6H, CH(CH_3)$_2$) 1.29 (d, $^3J_{HH}$ = 6.8 Hz, 3H, CH(CH_3)$_2$), 1.32 (d, $^3J_{HH}$ = 6.8 Hz, 6H, CH(CH_3)$_2$), 1.40 (br, 6H, CCH_3), 1.46-1.49 (m, 9H, CH(CH_3)$_2$) 1.50 (s, 3H, CCH_3), 1.53 (s, 3H, CCH_3), 1.60 (s, 6H, CCH_3), 1.62 (d, $^3J_{HH}$ = 6.8 Hz, 3H, CH(CH_3)$_2$), 1.17 (d, $^3J_{HH}$ = 6.8 Hz, 3H, CH(CH_3)$_2$), 1.72 (d, $^3J_{HH}$ = 6.8 Hz, 3H, CH(CH_3)$_2$), 1.80 (d, $^3J_{HH}$ = 6.8 Hz, 3H, CH(CH_3)$_2$), 2.96 (sept, $^3J_{HH}$ = 6.8 Hz, 1H,

CH(CH$_3$)$_2$), 3.08 (sept, $^3J_{HH}$ = 6.8 Hz, 1H, CH(CH$_3$)$_2$), 3.31 (sept, $^3J_{HH}$ = 6.8 Hz, 0.5H, CH(CH$_3$)$_2$), 3.44 (sept, $^3J_{HH}$ = 6.8 Hz, 1.5H, CH(CH$_3$)$_2$), 3.57 (sept, $^3J_{HH}$ = 6.8 Hz, 2.5H, CH(CH$_3$)$_2$), 4.09 (sept, $^3J_{HH}$ = 6.8 Hz, 1.5H, CH(CH$_3$)$_2$), 4.54 (sept, $^3J_{HH}$ = 6.8 Hz, 1H, CH(CH$_3$)$_2$), 4.63 (sept, $^3J_{HH}$ = 6.8 Hz, 1H, CH(CH$_3$)$_2$), 4.96 (s, 1H, CH), 5.06 (s, 1H, CH), 6.95 (d, $^3J_{HH}$ = 9.1 Hz, 1H, C_6H_3), 7.98-7.02 (m, 4H, C_6H_3), 7.07 (d, $^3J_{HH}$ = 7.6 Hz, 1H, C_6H_3), 7.23 (d, $^3J_{HH}$ = 9.2 Hz, 1H, C_6H_3), 7.28 (d, $^3J_{HH}$ = 7.6 Hz, 1H, C_6H_3), 7.31 (t, $^3J_{HH}$ = 7.6 Hz, 2H, C_6H_3), 7.35 (d, $^3J_{HH}$ = 8.0 Hz, 1H, C_6H_3), 7.39 (d, $^3J_{HH}$ = 9.2 Hz, 1H, C_6H_3). $^{13}C\{^1H\}$ NMR (125 MHz, C_6D_6, 298 K) δ = 10.2 (CH_3), 24.3, 24.5, 24.7, 24.8, 25.1, 25.2, 25.3, 25.5, 25.6, 25.7, 25.8, 26.2, 26.4, 26.5 (CH(CH$_3$)$_2$), 27.0, 27.9, 28.1, 28.3, 28.4, 28.8, 29.0, 29.1, 29.4, 29.8 (CH(CH$_3$)$_2$), 51.7 (CC), 98.5, 100.2 (CH), 123.4, 123.7, 123.8, 123.9, 124.0, 125.7, 125.9, 126.0, 126.2, 126.9, 127.0, 142.8, 143.7, 145.0, 146.1, 146.2, 146.4, 146.5, 146.6, 146.9, 147.0, 147.1, 147.2, 161.5 (dd, J_{CP} = 6.23 and 129.85 Hz, PCPC), 168.6, 169.1, 169.8, 170.2 (C_6H$_3$), 231.5 (dd, J_{CP} = 97.10 and 110.96 Hz, PCPC). $^{31}P\{^1H\}$ (121 MHz, C_6D_6, 298 K) δ = 153.34 (d, $^2J_{PP}$ = 22.1 Hz, PCP), 73.94 (d, $^2J_{PP}$ = 20.1 Hz, PCP). ATR-IR: ν 2958, 2925, 2865, 1733, 1551, 1525, 1459, 1434, 1391, 1315, 1253, 1173, 1098, 1018, 936, 853, 795, 761, 685, 639, 557 cm^{-1}.

Synthesis of compound 5.

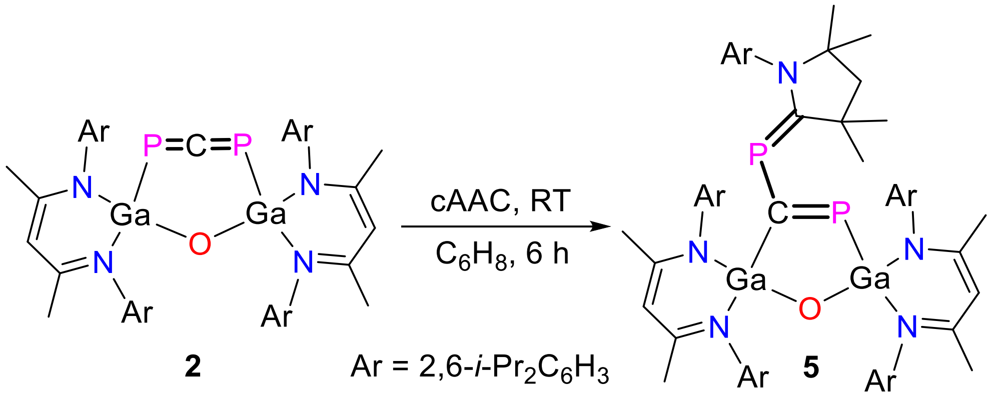

To a toluene (5 mL) solution of **2** (100 mg, 0.094 mmol), cAAC·LiOTf (42 mg, 0.094 mmol) was added in one portion at ambient temperature and the resulting reaction mixture was stirred for 6 hours. During this time the olive color turned to wine-red. All volatiles were then removed in *vacuo* and the residue was dissolved in *n*-hexane (5 mL) and filtered through a plug of celite. After drying the filterate in *vacuo*, compound **5** was isolated as a wine-red solid in 93% (118 mg) yield. M.p. 143 °C (dec.). Anal. calcd. (%) for $C_{79}H_{113}Ga_2N_5OP_2$ (1350.17): C, 70.28; H, 8.44; N, 5.19. Found: C, 70.31; H, 8.45; N, 5.19. ^1H NMR (400 MHz, C_6D_6, 298 K) δ = 0.78 (d, $^3J_{HH}$ = 6.8 Hz, 6H, CH(CH_3)$_2$), 1.12 (pst, $^3J_{HH}$ = 6.8 Hz, 12H, CH(CH_3)$_2$), 1.18 (d, $^3J_{HH}$ = 6.8 Hz, 12H, CH(CH_3)$_2$), 1.21 (s, 6H, CH_3), 1.23 (d, $^3J_{HH}$ = 6.8 Hz, 6H, CH(CH_3)$_2$), 1.30 (d, $^3J_{HH}$ = 6.8 Hz, 6H, CH(CH_3)$_2$), 1.37 (d, $^3J_{HH}$ = 6.8 Hz, 6H, CH(CH_3)$_2$), 1.42 (s, 6H, CCH_3), 1.47 (s, 6H, CCH_3), 1.48 (d, $^3J_{HH}$ = 6.8 Hz, 6H, CH(CH_3)$_2$), 1.60 (d, $^3J_{HH}$ = 6.8 Hz, 6H, CH(CH_3)$_2$), 2.17 (s, 3H, CH_3), 2.19 (s, 3H, CH_3), 2.97 (sept, $^3J_{HH}$ = 6.8

Hz, 2H, CH(CH$_3$)$_2$), 3.22 (sept, $^3J_{HH}$ = 6.8 Hz, 4H, CH(CH$_3$)$_2$), 3.50 (sept, $^3J_{HH}$ = 6.8 Hz, 2H, CH(CH$_3$)$_2$), 3.69 (sept, $^3J_{HH}$ = 6.8 Hz, 2H, CH(CH$_3$)$_2$), 4.84 (s, 1H, CH), 4.88 (s, 1H, CH), 6.96 (d, $^3J_{HH}$ = 7.6 Hz, 2H, C$_6H_3$), 7.01 (d, $^3J_{HH}$ = 7.6 Hz, 2H, C$_6H_3$), 7.05–7.08 (m, 4H, C$_6H_3$), 7.12–7.14 (m, 3H, C$_6H_3$), 7.18–7.22 (m, 5H, C$_6H_3$). ^{13}C{^{1}H} NMR (100 MHz, C$_6$D$_6$, 298 K) δ = 14.4 (CH_3), 21.4, 24.1, 24.8, 25.0, 25.2, 25.7, 26.4 (CH(CH$_3$)$_2$), 27.4, 28.4, 28.8, 29.0, 29.2, 30.6, 30.8 (CH(CH$_3$)$_2$), 50.7, 50.8, 56.8, 68.9 (CC), 96.4, 99.8 (CH), 124.4, 124.6, 124.8, 125.7, 126.3, 126.4, 128.6, 129.3, 137.6, 137.9, 143.0, 143.2, 143.4, 143.6, 144.3, 144.8, 147.9, 169.4, 169.7 (C_6H$_3$), 200.8 (dd, J_{CP} = 12.54 and 112.83 Hz, PCPC), 249.3 (dd, J_{CP} = 113.34 and 121.01 Hz, PCPC). ^{31}P{^{1}H} (121 MHz, C$_6$D$_6$, 298 K) δ = 434.01 (d, $^2J_{PP}$ = 53.47 Hz, PCP), 143.46 (d, $^2J_{PP}$ = 52.74 Hz, PCP). ATR-IR: ν 2959, 2929, 2866, 1623, 1549, 1526, 1437, 1384, 1363, 1317, 1257, 1199, 1177, 1141, 1098, 1019, 934, 864, 796, 757, 670, 620 cm^{-1}.

Synthesis of compound 6.

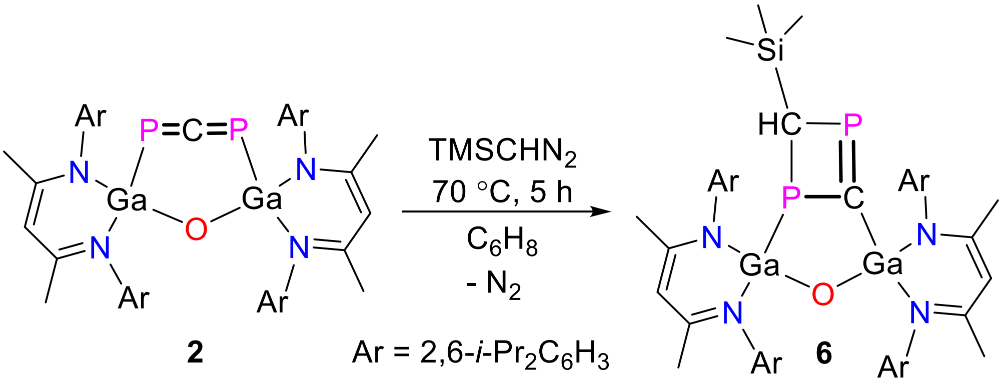

2 Ar = 2,6-*i*-Pr$_2$C$_6$H$_3$ **6**

To a toluene (5 mL) solution of **2** (100 mg, 0.094 mmol), TMSCHN$_2$ (157 µL, 0.094 mmol, 0.6 M in *n*-hexane) was added in one portion at ambient temperature and the resulting mixture was then heated to 70 °C for 3 hours. During this time the olive color turned to orange. All volatiles were then removed in *vacuo* to yield **6** as an orange solid. Yield: 93% (100 mg). M.p. 137 °C (dec.). Single crystals suitable for X-ray diffraction were grown by storing a saturated *n*-hexane solution of **6** at ambient temperature for 12 hours. Anal. calcd. (%) for C$_{63}$H$_{92}$Ga$_2$N$_4$OP$_2$Si (1150.91): C, 65.75; H, 8.06; N, 4.87. Found: C, 65.73; H, 8.01; N, 4.84. ^{1}H NMR (400 MHz, C$_6$D$_6$, 298 K) δ = –0.02 (s, 9H, Si(CH_3)$_3$), 0.74 (d, $^3J_{HH}$ = 6.8 Hz, 3H, CH(CH_3)$_2$), 0.83 (br, 1H, CHSi(CH$_3$)$_3$) 0.90 (pst, $^3J_{HH}$ = 6.8 Hz, 6H, CH(CH_3)$_2$), 0.92 (d, $^3J_{HH}$ = 6.8 Hz, 3H, CH(CH_3)$_2$), 0.93 (d, $^3J_{HH}$ = 6.8 Hz, 3H, CH(CH_3)$_2$), 1.01 (d, $^3J_{HH}$ = 6.8 Hz, 3H, CH(CH_3)$_2$), 1.07 (d, $^3J_{HH}$ = 6.8 Hz, 3H, CH(CH_3)$_2$), 1.11 (d, $^3J_{HH}$ = 6.8 Hz, 3H, CH(CH_3)$_2$), 1.13 (d, $^3J_{HH}$ = 6.8 Hz, 3H, CH(CH_3)$_2$), 1.16 (d, $^3J_{HH}$ = 6.8 Hz, 6H, CH(CH_3)$_2$), 1.24 (br, 6H, CCH_3), 1.29 (d, $^3J_{HH}$ = 6.8 Hz, 3H, CH(CH_3)$_2$), 1.38 (d, $^3J_{HH}$ = 6.8 Hz, 3H, CH(CH_3)$_2$), 1.40 (s, 3H, CCH_3), 1.41 (d, $^3J_{HH}$ = 6.8 Hz, 3H, CH(CH_3)$_2$), 1.43 (d, $^3J_{HH}$ = 6.8 Hz, 3H, CH(CH_3)$_2$), 1.44 (s, 3H, CCH_3), 1.49 (s, 3H, CCH_3), 1.51 (s, 3H, CCH_3), 1.60 (d, $^3J_{HH}$ = 6.5 Hz, 3H, CH(CH_3)$_2$), 1.63 (d,

${}^{3}J_{HH}$ = 6.5 Hz, 3H, CH(CH_3)$_2$), 1.65 (d, ${}^{3}J_{HH}$ = 6.5 Hz, 3H, CH(CH_3)$_2$), 2.77 (sept, ${}^{3}J_{HH}$ = 6.7 Hz, 1H, CH(CH$_3$)$_2$), 3.96 (sept, ${}^{3}J_{HH}$ = 6.7 Hz, 1H, CH(CH$_3$)$_2$), 3.02 (sept, ${}^{3}J_{HH}$ = 6.7 Hz, 1H, CH(CH$_3$)$_2$), 3.13 (sept, ${}^{3}J_{HH}$ = 6.7 Hz, 1H, CH(CH$_3$)$_2$), 3.27 (sept, ${}^{3}J_{HH}$ = 6.7 Hz, 1H, CH(CH$_3$)$_2$), 3.37 (sept, ${}^{3}J_{HH}$ = 6.7 Hz, 1H, CH(CH$_3$)$_2$), 4.27 (sept, ${}^{3}J_{HH}$ = 6.7 Hz, 1H, CH(CH$_3$)$_2$), 4.41 (sept, ${}^{3}J_{HH}$ = 6.7 Hz, 1H, CH(CH$_3$)$_2$), 4.82 (s, 1H, CH), 4.83 (s, 1H, CH), 6.98 (d, ${}^{3}J_{HH}$ = 5.0 Hz, 1H, C$_6$$H_3$), 7.10–7.13 (m, 5H, C$_6$$H_3$), 7.17–7.27 (m, 5H, C$_6$$H_3$), 7.28–7.32 (m, 1H, C$_6$$H_3$). ^{13}C{^{1}H} NMR (100 MHz, C$_6$D$_6$, 298 K) δ = −1.4, 1.4, 14.4 (CH_3), 23.1, 23.9, 24.3, 24.4, 24.5, 24.7, 25.2, 25.3, 25.5, 266, 26.9 (CH(CH$_3$)$_2$), 28.0, 28.1, 28.9, 29.1, 29.3, 29.5 (CH(CH$_3$)$_2$), 32.0 (CCH), 35.1 (dd, J_{CP} = 28.05 and 36.59 Hz, PCPCH), 97.2, 97.3 (CH), 123.6, 123.8, 124.2, 124.3, 124.4, 124.5, 125.4, 125.7, 127.2, 127.3, 142.4, 143.3, 143.5, 143.8, 144.0, 144.1, 144.3, 144.5, 144.6, 145.3, 46.7, 146.8, 168.4, 169.0, 169.8, 171.0 (C_6H$_3$), 244.94 (dd, J_{CP} = 51.55 and 77.77 Hz, PCPC). ^{31}P{^{1}H} (161 MHz, C$_6$D$_6$, 298 K) δ = 386.48 (d, ${}^{2}J_{PP}$ = 96.8 Hz, PP), 89.30 (d, ${}^{2}J_{PP}$ = 96.8 Hz, PP). ATR-IR: v 2957, 2922, 2866, 1528, 1437, 1389, 1315, 1257, 1176, 1098, 1019, 934, 854, 796, 759, 711, 637, 527 cm^{-1}.

2. Spectroscopic Characterization

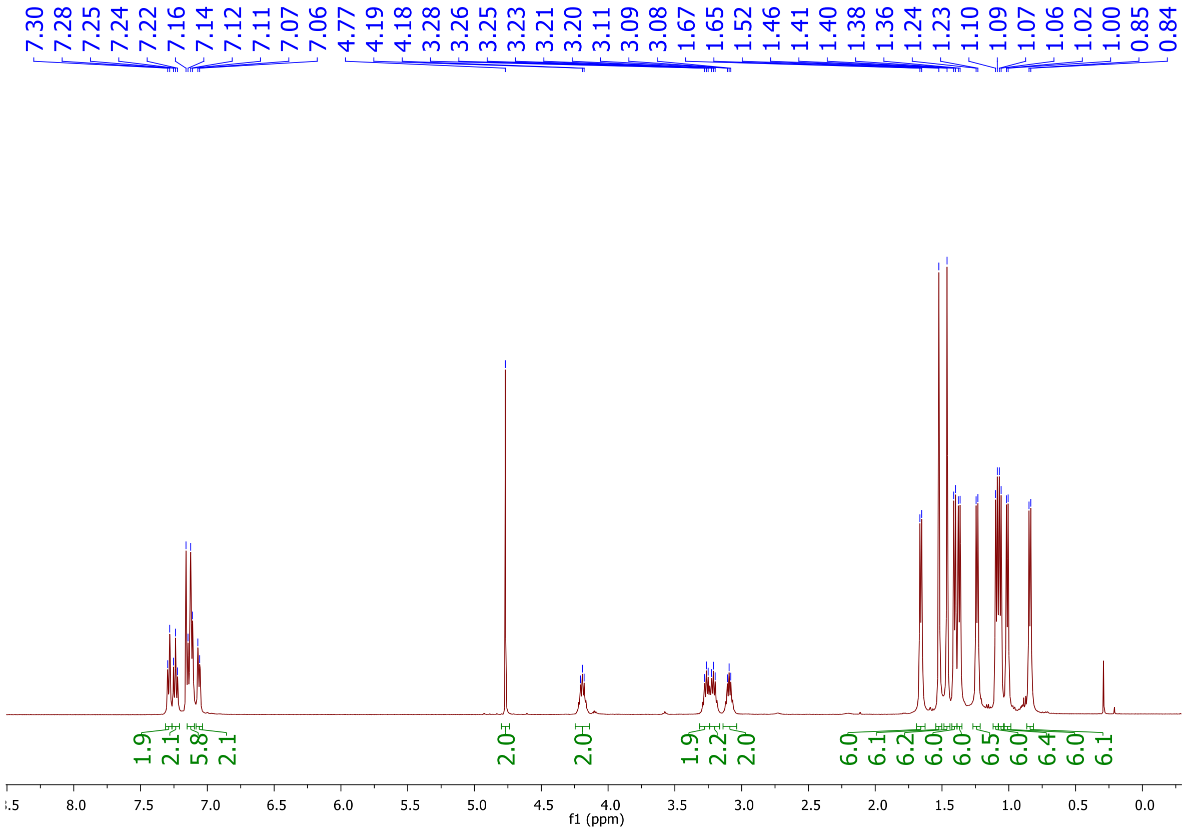

Figure S1. ^{1}H NMR (300 MHz, C_6D_6, 298 K) spectrum of compound **2**.

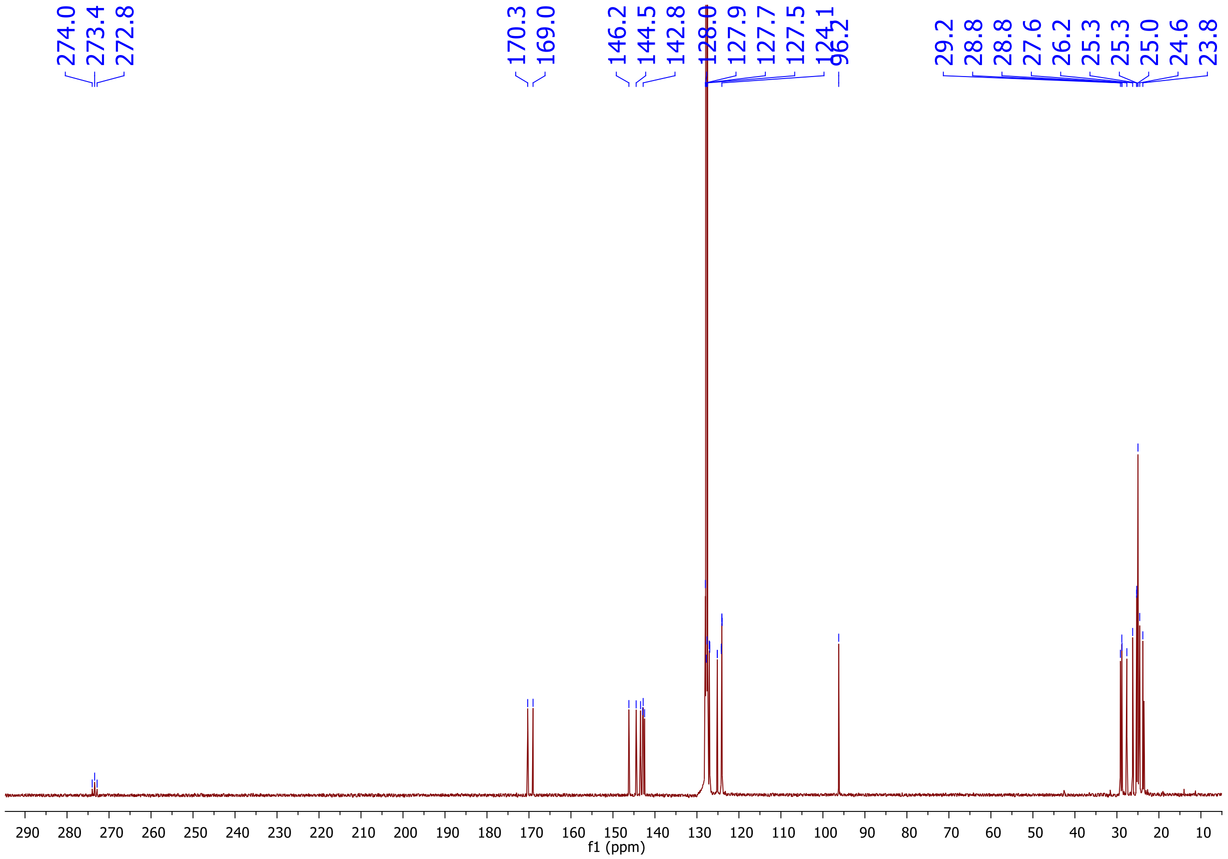

Figure S2. ^{13}C{^{1}H} NMR (75 MHz, C_6D_6, 298 K) spectrum of compound **2**.

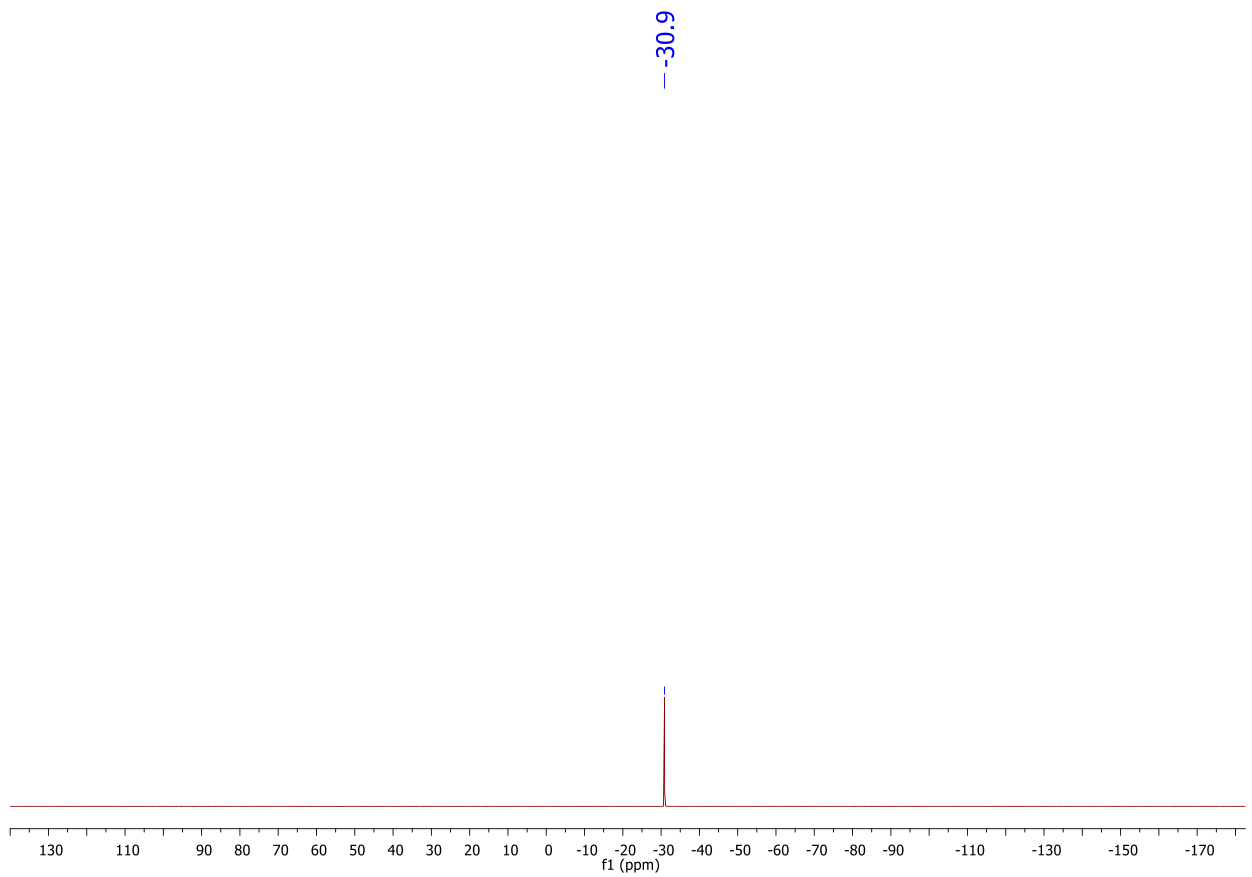

Figure S3. $^{31}P\{^{1}H\}$ NMR (121 MHz, C_6D_6, 298 K) spectrum of compound **2**.

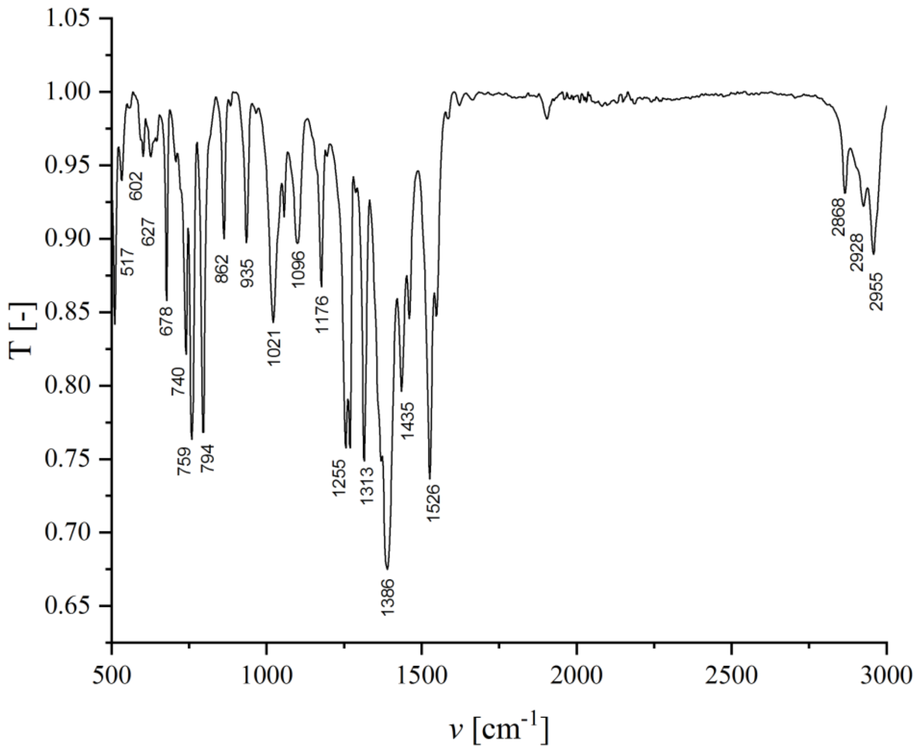

Figure S4. ATR-IR spectrum of compound **2**.

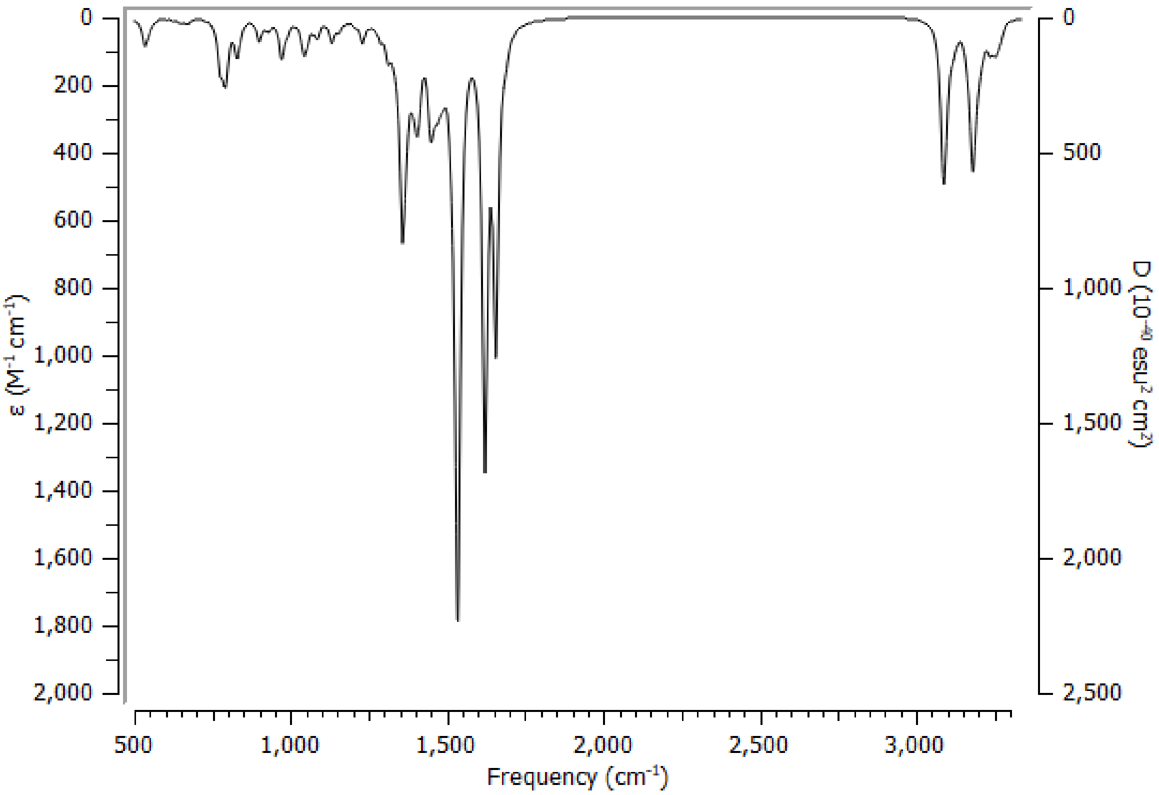

Figure S5. IR spectrum of compound **2** simulated by means of PBE0-D3BJ/def2-SVP. The asymmetric stretching frequency for the 1,3-diphosphaallene unit is calculated to a value of 1347 cm^{-1}.

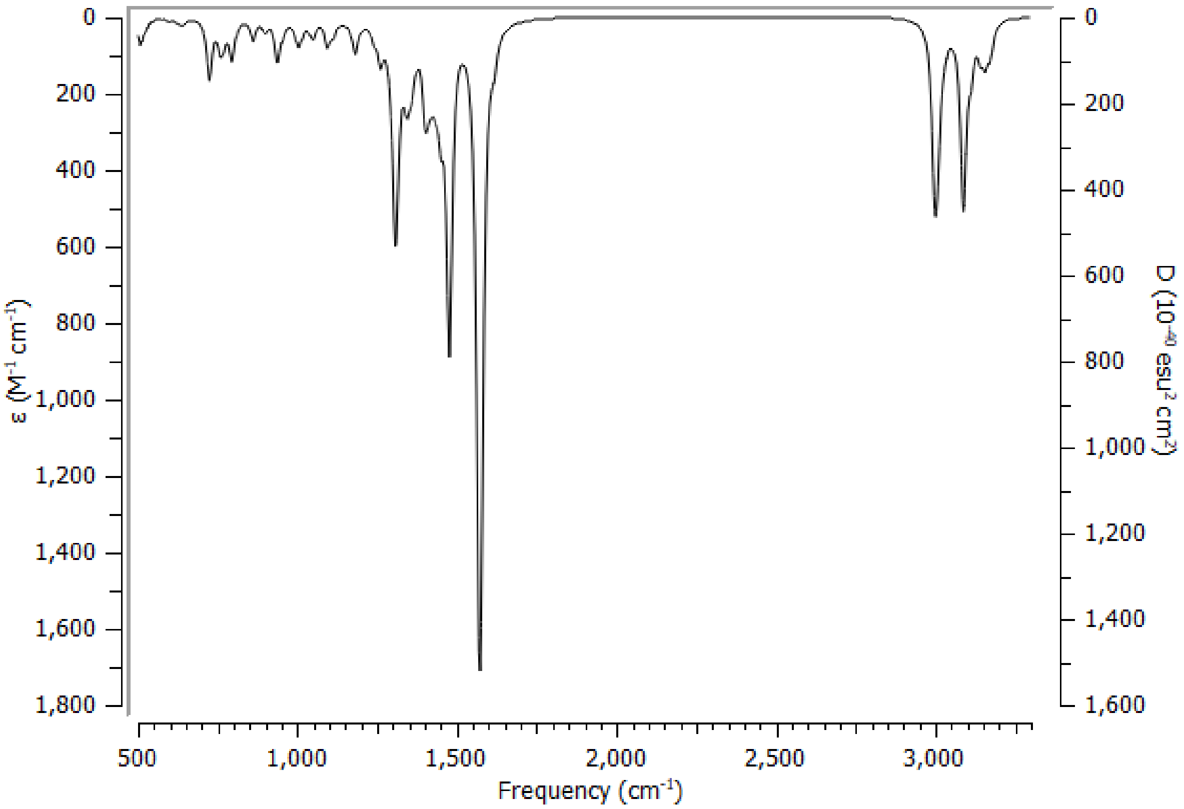

Figure S6. IR spectrum of compound **2** simulated by means of PBE-D3BJ/def2-SVP. The asymmetric stretching frequency for the 1,3-diphosphaallene unit is calculated to a value of 1297 cm^{-1}.

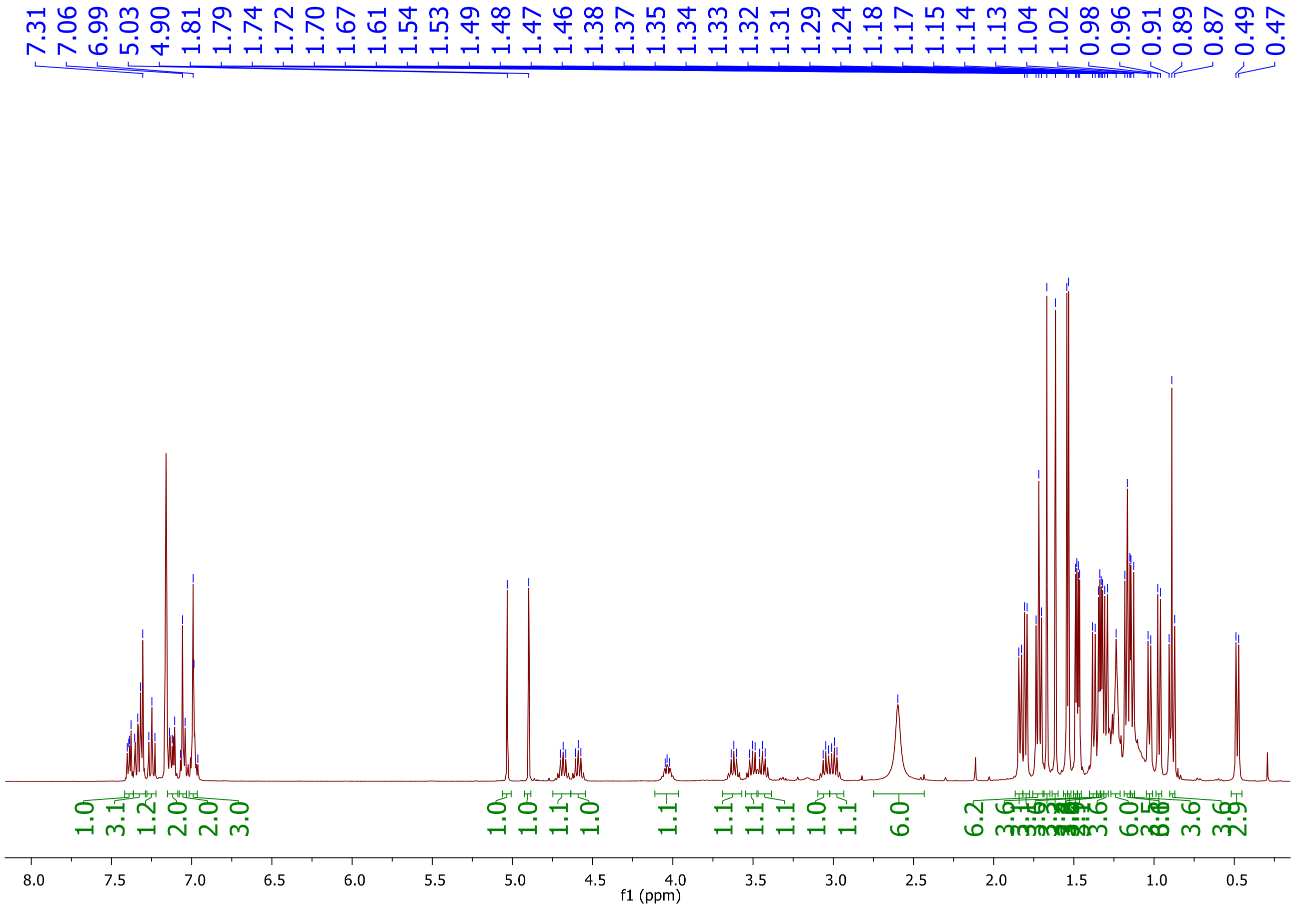

Figure S7. ^{1}H NMR (400 MHz, C_6D_6, 298 K) spectrum of compound **3**.

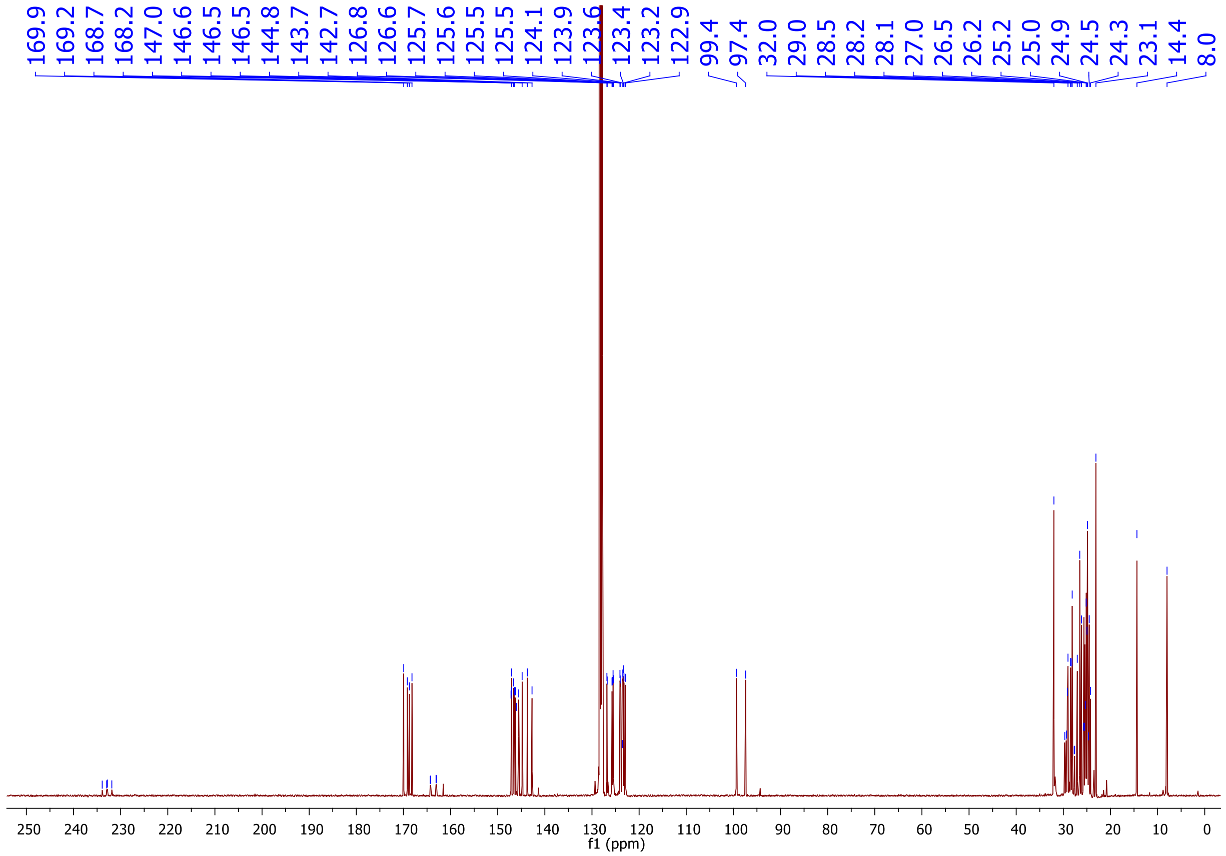

Figure S8. $^{13}C\{^1H\}$ NMR (100 MHz, C_6D_6, 298 K) spectrum of compound **3**.

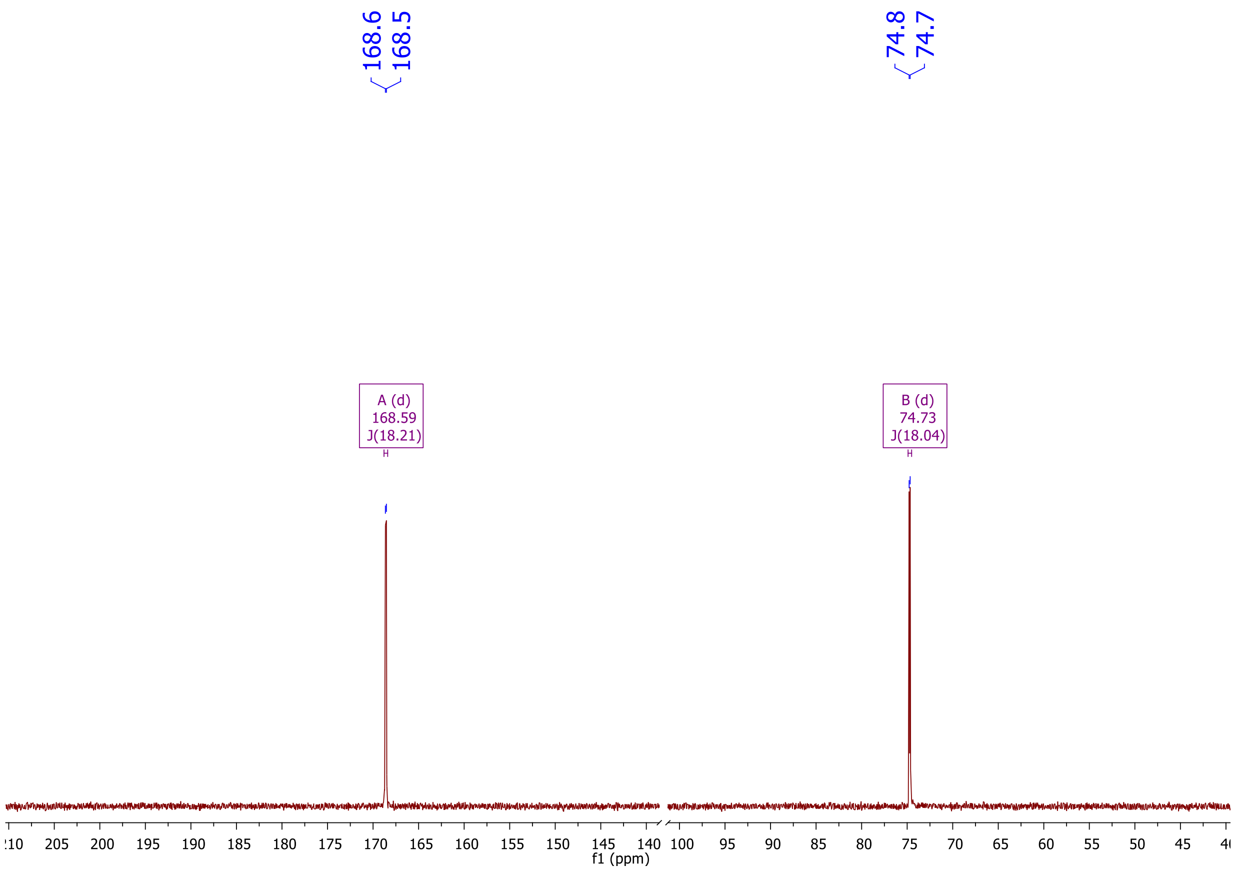

Figure S9. $^{31}P\{^{1}H\}$ NMR (161 MHz, C_6D_6, 298 K) spectrum of compound **3**.

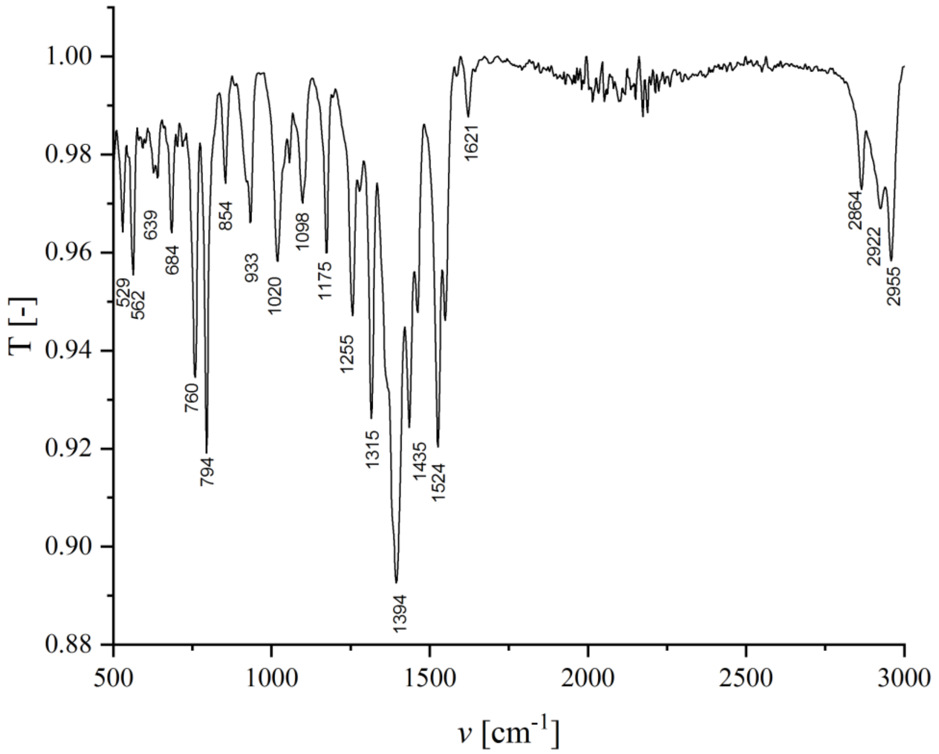

Figure S10. ATR-IR spectrum of compound **3**.

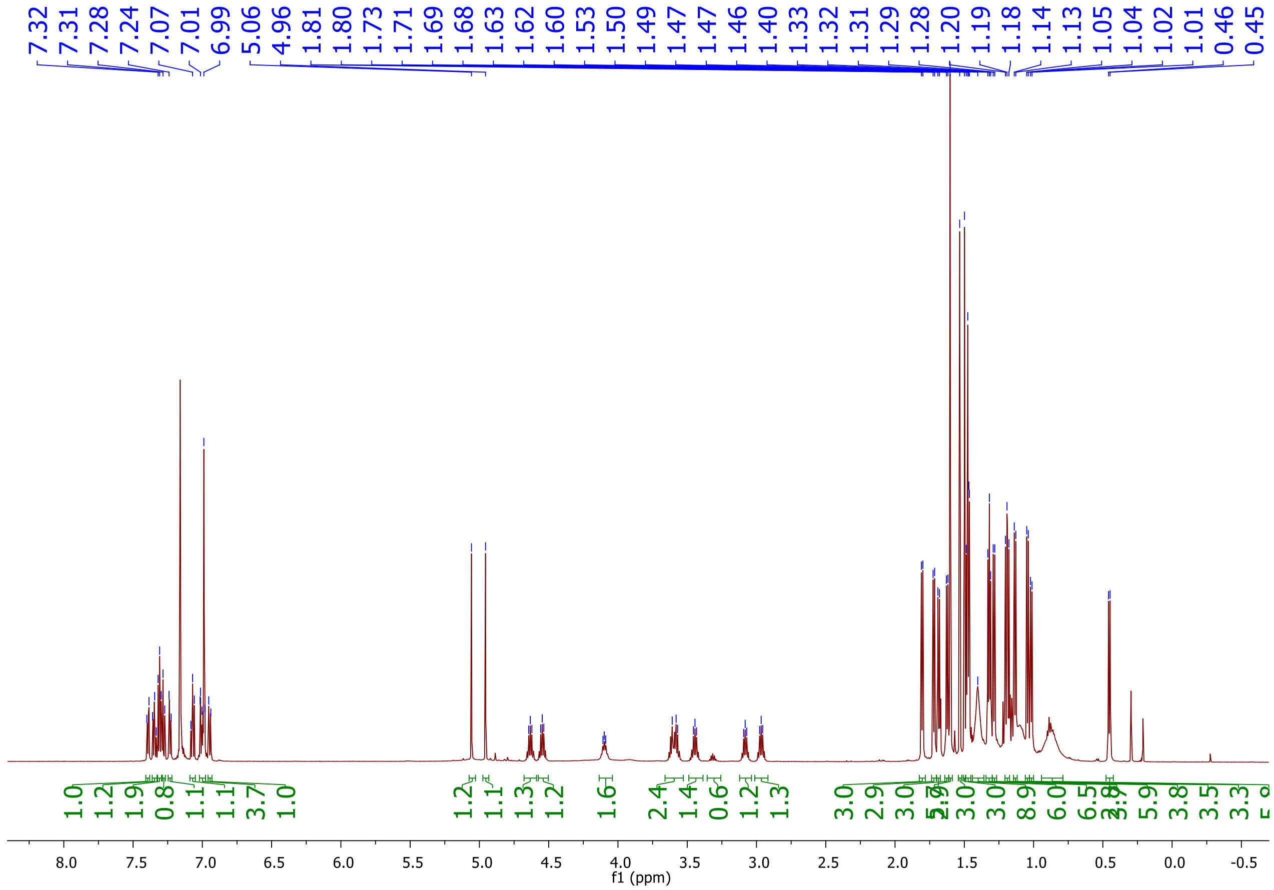

Figure S11. ^{1}H NMR (400 MHz, C_6D_6, 298 K) spectrum of compound **4**.

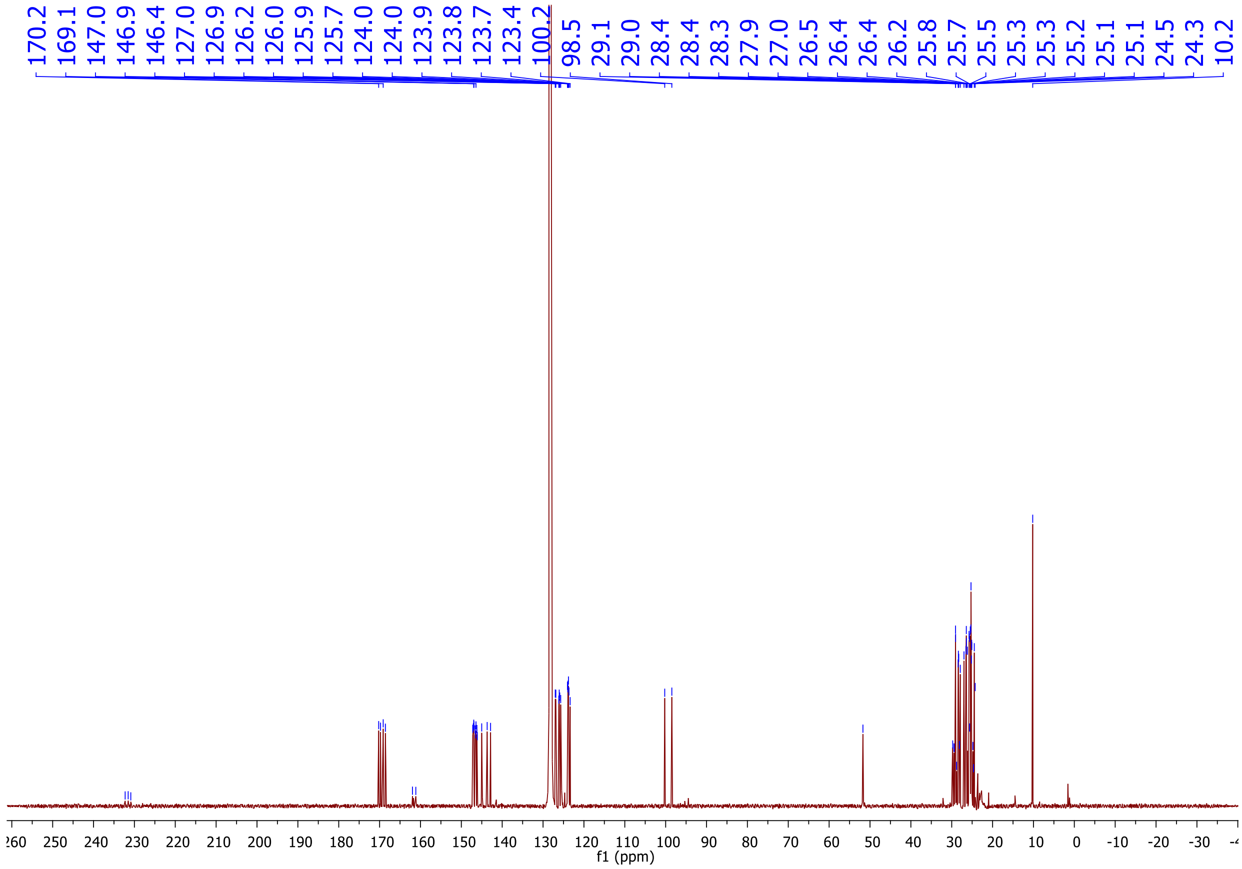

Figure S12. ^{13}C{^{1}H} NMR (100 MHz, C_6D_6, 298 K) spectrum of compound **4**.

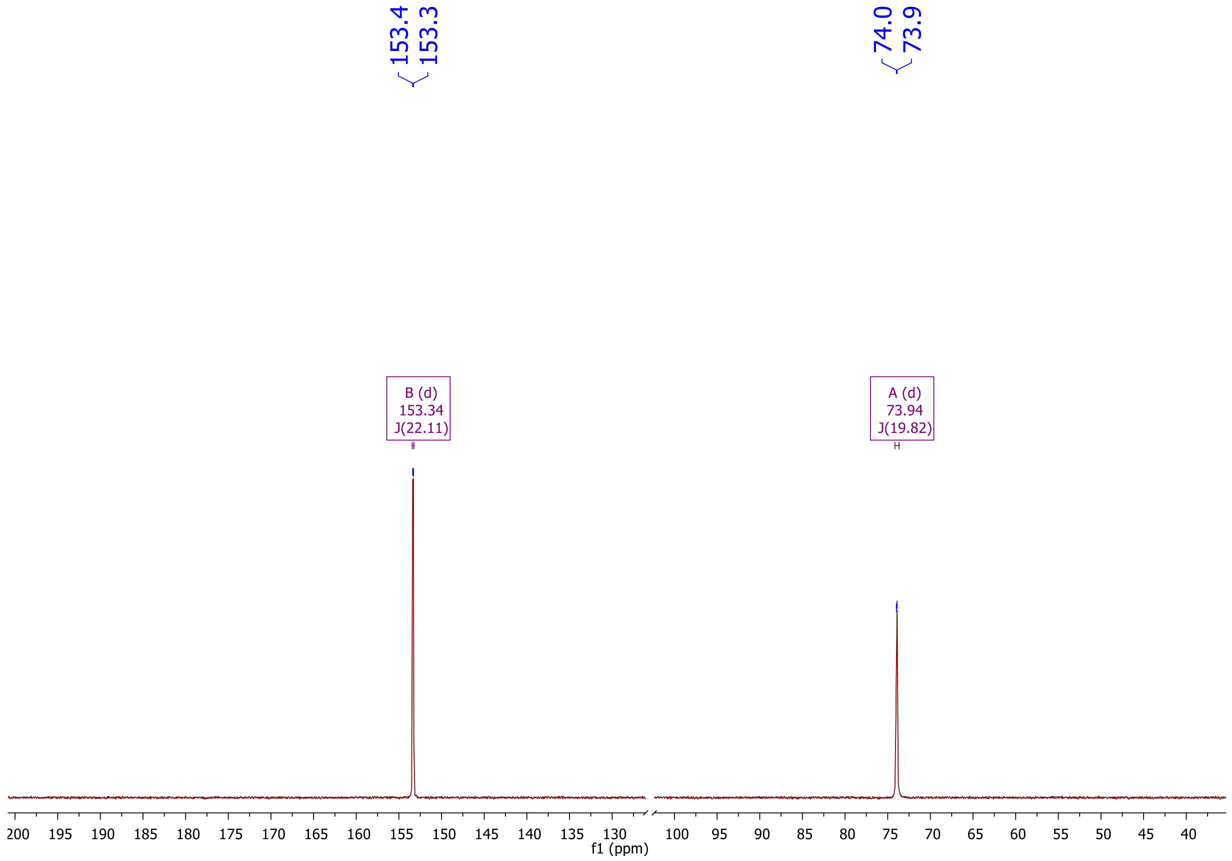

Figure S13. $^{31}P\{^{1}H\}$ NMR (161 MHz, C_6D_6, 298 K) spectrum of compound **4**.

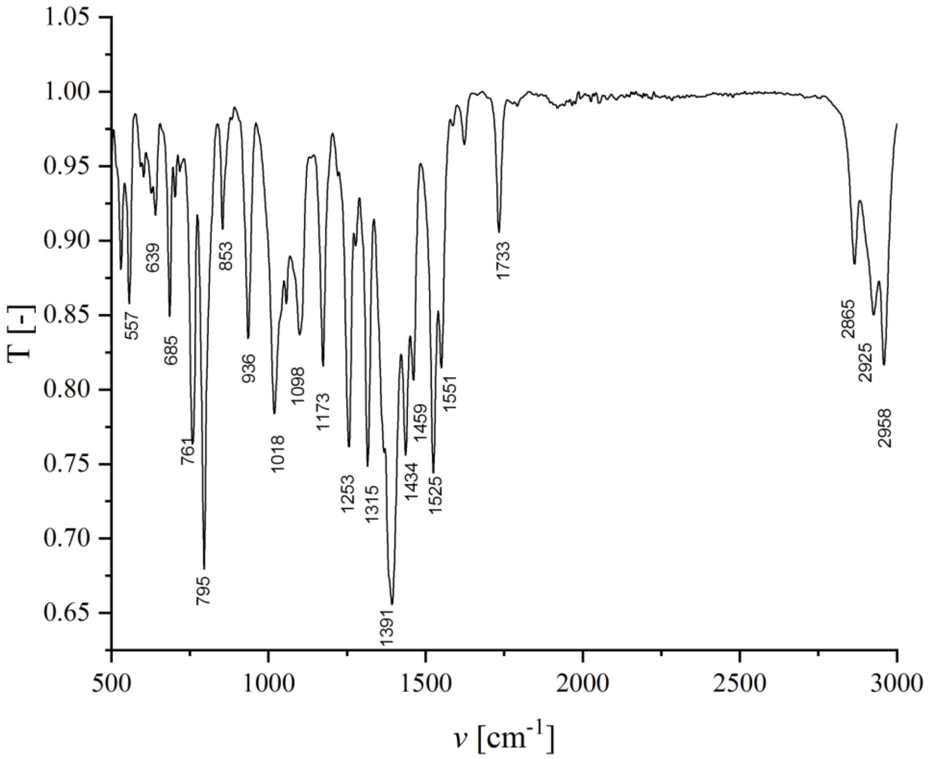

Figure S14. ATR-IR spectrum of compound **4**.

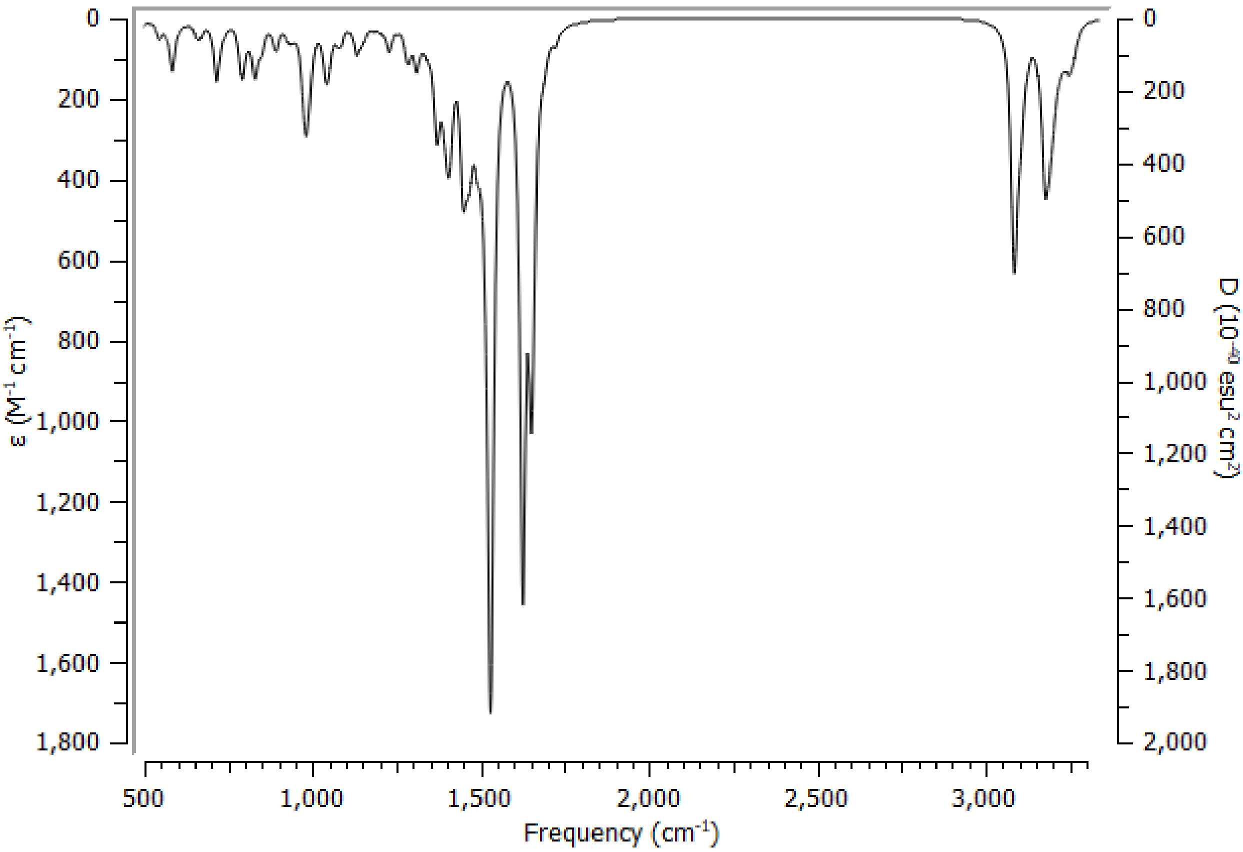

Figure S15. IR spectrum of compound **4** simulated by means of PBE0-D3BJ/def2-SVP.

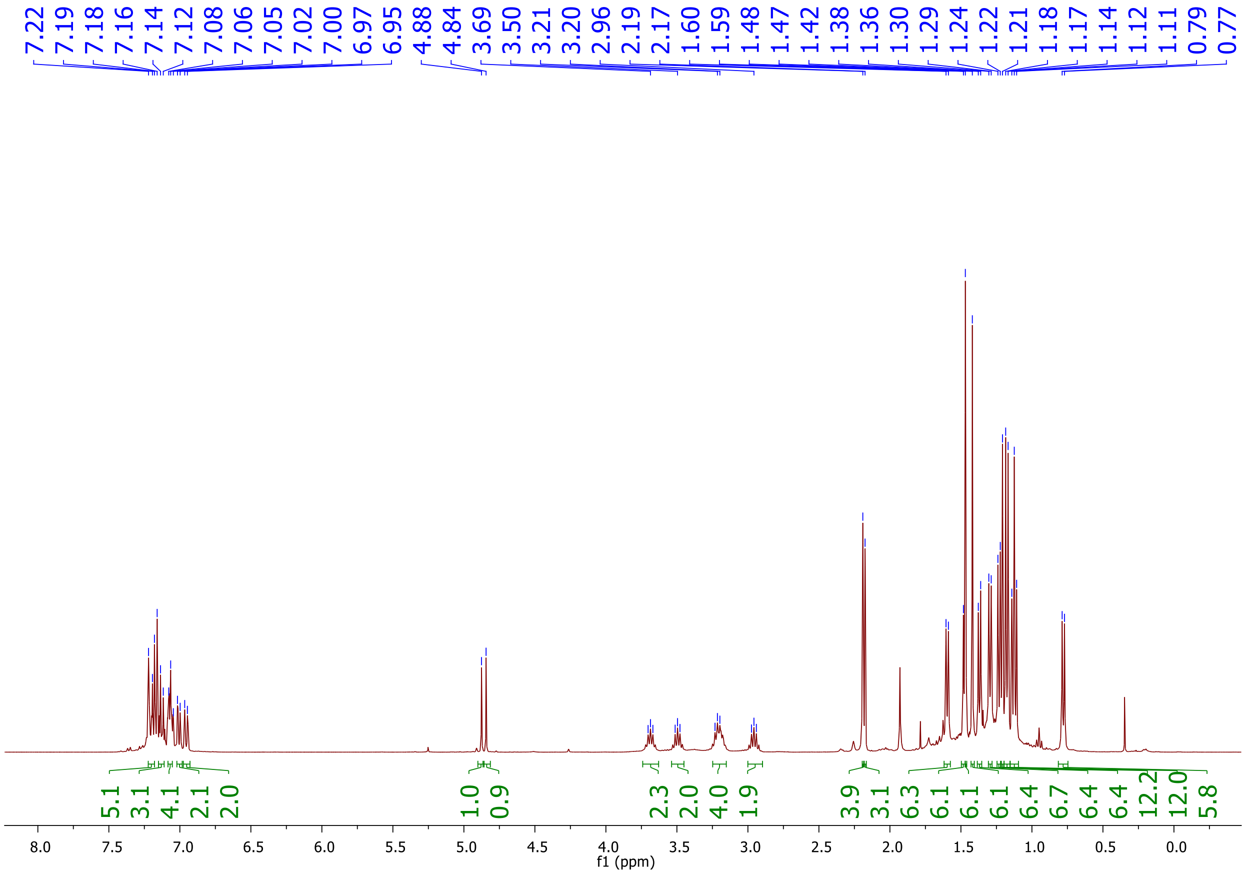

Figure S16. ^{1}H NMR (400 MHz, C_6D_6, 298 K) spectrum of compound **5**.

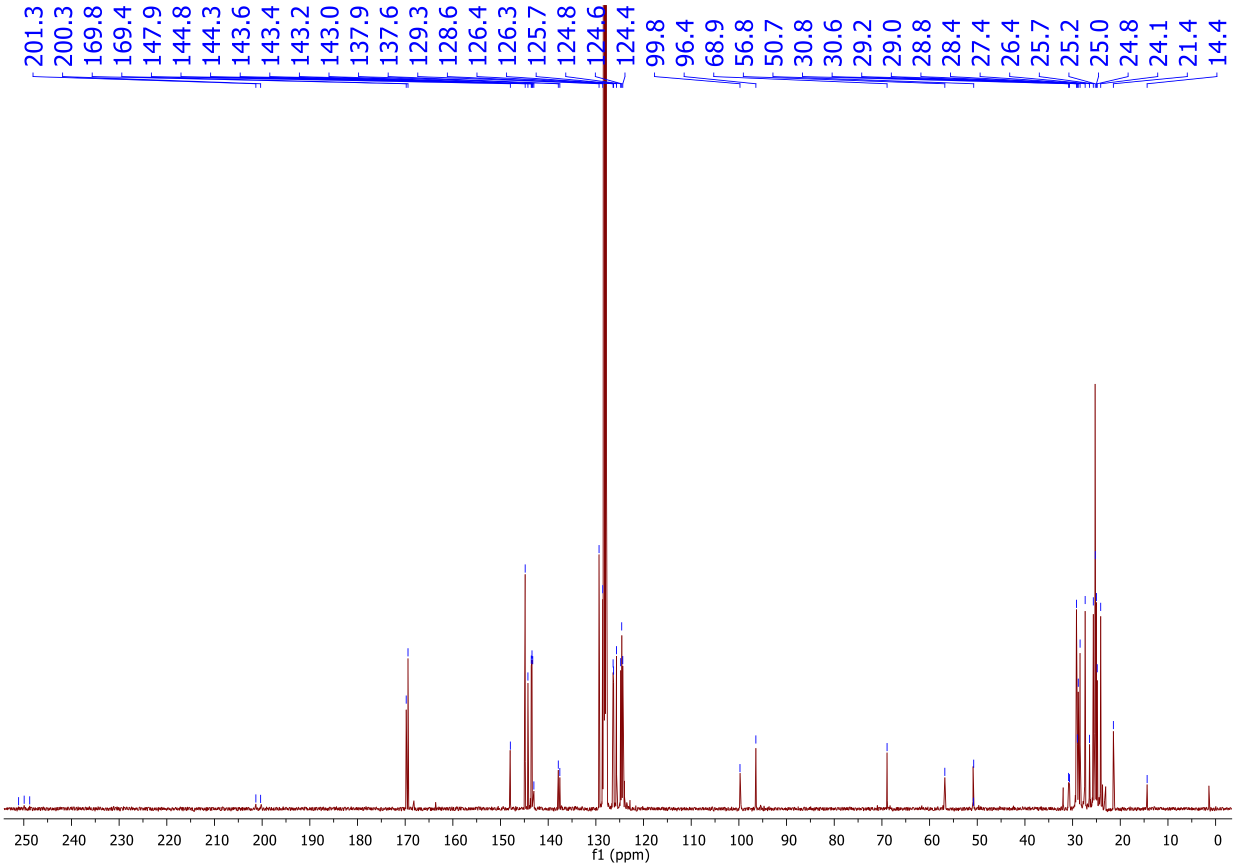

Figure S17. $^{13}C\{^{1}H\}$ NMR (100 MHz, C_6D_6, 298 K) spectrum of compound **5**.

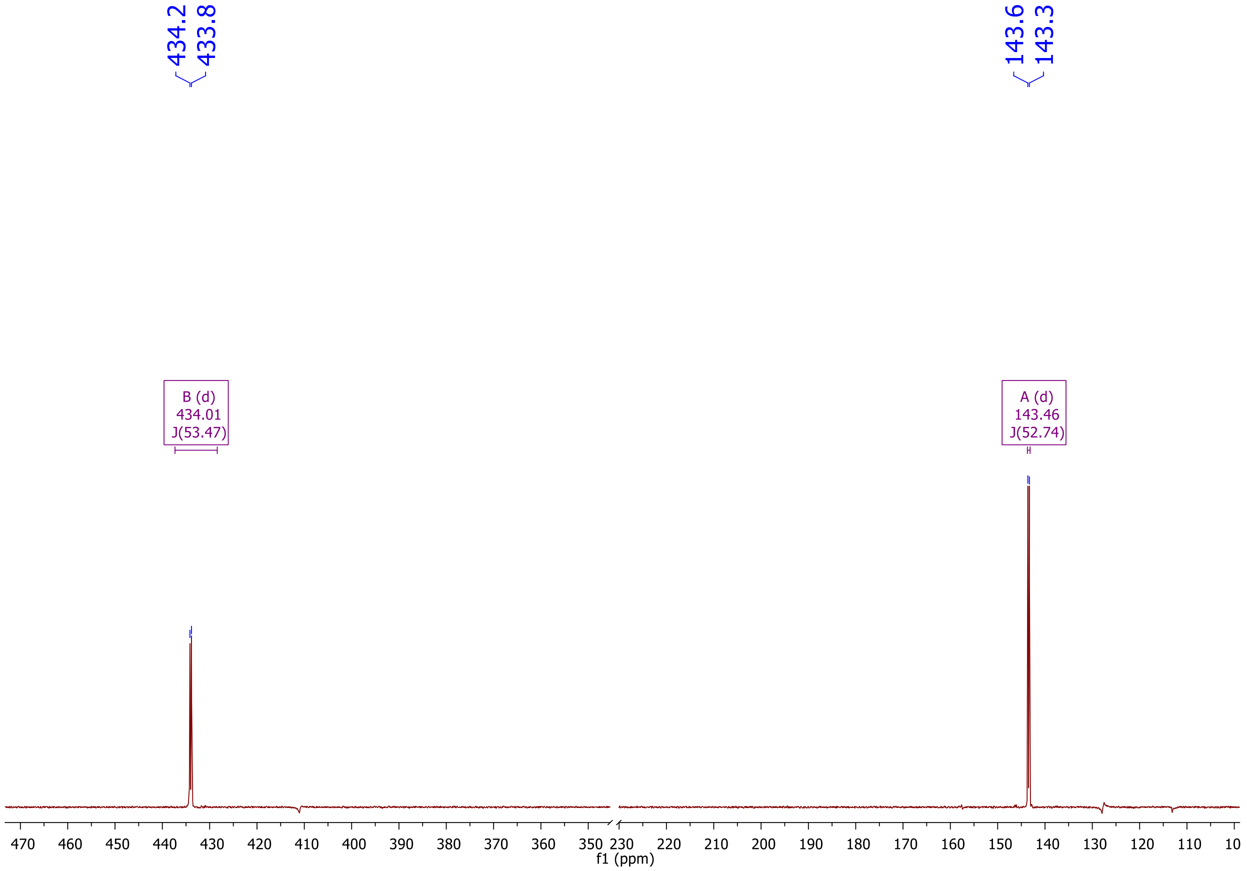

Figure S18. $^{31}P\{^{1}H\}$ NMR (161 MHz, C_6D_6, 298 K) spectrum of compound **5**.

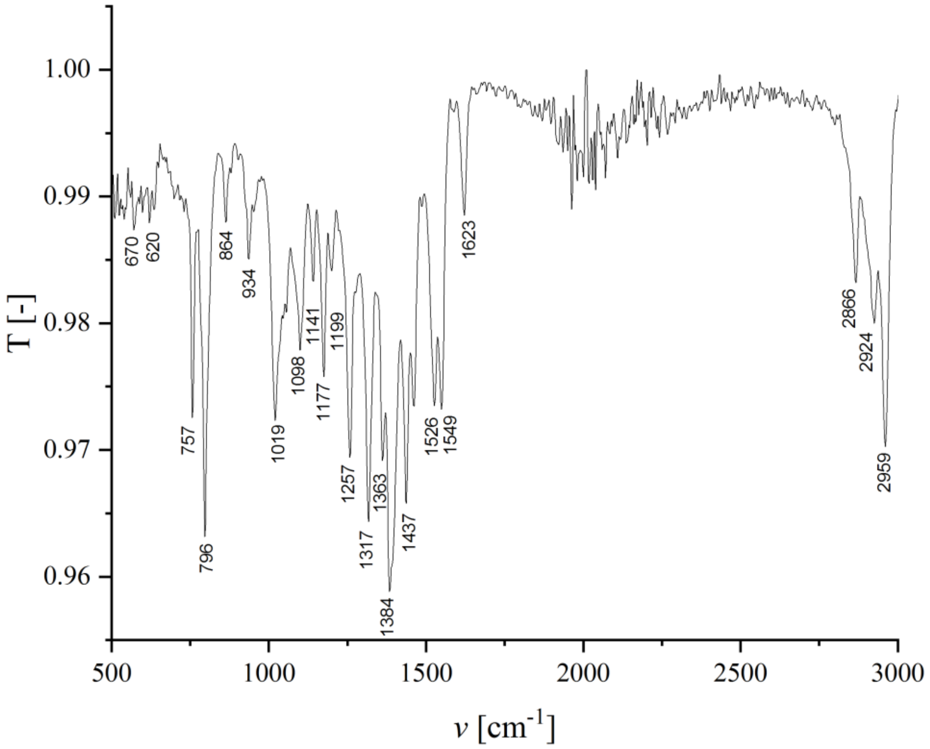

Figure S19. ATR-IR spectrum of compound **5**.

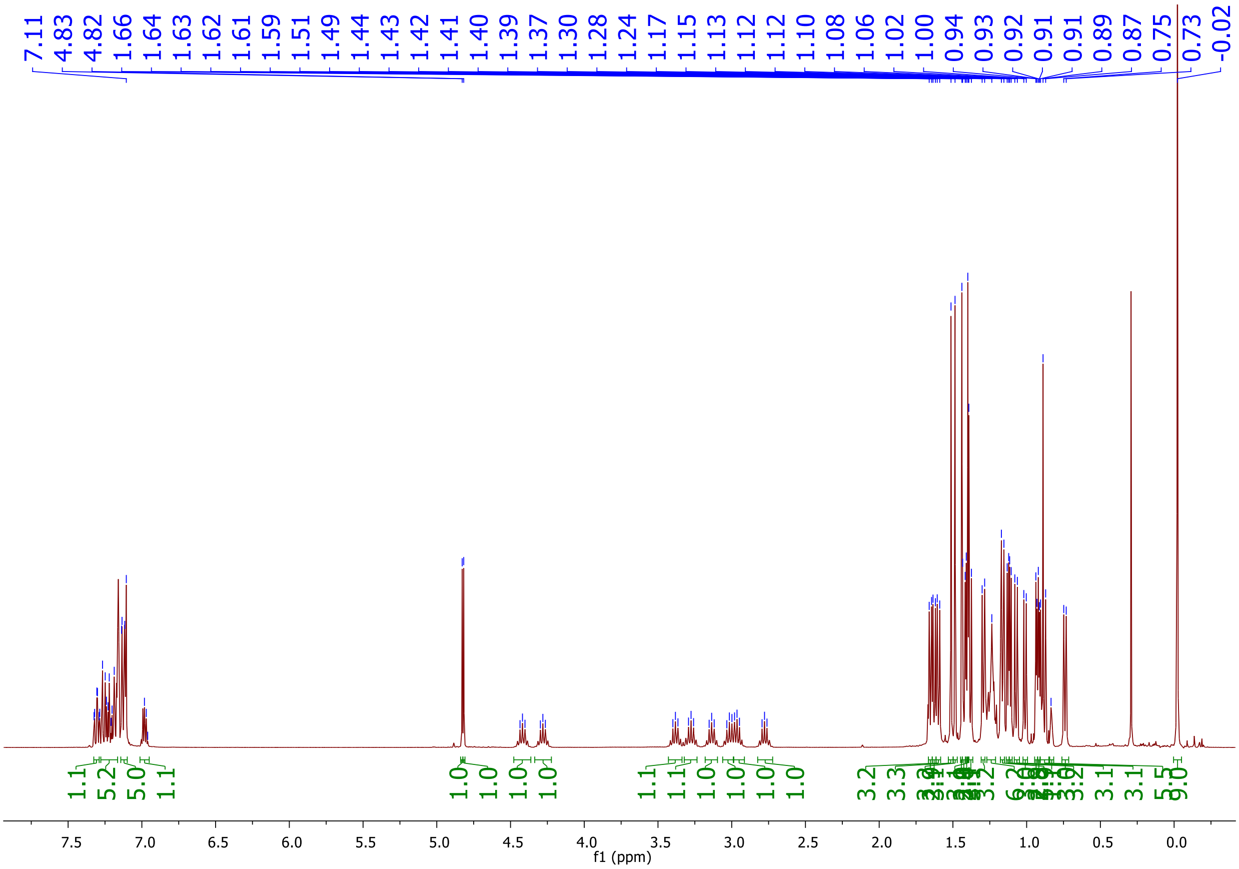

Figure S20. ^{1}H NMR (400 MHz, C_6D_6, 298 K) spectrum of compound **6**.

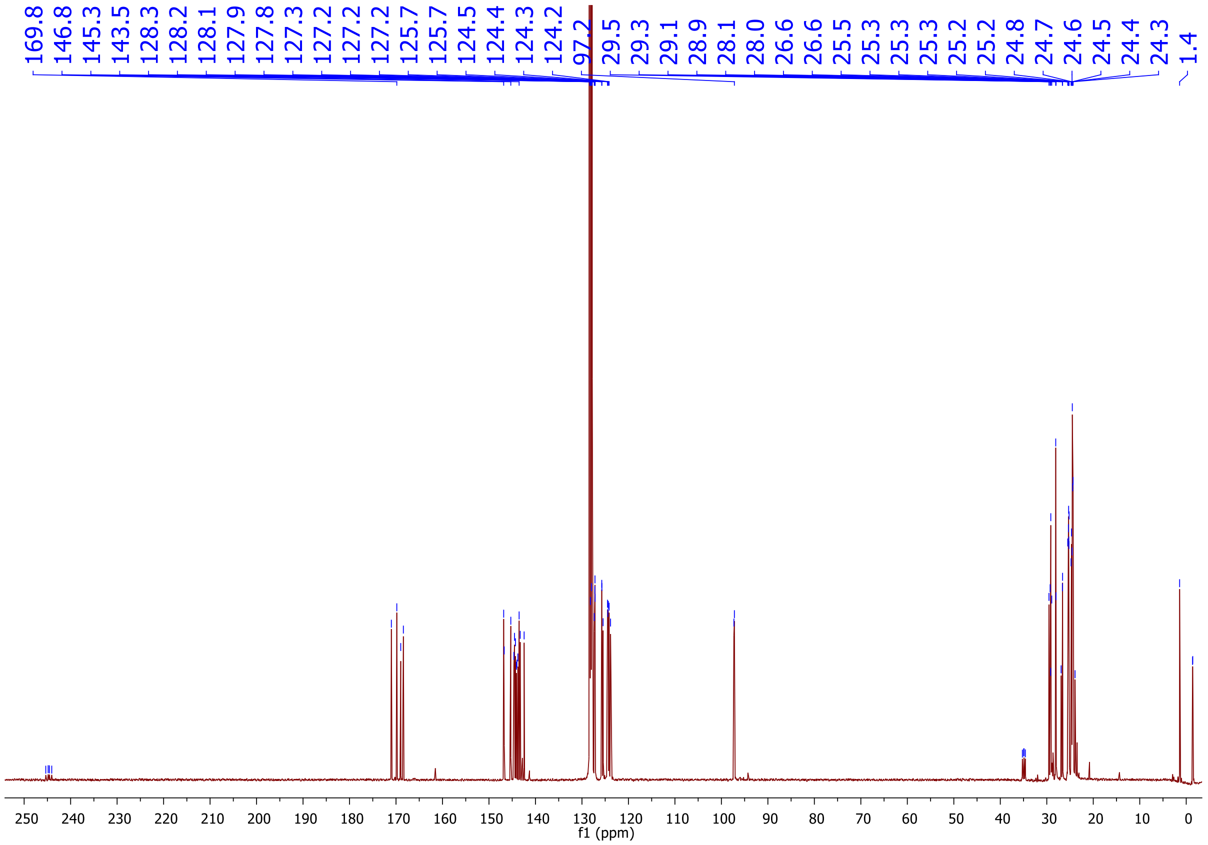

Figure S21. $^{13}C\{^{1}H\}$ NMR (100 MHz, C_6D_6, 298 K) spectrum of compound **6**.

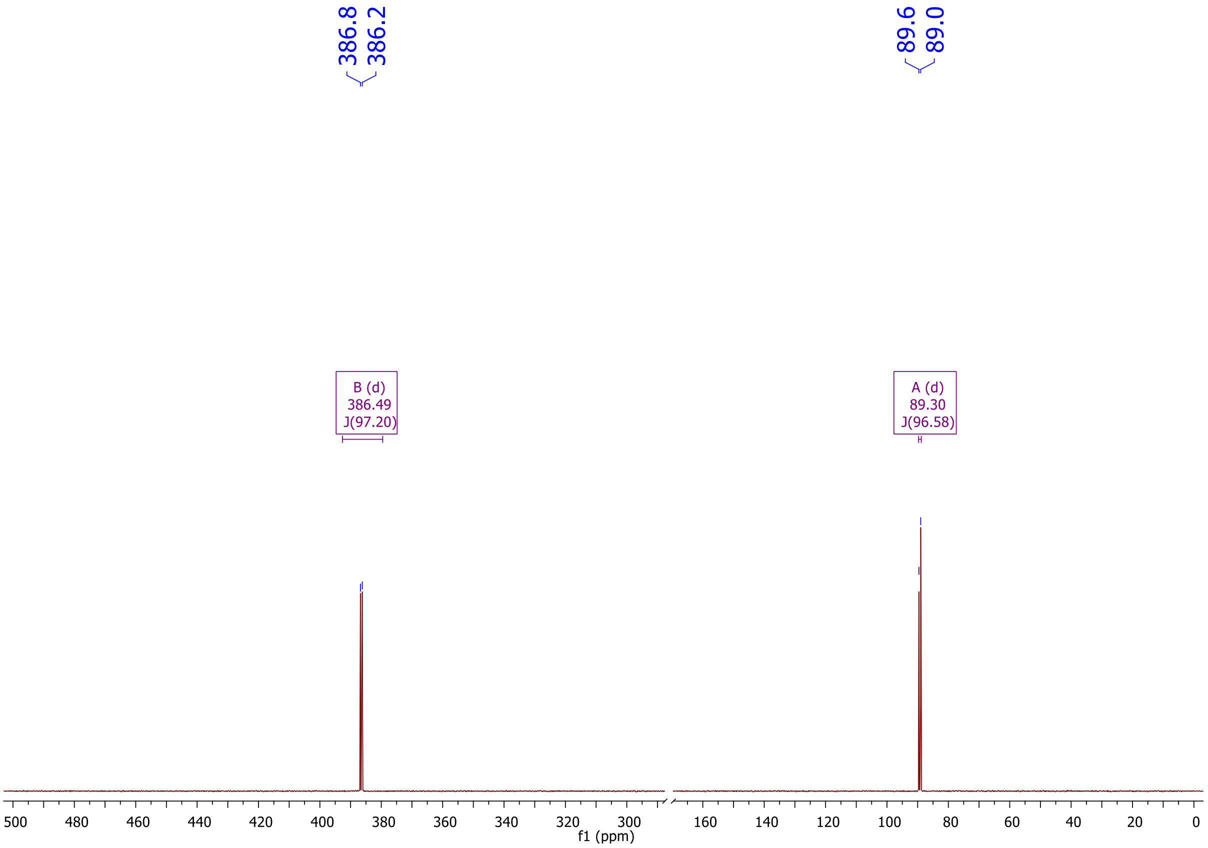

Figure S22. $^{31}P\{^{1}H\}$ NMR (161 MHz, C_6D_6, 298 K) spectrum of compound **6**.

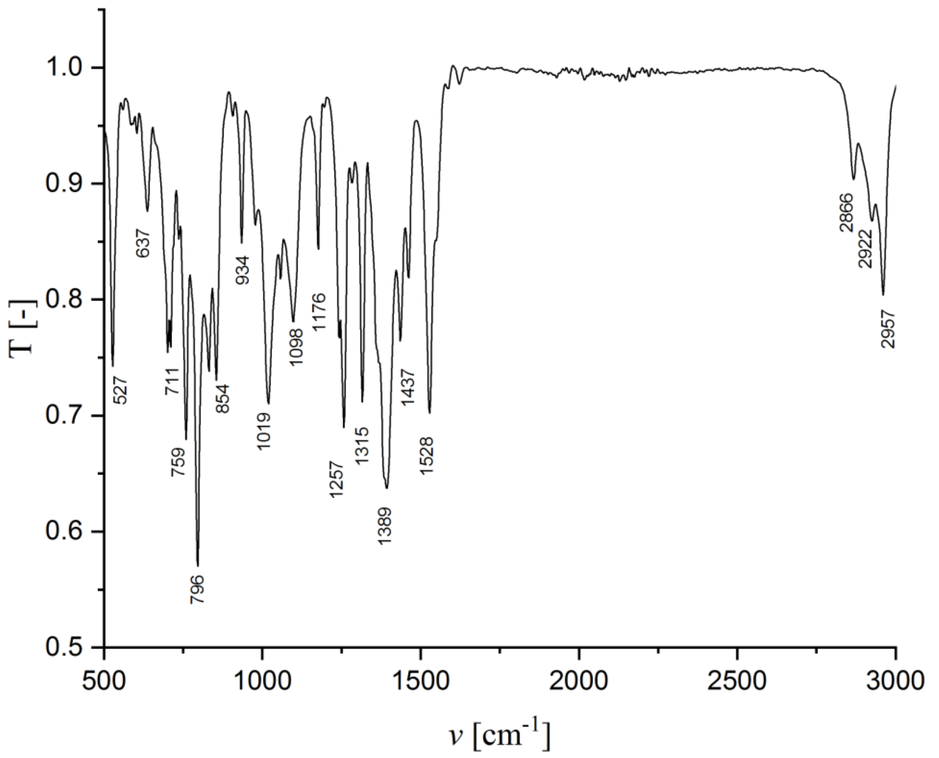

Figure S23. ATR-IR spectrum of compound **6**.

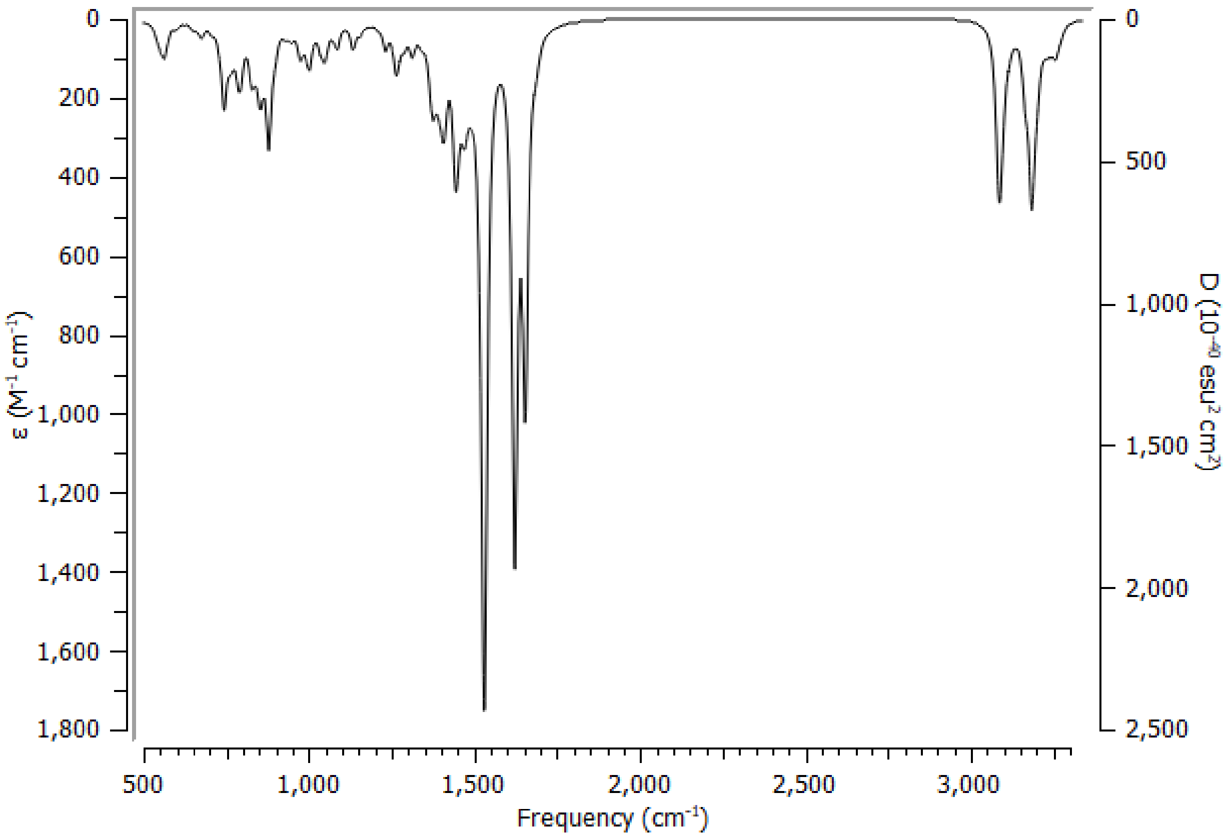

Figure S24. IR spectrum of compound **6** simulated by means of PBE0-D3BJ/def2-SVP.

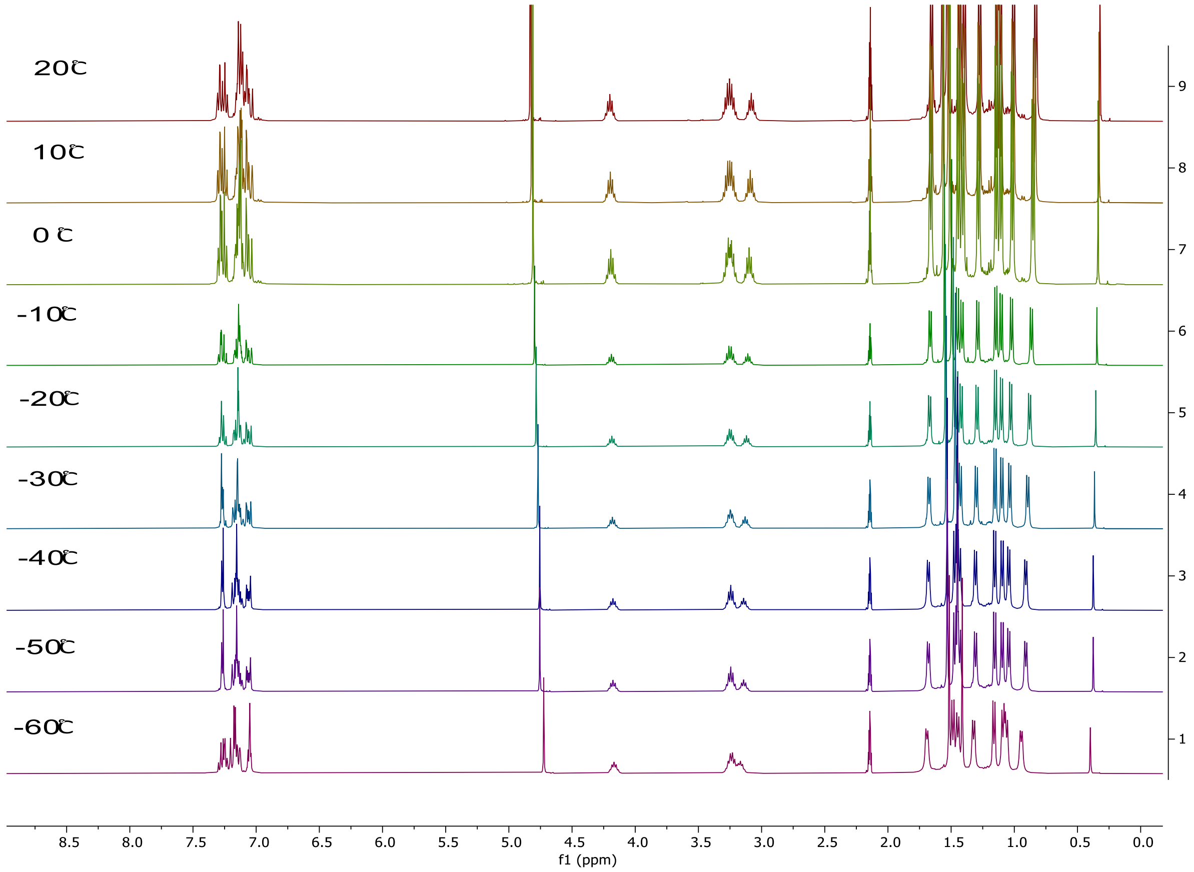

Figure S25. Stacked variable temperature (VT, C_7D_8) ^1H NMR spectra of **2**.

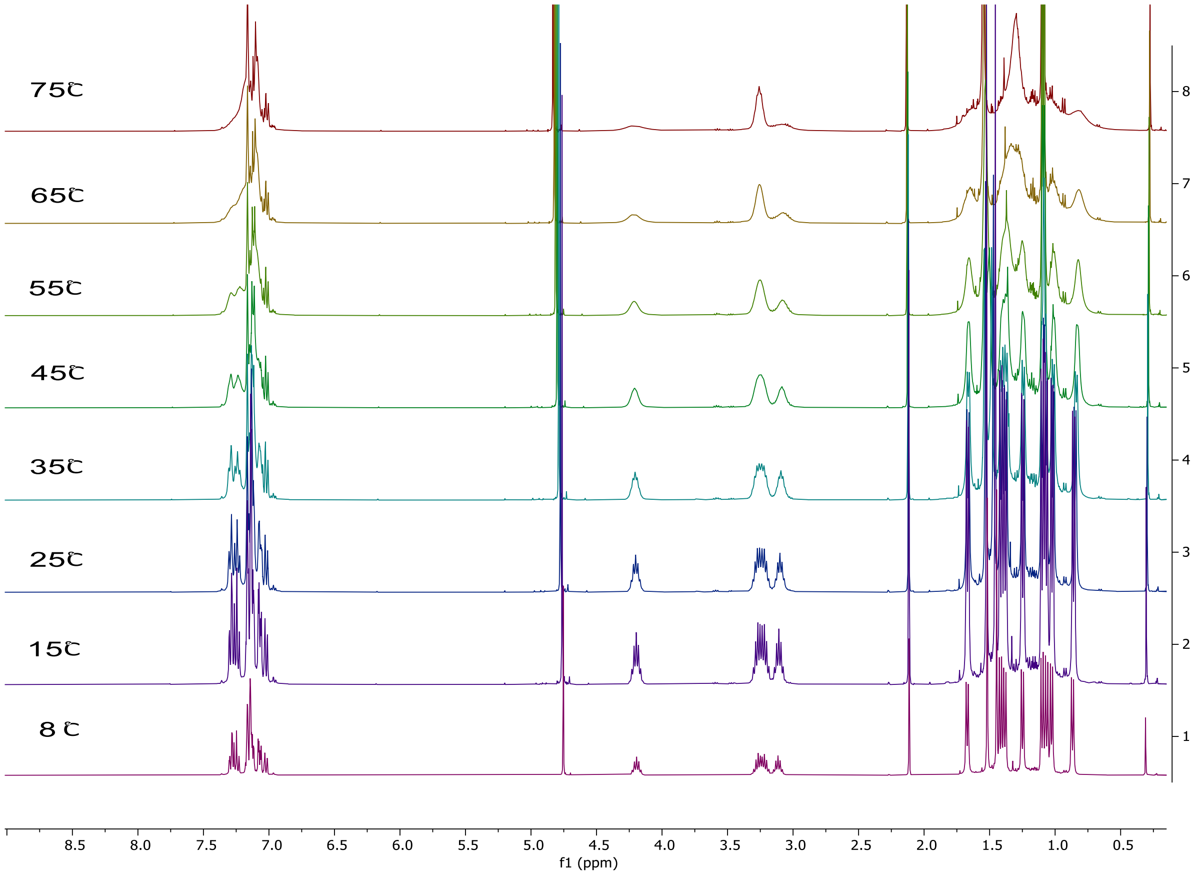

Figure S26. Stacked variable temperature (VT, C_7D_8) ^1H NMR spectra of **2**.

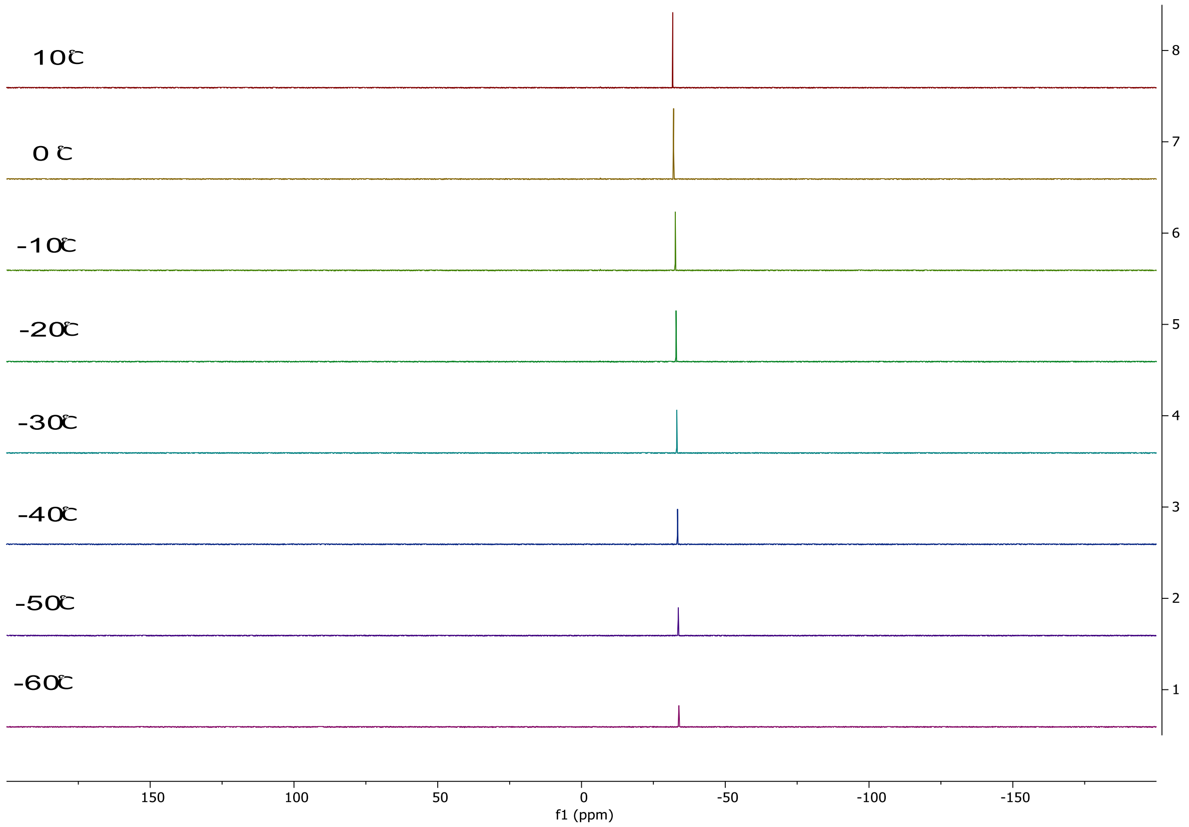

Figure S27. Stacked variable temperature (VT, C_7D_8) ^{31}P NMR spectra of **2**.

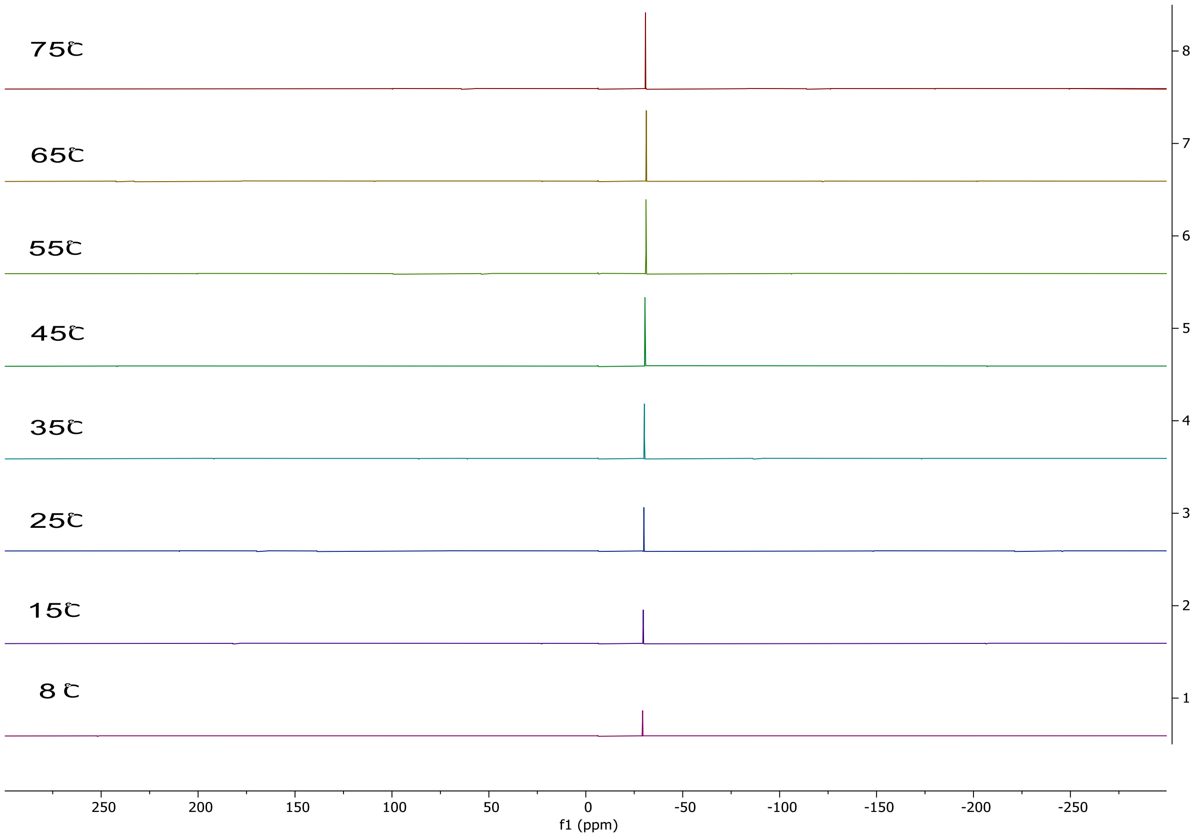

Figure S28. Stacked variable temperature (VT, C_7D_8) ^{31}P NMR spectra of **2**.

3. X-Ray Crystallographic Analysis

The crystals were mounted on nylon loops in inert oil. Data for compound **2–4**, and **6** were collected on a Bruker AXS D8 Venture diffractometer with Photon II detector (mono-chromated Cu$_{K\alpha}$ radiation, λ = 1.54178 Å, micro-focus source) at 100(2) K. The structures were solved by Direct Methods (SHELXS-97)[6] and refined anisotropically by full-matrix least-squares on F^2 (SHELXL-2014).[7-9] Absorption corrections were performed semi-empirically from equivalent reflections on basis of multi-scans (Bruker AXS APEX2). Hydrogen atoms were refined using a riding model or rigid methyl groups. In **2** an isopropyl group is disorerd over two positions. Its corresponding bond lengths and angles were restrained to be equal (SADI) and RIGU restraints applied to its atoms' anisotropic displacement parameters. For C27 and C27' additional SIMU restaints were used. In **3** the central moiety is disorderd over two positions. The corresponding bond lengths and angles of the five-membered ring and the P–C bond lengths were restrained to be equal (SADI). A part of a dipp group is disordered correlated to this. The phenyl ring was restrained to planarity (FLAT) for the larger component and constrained to a regular hexagon (AFIX 66) for the smaller one and the bond lengths and angles of the *i*-propyl group were restrained to be equal (SADI). The solvent molecules are disordered over centres of inversion. The local symmetry was ignored in the refinement (negative PART) and the bond lengths and angles were restrained to be equal (SADI). RIGU and SIMU restraints were applied to the displacement parameters of all disordered atoms. Due to their close proximity P1 and C59' were refined with common displacement parameters (EADP). In **4** the displacement parameters of benzene molecule of residue 2 suggest that the molecule is not exactly placed on the centre of inversion and is slightly disordered. Seperating two alternate positions failed. RIGU restraints were applied to the displacement parameters of the solvent molecules' atoms. The residual electron density shows a second orientation of the PCP-carbene moiety, however its occupancy is too small for a successful refinement. The central moiety of **6** is disordered over two positions. This correlates with the disorder of one of the *i*-propyl groups. Two further *i*-propyl groups are disordered independently of this. In addition, the solvent molecule is highly disordered and could only be modelled crudely by three alternate position. Its bond lengths and angles were restrained to be equal (SADI) and RIGU and SIMU restrained were applied to the displacement parameters.

CCDC-2362185 (mks_018m, **2**), -2362186 (mks_101m, **3**), -2362187 (mks_022m, **4**), and -2362188 (mks_100m, **6**) contain the supplementary crystallographic data for this paper. These data can be obtained free of charge from The Cambridge Crystallographic Data Centre via www.ccdc.cam.ac.uk/data_request/cif.

Table S1. Crystal data and structure refinement of **2** and **3**.

Compound	**2**	**3**
Emp. formula	$C_{59}H_{82}Ga_2N_4OP_2$	$C_{72}H_{108}Ga_2N_6OP_2$
Formula weight	1064.66	1275.02
Temperature [K]	100(2)	100(2)
Crystal system	monoclinic	monoclinic
Space group	$P2_1/c$	$C2/c$
a [Å]	23.5423(10)	26.3977(9)
b [Å]	13.8181(6)	13.4903(5)
c [Å]	17.4303(8)	40.1349(13)
α [°]	90	90
β [°]	96.3710(11)	91.7723(15)
γ [°]	90	90
V [Å^3]	5635.2(4)	14285.7(9)
Z	4	8
ρ [Mgm^{-3}]	1.255	1.186
μ [mm^{-1}]	2.023	1.681
F (000)	2256	5456
Crystal size [mm]	0.221 × 0.221 × 0.192	0.367 × 0.102 × 0.100
θ max [°]	80.782	79.834
Index ranges	$-29 \leq h \leq 25$;<br>$-17 \leq k \leq 17$<br>$-22 \leq l \leq 22$	$-33 \leq h \leq 33$<br>$-17 \leq k \leq 17$<br>$-50 \leq l \leq 51$
No. of reflect. collected	210810	304149
Unique reflect.	12297	15479
R_{int}	0.0254	0.0765
Data / restraints / params.	12297 / 31 / 663	13371/ 1033 / 1066
Goodness-of-fit on F^2	1.052	1.079
$R1$ [I>2$\sigma(I)$]	0.0238	0.0479
$wR2$ [I>2$\sigma(I)$]	0.0627	0.1128
$R1$ [all data]	0.0239	0.0568
$wR2$ [all data]	0.0628	0.1188
$\Delta\rho_{final}$ (max/min) [e·Å^{-3}]	0.379/-0.411	0.422/-0.659

Table S2. Crystal data and structure refinement of **4** and **6**.

Compound	**4**	**6**
Emp. formula	$C_{79}H_{111}Ga_2N_6OP_2$	$C_{69}H_{106}Ga_2N_4OP_2Si$
Formula weight	1362.11	1237.04
Temperature [K]	100(2)	100(2)
Crystal system	monoclinic	monoclinic
Space group	*C* 2/*c*	$P\ 2_1/c$
a [Å]	20.7310(4)	15.1894(6)
b [Å]	18.2400(4)	13.1269(5)
c [Å]	39.5206(7)	34.6139(13)
α [°]	90	90
β [°]	95.9543(13)	96.719(2)
γ [°]	90	90
V [Å^3]	14863.4(5)	6854.3(5)
Z	8	4
ρ [Mgm^{-3}]	1.217	1.199
μ [mm^{-1}]	1.650	1.890
F (000)	5816	2648
Crystal size [mm]	0.319 × 0.203 × 0.074	0.242 × 0.168 × 0.049
θ max [°]	81.015	80.847
Index ranges	$-26 \leq h \leq 26$; $-23 \leq k \leq 23$ $-50 \leq l \leq 50$	$-19 \leq h \leq 19$ $-16 \leq k \leq 16$ $-44 \leq l \leq 44$
No. of reflect. collected	473924	297275
Unique reflect.	16343	14958
R_{int}	0.0292	0.0783
Data / restraints / params.	16343 / 45 / 838	13022 / 343 / 1017
Goodness-of-fit on F^2	1.109	1.187
$R1$ [$I>2\sigma(I)$]	0.0351	0.0580
$wR2$ [$I>2\sigma(I)$]	0.0899	0.1255
$R1$ [all data]	0.0352	0.0674
$wR2$ [all data]	0.0899	0.1307
$\Delta\rho_{final}$ (max/min) [e·Å^{-3}]	1.771/-0.646	0.604/-0.462

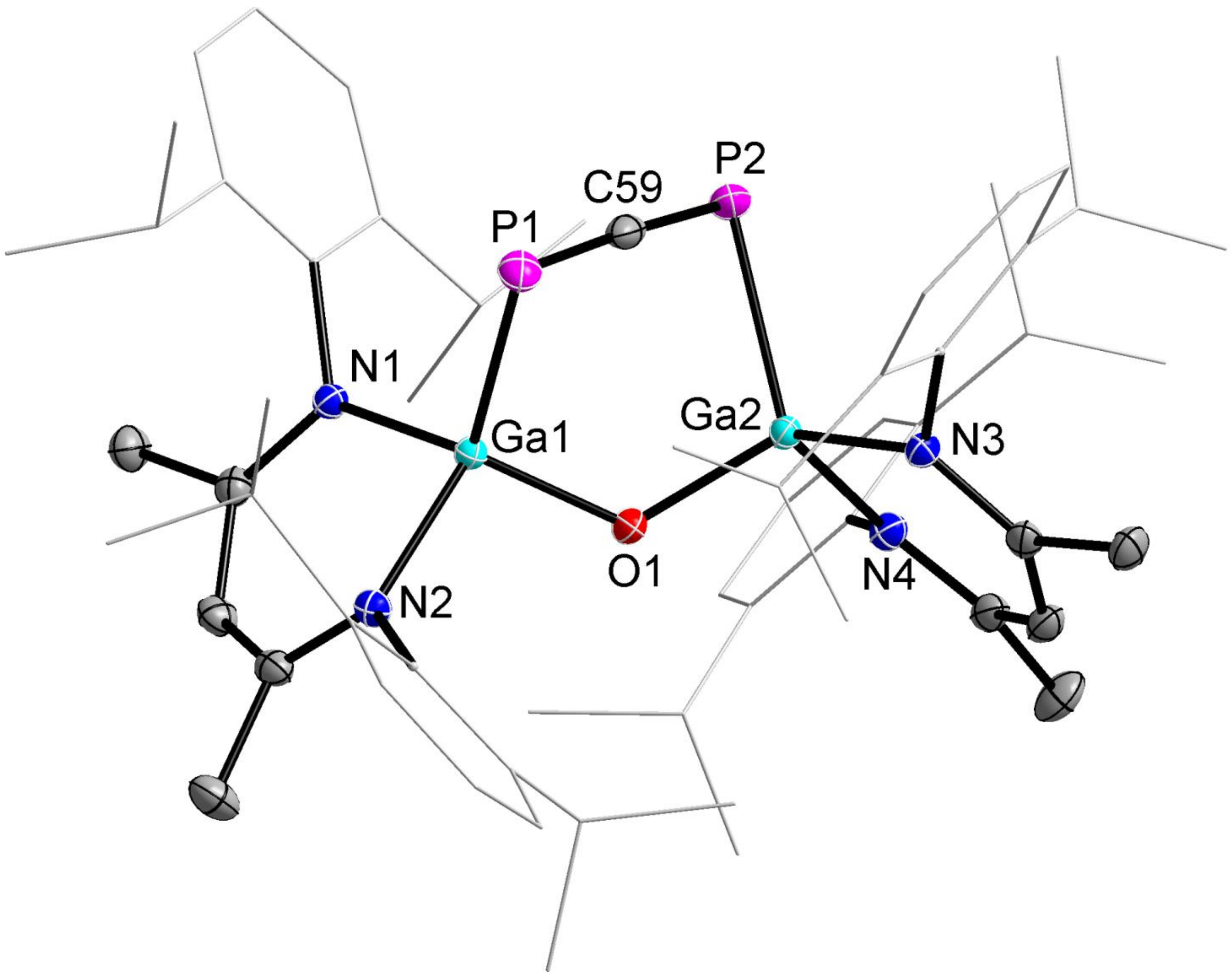

Figure S29. Molecular structure of compound **2** with thermal ellipsoids at 50% probability level. The hydrogen atoms and alternate positions of the disordered parts are omitted for clarity. Selected bond length (Å) and angels (°): Ga(1)–O(1) 1.8082(8), Ga(1)–N(2) 1.9629(9), Ga(1)–N(1) 1.9745(9), Ga(1)–P(1) 2.3767(3), Ga(2)–O(1) 1.8046(8), Ga(2)–N(3) 1.9728(10), Ga(2)–N(4) 1.9773(10), Ga(2)–P(2) 2.3845(3), P(1)–C(59) 1.6516(13), P(2)–C(59) 1.6506(13); N(2)–Ga(1)–N(1) 95.48(4), O(1)–Ga(1)–P(1) 107.78(3), N(2)–Ga(1)–P(1) 118.03(3), N(1)–Ga(1)–P(1) 110.91(3), O(1)–Ga(2)–N(3) 121.18(4), O(1)–Ga(2)–N(4) 102.86(4), N(3)–Ga(2)–N(4) 95.45(4), O(1)–Ga(2)–P(2) 107.62(3), N(3)–Ga(2)–P(2) 110.97(3), N(4)–Ga(2)–P(2) 118.83(3), C(59)–P(1)–Ga(1) 81.63(4), C(59)–P(2)–Ga(2) 81.39(4), Ga(2)–O(1)–Ga(1) 123.63(4).

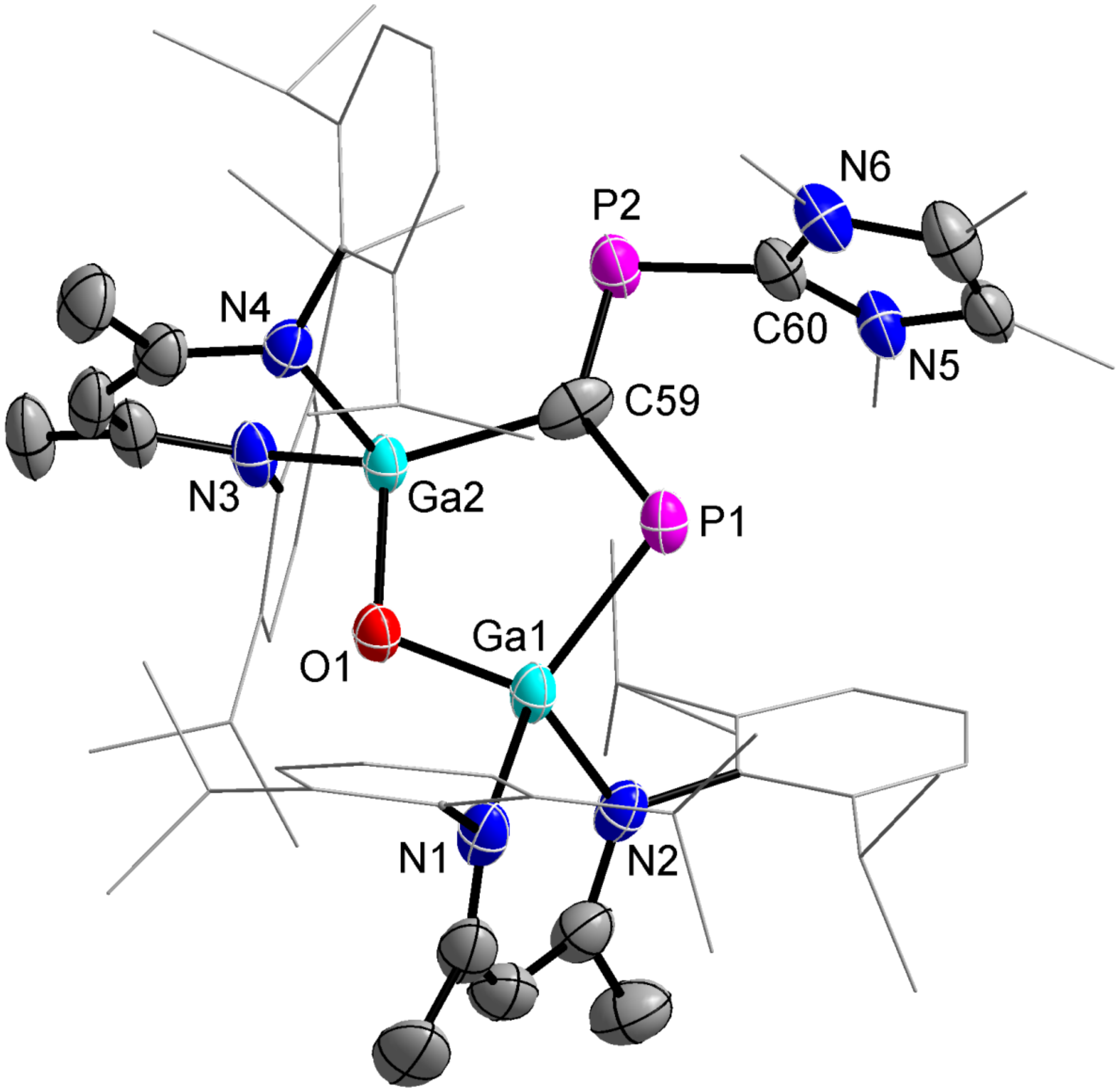

Figure S30. Molecular structure of compound **3** with thermal ellipsoids at 50% probability level. The hydrogen atoms, alternate positions of the disordered parts, and disordered solvent molecules (*n*-hexane) are omitted for clarity. are omitted for clarity. Selected bond length (Å) and angels (°): Ga(1)–O(1) 1.8239(16), Ga(1)–N(1) 2.006(2), Ga(1)–N(2) 2.007(2), Ga(1)–P(1) 2.2926(11), Ga(2)–O(1) 1.8127(17), Ga(2)–N(4) 1.998(2), Ga(2)–N(3) 2.021(2), Ga(2)–C(59) 2.092(8), P(1)–C(59) 1.801(6), P(2)–C(59) 1.623(7), P(2)–C(60) 1.847(3), N(5)–C(60) 1.342(4), N(6)–C(60) 1.335(4); N(1)–Ga(1)–N(2) 92.92(9), O(1)–Ga(1)–P(1) 107.50(6), N(4)–Ga(2)–N(3) 93.06(9), O(1)–Ga(2)–C(59) 108.38(19), Ga(2)–O(1)–Ga(1) 110.01(8), P(2)–C(59)–P(1) 128.9(5), P(2)–C(59)–Ga(2) 119.9(3), P(1)–C(59)–Ga(2) 109.3(4), C(59)–P(2)–C(60) 97.5(3).

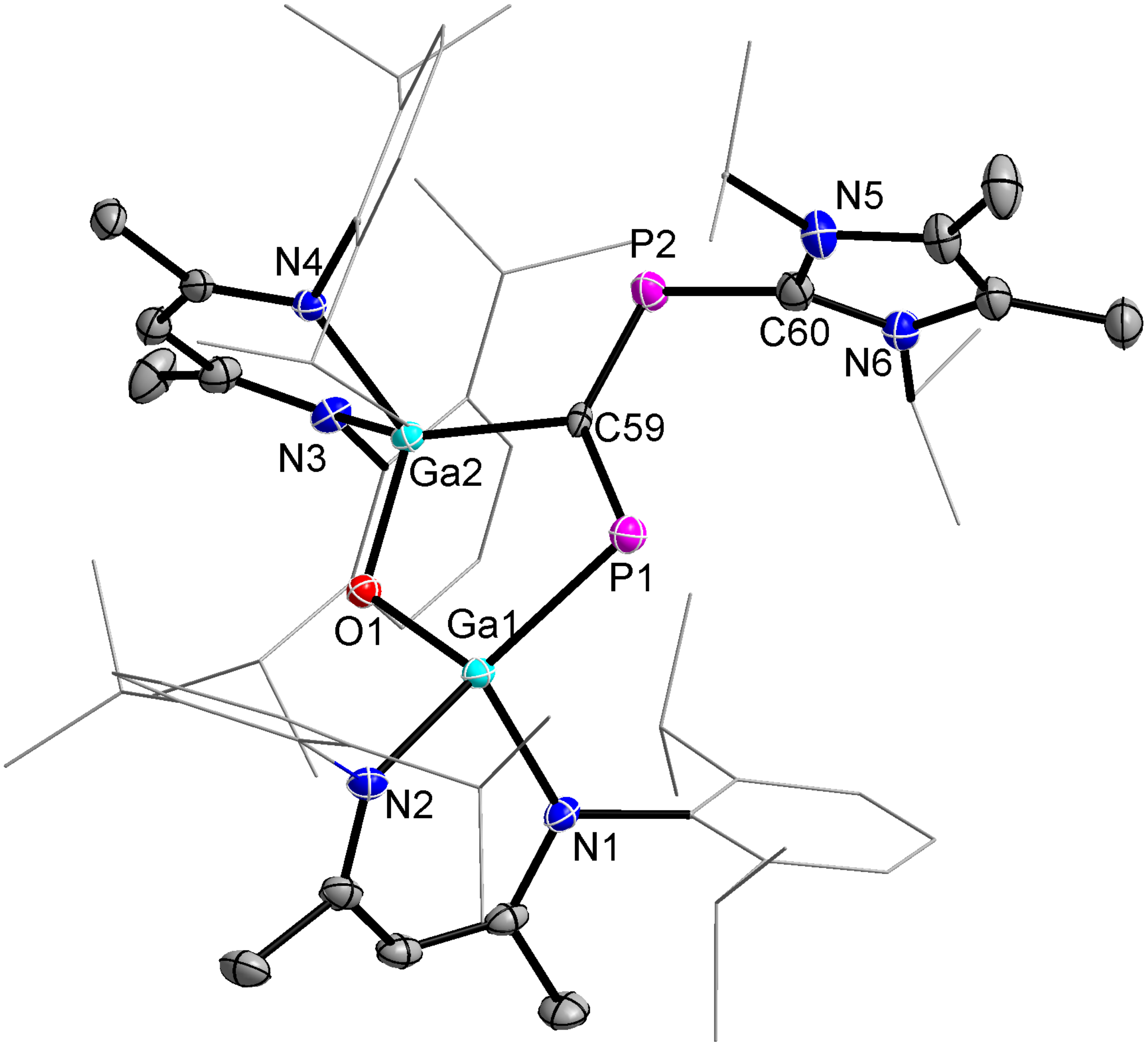

Figure S31. Molecular structure of compound **4** with thermal ellipsoids at 50% probability level. The hydrogen atoms and solvent molecules (benzene) are omitted for clarity. Selected bond length (Å) and angels (°): Ga(1)–O(1) 1.8241(11), Ga(1)–N(1) 2.0160(13), Ga(1)–N(2) 2.0319(14), Ga(1)–P(1) 2.3351(4), Ga(2)–O(1) 1.8140(11), Ga(2)–N(4) 2.0033(13), Ga(2)–N(3) 2.0249(13), Ga(2)–C(59) 2.0285(14), P(1)–C(59) 1.7716(15), P(2)–C(59) 1.6990(15), P(2)–C(60) 1.8571(17), N(5)–C(60) 1.354(2), N(6)–C(60) 1.354(2); N(1)–Ga(1)–N(2) 91.01(6), O(1)–Ga(1)–P(1) 106.85(4), O(1)–Ga(2)–C(59) 107.17(5), C(59)–P(1)–Ga(1) 96.56(5), C(59)–P(2)–C(60) 105.29(7), Ga(2)–O(1)–Ga(1) 111.21(6), P(2)–C(59)–P(1) 129.91(8), P(2)–C(59)–Ga(2) 114.91(8), P(1)–C(59)–Ga(2) 114.92(8).

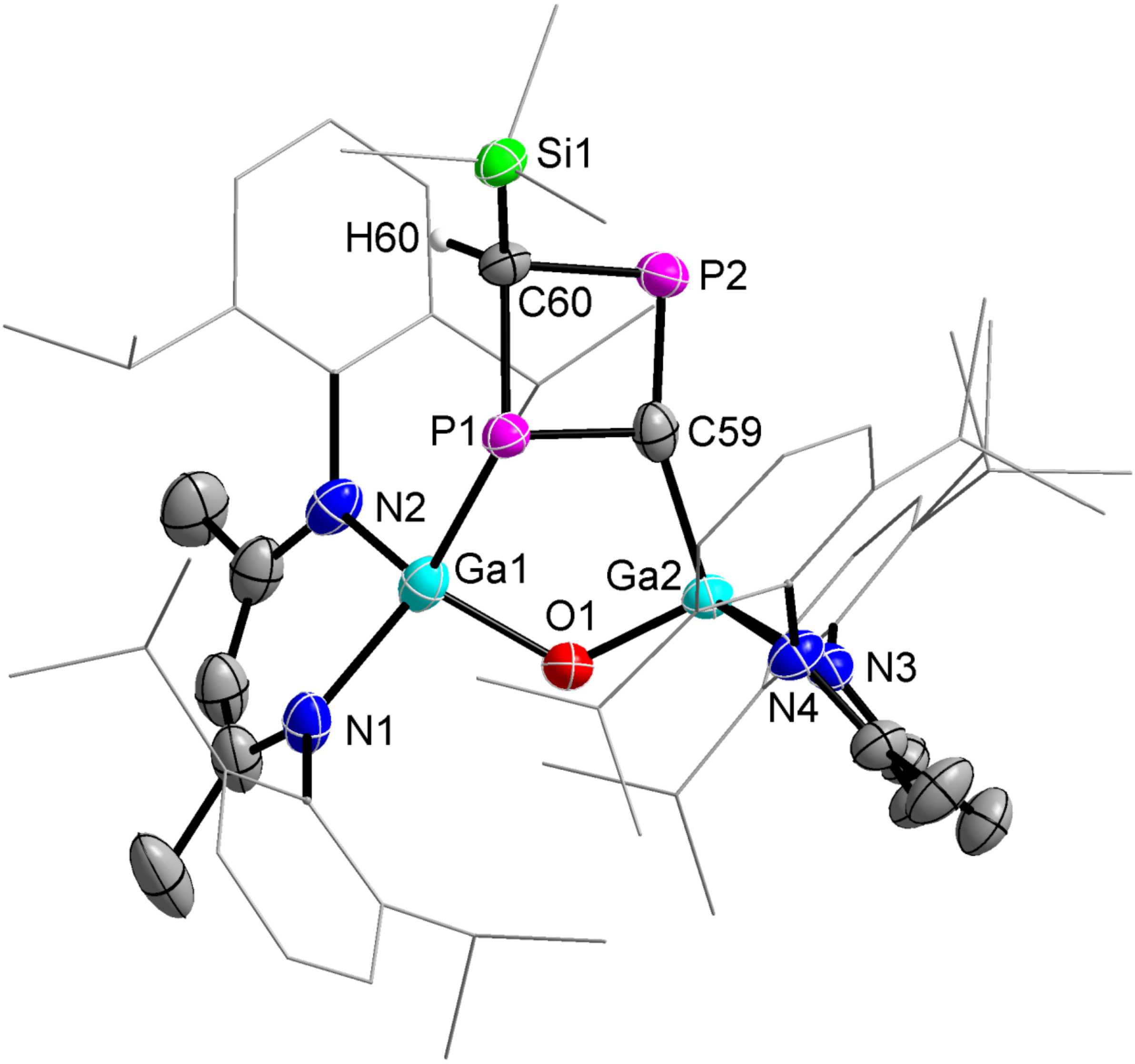

Figure S32. Molecular structure of compound **6** with thermal ellipsoids at 50% probability level. The hydrogen atoms, alternate positions of the disordered parts, and solvent molecules (*n*-hexane) are omitted for clarity. Selected bond length (Å) and angels (°): Ga(1)–O(1) 1.820(2), Ga(1)–N(1) 1.980(3), Ga(1)–N(2) 1.982(3), Ga(1)–P(1) 2.503(2), Ga(2)–O(1) 1.815(2), Ga(2)–C(59) 1.940(10), Ga(2)–N(3) 1.977(2), Ga(2)–N(4) 1.989(3) P(1)–C(59) 1.827(9), P(1)–C(60) 1.888(6), P(2)–C(59) 1.720(10), P(2)–C(60) 1.875(6), Si(1)–C(60) 1.874(6); N(1)–Ga(1)–N(2) 94.20(12), O(1)–Ga(1)–P(1) 95.28(8) O(1)–Ga(2)–C(59) 102.7(3), N(3)–Ga(2)–N(4) 94.14(10) C(59)–P(1)–C(60) 84.9(4), C(59)–P(1)–Ga(1) 91.6(3), C(60)–P(1)–Ga(1) 121.62(19), C(59)–P(2)–C(60) 88.3(3), P(2)–C(59)–P(1) 96.9(5), P(2)–C(59)–Ga(2) 150.8(6), P(1)–C(59)–Ga(2) 110.4(5), P(2)–C(60)–P(1) 89.8(2), Ga(2)–O(1)–Ga(1) 118.55(12).

4. UV-vis Spectroscopy

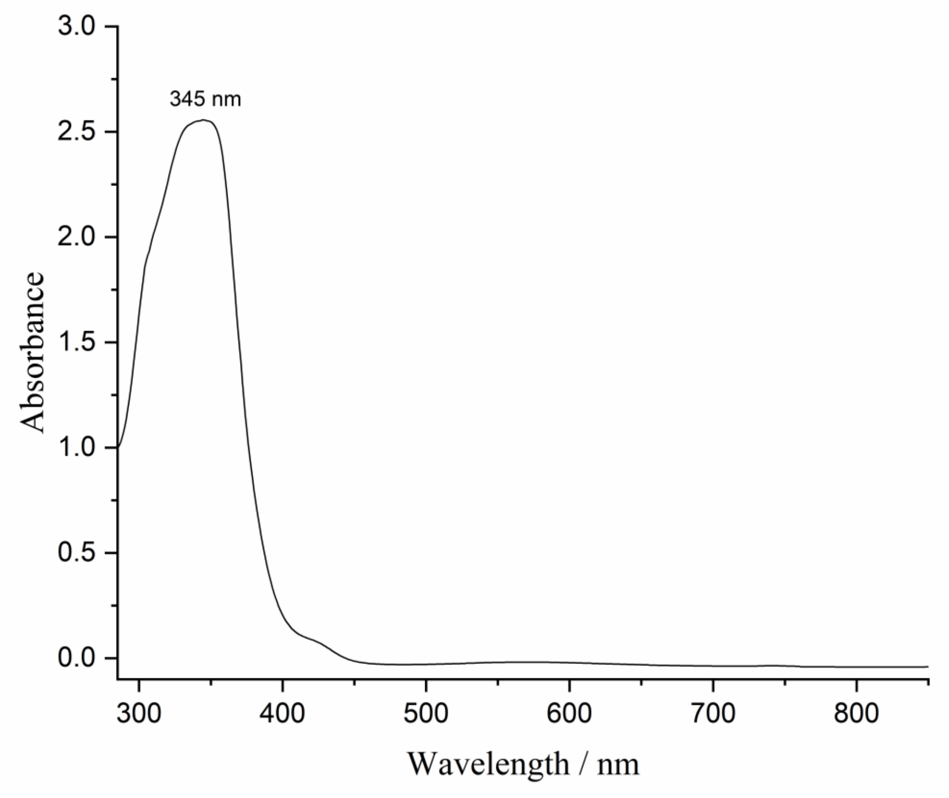

Figure S33. UV-visible spectrum of **2** (10^{-4} M) recorded in THF.

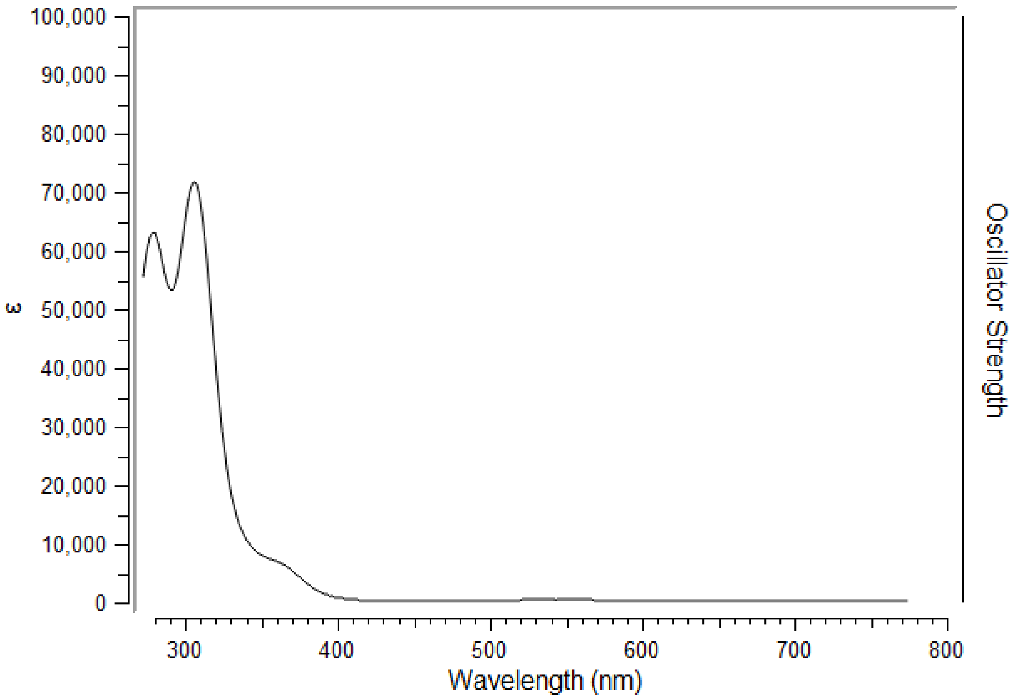

Figure S34. UV-visible spectrum of **2** simulated at TD-PBE0-D3BJ(SMD,THF)/def2-SVP level of theory.

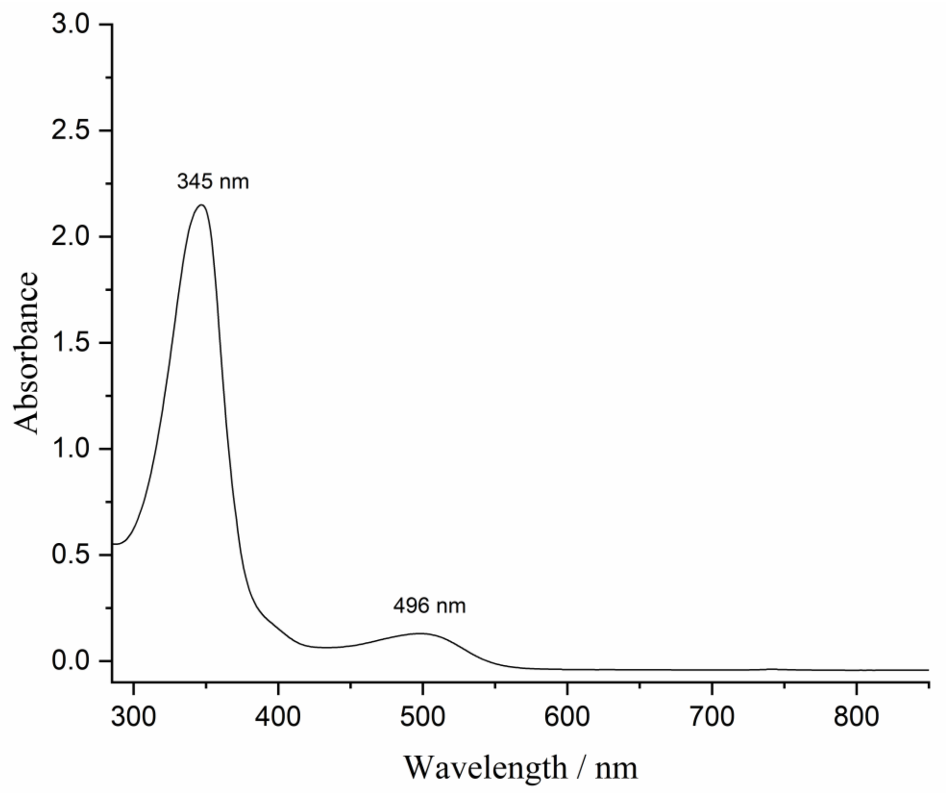

Figure S35. UV-visible spectrum of **3** (10^{-4} M) recorded in THF.

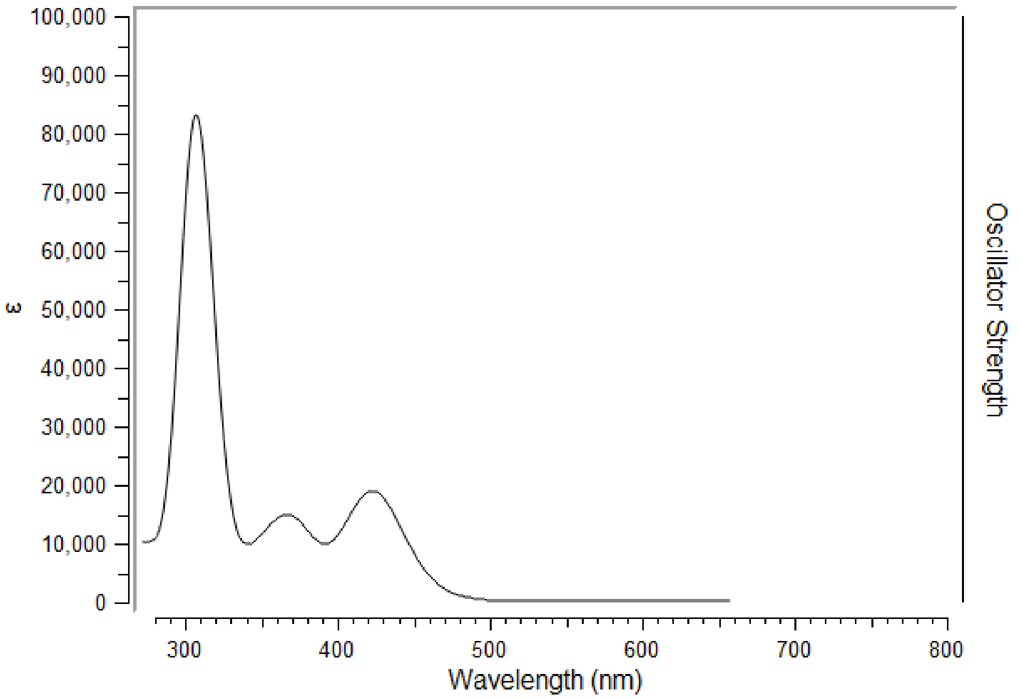

Figure S36. UV-visible spectrum of **3** simulated at TD-PBE0-D3BJ(SMD,THF)/def2-SVP level of theory.

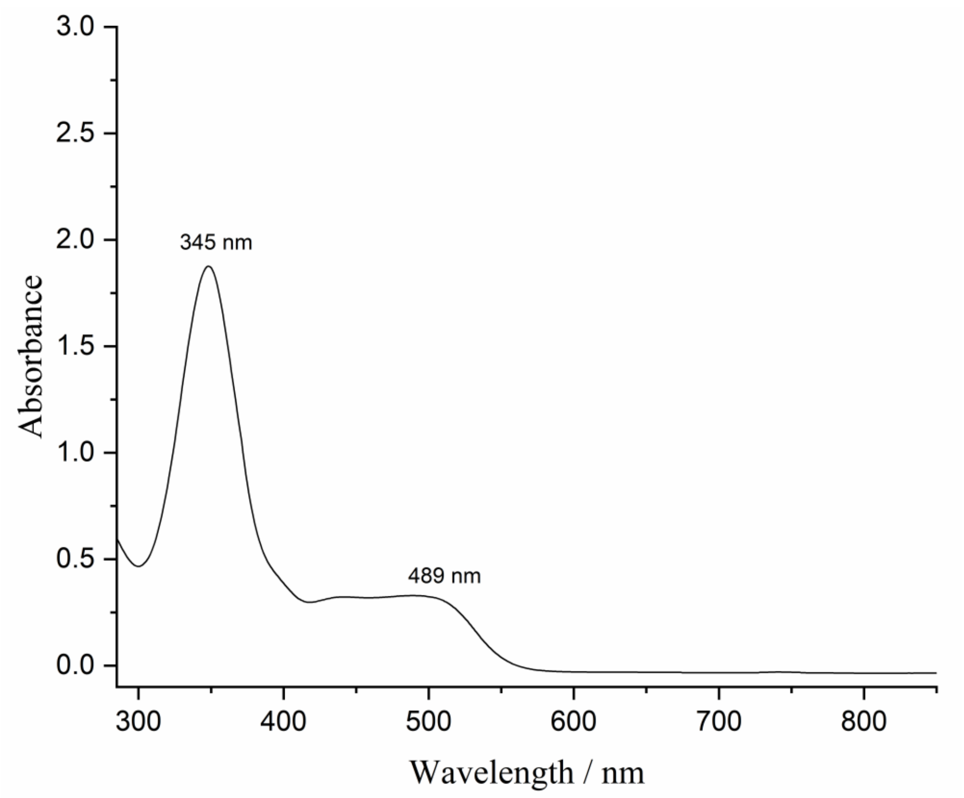

Figure S37. UV-visible spectrum of **4** (10^{-4} M) recorded in THF.

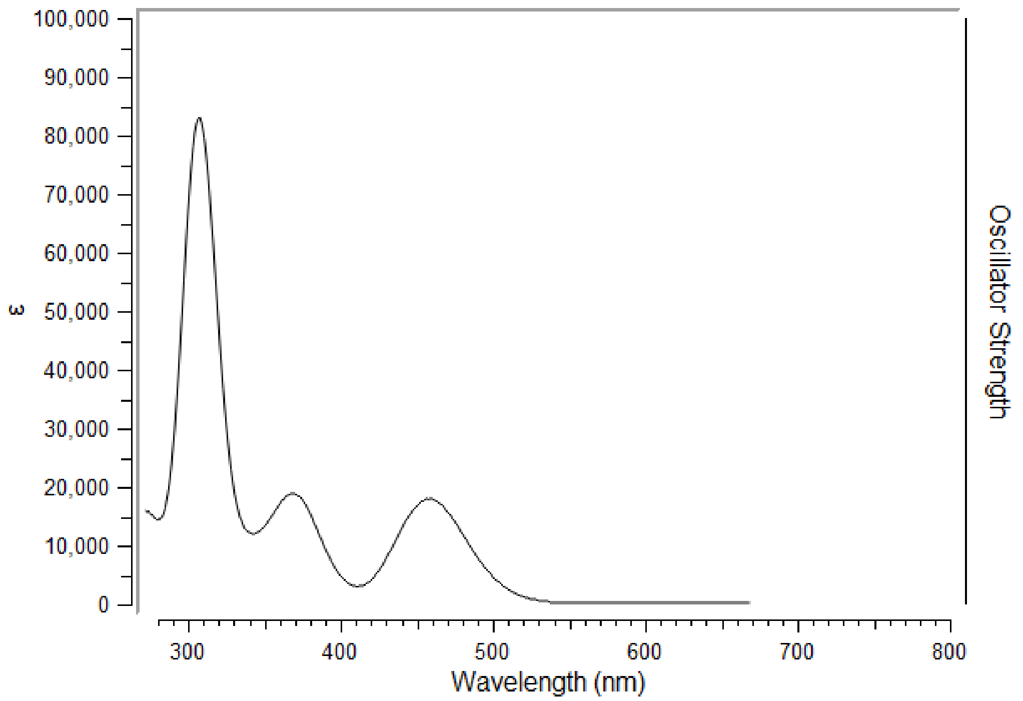

Figure S38. UV-visible spectrum of **4** simulated at TD-PBE0-D3BJ(SMD,THF)/def2-SVP level of theory.

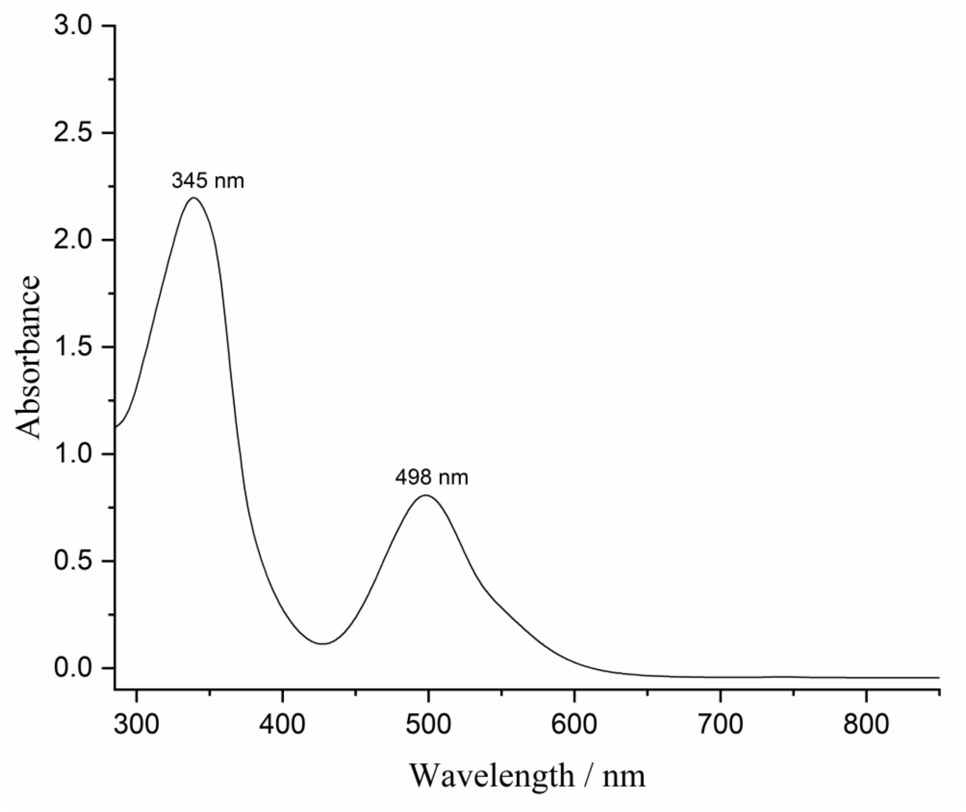

Figure S39. UV-visible spectrum of **5** (10^{-4} M) recorded in THF.

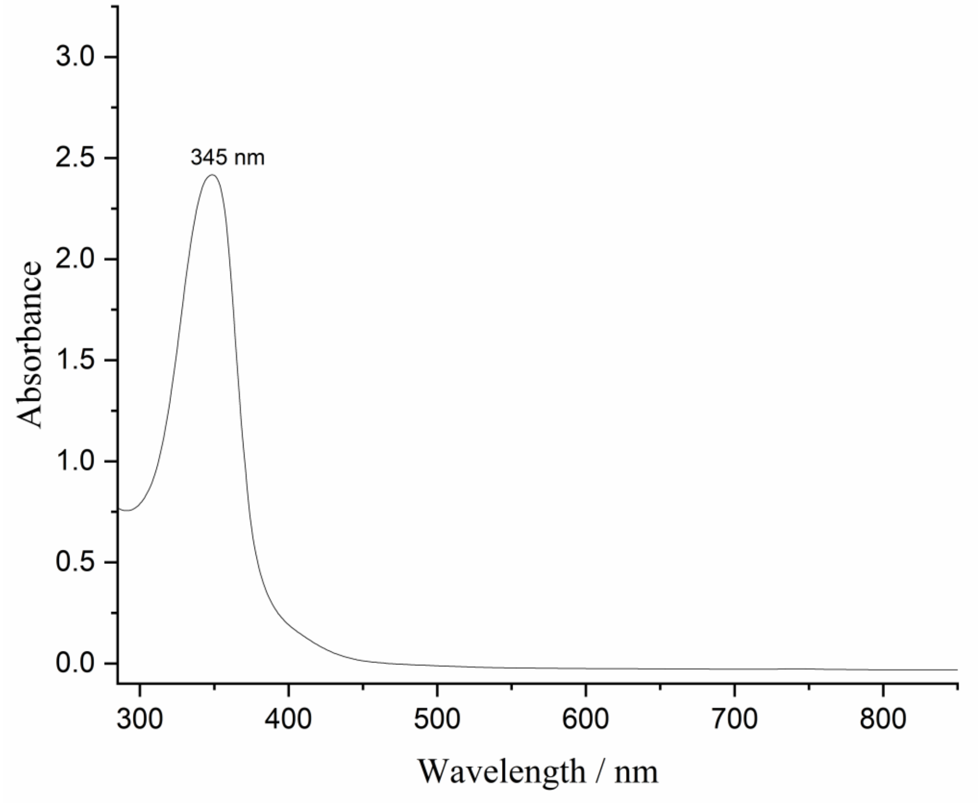

Figure S40. UV-visible spectrum of **6** (10^{-4} M) recorded in THF.

5. Computational Details

All calculations were performed by using the program package Gaussian 16[10]. The geometrical parameters of all stationary points were optimized by means of PBE[11] and PBE0[12] with the dispersion correction D3BJ[13]. As basis set def2-SVP[14] was employed. For all stationary points, no symmetry restriction was applied. Frequency calculations were carried out at each of the structures to verify the nature of the stationary point. It turned out that all transition states have exactly one imaginary frequency, whereas all other structures have none. Furthermore, the energies of the stationary points were calculated using the density functionals PBE0-D3BJ and PBE0-D3BJ with the basis set def2-TZVP[14]. To take solvent effects into account, the solvent model SMD[15] (THF and toluene as solvents) was used for the single-point calculations.

To simulate UV-visible spectra, the first 50 transitions were calculated using TD-PBE0-D3BJ(SMD,THF)/def2-SVP. The peak half-width at half-height was set to 0.15 eV to display the spectra.

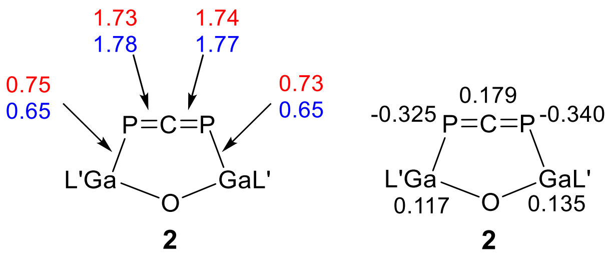

Figure S41. Mayer (red), Wiberg (blue) bond orders and NBO-charges (black) of compound **2** calculated by means of PBE0-D3BJ(SMD,toluene as solvent)/def2-TZVP//PBE0-D3BJ/def2-SVP. L' = HC[C(Me)NDipp]$_2$, Dipp = 2,6-^{i}Pr$_2$C$_6$H$_3$.

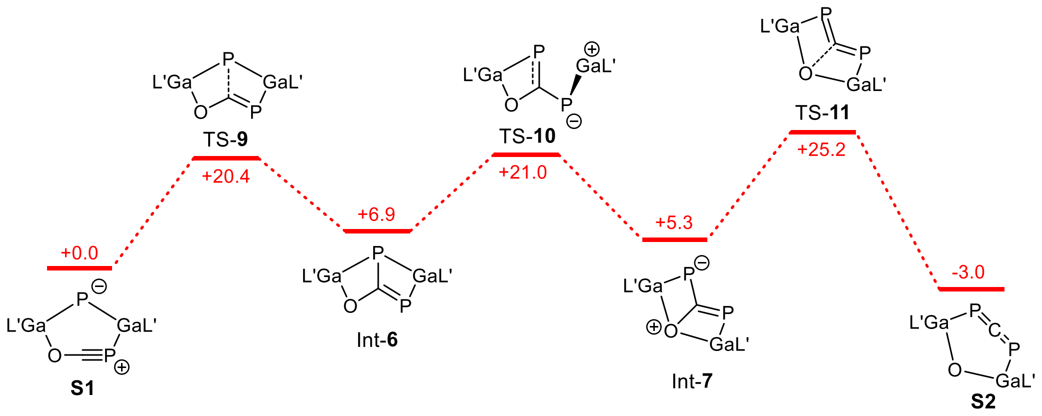

Figure S42. Gibbs energies (G) for the rearrangement from **S1** to **S2** calculated by means of PBE0-D3BJ(SMD,THF as solvent)/def2-TZVP//PBE0-D3BJ/def2-SVP. L' = HC[C(Me)NMe]$_2$. The values are given in kcal/mol.

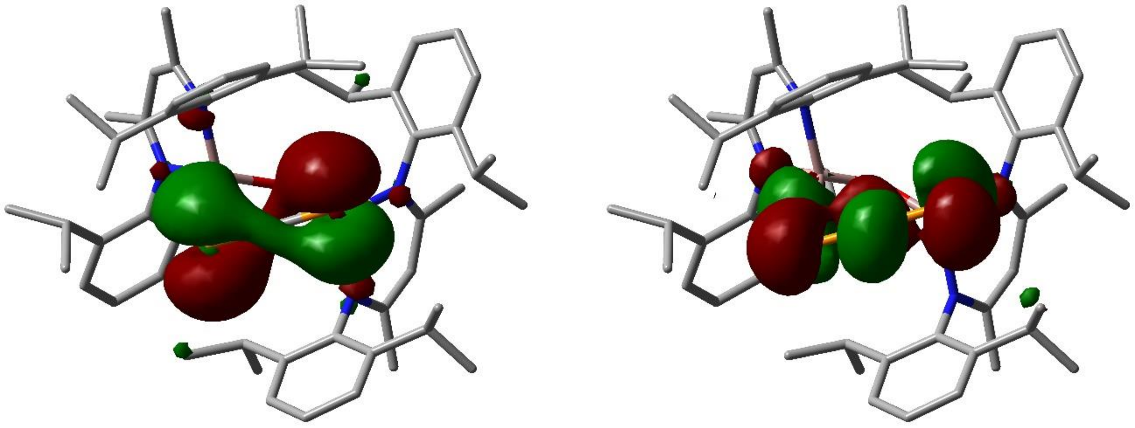

Figure S43. Occupied (left) and virtual (right) natural transition orbitals (isovalue = 0.030 au) for the transition λ_1 of **2** calculated by means of TD-PBE0-D3BJ(SMD,THF)/def2-SVP level of theory (546.71 nm, f = 0.0042). Hydrogen atoms are omitted for clarity.

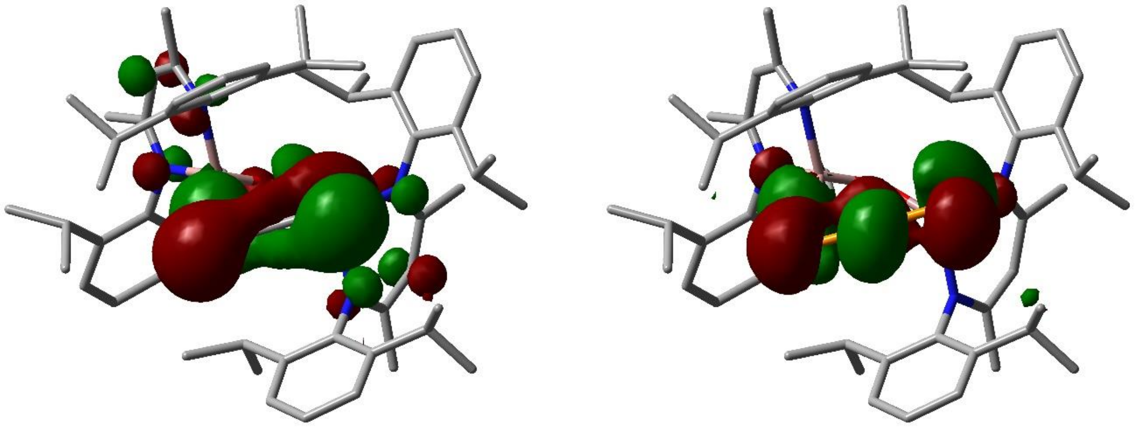

Figure S44. Occupied (left) and virtual (right) natural transition orbitals (isovalue = 0.030 au) for the transition λ_2 of **2** calculated by means of TD-PBE0-D3BJ(SMD,THF)/def2-SVP level of theory (401.27 nm, f = 0.0048). Hydrogen atoms are omitted for clarity.

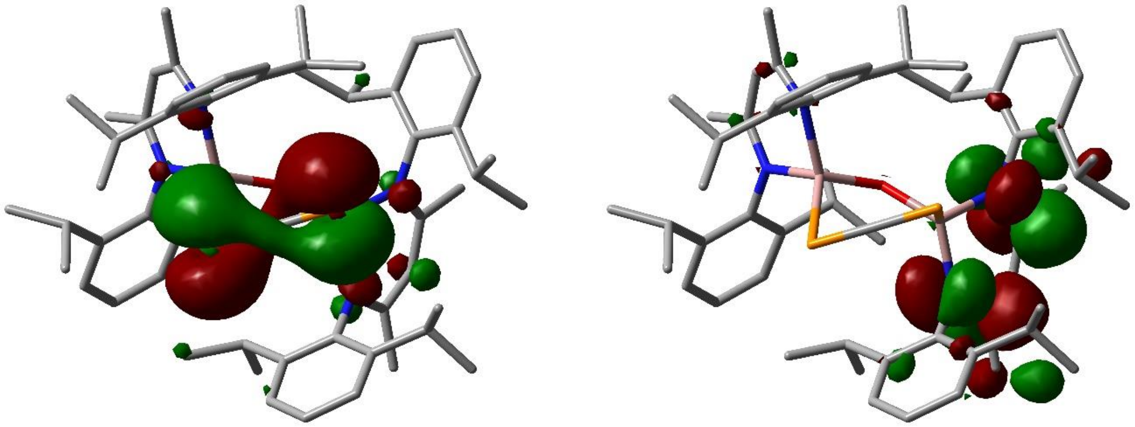

Figure S45. Occupied (left) and virtual (right) natural transition orbitals (isovalue = 0.030 au) for the transition λ_3 of **2** calculated by means of TD-PBE0-D3BJ(SMD,THF)/def2-SVP level of theory (369.71 nm, f = 0.0277). Hydrogen atoms are omitted for clarity.

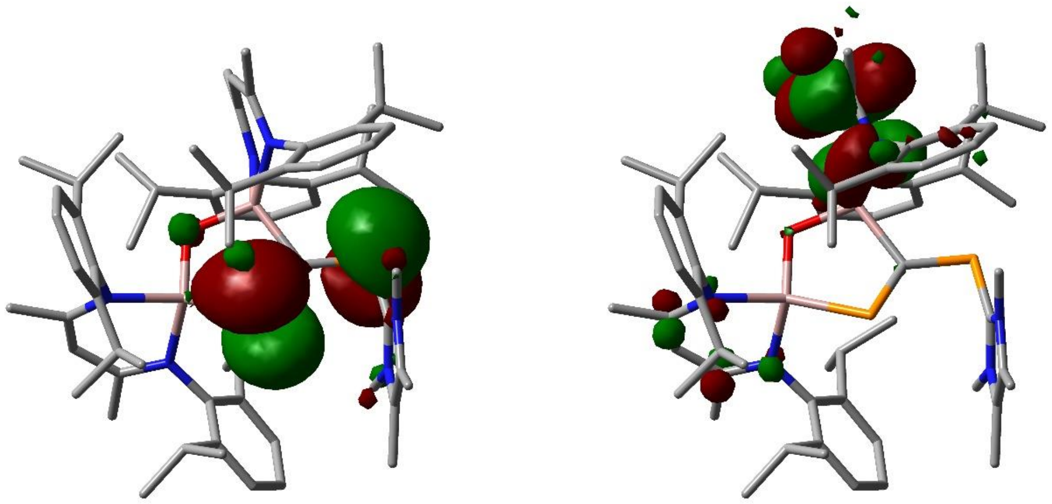

Figure S46. Occupied (left) and virtual (right) natural transition orbitals (isovalue = 0.030 au) for the transition λ_1 of **3** calculated by means of TD-PBE0-D3BJ(SMD,THF)/def2-SVP level of theory (470.68 nm, f = 0.0050). Hydrogen atoms are omitted for clarity.

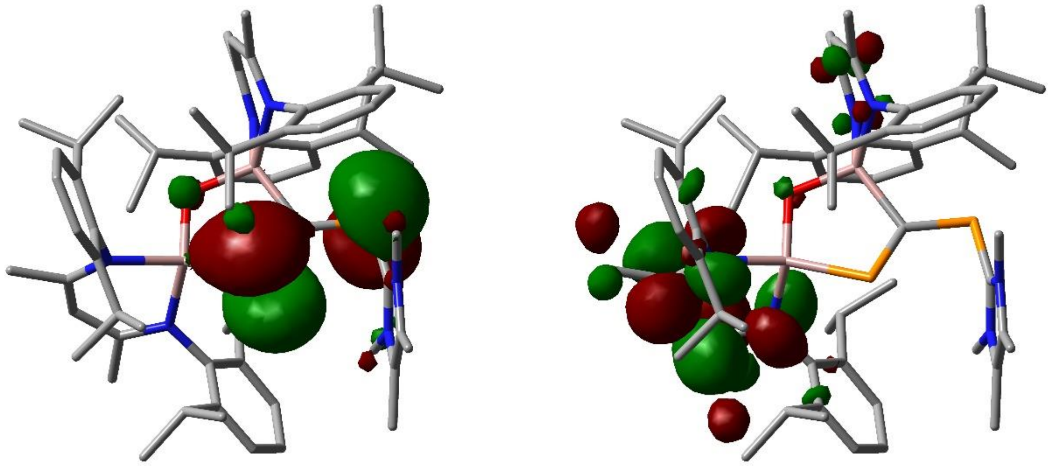

Figure S47. Occupied (left) and virtual (right) natural transition orbitals (isovalue = 0.030 au) for the transition λ_2 of **3** calculated by means of TD-PBE0-D3BJ(SMD,THF)/def2-SVP level of theory (457.48 nm, f = 0.0005). Hydrogen atoms are omitted for clarity.

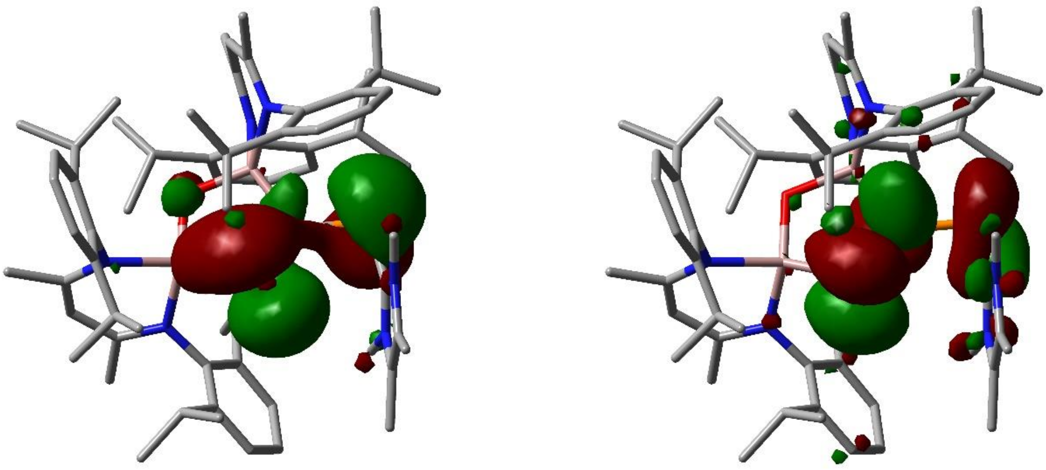

Figure S48. Occupied (left) and virtual (right) natural transition orbitals (isovalue = 0.030 au) for the transition λ_3 of **3** calculated by means of TD-PBE0-D3BJ(SMD,THF)/def2-SVP level of theory (429.17 nm, f = 0.2010). Hydrogen atoms are omitted for clarity.

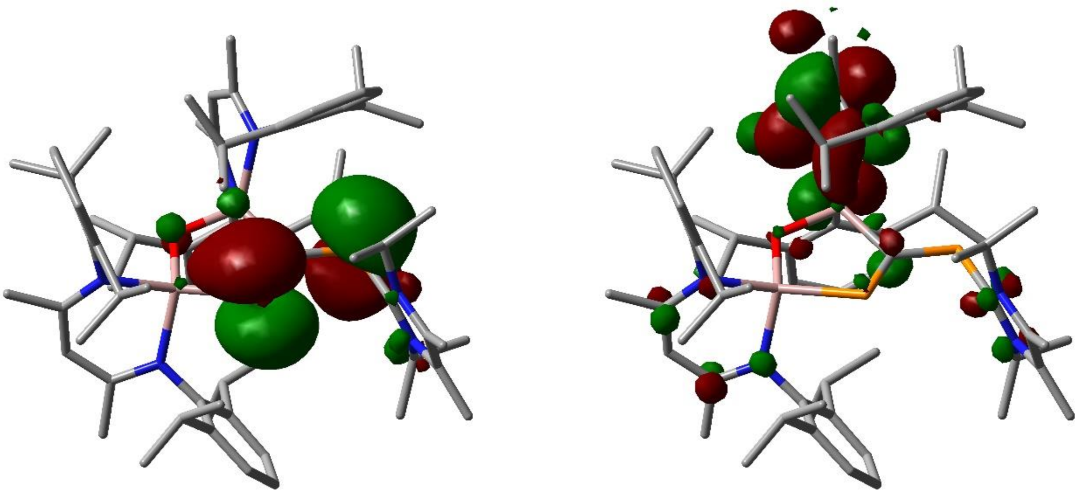

Figure S49. Occupied (left) and virtual (right) natural transition orbitals (isovalue = 0.030 au) for the transition λ_1 of **4** calculated by means of TD-PBE0-D3BJ(SMD,THF)/def2-SVP level of theory (477.87 nm, f = 0.0262). Hydrogen atoms are omitted for clarity.

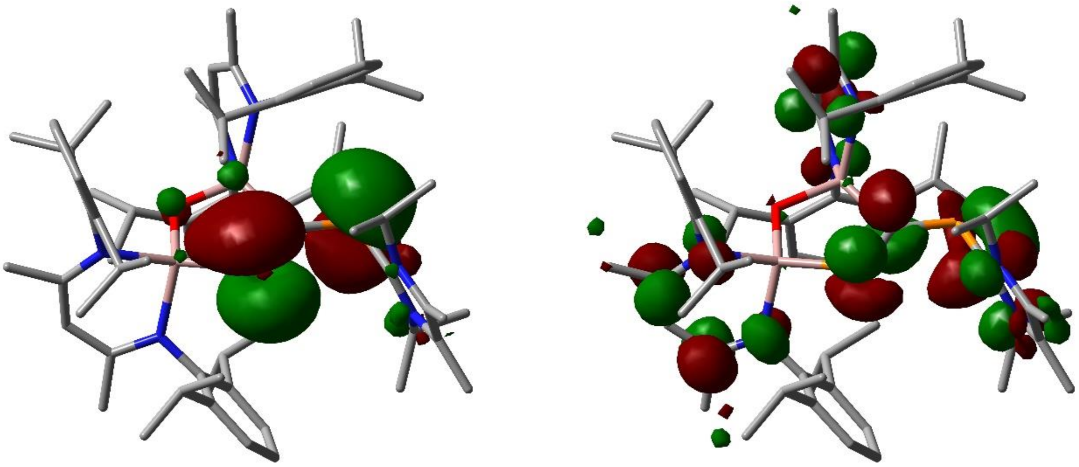

Figure S50. Occupied (left) and virtual (right) natural transition orbitals (isovalue = 0.030 au) for the transition λ_2 of **4** calculated by means of TD-PBE0-D3BJ(SMD,THF)/def2-SVP level of theory (462.51 nm, f = 0.1202). Hydrogen atoms are omitted for clarity.

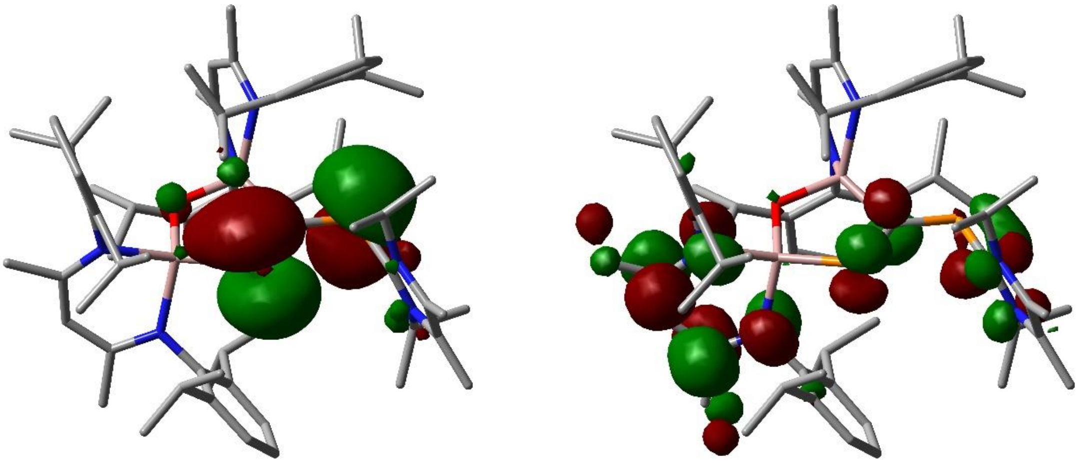

Figure S51. Occupied (left) and virtual (right) natural transition orbitals (isovalue = 0.030 au) for the transition λ_3 of **4** calculated by means of TD-PBE0-D3BJ(SMD,THF)/def2-SVP level of theory (459.02 nm, f = 0.0575). Hydrogen atoms are omitted for clarity.

6. Cartesian Coordinates and Absolute Energies for the Calculated Compound

Table S3. Absolute energies [au] of the calculated compounds by means of different methods.

Compound	*E*[a]	*G*[a]	*E*[b]
**1**	-7117.908404	-7116.769972	-7121.392846
TS-**1**	-7117.866524	-7116.729604	-7121.353648
Int-**1**	-7117.894501	-7116.757053	-7121.386668
TS-**2**	-7117.85555	-7116.719799	-7121.356677
Int-**2**	-7117.883697	-7116.746984	-7121.377373
TS-**3**	-7117.866957	-7116.726851	-7121.355707
**2**	-7117.914977	-7116.774319	-7121.397577

[a] PBE-D3BJ/def2-SVP.
[b] PBE-D3BJ(THF as solvent)/def2-TZVP//PBE-D3BJ/def2-SVP

Table S4. Absolute energies [au] of the calculated compounds by means of different methods.

Compound	*E*[a]	*G*[a]	*E*[b]
**1**	-7118.383810	-7117.199557	-7121.837100
TS-**1**	-7118.33785	-7117.156071	-7121.793403
Int-**1**	-7118.370812	-7117.188198	-7121.832377
TS-**2**	-7118.328072	-7117.146251	-7121.799760
Int-**2**	-7118.357969	-7117.174100	-7121.820931
TS-**3**	-7118.338231	-7117.152670	-7121.795936
**2**	-7118.390575	-7117.204999	-7121.841793
**S1**	-5410.974588	-5410.635437	-5412.666189
TS-**9**	-5410.932166	-5410.591637	-5412.634995
Int-**6**	-5410.956915	-5410.615872	-5412.657039
TS-**10**	-5410.926627	-5410.585439	-5412.634847
Int-**7**	-5410.958180	-5410.617989	-5412.658837
TS-**11**	-5410.926917	-5410.591865	-5412.621926
**S2**	-5410.978546	-5410.639840	-5412.670553

[a] PBE0-D3BJ/def2-SVP.
[b] PBE0-D3BJ(THF as solvent)/def2-TZVP//PBE0-D3BJ/def2-SVP

Table S5. Absolute energies [au] of the calculated compounds by means of different methods.

Compound	*E*[a]	*G*[a]	*E*[b]
**2**	-7118.390575	-7117.204999	-7121.841793
$TMSCHN_2$	-556.713335	-556.614042	-557.112765
N_2	-109.315467	-109.328140	-109.441044
TS-**4**	-7675.100069	-7673.791680	-7678.945449
Int-**3**	-7675.113130	-7673.803293	-7678.958043
TS-**5**	-7675.107953	-7673.798597	-7678.951540
Int-**4**	-7565.852469	-7564.549691	-7569.574901
TS-**8**	-7565.831909	-7564.528176	-7569.554346
**6**	-7565.878492	-7564.570738	-7569.600731
TS-**6**	-7675.109948	-7673.799508	-7678.953727
Int-**5**	-7675.156428	-7673.842386	-7679.004713
TS-**7**	-7675.135037	-7673.824409	-7678.982819

[a] PBE0-D3BJ/def2-SVP.
[b] PBE0-D3BJ(toluene as solvent)/def2-TZVP//PBE0-D3BJ/def2-SVP

Cartesian coordinates of the optimized geometry of **1** at PBE-D3BJ/def2-SVP level of theory:

Ga	-1.67106500	-0.08630200	0.64400000
Ga	1.66044500	0.10023300	-0.47706500
P	0.42763800	0.04980700	1.41695400

P	0.80342000	0.13140200	-2.92384800
O	-1.64242800	-0.14097700	-1.50360100
C	-0.60694000	-0.02404700	-2.13674400
N	-3.10956900	1.29443700	0.88373100
N	-2.93116300	-1.61381000	0.95313100
N	2.93737800	1.64028600	-0.74925800
N	3.08201200	-1.28932600	-0.84020900
C	-4.21465000	1.05188600	1.61903400
C	-4.59272600	-0.23036800	2.06508400
H	-5.48936300	-0.26992600	2.69509100
C	-4.05201100	-1.47624200	1.69163800
C	-5.12559800	2.19928100	1.98976400
H	-6.02569100	1.83329800	2.51441300
H	-5.43630700	2.77512600	1.09707100
H	-4.59797300	2.91906800	2.64537300
C	-4.79753000	-2.71044900	2.14394600
H	-5.72636100	-2.43736600	2.67504900
H	-4.16415000	-3.31746900	2.81995100
H	-5.04913800	-3.37036500	1.29192100
C	-2.95237900	2.62345300	0.35154800
C	-2.32964800	3.63955800	1.12555300
C	-2.30351200	4.94740900	0.60270100
H	-1.82425200	5.74643500	1.18867900
C	-2.88019500	5.25045800	-0.63485400
H	-2.86168600	6.28321600	-1.01533600
C	-3.46233500	4.23121000	-1.39789100
H	-3.89594800	4.46834700	-2.38123100
C	-3.50079900	2.90434600	-0.93223500
C	-1.70985700	3.37443800	2.49367000
H	-1.87539700	2.30512800	2.73786900
C	-2.35275200	4.22716200	3.60315700
H	-3.45258800	4.09862400	3.66204100
H	-2.15556500	5.30785000	3.44651200
H	-1.92816300	3.95457500	4.59071700
C	-0.19367600	3.60945000	2.45863000
H	0.25192100	3.40330400	3.45355600
H	0.04730700	4.65771600	2.19201100
H	0.29654200	2.93605600	1.72922500
C	-4.15639100	1.82074400	-1.78431000
H	-3.87591400	0.84574300	-1.34315700
C	-5.69270100	1.91089800	-1.75072600
H	-6.14204800	1.11024600	-2.37442300
H	-6.04636000	2.88643400	-2.14529800
H	-6.09181800	1.79582500	-0.72408500
C	-3.64208800	1.83256800	-3.23215400
H	-4.03468400	0.95578500	-3.78622400
H	-2.53655800	1.79230900	-3.27032500
H	-3.96936400	2.74059800	-3.77981300
C	-2.63278700	-2.92721400	0.44549700
C	-1.84183100	-3.83369900	1.20077800
C	-1.67129900	-5.13897000	0.69781300
H	-1.05993600	-5.85443900	1.26866900
C	-2.27067900	-5.54422800	-0.49910400
H	-2.13739400	-6.57471100	-0.86258000
C	-3.02449500	-4.62879800	-1.24417900
H	-3.47607500	-4.94452600	-2.19700300
C	-3.20885300	-3.30690000	-0.80089100
C	-1.19801500	-3.45784700	2.53128400
H	-1.45306000	-2.39891100	2.74112600
C	-1.71894800	-4.32126600	3.69521500
H	-2.82174100	-4.27957100	3.80184900
H	-1.27499800	-3.98192800	4.65318900
H	-1.44123600	-5.38729100	3.56071100
C	0.33106800	-3.55297700	2.44895200
H	0.79053800	-3.23323400	3.40624100
H	0.72744200	-2.89143500	1.65540700
H	0.66250100	-4.59051400	2.24474900
C	-4.03883700	-2.33369500	-1.63537900

H	-3.87509100	-1.32302800	-1.21481500
C	-5.54656100	-2.62950400	-1.53993300
H	-5.77978100	-3.64865000	-1.91331300
H	-6.12295300	-1.90485200	-2.15179500
H	-5.91910500	-2.55643300	-0.49980000
C	-3.58690400	-2.29714800	-3.10335200
H	-3.78845900	-3.25710300	-3.62224100
H	-2.50465800	-2.08362400	-3.18633300
H	-4.13501900	-1.50527400	-3.65357500
C	3.98735100	1.52160600	-1.58148400
C	4.43605000	0.28634300	-2.10190700
H	5.27500000	0.34947800	-2.80568800
C	4.10907200	-1.01619800	-1.66488600
C	4.78098700	2.75176000	-1.95984600
H	5.10013900	3.30755200	-1.05606300
H	4.16398200	3.45608200	-2.55141000
H	5.67391400	2.47951000	-2.54990800
C	5.01416800	-2.14008100	-2.11419300
H	5.49478200	-2.62495400	-1.24089000
H	5.80167800	-1.76794900	-2.79308400
H	4.43931600	-2.93598300	-2.62611100
C	2.68457200	2.92399000	-0.15309500
C	3.14349900	3.15945200	1.17239700
C	3.01790100	4.45718500	1.70024800
H	3.38083400	4.66185700	2.71743800
C	2.42580000	5.48831700	0.96128600
H	2.34016800	6.49741000	1.39263300
C	1.91340900	5.22155800	-0.31191700
H	1.40968000	6.02187500	-0.87486800
C	2.02913600	3.94428900	-0.89282000
C	3.77117600	2.04198600	1.99730900
H	3.29545800	1.09725600	1.65912300
C	5.28339300	1.91999800	1.73777700
H	5.72281800	1.11283600	2.35923600
H	5.80469100	2.86639000	1.99237800
H	5.50416600	1.68071700	0.67930200
C	3.47637600	2.17313400	3.49657500
H	2.38759900	2.25945800	3.68017200
H	3.98143700	3.05162700	3.94882500
H	3.84122400	1.27724000	4.03587800
C	1.40412900	3.67921100	-2.25520000
H	1.77489600	2.69795800	-2.61579000
C	1.76281900	4.73453400	-3.31272000
H	1.35809600	4.43788400	-4.30163500
H	2.85907100	4.86676300	-3.41970600
H	1.33131900	5.72697700	-3.06769300
C	-0.11909100	3.55612800	-2.10490400
H	-0.39105200	2.79931900	-1.34321500
H	-0.58899200	3.26022400	-3.06454700
H	-0.56379400	4.51798700	-1.78358000
C	2.98344800	-2.61660400	-0.29480900
C	3.59895000	-2.88707400	0.95708800
C	3.56538000	-4.20386700	1.45179000
H	4.04491800	-4.42591000	2.41764500
C	2.93090900	-5.22972900	0.74350900
H	2.91398500	-6.25338600	1.14787500
C	2.30536800	-4.94340500	-0.47452700
H	1.79123000	-5.74633400	-1.02437500
C	2.32427800	-3.64589300	-1.01979100
C	4.30309300	-1.80271500	1.76252300
H	4.19672600	-0.85300600	1.20166500
C	5.80836900	-2.08803700	1.90116000
H	6.31513300	-1.26308000	2.44289200
H	6.29801300	-2.19299400	0.91181800
H	5.99409600	-3.02382900	2.46814300
C	3.63562700	-1.60824600	3.13338700
H	2.57660800	-1.30087400	3.01650200
H	4.16324500	-0.82526900	3.71471100

H	3.66180700	-2.54127700	3.73361200
C	1.63014200	-3.38014200	-2.34718500
H	1.89795400	-2.35298400	-2.66848400
C	2.06641500	-4.34685200	-3.45959900
H	1.75346200	-5.38972500	-3.24566000
H	3.16669300	-4.35254600	-3.60076300
H	1.60207400	-4.05563800	-4.42376000
C	0.10759400	-3.41125300	-2.16307400
H	-0.24039400	-4.42505900	-1.88473000
H	-0.40824500	-3.11339800	-3.09807700
H	-0.21736900	-2.72393400	-1.35805700

Cartesian coordinates of the optimized geometry of **2** at PBE-D3BJ/def2-SVP level of theory:

Ga	-1.58141200	-0.23385100	-0.05217900
Ga	1.58123800	0.23394800	-0.05203400
P	-1.45757000	0.80615600	-2.20050900
P	1.45828600	-0.80672900	-2.20017300
O	-0.00004500	-0.00033000	0.84618800
N	-2.38483200	-2.06490000	-0.21202300
N	-2.88344000	0.43598500	1.29831200
N	2.38451500	2.06500300	-0.21195200
N	2.88348300	-0.43588000	1.29834100
C	-3.21414800	-2.54779000	0.72921100
C	-3.65635200	-1.80648000	1.84716100
H	-4.26689700	-2.36612300	2.56744100
C	-3.58755700	-0.41522500	2.06714700
C	-3.78428100	-3.94682400	0.61138300
H	-4.88090200	-3.89590500	0.46003500
H	-3.61877400	-4.49334600	1.56093200
H	-3.34386700	-4.52292200	-0.21956700
C	-4.38997100	0.13364600	3.22510800
H	-5.02066200	0.98698900	2.90898500
H	-3.71726100	0.52406000	4.01305300
H	-5.03284300	-0.64828600	3.66634500
C	-2.25980800	-2.75988600	-1.46708800
C	-3.26744400	-2.57278100	-2.45437000
C	-3.14402800	-3.26721300	-3.67247100
H	-3.91588100	-3.13597000	-4.44626000
C	-2.05882700	-4.11279100	-3.91621800
H	-1.97795000	-4.64722900	-4.87500300
C	-1.06632700	-4.26857700	-2.94164700
H	-0.20979100	-4.92382200	-3.14942300
C	-1.13664000	-3.59962400	-1.70513500
C	-4.47989700	-1.66922800	-2.23973200
H	-4.32729500	-1.11953200	-1.28884100
C	-5.77964300	-2.48456600	-2.10598400
H	-6.63717100	-1.81820600	-1.87804100
H	-5.71533400	-3.24511000	-1.30496100
H	-6.01052900	-3.01845000	-3.05121200
C	-4.62891700	-0.62554100	-3.35875600
H	-4.83708100	-1.10366900	-4.33789600
H	-3.70913100	-0.01644900	-3.46389400
H	-5.47603200	0.05648600	-3.13991500
C	-0.04778900	-3.80434400	-0.65416700
H	0.12524600	-2.81596400	-0.17296600
C	-0.46787200	-4.79141100	0.45165700
H	-1.31414000	-4.42006200	1.05454700
H	0.38072600	-4.95817200	1.14496500
H	-0.75468800	-5.77063600	0.01460400
C	1.28521800	-4.26698900	-1.25240700
H	1.60469600	-3.60928500	-2.08484300
H	1.22662800	-5.31029000	-1.62743900
H	2.06941200	-4.24344200	-0.47332000
C	-3.00340400	1.84540000	1.53786300
C	-3.93214700	2.58177900	0.74808400

C	-4.13373400	3.94048300	1.05242300
H	-4.85558800	4.52446200	0.46375100
C	-3.41705000	4.56643300	2.08089900
H	-3.58959800	5.63021300	2.30427200
C	-2.46260000	3.84249700	2.80310400
H	-1.88021700	4.34689900	3.58971000
C	-2.23012000	2.47544600	2.55183900
C	-4.68921000	1.91456600	-0.39848800
H	-4.02199600	1.12138300	-0.79768300
C	-5.97767900	1.21710000	0.07534000
H	-6.66382100	1.94133400	0.56197500
H	-5.77035400	0.40144300	0.79375700
H	-6.51321600	0.76904700	-0.78712300
C	-4.98950500	2.87356100	-1.55820400
H	-4.07735600	3.40529000	-1.89197500
H	-5.75261900	3.63194500	-1.28591700
H	-5.38837900	2.30769400	-2.42325800
C	-1.16585100	1.73580400	3.35880300
H	-1.16276900	0.68210400	3.01821100
C	-1.43308200	1.77352000	4.87398500
H	-1.35804000	2.80548200	5.27565400
H	-0.68273800	1.15629400	5.41004200
H	-2.43739200	1.38966700	5.14368200
C	0.23567100	2.29326500	3.06292700
H	0.46540500	2.21568200	1.98625000
H	1.00638800	1.71237900	3.60908800
H	0.32882900	3.35425700	3.37491300
C	3.21405000	2.54787000	0.72913500
C	3.65649500	1.80658700	1.84698600
H	4.26717500	2.36623100	2.56715000
C	3.58780100	0.41530900	2.06695900
C	3.78401400	3.94696800	0.61123900
H	4.88061700	3.89619000	0.45973000
H	3.61858800	4.49343600	1.56083800
H	3.34338100	4.52303400	-0.21961400
C	4.39052100	-0.13358200	3.22471800
H	5.02170800	-0.98642200	2.90822700
H	3.71802200	-0.52477400	4.01244600
H	5.03292500	0.64853100	3.66631200
C	2.25952500	2.75985600	-1.46710600
C	3.26734400	2.57295100	-2.45423700
C	3.14388600	3.26723400	-3.67242600
H	3.91589700	3.13615600	-4.44608500
C	2.05846300	4.11244400	-3.91642000
H	1.97753300	4.64672800	-4.87528600
C	1.06580500	4.26807200	-2.94198200
H	0.20911500	4.92305500	-3.14994600
C	1.13618500	3.59932200	-1.70537100
C	4.48000700	1.66971600	-2.23945100
H	4.32758800	1.12018900	-1.28843100
C	5.77962500	2.48531800	-2.10597800
H	6.01058300	3.01869400	-3.05147500
H	6.63720300	1.81918400	-1.87755800
H	5.71511500	3.24631600	-1.30540300
C	4.62919100	0.62584200	-3.35827200
H	3.70959300	0.01642600	-3.46316600
H	5.47657400	-0.05584700	-3.13943100
H	4.83702400	1.10385700	-4.33753800
C	0.04729300	3.80406500	-0.65445300
H	-0.12572300	2.81571500	-0.17318200
C	0.46736000	4.79122300	0.45129700
H	0.75400400	5.77046600	0.01417500
H	1.31373500	4.41999300	1.05411000
H	-0.38118400	4.95790200	1.14469000
C	-1.28571500	4.26662400	-1.25273900
H	-1.22709400	5.30982900	-1.62802600
H	-2.06986400	4.24329200	-0.47360400
H	-1.60527000	3.60874000	-2.08500700

C	3.00349400	-1.84529400	1.53775800
C	3.93225100	-2.58155100	0.74786600
C	4.13392700	-3.94026800	1.05207300
H	4.85583900	-4.52413100	0.46335000
C	3.41725300	-4.56636700	2.08046900
H	3.58984900	-5.63016000	2.30374100
C	2.46275700	-3.84255800	2.80274500
H	1.88039100	-4.34708500	3.58928700
C	2.23023800	-2.47547900	2.55166900
C	4.68923100	-1.91420400	-0.39865800
H	4.02223000	-1.12060300	-0.79734400
C	5.97815000	-1.21743300	0.07493700
H	6.51343200	-0.76912300	-0.78755200
H	6.66428000	-1.94213600	0.56090100
H	5.77146000	-0.40206500	0.79387100
C	4.98873400	-2.87294100	-1.55881300
H	4.07619200	-3.40397000	-1.89261200
H	5.75143200	-3.63189200	-1.28694500
H	5.38778100	-2.30697600	-2.42371800
C	1.16605700	-1.73590800	3.35881900
H	1.16281200	-0.68223200	3.01816300
C	1.43365500	-1.77352900	4.87395000
H	2.43786900	-1.38927500	5.14341600
H	1.35910200	-2.80553100	5.27561000
H	0.68319900	-1.15658500	5.41017600
C	-0.23548000	-2.29348100	3.06330500
H	-1.00608600	-1.71271600	3.60975500
H	-0.32849500	-3.35451300	3.37521000
H	-0.46555000	-2.21579200	1.98670700
C	0.00036700	-0.00030500	-2.22961900

Cartesian coordinates of the optimized geometry of Int-**1** at PBE-D3BJ/def2-SVP level of theory:

Ga	-2.06624100	0.56173400	-0.31921400
Ga	2.20735400	-0.68142400	-0.62604600
P	-0.13907900	-0.68507100	0.04921400
P	1.25339700	-0.60440400	-2.74829400
O	-1.29742600	0.33795500	-2.14173700
N	-2.93089700	2.29449600	-0.05281700
N	-3.73325100	-0.50480100	-0.51488600
N	2.95754500	-2.25307300	0.36195800
N	3.73621400	0.52614100	-0.18398500
C	-4.23791200	2.49467500	-0.34023100
C	-5.14139500	1.46691100	-0.66081000
H	-6.16979300	1.79042500	-0.86634800
C	-4.93246300	0.06305000	-0.68108100
C	-4.76757400	3.90804400	-0.28387100
H	-5.80674300	3.95742600	-0.65467800
H	-4.13031800	4.58666300	-0.88521900
H	-4.74191100	4.30153100	0.75248900
C	-6.15208700	-0.79752000	-0.91275700
H	-6.00478200	-1.83015600	-0.54790500
H	-6.36606400	-0.85552800	-1.99973900
H	-7.03948100	-0.35252300	-0.42481100
C	-2.14904700	3.36979600	0.49025400
C	-2.27386600	3.67151100	1.87609400
C	-1.51254300	4.73405600	2.39325700
H	-1.59353000	4.98803900	3.46045100
C	-0.63983500	5.46407300	1.57428000
H	-0.04925400	6.28998800	1.99983500
C	-0.50922000	5.13344600	0.22189100
H	0.19243800	5.69519400	-0.41283000
C	-1.26163000	4.08834100	-0.34985300
C	-3.15557200	2.82238400	2.78788900
H	-3.93119700	2.34330900	2.15676500
C	-3.88705900	3.63581600	3.86425400

H	-4.59345200	2.98819900	4.42196100
H	-4.46484600	4.47337600	3.42320800
H	-3.18586200	4.06595500	4.60868100
C	-2.32937000	1.68519600	3.41860800
H	-1.55843200	2.09304800	4.10385400
H	-1.79909700	1.09016900	2.64707400
H	-2.97924100	0.99790000	3.99851000
C	-1.11041100	3.75580100	-1.82761600
H	-1.80837700	2.92762000	-2.06015100
C	-1.49459900	4.94663700	-2.72194900
H	-2.52967400	5.29456500	-2.52531600
H	-1.42797500	4.66230000	-3.79176000
H	-0.81549900	5.81025900	-2.56389300
C	0.30644300	3.25176800	-2.14101000
H	0.59902400	2.41612300	-1.47564900
H	1.06193000	4.05355500	-2.01924900
H	0.36261500	2.87474100	-3.18097900
C	-3.54831400	-1.92990400	-0.52429000
C	-3.32604600	-2.57526000	0.72489600
C	-3.09131800	-3.96004600	0.72851300
H	-2.90786600	-4.47513500	1.68152900
C	-3.06513700	-4.69002700	-0.46611300
H	-2.87431100	-5.77384700	-0.44525200
C	-3.25748400	-4.03546700	-1.68537000
H	-3.20825500	-4.61007500	-2.62279600
C	-3.49080500	-2.64725300	-1.74891900
C	-3.35602400	-1.78049500	2.02463700
H	-2.95892500	-0.76919300	1.78792700
C	-4.79341800	-1.58927500	2.54035700
H	-5.25866900	-2.56978000	2.77265300
H	-5.43656000	-1.07813900	1.79803500
H	-4.79962500	-0.98008800	3.46821800
C	-2.44923200	-2.36450800	3.11206200
H	-1.43308500	-2.56087000	2.71943300
H	-2.85048600	-3.31545800	3.52064500
H	-2.36476000	-1.65597100	3.96094900
C	-3.65176000	-1.98669500	-3.11452500
H	-3.79191800	-0.89924500	-2.95165900
C	-4.88184700	-2.53225200	-3.86485700
H	-4.74465500	-3.60119600	-4.12976600
H	-5.03837100	-1.97562600	-4.81149800
H	-5.81053000	-2.46088500	-3.26499600
C	-2.38868100	-2.15432600	-3.97761100
H	-1.49467400	-1.73653300	-3.48069400
H	-2.51436300	-1.62608900	-4.94479400
H	-2.19364600	-3.22345400	-4.20247600
C	4.27898100	-2.39052900	0.56276400
C	5.20380500	-1.33995900	0.37741700
H	6.25415800	-1.59509100	0.56533000
C	4.94267000	0.03169400	0.15252100
C	4.80939900	-3.71448300	1.06124700
H	4.48583800	-3.90308500	2.10403900
H	5.91337700	-3.73916900	1.02594400
H	4.40150400	-4.54948200	0.45865200
C	6.10620800	0.98261400	0.31721100
H	5.81642900	1.87270800	0.90827700
H	6.44376100	1.36118600	-0.66784600
H	6.95868700	0.48060700	0.80808700
C	2.05531300	-3.22591700	0.90796700
C	1.73796600	-3.15325900	2.29425900
C	0.87529500	-4.12390800	2.83442900
H	0.62641600	-4.08506200	3.90647800
C	0.31548500	-5.12487600	2.03149200
H	-0.35849400	-5.87529500	2.47250800
C	0.60040300	-5.15272300	0.66274300
H	0.13622100	-5.92150200	0.02666900
C	1.46347600	-4.20977400	0.07320700
C	2.25723000	-2.03318900	3.19246500

H	2.90517700	-1.37827800	2.57670100
C	3.10656900	-2.55826300	4.36243300
H	2.50684200	-3.19586500	5.04467800
H	3.50854000	-1.71506600	4.96109400
H	3.96710900	-3.16248000	4.01213900
C	1.09560300	-1.16835000	3.71222300
H	0.50066800	-0.75266800	2.87462700
H	1.47958400	-0.32281900	4.31833600
H	0.40948200	-1.75609300	4.35558500
C	1.72491700	-4.26652200	-1.42589000
H	2.31040200	-3.36406200	-1.69638600
C	2.55663800	-5.50149800	-1.81301400
H	2.01930100	-6.44129900	-1.56662100
H	3.53142200	-5.52708300	-1.28437100
H	2.76268000	-5.50748100	-2.90300800
C	0.41222500	-4.20268000	-2.22106600
H	-0.20153300	-5.11602000	-2.07458800
H	0.62177500	-4.10030800	-3.30476700
H	-0.19374300	-3.32968100	-1.91197500
C	3.55559800	1.94737100	-0.28975800
C	2.94741400	2.62644000	0.80290500
C	2.82386100	4.02575400	0.73613400
H	2.35844000	4.56511100	1.57276200
C	3.27898500	4.73951400	-0.37882800
H	3.18357800	5.83590800	-0.40940400
C	3.83259200	4.05230800	-1.46340100
H	4.15844100	4.61423900	-2.35208200
C	3.97269900	2.65072400	-1.45310300
C	2.48182400	1.86646700	2.03964700
H	2.22189000	0.83886400	1.70904400
C	3.61048900	1.74318600	3.07935600
H	3.25805500	1.19357600	3.97653300
H	3.95543900	2.74561900	3.40864600
H	4.48581600	1.19661400	2.67765700
C	1.21518000	2.46350900	2.65854600
H	0.41000000	2.56236100	1.90509700
H	1.39043800	3.46546400	3.10071200
H	0.84370100	1.80944200	3.47113100
C	4.53199400	1.95642300	-2.69149900
H	4.64667200	0.87900900	-2.45292800
C	5.90884100	2.50671300	-3.10601900
H	6.64489400	2.48588900	-2.27680800
H	5.83583500	3.55831300	-3.45220200
H	6.32436400	1.91361000	-3.94582800
C	3.54215700	2.06360200	-3.86535400
H	3.94419900	1.54485900	-4.75997100
H	3.36212500	3.12430000	-4.13860900
H	2.57154400	1.59164600	-3.60977500
C	-0.17193400	-0.22928700	-1.74084200

Cartesian coordinates of the optimized geometry of Int-**2** at PBE-D3BJ/def2-SVP level of theory:

Ga	2.09485000	-0.21944400	-0.34756800
Ga	-2.09776900	0.19371300	-0.34553900
P	1.52516700	-0.56161300	-2.52448000
P	-1.53565800	0.43012100	-2.54016800
O	-0.00226200	-0.02059900	-0.33762800
N	3.18223500	1.43660500	-0.02902700
N	2.74445000	-1.15229100	1.28657400
N	-3.20091000	-1.45323900	0.01151700
N	-2.73039300	1.16253100	1.26965600
C	3.87366900	1.68813000	1.09253400
C	3.98526200	0.76350000	2.15475900
H	4.58760500	1.09575300	3.00915700
C	3.55211900	-0.57625400	2.20521700
C	4.61378100	2.99750500	1.22425700

H	5.30645900	3.15079700	0.37352600
H	5.18316300	3.04428800	2.16958300
H	3.89899700	3.84402500	1.19140900
C	4.06125200	-1.40845100	3.36163300
H	4.44903100	-2.38383300	3.00837500
H	3.25138100	-1.63969300	4.08139100
H	4.86286800	-0.87439600	3.90188500
C	3.43340700	2.24281700	-1.19880600
C	4.45689000	1.81402200	-2.08980200
C	4.72787600	2.60195600	-3.22214500
H	5.51878800	2.28687700	-3.91955200
C	4.00230100	3.77024900	-3.48333300
H	4.22658900	4.37162800	-4.37738000
C	2.98092900	4.15953000	-2.61184500
H	2.39581700	5.06619900	-2.82929000
C	2.67409900	3.40884500	-1.46085100
C	5.26607100	0.54436600	-1.84304700
H	4.75208800	-0.02746900	-1.04315100
C	6.67849700	0.86649800	-1.32358700
H	7.24654400	-0.06559600	-1.12357900
H	6.64692800	1.45014600	-0.38211000
H	7.24915800	1.45936000	-2.06845600
C	5.31756000	-0.35990700	-3.08369700
H	5.87561200	0.11436200	-3.91688400
H	4.29596100	-0.59480200	-3.44402800
H	5.83475500	-1.31203000	-2.84619200
C	1.52314500	3.84250100	-0.56747900
H	1.50071300	3.15545100	0.30037600
C	1.70049900	5.27305300	-0.03116700
H	2.66248600	5.40514300	0.50605100
H	0.87703200	5.52342200	0.66724700
H	1.67672000	6.01924300	-0.85212600
C	0.18297000	3.69312400	-1.30445900
H	0.03429500	2.66635400	-1.69259800
H	0.12357300	4.38795900	-2.16838100
H	-0.65940300	3.92690100	-0.62576200
C	2.44520000	-2.55243800	1.42053300
C	3.11995600	-3.48242400	0.58081700
C	2.87681000	-4.85465600	0.77893000
H	3.39379500	-5.58847700	0.14452300
C	1.98948000	-5.30045600	1.76470500
H	1.81833300	-6.37816600	1.90670500
C	1.30321300	-4.37023000	2.55338400
H	0.58682700	-4.72492600	3.30932000
C	1.50647600	-2.98663200	2.39619700
C	4.11337400	-3.02157400	-0.48051500
H	3.78254400	-2.01623200	-0.81961500
C	5.52755200	-2.86515700	0.10578700
H	5.89646400	-3.83054500	0.51071300
H	5.55497700	-2.11869200	0.92383700
H	6.23917100	-2.52919500	-0.67603700
C	4.11578600	-3.90578100	-1.73313400
H	3.09308400	-4.01426000	-2.14475100
H	4.52732400	-4.91714700	-1.53493900
H	4.74672500	-3.44443100	-2.51832100
C	0.69858400	-2.00398200	3.23803500
H	1.19718600	-1.01715300	3.17494700
C	0.62229200	-2.39485300	4.72227500
H	0.01212300	-3.30817900	4.87772400
H	0.14480600	-1.58262900	5.30771300
H	1.62419000	-2.58769400	5.15752500
C	-0.70897900	-1.82489900	2.64845000
H	-0.65465500	-1.40999600	1.62434900
H	-1.31653000	-1.13733800	3.27006400
H	-1.24825100	-2.79202200	2.59698700
C	-3.92244500	-1.65428300	1.12165500
C	-4.00771600	-0.71166600	2.17351600
H	-4.62983900	-1.01267400	3.02588900

C	-3.55518500	0.62017000	2.19509000
C	-4.78416100	-2.88930600	1.24896400
H	-5.79460800	-2.68749600	0.83745600
H	-4.90646100	-3.16776400	2.31264600
H	-4.37220900	-3.74294600	0.68209400
C	-4.07325600	1.49832700	3.31166900
H	-4.54810400	2.41060700	2.89842300
H	-3.25619400	1.85092600	3.97001900
H	-4.81261100	0.95561300	3.92657700
C	-3.45599500	-2.26890300	-1.14922700
C	-4.60563500	-1.99098600	-1.93864000
C	-4.83939000	-2.78457900	-3.07658600
H	-5.72153300	-2.57937400	-3.70259800
C	-3.96302800	-3.81355000	-3.43571300
H	-4.16161500	-4.42138600	-4.33147400
C	-2.82249100	-4.05420200	-2.66191500
H	-2.12429600	-4.85081400	-2.95713400
C	-2.54121100	-3.28933200	-1.51548900
C	-5.54711700	-0.82695600	-1.64166000
H	-5.23649200	-0.36779800	-0.68105200
C	-7.01020400	-1.27401300	-1.48307900
H	-7.41211700	-1.68909200	-2.43019500
H	-7.65218800	-0.41474900	-1.19992500
H	-7.12417400	-2.05632700	-0.70567300
C	-5.42205200	0.25573300	-2.72885900
H	-4.37091800	0.58385500	-2.85125600
H	-6.04353600	1.13958700	-2.47614300
H	-5.76436700	-0.12776200	-3.71200700
C	-1.31088900	-3.59525900	-0.67434800
H	-1.08061900	-2.68407000	-0.08697800
C	-1.60513200	-4.73325200	0.32102400
H	-1.89625800	-5.65813600	-0.21976600
H	-2.42859600	-4.47929900	1.01738600
H	-0.70510400	-4.95774300	0.92608500
C	-0.06884900	-3.91869800	-1.51380700
H	-0.17607800	-4.87732000	-2.06372100
H	0.81045300	-4.02098400	-0.84886200
H	0.15326000	-3.10866300	-2.23613900
C	-2.42261100	2.56454000	1.36370900
C	-3.11361900	3.48393200	0.52806900
C	-2.83387800	4.85669500	0.67237400
H	-3.36776100	5.58087100	0.03874600
C	-1.88932000	5.31394100	1.59657700
H	-1.68469800	6.39102700	1.69149700
C	-1.19527500	4.39299800	2.39055500
H	-0.44033000	4.75406800	3.10535000
C	-1.44542800	3.01199400	2.29608700
C	-4.15820200	3.04071000	-0.49017800
H	-4.12864200	1.93266400	-0.54444500
C	-5.57601400	3.42951500	-0.03560400
H	-6.33279700	3.06104300	-0.75844500
H	-5.68695900	4.53146200	0.03675800
H	-5.81891900	3.00155600	0.95826600
C	-3.84158100	3.56696200	-1.89841600
H	-2.85321500	3.19852700	-2.24046000
H	-3.83791200	4.67579900	-1.93350500
H	-4.60326000	3.21390100	-2.62178500
C	-0.65677300	2.04276800	3.17002600
H	-1.15263100	1.05471100	3.10563100
C	-0.62187700	2.45592700	4.65042000
H	-1.63622000	2.63815300	5.06027700
H	-0.03160900	3.38239000	4.80521500
H	-0.14566700	1.66035100	5.25955100
C	0.76675300	1.85976300	2.62613000
H	1.34532100	1.15389500	3.25422400
H	1.31889800	2.82183400	2.61687200
H	0.74425400	1.46875300	1.59148600
C	-0.00593100	-0.05583000	-1.76317900

Cartesian coordinates of the optimized geometry of TS-**1** at PBE-D3BJ/def2-SVP level of theory:

Ga	1.86826100	0.36635600	0.53393700
Ga	-1.77333100	-0.29592200	-0.75931500
P	-0.06393300	-0.81073300	0.71292800
P	-0.62419500	-0.06976400	-2.81543600
O	1.82153500	0.17436700	-1.48404900
C	0.60337200	-0.15357400	-1.63349400
N	3.45866500	-0.76729000	0.92084500
N	2.75658500	2.08901800	0.84384300
N	-2.78902200	-2.03992400	-0.77034600
N	-3.41860600	0.84571000	-0.70742400
C	4.54530100	-0.28591800	1.53985100
C	4.70115900	1.07367800	1.90432500
H	5.61493600	1.30791400	2.46528300
C	3.91949000	2.17985300	1.52968600
C	5.71523600	-1.19008200	1.85807000
H	6.01894400	-1.05064900	2.91416000
H	6.58617000	-0.90479100	1.23406100
H	5.49335300	-2.25565700	1.67751200
C	4.42433400	3.55233800	1.90766100
H	5.38797300	3.48601600	2.44271600
H	3.68924300	4.07550400	2.55155600
H	4.55213500	4.19107000	1.01185900
C	3.41744200	-2.13367200	0.46375700
C	2.80802400	-3.12097700	1.28628900
C	2.78739000	-4.44832100	0.82034900
H	2.31516300	-5.22562600	1.43816600
C	3.35221300	-4.79460600	-0.41229800
H	3.32835800	-5.84029500	-0.75513900
C	3.93041300	-3.80646900	-1.21491600
H	4.35637700	-4.08056500	-2.19201000
C	3.96679200	-2.45955900	-0.80655700
C	2.24407100	-2.78825700	2.66418900
H	2.03376500	-1.69823000	2.67777400
C	3.27188500	-3.08387200	3.77297800
H	4.21073900	-2.51358500	3.64128200
H	3.53477300	-4.16239000	3.78548800
H	2.85492800	-2.82550900	4.76817200
C	0.91966400	-3.50505700	2.95154400
H	0.49719600	-3.14755400	3.91286600
H	1.04811900	-4.60344500	3.04241000
H	0.18086600	-3.30365500	2.15273100
C	4.61251200	-1.42101000	-1.72009300
H	4.41795000	-0.42198700	-1.28175200
C	6.13820700	-1.61653600	-1.79958200
H	6.60278900	-0.81925300	-2.41570600
H	6.38909400	-2.59095900	-2.26814600
H	6.61505300	-1.59933200	-0.80057900
C	4.00008100	-1.42862500	-3.13082900
H	4.43063500	-0.60582400	-3.73806700
H	2.90515100	-1.28518200	-3.09340300
H	4.21418500	-2.37878500	-3.66258600
C	2.15102400	3.29841600	0.35276400
C	1.24081500	4.01271000	1.17598400
C	0.71065500	5.22085500	0.68567800
H	-0.00515000	5.78338900	1.30327400
C	1.09106900	5.72282200	-0.56402400
H	0.68056500	6.67974200	-0.92119300
C	1.97895100	4.99800200	-1.36728800
H	2.25688900	5.38716200	-2.35870300
C	2.51192800	3.76761100	-0.94023000
C	0.84019200	3.50218800	2.55449900
H	1.55756500	2.70351100	2.83458800
C	0.92165200	4.59305300	3.63484000

H	1.91608400	5.08467200	3.65648600
H	0.73317400	4.15647200	4.63658000
H	0.16092100	5.38485100	3.47655200
C	-0.55932100	2.86934300	2.51400800
H	-0.84579700	2.49189900	3.51673100
H	-0.59865200	2.01551100	1.80774000
H	-1.32234100	3.60567200	2.19336600
C	3.46582500	2.99748000	-1.84876800
H	3.66608300	2.02040800	-1.36728900
C	4.81356400	3.72524200	-2.00056400
H	4.68362100	4.71922100	-2.47732200
H	5.50444100	3.13533700	-2.63744000
H	5.31123700	3.88503900	-1.02294600
C	2.83658900	2.70397700	-3.22045300
H	2.64040500	3.63625700	-3.78997800
H	1.88502800	2.14925700	-3.11510700
H	3.52266300	2.07938000	-3.82854100
C	-3.95232000	-2.08269400	-1.43669700
C	-4.67650100	-0.92463600	-1.81576300
H	-5.58990200	-1.11069500	-2.39408000
C	-4.50759200	0.40367400	-1.36764300
C	-4.56538500	-3.42266000	-1.77302500
H	-4.69031800	-4.04144500	-0.86262600
H	-3.89582900	-3.99694700	-2.44297500
H	-5.54743100	-3.30057500	-2.26334100
C	-5.65395600	1.36075000	-1.59827500
H	-6.12182200	1.64084300	-0.63269300
H	-6.42673100	0.90561100	-2.24278400
H	-5.30424700	2.30648200	-2.05491100
C	-2.25736300	-3.25708400	-0.22031500
C	-2.58263300	-3.58500200	1.12716900
C	-2.15757100	-4.82719000	1.63175900
H	-2.40586300	-5.10261600	2.66635500
C	-1.40194600	-5.71020100	0.85033700
H	-1.07873400	-6.67683700	1.26622900
C	-1.03203300	-5.34116800	-0.44551700
H	-0.40027300	-6.01493200	-1.04450000
C	-1.44276000	-4.11551000	-1.00514600
C	-3.35166700	-2.60809500	2.01341000
H	-3.05831700	-1.59072300	1.67696000
C	-4.87763000	-2.72708700	1.84493000
H	-5.39918400	-2.02497000	2.52838000
H	-5.22305700	-3.75389600	2.08739000
H	-5.20484300	-2.48724000	0.81551600
C	-2.96547900	-2.72747300	3.49359100
H	-1.86995400	-2.64425200	3.63097700
H	-3.29954200	-3.68900300	3.93518800
H	-3.44695200	-1.92161300	4.08102900
C	-0.94800400	-3.73330800	-2.39330600
H	-1.39085300	-2.74959900	-2.65244200
C	-1.35344000	-4.74336700	-3.47920900
H	-1.00774200	-4.39695900	-4.47455000
H	-2.45204100	-4.88523100	-3.53546000
H	-0.89996500	-5.74045800	-3.29933500
C	0.57811800	-3.54770300	-2.36885600
H	0.88573800	-2.87197300	-1.54697500
H	0.93480100	-3.11155600	-3.32403200
H	1.09772900	-4.51568600	-2.21672500
C	-3.43068200	2.16402200	-0.14046300
C	-3.95426200	2.34727100	1.16865900
C	-3.98702900	3.64860200	1.70291900
H	-4.39287500	3.80356600	2.71463700
C	-3.50326400	4.74369500	0.97724200
H	-3.53195300	5.75353800	1.41453500
C	-2.97873400	4.54620700	-0.30479500
H	-2.59392400	5.40616100	-0.87411900
C	-2.94216600	3.26716500	-0.89285000
C	-4.46551700	1.17875500	2.00238300

H	-4.43945400	0.27498100	1.36169000
C	-5.92042900	1.38085900	2.45727300
H	-6.29178200	0.47658800	2.98183600
H	-6.59564400	1.57639800	1.59970900
H	-6.01738100	2.23443700	3.15976200
C	-3.53883700	0.91832100	3.20155500
H	-2.52171600	0.63215700	2.86825400
H	-3.93809300	0.09860100	3.83161900
H	-3.44799700	1.82008200	3.84169600
C	-2.40347900	3.10893900	-2.30853000
H	-2.47907000	2.03686600	-2.57980900
C	-3.22313800	3.91035400	-3.33474300
H	-3.15501800	5.00207400	-3.14631700
H	-4.29747000	3.63630600	-3.31844200
H	-2.84254100	3.72449000	-4.35976900
C	-0.92013300	3.49302000	-2.38026700
H	-0.77631500	4.57551300	-2.19337000
H	-0.50204800	3.25542200	-3.37872100
H	-0.32073700	2.94161500	-1.63016700

Cartesian coordinates of the optimized geometry of TS-**2** at PBE-D3BJ/def2-SVP level of theory:

Ga	2.66294600	0.12244500	-0.14661700
Ga	-2.55156500	-0.06198200	-0.14304900
P	1.22269000	-0.47485000	-1.87788000
P	-1.29157600	1.41668800	-1.15260900
O	0.91328900	0.95132400	0.37964500
N	3.83171100	1.72628700	-0.32372200
N	3.59655800	-0.63464500	1.41733200
N	-3.14330100	-1.93459900	-0.26123100
N	-3.96877500	0.51661600	1.10991400
C	4.82852100	2.02240600	0.51339000
C	5.15273100	1.23110100	1.64554800
H	5.97541000	1.61179000	2.26436100
C	4.60781500	0.00163800	2.05309000
C	5.67619400	3.25166500	0.28361700
H	6.73940600	3.03095700	0.49755200
H	5.36224300	4.05880600	0.97670600
H	5.57684200	3.63632700	-0.74737300
C	5.22238100	-0.66533800	3.26224000
H	5.72455200	-1.61254900	2.97835100
H	4.44328800	-0.94283600	3.99943200
H	5.96242400	-0.00405500	3.74667700
C	3.50853300	2.53847500	-1.46221000
C	3.88596900	2.06094900	-2.74881600
C	3.50756400	2.81236100	-3.87419500
H	3.79139600	2.46179400	-4.87710000
C	2.76415300	3.99144300	-3.73856200
H	2.46806900	4.56196000	-4.63193800
C	2.38764700	4.43522700	-2.46739500
H	1.79018400	5.35440600	-2.36840900
C	2.74394400	3.72519400	-1.30410000
C	4.72457600	0.79663400	-2.90250500
H	4.53371000	0.16743100	-2.00646900
C	6.22702300	1.13206600	-2.90317000
H	6.83818500	0.20793200	-2.96989700
H	6.52737200	1.67309600	-1.98398300
H	6.48491400	1.77581000	-3.76987400
C	4.33372000	-0.04766600	-4.12131400
H	4.59039400	0.45439000	-5.07718000
H	3.24576300	-0.26034200	-4.11642100
H	4.87695300	-1.01470600	-4.10587900
C	2.30252900	4.25531400	0.05698800
H	2.65417000	3.54068200	0.82830700
C	2.93094300	5.63175600	0.34941200
H	4.03405400	5.62480200	0.24682000

H	2.68114000	5.96286800	1.37846400
H	2.54128500	6.40169200	-0.34844300
C	0.77156400	4.33182400	0.17582700
H	0.29693400	3.34529000	0.01174000
H	0.34376700	5.04593200	-0.55809200
H	0.48244500	4.68274800	1.18817800
C	3.25984000	-1.97069500	1.81971300
C	4.03282800	-3.05290400	1.30927600
C	3.72487300	-4.35459500	1.74252800
H	4.30972400	-5.20381200	1.35956600
C	2.68351800	-4.58809900	2.65080000
H	2.46158400	-5.61340200	2.98335800
C	1.91637900	-3.51644800	3.11883200
H	1.09204200	-3.70570300	3.82371200
C	2.17868200	-2.19282300	2.71091500
C	5.13110900	-2.81691000	0.27654500
H	5.45814500	-1.76135800	0.37112100
C	6.37019800	-3.69691400	0.49294000
H	6.15634300	-4.76978600	0.30804200
H	6.76444200	-3.60500800	1.52556700
H	7.17648200	-3.40293200	-0.20904800
C	4.56669800	-2.98882100	-1.14714400
H	3.70071000	-2.31911400	-1.32574500
H	4.21729000	-4.02969900	-1.30826700
H	5.34065700	-2.76124300	-1.90920500
C	1.31207600	-1.05534400	3.23992700
H	1.67820600	-0.11155300	2.78913500
C	1.42034500	-0.92111200	4.76890000
H	1.02628300	-1.81985800	5.28745800
H	0.83546600	-0.04748600	5.12343300
H	2.47046700	-0.78029400	5.09626900
C	-0.15132500	-1.22435300	2.80029300
H	-0.23384200	-1.16109500	1.69793500
H	-0.77809300	-0.41834300	3.22534000
H	-0.57432200	-2.19495900	3.12954200
C	-4.09969300	-2.45154900	0.54073800
C	-4.74731900	-1.73044000	1.56663300
H	-5.44240300	-2.31161300	2.18528900
C	-4.74419600	-0.33563300	1.79957300
C	-4.57995000	-3.86181700	0.28813500
H	-5.29117500	-3.86430500	-0.56365500
H	-5.10716500	-4.25969200	1.17431800
H	-3.75429900	-4.54049900	0.00779600
C	-5.68265100	0.20917700	2.84846500
H	-6.30460500	1.02735700	2.43437000
H	-5.11012300	0.65317500	3.68727900
H	-6.33996800	-0.58327500	3.24821300
C	-2.75266800	-2.67756700	-1.43650900
C	-3.58072500	-2.60026500	-2.58829800
C	-3.23203600	-3.37110400	-3.71291700
H	-3.86158200	-3.32237300	-4.61456600
C	-2.09340700	-4.18102600	-3.70678100
H	-1.83595800	-4.78005800	-4.59342700
C	-1.26144200	-4.20306500	-2.58139300
H	-0.34651700	-4.80986100	-2.60175300
C	-1.56016900	-3.45106300	-1.42981800
C	-4.77757900	-1.65859000	-2.67580700
H	-4.95925600	-1.23447300	-1.66703000
C	-6.07113800	-2.36931800	-3.10481500
H	-5.99925300	-2.76398600	-4.13910600
H	-6.92663300	-1.66349700	-3.08039800
H	-6.31526600	-3.22226700	-2.43919800
C	-4.44528900	-0.48510100	-3.61650700
H	-3.54684200	0.06619500	-3.26820500
H	-5.29497400	0.22537800	-3.67288600
H	-4.24074600	-0.84882200	-4.64447700
C	-0.61448500	-3.45558000	-0.23288400
H	-0.54589000	-2.39839100	0.10701500

C	-1.13394500	-4.29917600	0.94634600
H	-1.34262000	-5.34065200	0.62303600
H	-2.05426700	-3.88405800	1.39806200
H	-0.36386900	-4.34324000	1.74175800
C	0.81012700	-3.88888100	-0.59262200
H	0.86722800	-4.97330000	-0.82530900
H	1.48320000	-3.70372900	0.26465700
H	1.19654800	-3.31057700	-1.45432300
C	-4.10209600	1.93728000	1.28070100
C	-4.82515500	2.66512400	0.29457200
C	-4.98321100	4.05050900	0.47671800
H	-5.54610100	4.63229000	-0.26639100
C	-4.41837400	4.70633900	1.57738400
H	-4.55550500	5.79114100	1.70224700
C	-3.64826000	3.98559800	2.49419800
H	-3.16278000	4.51358600	3.32903000
C	-3.46030800	2.59667400	2.36051600
C	-5.38829200	1.96875500	-0.94144100
H	-4.66482200	1.16614900	-1.21038000
C	-6.73858600	1.28373000	-0.66611600
H	-7.11413800	0.77992200	-1.58095600
H	-7.49913000	2.02807300	-0.35071000
H	-6.66201400	0.51571900	0.12751000
C	-5.47949900	2.89372600	-2.16068700
H	-4.51010100	3.39159300	-2.35978100
H	-6.25558900	3.67637900	-2.03279200
H	-5.75445200	2.31035100	-3.06151700
C	-2.52079400	1.87685400	3.32061100
H	-2.61022300	0.78769300	3.12706600
C	-2.87706300	2.11697700	4.79712500
H	-3.93318400	1.86145500	5.02166500
H	-2.72465100	3.17701400	5.08684900
H	-2.22979200	1.50179600	5.45522400
C	-1.06246100	2.28112200	3.02903800
H	-0.37080200	1.79414100	3.74801700
H	-0.93284800	3.37821500	3.13301100
H	-0.75146000	1.99467100	2.00404300
C	0.33436100	0.57222600	-0.75879000

Cartesian coordinates of the optimized geometry of TS-**2** at PBE-D3BJ/def2-SVP level of theory:

Ga	-1.89289100	0.02918800	-0.23846200
Ga	1.89288100	-0.02919100	-0.23853900
P	-1.49707300	0.71292200	-2.40564900
P	1.49697200	-0.71289300	-2.40572000
O	0.00000000	-0.00000100	0.01479700
N	-2.83413700	-1.74275800	-0.15211600
N	-2.80112300	0.80618300	1.34184400
N	2.83413300	1.74275300	-0.15222200
N	2.80116900	-0.80619100	1.34173300
C	-3.62306900	-2.10421800	0.86795300
C	-3.86817200	-1.28639100	1.99696500
H	-4.49096300	-1.74079200	2.77816400
C	-3.56971400	0.07615700	2.17982700
C	-4.37570400	-3.41645300	0.82945800
H	-5.44809700	-3.22551500	0.62084000
H	-4.32207100	-3.91000000	1.81936700
H	-3.99547500	-4.10235300	0.05331000
C	-4.17953200	0.75291200	3.38670400
H	-4.66279600	1.70985800	3.10920100
H	-3.40034700	1.00390800	4.13333000
H	-4.92526200	0.09697700	3.86960600
C	-2.88388300	-2.47590300	-1.39120900
C	-3.96150200	-2.23480900	-2.28689300
C	-3.98798300	-2.94557400	-3.50152600
H	-4.81444000	-2.77168400	-4.20783800

C	-2.97714900	-3.85217100	-3.83155300
H	-3.01143400	-4.39414800	-4.78887300
C	-1.91011500	-4.05625000	-2.94848300
H	-1.10931900	-4.75589300	-3.22406100
C	-1.83485400	-3.37715400	-1.71876000
C	-5.06900500	-1.22362000	-1.99973300
H	-4.85241500	-0.74557900	-1.02273400
C	-6.45022800	-1.89468900	-1.89190100
H	-7.22429000	-1.15248200	-1.60693900
H	-6.46137000	-2.70810200	-1.14015900
H	-6.75521700	-2.33851700	-2.86212400
C	-5.09823400	-0.10972100	-3.06079500
H	-5.36512500	-0.51294400	-4.05929000
H	-4.11005000	0.38278600	-3.15352000
H	-5.85585200	0.65744300	-2.79931800
C	-0.67938900	-3.63315700	-0.75698000
H	-0.42890400	-2.65687800	-0.28780200
C	-1.08133000	-4.61392500	0.36089300
H	-1.89910600	-4.22469700	0.99373400
H	-0.21359400	-4.81180500	1.02084800
H	-1.41344100	-5.58122200	-0.07114100
C	0.58980000	-4.14171100	-1.44863100
H	0.89541100	-3.46860900	-2.27405100
H	0.46212400	-5.17134100	-1.84467500
H	1.41834000	-4.17482600	-0.71596600
C	-2.70212200	2.22463200	1.54552300
C	-3.51246500	3.08114600	0.74710600
C	-3.46076500	4.46535700	0.99531600
H	-4.08356000	5.14361000	0.39493600
C	-2.62432700	4.99488600	1.98582100
H	-2.60045900	6.08048800	2.16458600
C	-1.80402900	4.14181200	2.73168900
H	-1.13055000	4.56488200	3.49261500
C	-1.81950500	2.74857500	2.52809900
C	-4.43214700	2.51713800	-0.33242000
H	-3.94544200	1.59686800	-0.72032100
C	-5.79669900	2.09803500	0.24442700
H	-6.31676700	2.96630500	0.70021900
H	-5.69629800	1.31343300	1.01917400
H	-6.44858000	1.69242300	-0.55659900
C	-4.60427800	3.45699900	-1.53211700
H	-3.62372400	3.77416300	-1.93795000
H	-5.18638400	4.36566800	-1.27308800
H	-5.15393100	2.93836100	-2.34213900
C	-0.88336200	1.85849100	3.33865100
H	-1.17744700	0.80765300	3.14919600
C	-0.97343600	2.11449000	4.85243300
H	-0.57438500	3.11376500	5.12292600
H	-0.37269000	1.36286500	5.40495900
H	-2.01520300	2.06312900	5.22913800
C	0.56650600	2.01024800	2.85369900
H	0.65690000	1.71615800	1.79148900
H	1.24933600	1.36988700	3.44711700
H	0.91887000	3.05624200	2.95676000
C	3.62310000	2.10421100	0.86782000
C	3.86823700	1.28638300	1.99682400
H	4.49105300	1.74078200	2.77800300
C	3.56978400	-0.07616500	2.17969400
C	4.37573900	3.41644400	0.82930200
H	5.44812600	3.22550100	0.62065800
H	4.32213200	3.90999400	1.81921000
H	3.99549300	4.10234300	0.05316100
C	4.17964200	-0.75291900	3.38655100
H	4.66290100	-1.70986300	3.10903000
H	3.40048200	-1.00392000	4.13320100
H	4.92538500	-0.09698400	3.86943000
C	2.88383600	2.47590100	-1.39131500
C	3.96141500	2.23479800	-2.28704400

C	3.98785600	2.94556700	-3.50167500
H	4.81428300	2.77167100	-4.20802100
C	2.97701900	3.85217800	-3.83165700
H	3.01127200	4.39415900	-4.78897500
C	1.91002300	4.05626400	-2.94854400
H	1.10922200	4.75591700	-3.22408700
C	1.83480300	3.37716400	-1.71882000
C	5.06891900	1.22359600	-1.99993100
H	4.85235300	0.74554400	-1.02293200
C	6.45014900	1.89465200	-1.89213000
H	6.75511200	2.33849400	-2.86235500
H	7.22421500	1.15243600	-1.60720200
H	6.46131900	2.70805300	-1.14037600
C	5.09810900	0.10971100	-3.06100900
H	4.10992000	-0.38278800	-3.15371400
H	5.85572800	-0.65746300	-2.79956200
H	5.36497800	0.51294400	-4.05950500
C	0.67937500	3.63317000	-0.75699700
H	0.42889400	2.65688900	-0.28782100
C	1.08136400	4.61392200	0.36087300
H	1.41347500	5.58121900	-0.07116100
H	1.89915300	4.22467600	0.99368600
H	0.21365000	4.81180800	1.02085700
C	-0.58983300	4.14174700	-1.44859800
H	-0.46215900	5.17138000	-1.84463200
H	-1.41834800	4.17486100	-0.71590400
H	-0.89548000	3.46866000	-2.27401700
C	2.70217700	-2.22464000	1.54541900
C	3.51249200	-3.08115500	0.74697500
C	3.46080500	-4.46536500	0.99519200
H	4.08357800	-5.14362000	0.39479100
C	2.62440600	-4.99489200	1.98573100
H	2.60054900	-6.08049300	2.16450200
C	1.80413300	-4.14181800	2.73162600
H	1.13068300	-4.56488700	3.49257700
C	1.81959600	-2.74858100	2.52802800
C	4.43212100	-2.51714300	-0.33259500
H	3.94534400	-1.59692800	-0.72053600
C	5.79665600	-2.09791800	0.24420400
H	6.44850000	-1.69232000	-0.55686000
H	6.31678500	-2.96612700	0.70004400
H	5.69622300	-1.31327000	1.01889800
C	4.60430100	-3.45705200	-1.53224700
H	3.62376500	-3.77432500	-1.93803900
H	5.18649400	-4.36565700	-1.27318600
H	5.15388800	-2.93840800	-2.34231000
C	0.88347800	-1.85849700	3.33860800
H	1.17755200	-0.80766000	3.14913800
C	0.97360200	-2.11449000	4.85238800
H	2.01538100	-2.06312500	5.22905900
H	0.57456100	-3.11376400	5.12289900
H	0.37287300	-1.36286300	5.40493100
C	-0.56640600	-2.01026100	2.85370500
H	-1.24921800	-1.36989800	3.44714100
H	-0.91876400	-3.05625500	2.95678500
H	-0.65683500	-1.71618000	1.79149600
C	-0.00004000	0.00001100	-1.91338700

Cartesian coordinates of the optimized geometry of **1** at PBE0-D3BJ/def2-SVP level of theory:

Ga	-1.66109000	-0.08304500	0.63838200
Ga	1.64991200	0.09709200	-0.45904800
P	0.42013800	0.05036500	1.41163400
P	0.76631800	0.13427300	-2.90049100
O	-1.63138100	-0.13907400	-1.45648100
C	-0.61334300	-0.02173700	-2.09488700

N	-3.07971700	1.28608500	0.87459700
N	-2.90685900	-1.59180500	0.95118700
N	2.90701600	1.61834100	-0.74654400
N	3.03955000	-1.28208700	-0.84502500
C	-4.17808500	1.05078900	1.59536500
C	-4.56032600	-0.21857300	2.04766800
H	-5.45553600	-0.25537100	2.66508400
C	-4.01865400	-1.45489200	1.67698000
C	-5.08930300	2.19205000	1.95049200
H	-5.98575900	1.82857300	2.46563000
H	-5.38901400	2.75787700	1.05828100
H	-4.56851100	2.90642200	2.60229400
C	-4.76432900	-2.67997500	2.12634300
H	-5.69309800	-2.40508800	2.63915300
H	-4.13992800	-3.26951600	2.81158000
H	-4.99911100	-3.34247500	1.28281400
C	-2.91965000	2.60837600	0.34859700
C	-2.30451500	3.61393600	1.12247500
C	-2.28359200	4.91657000	0.61262500
H	-1.81153400	5.70802100	1.19961600
C	-2.85801600	5.22334800	-0.61407600
H	-2.84479000	6.25129700	-0.98454900
C	-3.43096000	4.21352300	-1.37893700
H	-3.86264600	4.45404200	-2.35333400
C	-3.46313300	2.89310400	-0.92418800
C	-1.68343500	3.34442600	2.48037400
H	-1.83182800	2.27876100	2.71278300
C	-2.32752800	4.17420000	3.59337700
H	-3.41631300	4.03154400	3.66121400
H	-2.14699200	5.25055700	3.44441400
H	-1.89399700	3.90122600	4.56779700
C	-0.18037500	3.59944300	2.44368500
H	0.26906200	3.37415000	3.42332700
H	0.04119900	4.65057000	2.20613700
H	0.31123700	2.95773400	1.69995500
C	-4.10948800	1.81919900	-1.78152200
H	-3.84376400	0.84958500	-1.34123900
C	-5.63554700	1.92027800	-1.77490800
H	-6.07553300	1.13212800	-2.40653300
H	-5.97311100	2.89322600	-2.16712600
H	-6.05073900	1.80230500	-0.76372700
C	-3.57256200	1.83011100	-3.21108700
H	-3.96157800	0.96593200	-3.77103100
H	-2.47520000	1.77824800	-3.22847100
H	-3.87715400	2.73740500	-3.75650200
C	-2.61264400	-2.89992200	0.45074100
C	-1.83070300	-3.79919700	1.20325100
C	-1.67051300	-5.09997900	0.71224600
H	-1.06701200	-5.81066700	1.28191600
C	-2.27226200	-5.50650100	-0.47162700
H	-2.14828600	-6.53290200	-0.82561800
C	-3.01701000	-4.59729600	-1.21555700
H	-3.47102600	-4.91479200	-2.15734800
C	-3.18940500	-3.28095200	-0.78243000
C	-1.18163300	-3.42151800	2.52163900
H	-1.42118600	-2.36627700	2.72304700
C	-1.69981100	-4.26652300	3.68767800
H	-2.79242600	-4.21427100	3.80401100
H	-1.24714100	-3.92672800	4.63196600
H	-1.43480200	-5.32805100	3.55898100
C	0.33627200	-3.53415500	2.43272700
H	0.80046000	-3.20022300	3.37327800
H	0.73216000	-2.89988400	1.62856600
H	0.65097500	-4.57268900	2.25168400
C	-4.01304100	-2.31424500	-1.61653500
H	-3.85537500	-1.31084000	-1.19906700
C	-5.51060000	-2.61432100	-1.53271500
H	-5.73537700	-3.62791100	-1.90226900

H	-6.08023900	-1.89877800	-2.14671200
H	-5.88915300	-2.53968400	-0.50348300
C	-3.55307100	-2.27610400	-3.07192300
H	-3.73552300	-3.23327000	-3.58550700
H	-2.48128100	-2.04921200	-3.14515300
H	-4.10348600	-1.49876800	-3.62406600
C	3.94184700	1.50069700	-1.57550000
C	4.37960200	0.27671500	-2.10825100
H	5.20953100	0.33863300	-2.80974700
C	4.05018300	-1.01493500	-1.67004300
C	4.73682800	2.72069200	-1.95034900
H	5.04932500	3.27306000	-1.05255500
H	4.12592200	3.41524400	-2.54378200
H	5.62427800	2.44472700	-2.53143600
C	4.94045600	-2.13460800	-2.13087800
H	5.41638700	-2.62604300	-1.26970500
H	5.72051800	-1.76313700	-2.80528000
H	4.35844300	-2.91209600	-2.64445400
C	2.66654700	2.89613900	-0.15016300
C	3.13034900	3.12684800	1.16257300
C	3.01796400	4.41605300	1.68834200
H	3.38526700	4.61733400	2.69582100
C	2.43219900	5.44410500	0.95849900
H	2.35522000	6.44594400	1.38795000
C	1.91645400	5.18336300	-0.30370700
H	1.42019700	5.98180900	-0.86005400
C	2.02198800	3.91430400	-0.88105800
C	3.75341000	2.01266400	1.98056800
H	3.27450700	1.07723500	1.65073700
C	5.25440700	1.88783400	1.71671000
H	5.69161900	1.08926300	2.33610900
H	5.77478500	2.82781900	1.96181500
H	5.46830000	1.64387000	0.66608900
C	3.46544700	2.14706300	3.47143700
H	2.38499700	2.23477800	3.65462600
H	3.97035500	3.01856200	3.91721100
H	3.82592100	1.25761100	4.00722000
C	1.39972700	3.65974200	-2.23786000
H	1.76645600	2.68803000	-2.60154900
C	1.75802700	4.71474400	-3.28248200
H	1.36149800	4.42197000	-4.26684400
H	2.84586700	4.85149100	-3.38352700
H	1.32398500	5.69659800	-3.03677700
C	-0.11404800	3.54311400	-2.08868800
H	-0.38694600	2.79011100	-1.33531500
H	-0.58205800	3.25776700	-3.04302100
H	-0.54921400	4.49907100	-1.76459200
C	2.94810000	-2.60287800	-0.30238100
C	3.57124200	-2.87234000	0.93336000
C	3.54465500	-4.18029500	1.42449500
H	4.03036900	-4.40133500	2.37821000
C	2.90925500	-5.19923300	0.72652100
H	2.89688900	-6.21563400	1.12754700
C	2.27861300	-4.91501200	-0.47815700
H	1.76613900	-5.71274000	-1.02085800
C	2.29249700	-3.62586400	-1.01795100
C	4.28299300	-1.79691000	1.72880800
H	4.17232300	-0.85243200	1.17683800
C	5.77973500	-2.08548400	1.84651100
H	6.29128200	-1.27134400	2.38320400
H	6.25352700	-2.18457100	0.85801600
H	5.96779000	-3.01846100	2.40128000
C	3.63773000	-1.60842900	3.10019800
H	2.58634300	-1.29882000	2.99879100
H	4.17247500	-0.83652100	3.67397700
H	3.66849400	-2.53802000	3.69047700
C	1.59654400	-3.36397700	-2.33598000
H	1.87105000	-2.35017000	-2.66359800

C	2.01278200	-4.33723300	-3.43744300
H	1.68473400	-5.36570500	-3.21930400
H	3.10422000	-4.36232900	-3.58022600
H	1.55402200	-4.04438100	-4.39448300
C	0.08509400	-3.38178900	-2.14216000
H	-0.26357200	-4.38416000	-1.85584800
H	-0.43098100	-3.09033400	-3.06927100
H	-0.22631900	-2.69241800	-1.34465600

Cartesian coordinates of the optimized geometry of **2** at PBE0-D3BJ/def2-SVP level of theory:

Ga	-1.55872500	-0.30985900	-0.02615400
Ga	1.55779500	0.31090500	-0.05677800
P	-1.50640500	0.78642700	-2.13310100
P	1.42558900	-0.73330600	-2.18763500
O	0.00663600	0.00520100	0.82702400
N	-2.23868500	-2.16434000	-0.20939100
N	-2.89242800	0.26185900	1.30290200
N	2.23602100	2.16249500	-0.26784000
N	2.91706100	-0.24454300	1.26184900
C	-3.04659000	-2.69924000	0.70203000
C	-3.54473300	-2.00632000	1.81747100
H	-4.13059200	-2.60349200	2.51517700
C	-3.54939800	-0.62505800	2.04946400
C	-3.53242800	-4.11933000	0.56661600
H	-4.62478300	-4.12581400	0.44365700
H	-3.31380400	-4.66582000	1.49559200
H	-3.07952200	-4.64922600	-0.27763400
C	-4.37377400	-0.13937700	3.20918700
H	-5.01206700	0.70736500	2.92222500
H	-3.71530000	0.22459600	4.01026800
H	-4.99862100	-0.94583600	3.60986800
C	-2.03662400	-2.84425400	-1.45417400
C	-3.01185800	-2.71101600	-2.46619900
C	-2.81307000	-3.39047200	-3.67156700
H	-3.55895700	-3.30068300	-4.46485500
C	-1.68525900	-4.17232000	-3.87704100
H	-1.54528000	-4.69594600	-4.82571600
C	-0.72655400	-4.27804600	-2.87609100
H	0.16333600	-4.88269800	-3.05310900
C	-0.87314500	-3.61826200	-1.65283900
C	-4.27542600	-1.88643600	-2.28867200
H	-4.17425800	-1.31763300	-1.35177600
C	-5.51442000	-2.77632400	-2.16419300
H	-6.41043800	-2.16717300	-1.96555200
H	-5.41793800	-3.51216400	-1.35521200
H	-5.68958300	-3.33498700	-3.09751400
C	-4.47339500	-0.87964300	-3.42047200
H	-4.64880400	-1.38286800	-4.38410300
H	-3.59642600	-0.22596500	-3.53203000
H	-5.35237800	-0.24777200	-3.21856200
C	0.18331100	-3.76858800	-0.57197400
H	0.27683400	-2.78724800	-0.07477000
C	-0.21165700	-4.79509300	0.49244800
H	-1.08702800	-4.48654300	1.07426400
H	0.61886800	-4.93267200	1.20061000
H	-0.43164300	-5.76996700	0.02822800
C	1.55451300	-4.13816100	-1.12470400
H	1.83993600	-3.48764000	-1.96315300
H	1.58374500	-5.18431900	-1.47037900
H	2.31136700	-4.03463100	-0.33612600
C	-3.09785500	1.65211500	1.54889800
C	-4.06933400	2.32543200	0.77395900
C	-4.35944800	3.65581800	1.08545100
H	-5.11714700	4.18982400	0.50932800
C	-3.68194800	4.31862300	2.10434500

H	-3.92020700	5.36020400	2.33237200
C	-2.68433000	3.66010500	2.81183800
H	-2.13662700	4.19382100	3.59268200
C	-2.36965100	2.32056500	2.55555800
C	-4.78353600	1.61998700	-0.36629400
H	-4.08530000	0.86028200	-0.75175900
C	-6.03668100	0.87789300	0.10111800
H	-6.75254700	1.57110900	0.57158900
H	-5.80139400	0.08536300	0.82482700
H	-6.54061700	0.40140500	-0.75473700
C	-5.11008500	2.55319100	-1.52786300
H	-4.22190900	3.11254600	-1.85479800
H	-5.89803200	3.27805600	-1.26879000
H	-5.47626700	1.97078100	-2.38607700
C	-1.27455000	1.64669200	3.36355300
H	-1.22209100	0.59805000	3.03829400
C	-1.54697200	1.69155100	4.86820000
H	-1.51055800	2.72294500	5.25341900
H	-0.78183000	1.11412200	5.41013600
H	-2.53086400	1.27950000	5.13693300
C	0.09024100	2.26381200	3.06498500
H	0.32577000	2.17821000	1.99836500
H	0.87976200	1.73586500	3.62156500
H	0.12653900	3.32548900	3.35684000
C	3.04680300	2.71536000	0.63097900
C	3.56658300	2.03409600	1.74341100
H	4.16290400	2.63891700	2.42570000
C	3.59543700	0.65309000	1.97579300
C	3.51567000	4.14102500	0.47902600
H	4.59641000	4.16053300	0.27788800
H	3.35803400	4.67578700	1.42656500
H	3.00586500	4.67762000	-0.32729100
C	4.52228200	0.17997700	3.06108900
H	5.44403800	-0.21820500	2.61055600
H	4.08670800	-0.63646900	3.64889800
H	4.79563200	1.00801700	3.72584200
C	2.01484100	2.84883800	-1.50520800
C	2.94830500	2.67286200	-2.54923300
C	2.77871100	3.41943700	-3.71874000
H	3.49491900	3.30635100	-4.53508900
C	1.71034800	4.29388900	-3.86311100
H	1.59559200	4.87274200	-4.78265800
C	0.76960400	4.40944700	-2.84666900
H	-0.08549400	5.07166400	-2.98547200
C	0.89068400	3.68741500	-1.65574100
C	4.11301400	1.70215300	-2.44495900
H	3.86996500	0.99409300	-1.63739400
C	5.42520700	2.39287300	-2.07184900
H	5.69701100	3.15346700	-2.82137000
H	6.24523900	1.65859300	-2.02365400
H	5.36760000	2.88949200	-1.09489300
C	4.29228400	0.88610400	-3.72333100
H	3.35020100	0.40295100	-4.02083900
H	5.04546800	0.09908600	-3.56856700
H	4.64248500	1.50848900	-4.56152500
C	-0.16260600	3.82225800	-0.56918700
H	-0.27535900	2.82401500	-0.11162100
C	0.23417600	4.79868800	0.54024700
H	0.48529700	5.78624700	0.12057900
H	1.08839600	4.44614300	1.12861000
H	-0.60850100	4.92729500	1.23603700
C	-1.52347700	4.22992000	-1.12101300
H	-1.53254100	5.28352200	-1.44417500
H	-2.28441900	4.12376700	-0.33743400
H	-1.81609900	3.59986500	-1.97321200
C	3.12466500	-1.63108600	1.53402300
C	4.17240900	-2.31012300	0.87559300
C	4.40334800	-3.65089600	1.19701000

H	5.21277600	-4.18880100	0.69707400
C	3.62023500	-4.31012100	2.13690300
H	3.81637600	-5.35768700	2.37729500
C	2.58153400	-3.63166700	2.76444600
H	1.96781800	-4.15261100	3.50365700
C	2.30913800	-2.28916400	2.48245800
C	5.05155400	-1.63182300	-0.15945900
H	4.73113500	-0.58149800	-0.22855800
C	6.52649700	-1.63981400	0.24434400
H	7.12624900	-1.06772100	-0.48069200
H	6.93069200	-2.66405200	0.27255500
H	6.68545100	-1.19548200	1.23802400
C	4.87069200	-2.26153400	-1.54028900
H	3.83086100	-2.18315000	-1.88594000
H	5.14280800	-3.32886000	-1.53169600
H	5.51246700	-1.75961800	-2.28039600
C	1.19801400	-1.57890000	3.23579500
H	1.07117000	-0.58869600	2.77919200
C	1.53723500	-1.40479300	4.71724200
H	2.46085400	-0.82845800	4.87169500
H	1.66325100	-2.37714100	5.22070000
H	0.72415700	-0.86847600	5.23158600
C	-0.14202800	-2.29008000	3.07826900
H	-0.93593200	-1.72186200	3.58630100
H	-0.13228400	-3.30331700	3.51146800
H	-0.41910100	-2.36439400	2.02046000
C	-0.04104500	0.02643100	-2.19499100

Cartesian coordinates of the optimized geometry of **6** at PBE0-D3BJ/def2-SVP level of theory:

Ga	1.39261200	-0.58185200	-0.38827300
Ga	-1.62785500	0.19308900	-0.31691000
P	0.95165800	1.14101500	1.16919700
P	-0.66218500	0.22710900	3.08768100
Si	1.55888000	2.26068200	4.06945400
C	-0.73243600	0.47384300	1.40882700
C	1.13663100	0.76265800	2.99828100
H	1.81540000	-0.07712100	3.21635300
C	0.24188700	3.57848700	3.82953400
H	0.54127600	4.53079500	4.29555900
H	-0.71889200	3.27158300	4.26983100
H	0.07107600	3.76380300	2.75714300
C	3.23301500	2.98381500	3.59220300
H	3.42764800	3.90508400	4.16478900
H	3.25142600	3.24239000	2.52172000
H	4.05787100	2.28316700	3.78883400
C	1.60016200	1.68236200	5.86292500
H	1.82215600	2.51144700	6.55371200
H	2.36643100	0.90342400	6.00511300
H	0.62723500	1.25199200	6.14976700
O	-0.22041500	-0.53338800	-1.22824400
N	2.61829700	-0.56188500	-1.95027200
N	2.15875000	-2.25080800	0.38146600
N	-3.16265900	-0.81714000	-1.04580700
N	-2.38423000	1.96958200	-0.79448900
C	3.24365400	-1.65902700	-2.36833700
C	3.25771900	-2.87836100	-1.67192900
H	3.79090300	-3.69428700	-2.15788500
C	2.87125000	-3.11035800	-0.34639800
C	4.06852300	-1.60550600	-3.62512100
H	4.35742700	-2.61343900	-3.94513500
H	4.98410200	-1.02385100	-3.43748600
H	3.54490400	-1.09596600	-4.44246800
C	3.38997600	-4.38066200	0.27329100
H	3.33745800	-5.19720000	-0.46006800
H	2.84429100	-4.66576600	1.17922800

H	4.44896300	-4.25339000	0.54477900
C	2.74397200	0.61832600	-2.74712200
C	1.85409300	0.83624300	-3.82415100
C	2.08138500	1.94271100	-4.64889400
H	1.41159900	2.11792600	-5.49459800
C	3.14311200	2.81200300	-4.42527800
H	3.30562800	3.66356400	-5.09016300
C	3.98546700	2.60159500	-3.34058900
H	4.80473600	3.29953100	-3.15054300
C	3.79896900	1.51530600	-2.48049100
C	0.69978500	-0.09961800	-4.13950000
H	0.63726600	-0.83098200	-3.32349100
C	0.90221100	-0.83917900	-5.46296300
H	0.05473900	-1.51707000	-5.65083900
H	1.81744700	-1.44875200	-5.46900200
H	0.96106000	-0.14088700	-6.31369800
C	-0.63599800	0.63942500	-4.14289900
H	-1.46214500	-0.07014700	-4.30329800
H	-0.69046500	1.39918900	-4.93932800
H	-0.80860600	1.12866000	-3.17718000
C	2.15734100	-2.39782200	1.80360400
C	1.00076900	-2.86100100	2.46631800
C	1.02697900	-2.94059300	3.86179400
H	0.13808400	-3.28023800	4.39340900
C	2.15981000	-2.58969100	4.58718100
H	2.15271200	-2.65246100	5.67774400
C	3.29747500	-2.15614400	3.91964100
H	4.18607600	-1.87911700	4.49206100
C	3.32085700	-2.05233900	2.52501000
C	-0.21882700	-3.31925500	1.69153200
H	-0.36419100	-2.60931200	0.85820500
C	-0.00190800	-4.71111300	1.09178400
H	-0.92189000	-5.04973200	0.59445500
H	0.24678700	-5.43546800	1.88416200
H	0.80040600	-4.72915000	0.34486700
C	-1.49359300	-3.32687500	2.52205300
H	-2.35779400	-3.50084600	1.86934500
H	-1.64101000	-2.37193200	3.04570700
H	-1.48327800	-4.13486400	3.27160800
C	4.57607000	-1.52665700	1.85292800
H	4.45503400	-1.63008400	0.76477100
C	5.82498400	-2.31108400	2.25470700
H	6.69413800	-1.97542400	1.66794800
H	5.70129600	-3.39237200	2.09452400
H	6.07135600	-2.16291800	3.31773300
C	4.75692000	-0.04005400	2.14730900
H	5.64951100	0.35382500	1.63758900
H	4.88260800	0.13391000	3.22737600
H	3.88752300	0.54447600	1.81600300
C	-3.97460800	-0.28865600	-1.96351900
C	-3.96778000	1.05572900	-2.35828000
H	-4.67990100	1.32291600	-3.13716900
C	-3.32698400	2.12871000	-1.71933500
C	-5.04917600	-1.14736400	-2.57273800
H	-5.47027500	-0.66620200	-3.46338500
H	-5.86107900	-1.29020000	-1.84227000
H	-4.68614400	-2.14892300	-2.83173300
C	-3.83533700	3.50517900	-2.05738500
H	-4.34134000	3.49595600	-3.03102600
H	-3.03890200	4.25681900	-2.06141900
H	-4.56257700	3.82802600	-1.29713800
C	-3.37481500	-2.18445000	-0.68919900
C	-2.69633200	-3.20796400	-1.38723200
C	-2.99124800	-4.53444400	-1.05378500
H	-2.48531500	-5.34026100	-1.59163100
C	-3.92141900	-4.84747400	-0.06931400
H	-4.13656800	-5.89146100	0.17049200
C	-4.57583600	-3.82620600	0.61033400

H	-5.30231800	-4.07501600	1.38735500
C	-4.31936900	-2.48461900	0.31513500
C	-1.72440800	-2.91922700	-2.51790300
H	-1.54766400	-1.83588700	-2.53094200
C	-2.29088100	-3.34878900	-3.87246400
H	-1.57645100	-3.10788700	-4.67533500
H	-3.23682300	-2.84140100	-4.11095800
H	-2.47653600	-4.43469900	-3.90851300
C	-0.36159900	-3.56296000	-2.28310500
H	0.33162300	-3.28788700	-3.09252300
H	-0.41681100	-4.66330700	-2.25279000
H	0.07793300	-3.20852000	-1.34373100
C	-2.05161700	3.11903600	-0.01020200
C	-0.99806500	3.96212800	-0.41018100
C	-0.83479800	5.17525700	0.26672600
H	-0.03659900	5.85422400	-0.03746200
C	-1.66326400	5.52593000	1.32519300
H	-1.52650400	6.48265600	1.83466000
C	-2.62714200	4.63052800	1.77303600
H	-3.22413900	4.88335800	2.65063900
C	-2.82795400	3.40427800	1.13363300
C	-0.04590700	3.57535100	-1.52674500
H	-0.01481800	2.47275700	-1.55262400
C	-0.49515700	4.05817700	-2.90702700
H	0.28470200	3.83495800	-3.65001000
H	-0.66214700	5.14782800	-2.90432000
H	-1.41734700	3.56917800	-3.24634600
C	1.37146600	4.05983500	-1.24602300
H	2.07146000	3.58980400	-1.94827200
H	1.67633300	3.79639400	-0.22370600
H	1.46517700	5.15054900	-1.37168600
C	-3.82578900	2.40259500	1.69407900
H	-3.45961300	1.40565800	1.40150500
C	-5.23754200	2.55694300	1.12571800
H	-5.92787100	1.86002900	1.62729800
H	-5.27992600	2.33623400	0.05038100
H	-5.61950100	3.57828800	1.28584400
C	-3.85889500	2.42969200	3.21958500
H	-4.47781800	1.60434400	3.60003300
H	-4.29388600	3.36361100	3.60839000
H	-2.84861800	2.30982800	3.63712000
C	4.72067500	1.34653100	-1.28706600
H	4.42808700	0.41868500	-0.77307500
C	6.18494500	1.20204200	-1.70009600
C	4.53697100	2.49985600	-0.30039800
H	5.22190400	2.39992800	0.55432700
H	3.50817100	2.52427900	0.08646600
H	4.73840600	3.47118600	-0.77885200
H	6.33127900	0.37123800	-2.40673100
H	6.56199700	2.11779300	-2.18219600
H	6.81675200	1.00987400	-0.81862500
C	-5.04041300	-1.38983700	1.07854100
H	-4.88443400	-0.44806500	0.53186800
C	-4.43526300	-1.21163500	2.47052900
H	-4.49368000	-2.14607600	3.05062800
H	-3.37649500	-0.91881200	2.41982500
H	-4.97347400	-0.43254700	3.03049700
C	-6.54752700	-1.62336800	1.16183300
H	-7.04370700	-0.74977800	1.61230900
H	-6.99067100	-1.79013700	0.16803700
H	-6.79497700	-2.49565500	1.78688300

Cartesian coordinates of the optimized geometry of $TMSCHN_2$ at PBE0-D3BJ/def2-SVP level of theory:

N	-2.06430000	-0.41309400	0.00005900
C	-0.90617800	-0.98973900	-0.00007600

N	-3.05810500	0.13592400	0.00010900
Si	0.67372800	0.01462700	0.00008000
C	0.17735700	1.82653000	0.00109200
H	-0.41788600	2.08108200	0.89211500
H	1.07249100	2.46831100	0.00115000
H	-0.41804300	2.08153900	-0.88971400
C	1.68062300	-0.40612700	1.53173600
H	1.92238200	-1.48040600	1.56212500
H	2.63083700	0.15200400	1.54312100
H	1.12493200	-0.15905200	2.44935300
C	1.67927800	-0.40448400	-1.53294300
H	1.12433200	-0.15242900	-2.44966700
H	2.63150100	0.15023800	-1.54270200
H	1.91708800	-1.47953400	-1.56691300
H	-0.94947900	-2.08343100	-0.00001600

Cartesian coordinates of the optimized geometry of N_2 at PBE0-D3BJ/def2-SVP level of theory:

N	0.00000000	0.00000000	0.54887900
N	0.00000000	0.00000000	-0.54887900

Cartesian coordinates of the optimized geometry of **S1** at PBE0-D3BJ/def2-SVP level of theory:

Ga	-1.76190500	0.25604900	-0.50868500
Ga	1.56644100	0.21090500	-0.31922500
P	-0.04212500	0.81067300	-1.77112400
P	1.31103100	-0.53641500	2.12033800
O	-1.27035800	0.49163600	1.67809900
C	-0.17535300	0.06381400	1.90568700
N	-2.52187600	-1.48774600	-0.11197500
N	-3.40637400	1.26301700	-0.39080900
N	2.79485700	-1.24582200	-0.78255700
N	2.99350100	1.50819900	0.04047400
C	-3.74076800	-1.63289600	0.39284900
C	-4.66178100	-0.57990200	0.53093500
H	-5.62429000	-0.84562800	0.96520400
C	-4.51837800	0.75996300	0.13497900
C	-4.18208000	-2.99861700	0.83581300
H	-5.18808400	-2.97000300	1.26987100
H	-3.48655500	-3.40866900	1.58442500
H	-4.19015600	-3.70195500	-0.01186000
C	-5.70323300	1.66505600	0.31423400
H	-6.54536700	1.13236300	0.77050300
H	-6.03267200	2.07417300	-0.65374600
H	-5.44446400	2.52502500	0.95150500
C	4.01425400	-1.30278300	-0.26331100
C	4.62825700	-0.22383000	0.39701300
H	5.61854100	-0.41681600	0.80727800
C	4.18649200	1.11294800	0.45632500
C	4.80674700	-2.57217300	-0.40202300
H	5.03237800	-2.77731400	-1.46072400
H	4.23732500	-3.43484500	-0.02271200
H	5.75584200	-2.50847600	0.14294600
C	5.14042500	2.13344900	1.00901600
H	5.39862900	2.88187300	0.24320900
H	6.06755700	1.66425600	1.35824000
H	4.68228300	2.68007100	1.84812500
C	-3.34019600	2.65083400	-0.80383300
H	-2.34037000	2.84323100	-1.22018100
H	-4.08630200	2.88826000	-1.58039500
H	-3.49456800	3.34206800	0.04210000
C	-1.63268600	-2.62253100	-0.24770500
H	-2.10330900	-3.46156000	-0.78527500
H	-0.75166400	-2.30285000	-0.82238200

H	-1.27820400	-2.98956400	0.73117000
C	2.61780300	2.90427300	0.06231200
H	1.63614300	3.00601700	-0.42463300
H	2.53169500	3.29451400	1.09189500
H	3.33708100	3.53704600	-0.48361700
C	2.25345000	-2.34624100	-1.54633500
H	1.98982500	-3.20967000	-0.90843100
H	1.33836800	-1.99619300	-2.04857700
H	2.95054300	-2.69960200	-2.32340000

Cartesian coordinates of the optimized geometry of **S2** at PBE0-D3BJ/def2-SVP level of theory:

Ga	1.55863800	-0.23105700	-0.23811300
Ga	-1.55859700	0.23103500	-0.23805600
P	1.24812400	-1.08689900	-2.44515800
P	-1.24831900	1.08670200	-2.44522100
O	0.00006200	0.00017500	0.63824500
N	2.71751700	1.32425800	-0.15797600
N	2.68439200	-1.45362900	0.76362300
N	-2.71741600	-1.32430500	-0.15770400
N	-2.68435100	1.45366100	0.76362800
C	3.85862900	1.32839700	0.51556700
C	4.37131500	0.21575900	1.20920400
H	5.31864500	0.37488500	1.72191400
C	3.82728000	-1.07571900	1.32103200
C	4.67276900	2.59121600	0.54484800
H	4.97126600	2.88673300	-0.47330600
H	5.57825400	2.46696800	1.14960600
H	4.08625800	3.42625600	0.95878600
C	4.60190600	-2.08192300	2.12500000
H	4.88432400	-2.94627500	1.50348100
H	3.99299400	-2.47175600	2.95569900
H	5.51518500	-1.64064400	2.54005900
C	-3.85868000	-1.32830800	0.51559100
C	-4.37145900	-0.21558600	1.20901300
H	-5.31890600	-0.37461500	1.72153400
C	-3.82733300	1.07585200	1.32091300
C	-4.67285300	-2.59110600	0.54483900
H	-4.97068200	-2.88704800	-0.47338500
H	-5.57872200	-2.46662400	1.14897300
H	-4.08660300	-3.42597300	0.95950500
C	-4.60194900	2.08209100	2.12484100
H	-4.88441500	2.94641500	1.50330100
H	-3.99300000	2.47197500	2.95548700
H	-5.51521100	1.64082200	2.53994800
C	-0.00010200	-0.00010000	-2.45555500
C	2.27031100	2.50150700	-0.87435600
H	1.33203600	2.27593700	-1.40045200
H	2.08003200	3.35114700	-0.19579000
H	3.00171400	2.82904500	-1.63195100
C	2.18470700	-2.80098200	0.92733200
H	2.90305300	-3.56087000	0.57606900
H	1.93329600	-3.02648600	1.97786000
H	1.26562500	-2.91823300	0.33646000
C	-2.18453800	2.80095700	0.92742600
H	-2.90331100	3.56099900	0.57740700
H	-1.93190700	3.02590900	1.97778900
H	-1.26614400	2.91854200	0.33554700
C	-2.27009500	-2.50170500	-0.87376000
H	-1.33141400	-2.27644400	-1.39926500
H	-2.08055500	-3.35140700	-0.19507200
H	-3.00109500	-2.82901300	-1.63185300

Cartesian coordinates of the optimized geometry of Int-**1** at PBE0-D3BJ/def2-SVP level of theory:

Ga	2.04491800	0.60548500	0.35680500
Ga	-2.16856200	-0.71794800	0.62925200
P	0.14841100	-0.61536800	-0.06477500
P	-1.22086600	-0.62474600	2.73179600
O	1.28963100	0.34227300	2.13147500
N	2.85453100	2.32342500	0.03378300
N	3.71379500	-0.41912500	0.50682200
N	-2.87147000	-2.28442700	-0.35225000
N	-3.70802900	0.44303000	0.19831300
C	4.14958300	2.55614500	0.29538600
C	5.07712500	1.56406300	0.61208200
H	6.09456800	1.90701500	0.79569600
C	4.89266900	0.16397800	0.64316000
C	4.64525300	3.97124700	0.21075300
H	5.67573600	4.04750400	0.57673500
H	3.99778700	4.64087700	0.79529200
H	4.61082800	4.33598800	-0.82683400
C	6.12985700	-0.66154100	0.85297000
H	5.98057500	-1.71030700	0.57010100
H	6.40742800	-0.63287700	1.91789200
H	6.96881100	-0.23934200	0.28370000
C	2.05174200	3.37191400	-0.51084700
C	2.16082700	3.66100700	-1.88943400
C	1.38905100	4.70254800	-2.40661000
H	1.45800400	4.94788900	-3.46836600
C	0.51970400	5.42351600	-1.59219500
H	-0.07943900	6.23301400	-2.01660500
C	0.40394500	5.10453100	-0.24585700
H	-0.29436600	5.65892800	0.38506900
C	1.16929200	4.08071800	0.32365900
C	3.04412700	2.82254000	-2.79629000
H	3.82097900	2.35660100	-2.17157300
C	3.75996500	3.63706400	-3.86932700
H	4.46914900	3.00055200	-4.42028000
H	4.32352200	4.47691800	-3.43445600
H	3.05790800	4.05233400	-4.60904300
C	2.23291500	1.68400500	-3.41916000
H	1.46346900	2.08074000	-4.09952400
H	1.71328600	1.08992700	-2.65176700
H	2.88400800	1.00760200	-3.99487800
C	1.03078300	3.75675400	1.79635000
H	1.75596600	2.96575600	2.03565200
C	1.36182400	4.96025700	2.67790900
H	2.37362300	5.34770400	2.48012000
H	1.30811800	4.67964300	3.74098300
H	0.65119900	5.78678500	2.51796000
C	-0.35733400	3.20183100	2.10499800
H	-0.61345900	2.36193500	1.44320700
H	-1.13538100	3.96959900	1.97991700
H	-0.40149800	2.82563000	3.13719000
C	3.55981300	-1.84047800	0.53171800
C	3.35250500	-2.49898800	-0.69928600
C	3.16292400	-3.88075900	-0.68874700
H	2.99178500	-4.40745700	-1.62851300
C	3.16868600	-4.59362000	0.50533000
H	3.01571700	-5.67547300	0.49693000
C	3.34466700	-3.92529400	1.70853800
H	3.32009100	-4.48748600	2.64500200
C	3.53038100	-2.53910800	1.75476900
C	3.35450900	-1.72298200	-2.00107600
H	2.97207500	-0.71490700	-1.77207700
C	4.77186300	-1.55089100	-2.54829800
H	5.22279900	-2.53052500	-2.77380400
H	5.42888900	-1.03726900	-1.83185100
H	4.76184300	-0.95962500	-3.47794300
C	2.42184900	-2.31558700	-3.04823900

H	1.42423600	-2.49785700	-2.62483900
H	2.80352600	-3.26817100	-3.44930100
H	2.31812300	-1.62453000	-3.89886700
C	3.68009700	-1.86160100	3.10425600
H	3.77333600	-0.77933500	2.93010300
C	4.93210700	-2.34724400	3.83925500
H	4.84062100	-3.40998900	4.11463200
H	5.07689600	-1.77829900	4.77090700
H	5.84244100	-2.24623000	3.23092600
C	2.44217300	-2.06893600	3.97743300
H	1.53330600	-1.70005700	3.48667600
H	2.55351600	-1.52356700	4.92739000
H	2.29758400	-3.13399700	4.21931000
C	-4.17815400	-2.45809900	-0.53226900
C	-5.12692700	-1.44381500	-0.33637800
H	-6.16422200	-1.72350900	-0.51315000
C	-4.89325800	-0.07455900	-0.12076400
C	-4.67798500	-3.78749300	-1.02231900
H	-4.37666700	-3.95590300	-2.06595000
H	-5.77134900	-3.84266200	-0.95930500
H	-4.23313500	-4.60503000	-0.43826000
C	-6.07920500	0.83725300	-0.26919000
H	-5.81126100	1.76245600	-0.79672800
H	-6.45079700	1.13573500	0.72172000
H	-6.89220300	0.33305600	-0.80452600
C	-1.96264700	-3.23541300	-0.90655800
C	-1.67266600	-3.16165200	-2.28739800
C	-0.81449100	-4.11556100	-2.83853600
H	-0.58588300	-4.07575600	-3.90680000
C	-0.23342500	-5.10213100	-2.04962800
H	0.43628300	-5.84004600	-2.49819400
C	-0.49273900	-5.13144600	-0.68581800
H	-0.01324100	-5.88905900	-0.06165600
C	-1.35119900	-4.20397800	-0.08760800
C	-2.22217100	-2.05822800	-3.17499300
H	-2.87153100	-1.42103200	-2.55696400
C	-3.06675300	-2.59672900	-4.32970500
H	-2.46470100	-3.21495100	-5.01441700
H	-3.48935800	-1.76671300	-4.91742200
H	-3.90454200	-3.21511500	-3.97537400
C	-1.09068500	-1.17689700	-3.70430900
H	-0.50161700	-0.74893300	-2.87995800
H	-1.49487200	-0.34843800	-4.30593500
H	-0.40373600	-1.74970400	-4.34638500
C	-1.58201700	-4.26094300	1.40840100
H	-2.17764000	-3.37863600	1.68677600
C	-2.36928500	-5.50614000	1.81643600
H	-1.81968300	-6.42727500	1.56328300
H	-3.34871100	-5.55667300	1.31639700
H	-2.54687000	-5.51075600	2.90319100
C	-0.26184500	-4.16306000	2.16849100
H	0.35621200	-5.06407500	2.02544200
H	-0.44790900	-4.04316700	3.24635400
H	0.31997600	-3.29443100	1.83050400
C	-3.56403800	1.86071700	0.30422200
C	-2.99349000	2.54953900	-0.78801400
C	-2.90684500	3.94189700	-0.72579600
H	-2.47163300	4.48880600	-1.56325800
C	-3.36363200	4.63959700	0.38659900
H	-3.29800800	5.73016900	0.41514400
C	-3.88374500	3.94398100	1.46980400
H	-4.21394500	4.49448300	2.35400900
C	-3.98600500	2.54875400	1.46153300
C	-2.53193100	1.80581800	-2.02617200
H	-2.24891800	0.79094100	-1.70648200
C	-3.66511800	1.66724000	-3.04408400
H	-3.31745700	1.13329600	-3.94246100
H	-4.03199300	2.65713600	-3.36018000

H	-4.51734900	1.10544900	-2.63542000
C	-1.29290400	2.42755000	-2.65345000
H	-0.48921800	2.54060600	-1.91210500
H	-1.49405300	3.41864000	-3.08859100
H	-0.91919000	1.78721700	-3.46486000
C	-4.52231300	1.84907600	2.69734100
H	-4.60697300	0.77556500	2.46681700
C	-5.90411100	2.36375900	3.10724300
H	-6.63161300	2.33600600	2.28222900
H	-5.85308000	3.40593500	3.45963200
H	-6.30597200	1.75992200	3.93536900
C	-3.54428900	1.99034000	3.86463100
H	-3.92806800	1.46407900	4.75279900
H	-3.40265900	3.04950200	4.13388400
H	-2.56572900	1.55454400	3.61471900
C	0.17874800	-0.23075800	1.73236800

Cartesian coordinates of the optimized geometry of Int-**2** at PBE0-D3BJ/def2-SVP level of theory:

Ga	2.04648900	-0.14686100	-0.31914800
Ga	-2.05755200	0.14196600	-0.32445000
P	1.50413000	-0.54795700	-2.47575200
P	-1.51485000	0.53240200	-2.48233000
O	-0.00555800	0.00123500	-0.31496900
N	3.03276300	1.56430600	-0.06669400
N	2.75234100	-0.98959900	1.30202700
N	-3.02836200	-1.57729700	-0.05726000
N	-2.75883200	0.99063900	1.29356800
C	3.76257400	1.85285100	0.99927400
C	3.91053300	0.98366900	2.09744400
H	4.52051000	1.36224300	2.91668300
C	3.52926000	-0.35492100	2.19282000
C	4.56846500	3.12143300	1.06101900
H	5.63430100	2.88436700	0.92215700
H	4.46491200	3.57748700	2.05587000
H	4.27844300	3.84346300	0.29049300
C	4.06351400	-1.12213000	3.37171000
H	4.50718800	-2.07598400	3.05331000
H	3.25822600	-1.37365400	4.07634800
H	4.81916100	-0.53329000	3.90428300
C	3.19675600	2.33739700	-1.26342000
C	4.29677000	2.06079200	-2.10076500
C	4.44759300	2.81816800	-3.26528700
H	5.29365400	2.61772500	-3.92740300
C	3.53191400	3.80526400	-3.60413300
H	3.66287900	4.38359400	-4.52172600
C	2.43576000	4.04037700	-2.78255100
H	1.70571000	4.80051800	-3.06515200
C	2.24149600	3.31291400	-1.60614700
C	5.29176200	0.95076300	-1.81256100
H	4.99282600	0.45896500	-0.87394300
C	6.71104700	1.48454400	-1.61570400
H	7.39737900	0.66858100	-1.33930900
H	6.75720300	2.25027500	-0.82773600
H	7.09555500	1.94200100	-2.54094300
C	5.26109200	-0.10917900	-2.91433800
H	5.59863100	0.30784400	-3.87598400
H	4.24410500	-0.50062800	-3.06356500
H	5.93102900	-0.94663200	-2.66416200
C	1.05438400	3.60431000	-0.71218000
H	0.82970000	2.67897500	-0.16032600
C	1.39639400	4.69186800	0.30847700
H	2.22314200	4.39916500	0.97031600
H	0.52143400	4.91188700	0.93702200
H	1.69180200	5.62177100	-0.20409000
C	-0.20340600	3.98108500	-1.48501100

H	-0.44619400	3.22228700	-2.24306600
H	-0.10958700	4.96529200	-1.97212200
H	-1.05369100	4.04291400	-0.79165000
C	2.55491900	-2.39729400	1.45610300
C	3.30485000	-3.28006500	0.64822400
C	3.15547200	-4.65421600	0.85655800
H	3.73004600	-5.35317800	0.24665900
C	2.28627400	-5.14648800	1.82281400
H	2.18644500	-6.22411300	1.97153800
C	1.53203200	-4.26403000	2.58666500
H	0.83676100	-4.65698500	3.33173600
C	1.64403200	-2.88121300	2.41812300
C	4.28372500	-2.76680000	-0.39209000
H	3.91182100	-1.78900300	-0.73767800
C	5.66814600	-2.53598100	0.21528800
H	6.07878700	-3.47297900	0.62468100
H	5.64005000	-1.79460800	1.02711700
H	6.36878300	-2.16418000	-0.54860600
C	4.35620100	-3.65051800	-1.63208000
H	3.35552100	-3.81603200	-2.05704900
H	4.81503500	-4.62941200	-1.42141600
H	4.97050400	-3.16356800	-2.40325300
C	0.77835000	-1.95031500	3.24627600
H	1.20911600	-0.94219100	3.17218200
C	0.73156600	-2.33039500	4.72505500
H	0.18194000	-3.27050100	4.88793200
H	0.21139700	-1.54906800	5.30085500
H	1.73696900	-2.45825800	5.15517400
C	-0.63075200	-1.86916600	2.66664500
H	-0.61002400	-1.47450400	1.64223700
H	-1.26914200	-1.21349100	3.27648000
H	-1.10669500	-2.86057600	2.63826300
C	-3.75613300	-1.85970000	1.01215700
C	-3.91434000	-0.97929600	2.10021800
H	-4.52733100	-1.35218200	2.91976600
C	-3.54322400	0.36330200	2.18219800
C	-4.55390100	-3.13260900	1.08277200
H	-5.61411600	-2.90981200	0.88743700
H	-4.49117400	-3.55558200	2.09524400
H	-4.22595600	-3.87588200	0.34824100
C	-4.10147300	1.14752200	3.33768600
H	-4.59814300	2.06179600	2.98214400
H	-3.30425500	1.47484900	4.01956300
H	-4.82083200	0.54451700	3.90342900
C	-3.19335900	-2.35929900	-1.24833200
C	-4.32081700	-2.12304000	-2.06134100
C	-4.46180300	-2.87296800	-3.23209100
H	-5.32698400	-2.69959200	-3.87686400
C	-3.51309900	-3.81748300	-3.59860200
H	-3.63563700	-4.38922500	-4.52146900
C	-2.39668400	-4.02067200	-2.79590200
H	-1.64437700	-4.75059200	-3.09805500
C	-2.21059500	-3.29942600	-1.61463500
C	-5.35454900	-1.05638100	-1.74732500
H	-5.09414000	-0.59884700	-0.78043700
C	-6.76341300	-1.63500400	-1.61305400
H	-7.11754600	-2.05021600	-2.56964700
H	-7.47606900	-0.85199100	-1.30973600
H	-6.80756600	-2.44391300	-0.86892600
C	-5.32349300	0.05208500	-2.80025500
H	-4.31248900	0.47072500	-2.90950700
H	-6.01706800	0.86375400	-2.52953900
H	-5.62782900	-0.33192200	-3.78650600
C	-1.00776900	-3.56698100	-0.73417900
H	-0.76773600	-2.62545000	-0.21598400
C	-1.34095300	-4.62106600	0.32397100
H	-1.64914900	-5.56360100	-0.15699500
H	-2.15587600	-4.30343500	0.98859600

H	-0.45850100	-4.82739400	0.94624900
C	0.23586400	-3.97990000	-1.51133600
H	0.12575500	-4.97863500	-1.96455000
H	1.09276200	-4.02873700	-0.82515800
H	0.47939400	-3.24733300	-2.29432100
C	-2.56395400	2.40041200	1.43533200
C	-3.32226600	3.28053600	0.63475700
C	-3.16269100	4.65653700	0.82804300
H	-3.74931000	5.35102100	0.22262300
C	-2.27502700	5.15427200	1.77274600
H	-2.16624600	6.23262300	1.90938900
C	-1.51659800	4.27347200	2.53581500
H	-0.81039900	4.66896900	3.26924100
C	-1.64094300	2.89045700	2.38430000
C	-4.32823200	2.78745800	-0.38849300
H	-4.17260800	1.70557600	-0.51865600
C	-5.75931000	2.97389600	0.11744100
H	-6.48299900	2.57814100	-0.61233900
H	-5.98944500	4.03969800	0.27581800
H	-5.92370500	2.45063700	1.07173300
C	-4.11982400	3.43268800	-1.75592200
H	-3.10930500	3.22177100	-2.13558100
H	-4.26083300	4.52428100	-1.72175000
H	-4.84232400	3.03049900	-2.48103400
C	-0.77968300	1.96380100	3.22158200
H	-1.21325000	0.95635500	3.15387600
C	-0.73842300	2.35495900	4.69773600
H	-1.74564700	2.48417000	5.12320300
H	-0.19082800	3.29704400	4.85594800
H	-0.21974800	1.57830800	5.28120100
C	0.63113100	1.87557300	2.64754700
H	1.26893000	1.23024000	3.26887800
H	1.10633600	2.86706700	2.60416600
H	0.61204700	1.46485800	1.62951600
C	-0.00492600	-0.00424500	-1.73244900

Cartesian coordinates of the optimized geometry of Int-**3** at PBE0-D3BJ/def2-SVP level of theory:

Ga	-1.74463700	0.27542500	0.26649900
Ga	0.92266600	-1.09171200	0.19129700
P	-0.34884600	1.40766200	-1.23237500
P	2.72860600	1.32354600	-0.81186700
O	-0.76969500	-1.15032300	0.84865100
N	-2.28732100	1.73635300	1.54515200
N	-3.57179800	-0.52823600	0.25472900
N	1.88852300	-2.10808300	1.60030900
N	1.49370500	-2.33209600	-1.27692200
N	2.17859300	2.58056100	-3.12948100
C	-3.31294400	1.61487500	2.37751500
C	-4.19964900	0.52730700	2.34772300
H	-4.95038400	0.50533200	3.13619400
C	-4.38007000	-0.39711500	1.30860700
C	-3.64049300	2.71021600	3.35643700
H	-4.06218100	2.27907700	4.27457600
H	-4.40561200	3.37233700	2.92278100
H	-2.77141100	3.32995400	3.60396300
C	-5.61775500	-1.24958500	1.39563500
H	-5.35257800	-2.30948300	1.49704700
H	-6.21664100	-1.17001900	0.47759700
H	-6.23363400	-0.95475600	2.25294900
C	-1.65008700	3.00803700	1.42307400
C	-0.42587700	3.24801500	2.07959800
C	0.14095000	4.52079800	1.98053100
H	1.07624500	4.72697000	2.50285000
C	-0.45940700	5.52562800	1.22906900
H	-0.00113400	6.51636000	1.17011900

C	-1.63897700	5.25868600	0.54706400
H	-2.09787600	6.03981800	-0.06450700
C	-2.25290600	4.00560000	0.62774700
C	0.25759400	2.16888600	2.89214700
H	-0.01126800	1.20249100	2.43397400
C	-0.23225300	2.15239000	4.34108400
H	0.31758300	1.39214600	4.91503600
H	-1.30161600	1.91630900	4.41707500
H	-0.06260200	3.13191600	4.81731900
C	1.77539100	2.27638100	2.85329900
H	2.14183000	3.17119700	3.38218600
H	2.14469400	2.29727300	1.81724600
H	2.21780800	1.40617600	3.35231000
C	-3.51089400	3.75378600	-0.18147900
H	-3.83856400	2.72315900	0.01934700
C	-4.65379900	4.68907600	0.21275600
H	-4.88295800	4.62841000	1.28665700
H	-5.57028400	4.43608400	-0.34289400
H	-4.40946300	5.73916600	-0.01335300
C	-3.21542500	3.86219700	-1.67803000
H	-2.90387800	4.88408400	-1.94718800
H	-4.11254900	3.62416400	-2.26940200
H	-2.40532700	3.17950600	-1.97600500
C	-3.97581400	-1.43978500	-0.76768300
C	-3.74754300	-2.82605300	-0.62495600
C	-4.27539900	-3.68392000	-1.59670100
H	-4.11559000	-4.76040900	-1.49601600
C	-4.98957400	-3.19766100	-2.68321200
H	-5.39865100	-3.88697500	-3.42570800
C	-5.15971000	-1.82554500	-2.83761200
H	-5.69229700	-1.44872300	-3.71194700
C	-4.65303200	-0.92459400	-1.89756900
C	-2.93842500	-3.41757900	0.51615200
H	-2.62664200	-2.59267400	1.17203000
C	-3.71287500	-4.45217700	1.33375800
H	-3.09596200	-4.80226900	2.17641200
H	-3.97291500	-5.33629500	0.72962000
H	-4.64944700	-4.05665000	1.75350200
C	-1.65128200	-4.04284300	-0.01714600
H	-1.02173900	-4.39413000	0.81399700
H	-1.06742600	-3.30356600	-0.57633100
H	-1.86033900	-4.90334600	-0.67431300
C	-4.82484700	0.57405000	-2.08782800
H	-3.89936200	1.04277000	-1.71221300
C	-5.98907800	1.13950200	-1.27040800
H	-5.83098400	1.03700300	-0.18885000
H	-6.93136500	0.63191700	-1.53259900
H	-6.11776500	2.21256200	-1.48190700
C	-4.97968900	0.97858600	-3.55102000
H	-4.18389000	0.55707900	-4.18131700
H	-4.93728700	2.07365800	-3.64413700
H	-5.94991400	0.65894000	-3.96326500
C	2.07917300	-3.41853200	1.46512100
C	1.87645600	-4.13252900	0.27366500
H	2.01157700	-5.21130900	0.34250600
C	1.69885600	-3.62330700	-1.01816500
C	2.54781300	-4.22833800	2.64376600
H	2.96711600	-5.18559600	2.31222600
H	1.70140800	-4.43742200	3.31314100
H	3.29589900	-3.68860400	3.23865400
C	1.74177900	-4.63642400	-2.13278100
H	2.51011700	-5.39845800	-1.94955200
H	1.90753500	-4.17941100	-3.11464900
H	0.77063700	-5.15610700	-2.15381200
C	2.17177600	-1.51274900	2.86561500
C	3.41855700	-0.87017500	3.03440300
C	3.74927800	-0.37843400	4.29924300
H	4.71391600	0.10924000	4.45094000

C	2.85823800	-0.48125500	5.36278900
H	3.12973300	-0.08722300	6.34502500
C	1.61457000	-1.06837800	5.16605500
H	0.91129400	-1.12943600	6.00049500
C	1.24153200	-1.59883500	3.92533300
C	4.38349700	-0.73484300	1.87150100
H	3.77069300	-0.67668600	0.95702600
C	5.28367700	-1.96438400	1.73766900
H	5.85681900	-2.13159500	2.66385800
H	6.00295000	-1.82859400	0.91538300
H	4.70759900	-2.87507300	1.52323900
C	5.21505400	0.54204900	1.92286800
H	5.96200900	0.51949400	2.73253100
H	4.57803600	1.42774700	2.05953600
H	5.76073200	0.66691100	0.97540600
C	-0.12395900	-2.25107600	3.78145100
H	-0.20087700	-2.64336500	2.75741300
C	-0.33196200	-3.39223100	4.78021500
H	0.47786600	-4.13609300	4.75946200
H	-1.27661600	-3.91577700	4.56437100
H	-0.39730000	-3.01518300	5.81343800
C	-1.25216700	-1.23260100	3.93204500
H	-2.22696500	-1.72077100	3.77861800
H	-1.16470500	-0.44424100	3.17732700
H	-1.25652800	-0.77766900	4.93564800
C	1.79227000	-1.82699800	-2.58095500
C	3.14940200	-1.59185800	-2.91369000
C	3.44812400	-1.13062500	-4.19835500
H	4.48944000	-0.95331900	-4.47363400
C	2.44514200	-0.88054000	-5.12704500
H	2.69897300	-0.51842600	-6.12619900
C	1.11738700	-1.07391900	-4.77116200
H	0.33086900	-0.85669800	-5.49564200
C	0.76360600	-1.54143400	-3.50107800
C	4.28198200	-1.82822800	-1.92652300
H	3.84751800	-1.73734200	-0.91856900
C	4.88620400	-3.22918700	-2.05001200
H	5.72443100	-3.34625300	-1.34524000
H	5.27437900	-3.39580400	-3.06787800
H	4.16134400	-4.02120100	-1.83147600
C	5.39653700	-0.79093800	-2.04798200
H	6.10123900	-0.89613300	-1.20866900
H	4.98819800	0.22880700	-2.02615700
H	5.98056800	-0.92280600	-2.97258200
C	-0.69776700	-1.77409100	-3.17671000
H	-0.80677700	-1.70429500	-2.08182900
C	-1.15616000	-3.16790700	-3.60803500
H	-0.58757700	-3.96268200	-3.11185700
H	-1.03274600	-3.29439300	-4.69593600
H	-2.21857800	-3.31018700	-3.36199100
C	-1.61368800	-0.72396600	-3.79316100
H	-2.62677000	-0.85197200	-3.39146600
H	-1.68329100	-0.83041800	-4.88778300
H	-1.27087100	0.29377900	-3.55385000
C	1.13075600	0.73201600	-0.59101700
C	2.27835600	2.92712800	-1.81561000
N	2.18086300	2.25967500	-4.20133200
Si	3.59115600	4.31395000	-1.53125700
C	3.18923300	5.72129200	-2.70702300
H	2.18700900	6.12927000	-2.50230200
H	3.91743200	6.54024200	-2.59243300
H	3.21920000	5.39727900	-3.75953200
C	3.41438100	4.83153000	0.25120900
H	3.64822200	3.99319700	0.92316100
H	4.09880500	5.66579200	0.47296000
H	2.38455900	5.15737700	0.45855200
C	5.26655900	3.56779500	-1.90952200
H	5.32514900	3.20198400	-2.94674600

H	6.06282000	4.31652700	-1.77113700
H	5.46827800	2.72013200	-1.23664500
H	1.23297400	3.19194400	-1.47909100

Cartesian coordinates of the optimized geometry of Int-**4** at PBE0-D3BJ/def2-SVP level of theory:

Ga	-1.71768300	0.51427500	0.13970100
Ga	0.83632600	-1.08297100	0.26091900
P	-0.35189900	1.10574200	-1.71418500
P	2.65891900	0.76834200	-1.79284600
O	-0.78992700	-0.78152400	1.01351500
N	-1.94520700	2.33429000	0.95454100
N	-3.58489800	-0.04464400	0.54026900
N	1.87534100	-1.70366600	1.83670900
N	1.16752100	-2.75586200	-0.76814200
C	-2.84515400	2.57612700	1.90195900
C	-3.84927500	1.66223300	2.25081600
H	-4.49320200	1.95855600	3.07690300
C	-4.24366000	0.50132300	1.56379300
C	-2.81773100	3.88465100	2.63922000
H	-3.75643100	4.05717600	3.17872500
H	-2.62513400	4.72649200	1.96043200
H	-1.99415500	3.87093300	3.37020800
C	-5.53371800	-0.12420000	2.02124200
H	-5.34692500	-1.10890700	2.46880900
H	-6.21248100	-0.29572500	1.17464900
H	-6.03354000	0.51079600	2.76152900
C	-1.21298300	3.41776100	0.37161300
C	0.01148800	3.85836900	0.90882500
C	0.68898300	4.89943100	0.26556900
H	1.64236600	5.24258400	0.67461300
C	0.18295400	5.49252900	-0.88298700
H	0.73278300	6.29873500	-1.37461800
C	-1.02860100	5.05256600	-1.40304000
H	-1.42799100	5.52027900	-2.30514800
C	-1.74537600	4.02143800	-0.79274400
C	0.63450100	3.22998500	2.13386600
H	-0.06038600	2.46443000	2.50613200
C	0.86913900	4.24042400	3.25654800
H	1.23638100	3.72459600	4.15746800
H	-0.04663300	4.78973500	3.52198200
H	1.62878100	4.98586800	2.97239200
C	1.93693800	2.52928300	1.76819600
H	2.67272600	3.24054800	1.36075300
H	1.77147400	1.75853200	1.00319100
H	2.37711300	2.05617200	2.65306300
C	-3.09243800	3.61118100	-1.35374400
H	-3.33141100	2.62104700	-0.93709500
C	-4.18233300	4.57519800	-0.88008100
H	-4.25721600	4.59558900	0.21691900
H	-5.16604100	4.28142300	-1.27716100
H	-3.97057500	5.60115800	-1.22152400
C	-3.08834400	3.48215300	-2.87394100
H	-2.95212200	4.45570800	-3.37012400
H	-4.04955400	3.07639200	-3.22359700
H	-2.28377600	2.81190600	-3.21190800
C	-4.16559100	-1.19522700	-0.07971800
C	-4.01886900	-2.47261000	0.50509700
C	-4.70651100	-3.54533100	-0.07406900
H	-4.61128500	-4.53803700	0.37281500
C	-5.49474000	-3.37750200	-1.20382700
H	-6.02609900	-4.22879300	-1.63578300
C	-5.58393600	-2.12423800	-1.80077400
H	-6.17877800	-2.00630300	-2.70739400
C	-4.92319700	-1.01757700	-1.26126800
C	-3.12907300	-2.74007400	1.70642600

H	-2.67952200	-1.78606800	2.01471500
C	-3.87867500	-3.35351800	2.88981000
H	-3.19414100	-3.47726600	3.74374100
H	-4.27966100	-4.35023900	2.64521400
H	-4.72288300	-2.73691400	3.23168300
C	-1.96631000	-3.64697900	1.30696900
H	-1.26864200	-3.77034900	2.14869400
H	-1.40082600	-3.20507200	0.47899600
H	-2.31762100	-4.64812600	1.00668600
C	-5.02469400	0.34369200	-1.93107300
H	-4.03127600	0.81458000	-1.83379600
C	-6.03693600	1.26131200	-1.24087000
H	-5.73335900	1.53070500	-0.22084200
H	-7.02745100	0.78108500	-1.19410600
H	-6.14789100	2.19866700	-1.80723600
C	-5.34391400	0.25998100	-3.42111300
H	-4.67084800	-0.42865800	-3.95130000
H	-5.23977000	1.25296100	-3.88249500
H	-6.37988700	-0.06937700	-3.59863300
C	1.94878600	-3.00523300	2.11119700
C	1.55747900	-4.02772800	1.23182900
H	1.60728800	-5.03987900	1.63122600
C	1.28334100	-3.92358700	-0.13641100
C	2.48849700	-3.45107100	3.44293400
H	2.72793100	-4.52062300	3.42544600
H	1.74277400	-3.27499700	4.23086300
H	3.38390800	-2.88220300	3.72658000
C	1.10262000	-5.22026400	-0.88015200
H	1.79969200	-5.98820900	-0.52126000
H	1.21142100	-5.10398400	-1.96437900
H	0.08381900	-5.58657700	-0.67485900
C	2.36537300	-0.78451700	2.81256300
C	3.66641200	-0.26333800	2.64141800
C	4.19426200	0.55455000	3.64283900
H	5.20412100	0.95418400	3.53360500
C	3.44303300	0.88673200	4.76593600
H	3.86784600	1.53259800	5.53800900
C	2.14235100	0.41368000	4.88980500
H	1.54839900	0.69815500	5.76201100
C	1.57547700	-0.43116200	3.92810300
C	4.48053800	-0.59744400	1.40617800
H	3.76257700	-0.80680200	0.59647200
C	5.31303300	-1.86377300	1.61336200
H	5.99174400	-1.74818700	2.47366600
H	5.92689100	-2.07473900	0.72462700
H	4.68072100	-2.74444900	1.79439300
C	5.35051100	0.56098300	0.93077600
H	6.18043300	0.77170800	1.62372400
H	4.75764500	1.47898400	0.80686900
H	5.79321800	0.31620600	-0.04616500
C	0.15293900	-0.92687600	4.12746400
H	-0.07469600	-1.63057800	3.31367200
C	-0.03762900	-1.63310400	5.47149400
H	0.69803400	-2.43296200	5.64280300
H	-1.04193000	-2.08204200	5.52398200
H	0.04853200	-0.92842900	6.31388900
C	-0.85364200	0.21433400	3.99490300
H	-1.87899400	-0.15809500	4.14364500
H	-0.80775900	0.64025300	2.98685800
H	-0.66887400	1.00736100	4.73807800
C	1.36210800	-2.69409400	-2.18255900
C	2.68436900	-2.75193200	-2.68608800
C	2.86665300	-2.71516400	-4.07118300
H	3.87842200	-2.76526600	-4.47771400
C	1.78890800	-2.59488800	-4.93897000
H	1.95429800	-2.56354800	-6.01851300
C	0.50215600	-2.49109800	-4.42667000
H	-0.33958800	-2.37227800	-5.11111700

C	0.26328800	-2.53397300	-3.05033200
C	3.89974600	-2.84918000	-1.77668600
H	3.59391700	-2.46370200	-0.79126000
C	4.36766800	-4.29359000	-1.58439600
H	5.25242700	-4.32467200	-0.92916700
H	4.64763500	-4.74134500	-2.55166200
H	3.59789100	-4.92904200	-1.13165200
C	5.07139800	-1.99955500	-2.26691900
H	5.86914600	-1.98635500	-1.50856900
H	4.76030000	-0.96381700	-2.45915300
H	5.51668000	-2.40990000	-3.18675400
C	-1.15628300	-2.44501900	-2.53082600
H	-1.10733400	-2.05809700	-1.49898600
C	-1.82557400	-3.81846100	-2.47383900
H	-1.27870900	-4.51457500	-1.82673600
H	-1.87613500	-4.26390200	-3.48056800
H	-2.84961100	-3.72848800	-2.08299800
C	-2.02266200	-1.48602900	-3.33800400
H	-2.97194100	-1.32755900	-2.81139500
H	-2.26733000	-1.89187500	-4.33258900
H	-1.53378900	-0.50968700	-3.47928800
C	1.05130900	0.38855400	-1.07436500
C	2.71912000	2.44313500	-1.80955000
H	1.84698100	2.99666900	-1.42901700
Si	4.19999000	3.43546600	-2.35397300
C	5.57294400	2.29888600	-2.95748400
H	5.91098500	1.62756300	-2.15288100
H	5.22713900	1.67169200	-3.79410900
H	6.44187200	2.88145600	-3.30284400
C	4.81087300	4.45901600	-0.89011800
H	5.13135600	3.80768200	-0.06202800
H	5.66321800	5.09750600	-1.17324400
H	4.00782100	5.11256100	-0.51374000
C	3.66530700	4.60596900	-3.73123800
H	3.31180600	4.04082500	-4.60757400
H	2.83654700	5.24718800	-3.39081700
H	4.49427400	5.25774200	-4.05110600

Cartesian coordinates of the optimized geometry of Int-**5** at PBE0-D3BJ/def2-SVP level of theory:

Ga	-1.71646100	-0.11076600	0.29074600
Ga	1.22223600	-0.97961700	-0.01009600
P	-0.49163000	1.30737600	-1.16164500
P	2.21259800	2.20283600	-0.20970200
O	-0.48183700	-1.42933600	0.44407400
N	-2.45858900	0.88846700	1.83298700
N	-3.34721000	-1.16006500	-0.04694300
N	2.26954500	-2.15203800	1.17578000
N	2.07010100	-1.68284400	-1.68897300
C	-3.45853000	0.38138600	2.55032200
C	-4.16881700	-0.77564300	2.19439800
H	-4.91440100	-1.10968300	2.91471700
C	-4.19269100	-1.42124100	0.95070500
C	-3.94106300	1.08170100	3.79171600
H	-4.08365600	0.34259700	4.59315800
H	-4.92070200	1.54317000	3.59908200
H	-3.25467100	1.86446800	4.13132000
C	-5.26807900	-2.45279900	0.75150300
H	-4.82529600	-3.45749000	0.71823100
H	-5.78775700	-2.31050200	-0.20564800
H	-5.99735500	-2.41881200	1.56897800
C	-2.05144500	2.24267600	2.06579000
C	-0.82770800	2.50822300	2.71376400
C	-0.44299800	3.84128900	2.87628200
H	0.50725500	4.06942900	3.35920100
C	-1.24616400	4.88469600	2.43232600

H	-0.92181600	5.91939600	2.56572200
C	-2.45207900	4.60651100	1.80758600
H	-3.07247000	5.42900100	1.44384200
C	-2.87309600	3.29123600	1.59978600
C	0.03000800	1.39197800	3.27630700
H	0.00442900	0.55631500	2.55447800
C	-0.52797700	0.88004300	4.60634500
H	0.13488200	0.10606600	5.01998800
H	-1.52614600	0.43876400	4.50164500
H	-0.59206700	1.70444400	5.33470400
C	1.48655100	1.78866200	3.46434800
H	1.60532000	2.52804100	4.27252400
H	1.91562500	2.20516500	2.54152300
H	2.07166800	0.90579200	3.74734400
C	-4.17166600	3.05833100	0.84758400
H	-4.33797100	1.97289700	0.77486300
C	-5.37250200	3.66385100	1.57683500
H	-5.44553600	3.31669000	2.61740100
H	-6.31002900	3.40066500	1.06193500
H	-5.30769800	4.76306200	1.60302700
C	-4.09030900	3.60575700	-0.57781700
H	-3.94565400	4.69738900	-0.57357400
H	-5.02313900	3.39678000	-1.12380700
H	-3.24053300	3.17216100	-1.12058300
C	-3.55173300	-1.81319600	-1.29971800
C	-3.08366500	-3.12815900	-1.51335500
C	-3.40229200	-3.74642400	-2.72809200
H	-3.05562200	-4.76683500	-2.90998200
C	-4.15151900	-3.09614000	-3.69999500
H	-4.39770900	-3.60474100	-4.63509500
C	-4.56847300	-1.78577400	-3.48871700
H	-5.12937000	-1.26918100	-4.26929600
C	-4.26462600	-1.11594900	-2.30103700
C	-2.25411900	-3.88996800	-0.49381300
H	-2.13421100	-3.24556600	0.38838300
C	-2.90066400	-5.21110500	-0.07284700
H	-2.30840000	-5.68300800	0.72693100
H	-2.94412500	-5.92433300	-0.91143400
H	-3.92765000	-5.08839200	0.30119400
C	-0.84648000	-4.15557900	-1.02425900
H	-0.23310900	-4.64560100	-0.25268400
H	-0.34945000	-3.21573800	-1.28708900
H	-0.86373500	-4.81233900	-1.90922500
C	-4.69893200	0.32400200	-2.08892400
H	-3.94025000	0.79174100	-1.44014000
C	-6.03879200	0.41548600	-1.35494000
H	-5.98607100	-0.00563100	-0.34180200
H	-6.82487600	-0.12149100	-1.90984200
H	-6.35296900	1.46610100	-1.25730200
C	-4.74451400	1.13023700	-3.38328100
H	-3.80686300	1.03985900	-3.94992300
H	-4.90033000	2.19473600	-3.15632600
H	-5.57260100	0.81395300	-4.03712400
C	2.72066400	-3.32975400	0.73709400
C	2.75832900	-3.71727100	-0.60828100
H	3.13220400	-4.72222000	-0.80034200
C	2.58171600	-2.90779900	-1.74048100
C	3.26066700	-4.32427600	1.72794700
H	3.76581800	-5.14957100	1.21326400
H	2.44480200	-4.73740300	2.33568700
H	3.96119000	-3.85139200	2.42938900
C	3.04422100	-3.50784100	-3.04199100
H	4.08746000	-3.84145400	-2.95826200
H	2.95381400	-2.81476200	-3.88530900
H	2.43937500	-4.40346400	-3.25140000
C	2.38833400	-1.87277200	2.57115800
C	3.49779100	-1.11214000	3.00204200
C	3.69125600	-0.94034400	4.37488000

H	4.54892700	-0.36621900	4.72940700
C	2.79534300	-1.47209100	5.29691500
H	2.96056300	-1.32594700	6.36694700
C	1.67876300	-2.16903800	4.85253300
H	0.96626600	-2.56365600	5.58133700
C	1.44712100	-2.38733300	3.48962200
C	4.46609300	-0.50998600	1.99834800
H	3.88334700	-0.28415300	1.08963100
C	5.56011100	-1.50510100	1.60375300
H	6.11131200	-1.84991300	2.49336500
H	6.28401800	-1.03288300	0.92230800
H	5.15330100	-2.38700400	1.09030500
C	5.08207700	0.80302500	2.47059900
H	5.78858900	0.65203200	3.30217400
H	4.31191800	1.51672100	2.79486900
H	5.64352400	1.26836500	1.64749800
C	0.21084600	-3.16162300	3.06220700
H	0.24300900	-3.26573600	1.96832800
C	0.13139300	-4.55039000	3.69973500
H	1.03358500	-5.15634800	3.53094400
H	-0.72563300	-5.10576100	3.28725100
H	-0.01618700	-4.48565500	4.78954000
C	-1.06470200	-2.38452700	3.38091400
H	-1.94576500	-2.92519000	3.00255700
H	-1.05208800	-1.40208200	2.89806600
H	-1.19363900	-2.24612500	4.46644000
C	2.28183000	-0.77226200	-2.77223500
C	3.54096500	-0.14191100	-2.89914500
C	3.71368100	0.79932200	-3.91853400
H	4.67992300	1.29723700	-4.02681500
C	2.67977900	1.11892800	-4.78726000
H	2.83164200	1.86271000	-5.57319400
C	1.44904700	0.48731400	-4.65288700
H	0.64111400	0.74427500	-5.33825300
C	1.22309700	-0.46407000	-3.65437100
C	4.72409100	-0.46131200	-1.99963200
H	4.35626900	-1.07222300	-1.16125800
C	5.78189600	-1.27655200	-2.74897600
H	6.59123900	-1.58070600	-2.06650800
H	6.23225100	-0.67696900	-3.55621800
H	5.36441300	-2.18155000	-3.20822500
C	5.37135900	0.79123800	-1.41111700
H	6.19150500	0.51230000	-0.73220000
H	4.64561200	1.39590100	-0.84917900
H	5.80579500	1.42975700	-2.19589000
C	-0.11980900	-1.16493300	-3.56172000
H	-0.35305000	-1.29134900	-2.48917900
C	-0.07835900	-2.56261700	-4.18301100
H	0.60915800	-3.23252900	-3.65590700
H	0.23579200	-2.50721000	-5.23768500
H	-1.07895400	-3.01800000	-4.14398400
C	-1.25856000	-0.37422200	-4.19109500
H	-2.21332000	-0.86106600	-3.95596500
H	-1.17207700	-0.34179800	-5.28906100
H	-1.29235000	0.65684000	-3.81043200
C	1.08406300	0.96322300	-0.37667900
C	1.33915300	3.54192400	-1.18122900
Si	2.32919600	5.15352800	-1.30688400
C	1.34604100	6.37663400	-2.34031600
H	0.33943800	6.49532300	-1.91012900
H	1.83044400	7.36541100	-2.37162700
H	1.23349000	6.01908900	-3.37613300
C	2.56258000	5.79786600	0.44234700
H	3.15815600	5.09110700	1.04183600
H	3.06735700	6.77649900	0.46019400
H	1.57701400	5.90144500	0.92337300
C	3.98060800	4.76032800	-2.12084600
H	3.82682000	4.29103300	-3.10598500

H	4.58304300	5.67107500	-2.26677800
H	4.56573300	4.05925800	-1.50507700
N	0.00357100	3.83893600	-0.62680900
N	-0.85724900	2.95879500	-0.63146500
H	1.16745600	3.06754100	-2.17580100

Cartesian coordinates of the optimized geometry of Int-**6** at PBE0-D3BJ/def2-SVP level of theory:

Ga	-2.00295700	-0.32984300	-0.08413000
Ga	2.16859900	-0.25538300	0.58717000
P	0.11735000	-1.17821900	-0.33005900
P	1.14527500	-0.67960000	2.59762200
O	-1.48410000	-0.76423700	1.75081300
C	-0.21067500	-0.88736000	1.47282400
N	-3.60027400	-1.26618300	-0.63695100
N	-2.74725400	1.44798300	-0.01413800
N	3.68767200	-1.17955300	-0.22612300
N	2.74003300	1.54129300	0.09157400
C	-4.77307400	-0.65976100	-0.78015700
C	-4.97373600	0.71957400	-0.59347600
H	-5.99240800	1.07391100	-0.74222800
C	-4.03375200	1.69832900	-0.22873100
C	-5.96720000	-1.48568700	-1.16358400
H	-6.86992600	-0.86897700	-1.23918400
H	-6.14600200	-2.27967800	-0.42182900
H	-5.80333800	-1.98543600	-2.13121900
C	-4.52648700	3.10822900	-0.07166900
H	-5.60185000	3.17809400	-0.27082700
H	-3.99912100	3.78750200	-0.75974800
H	-4.33480300	3.47551400	0.94865300
C	4.52187800	-0.59844900	-1.07323500
C	4.50593900	0.77887300	-1.36678300
H	5.24549600	1.11981600	-2.08973100
C	3.70288000	1.77747800	-0.79217200
C	5.56084700	-1.43929100	-1.76011500
H	5.08976300	-2.26091400	-2.32189800
H	6.23585000	-1.90372800	-1.02397200
H	6.16369300	-0.84093900	-2.45298100
C	3.97477100	3.19867000	-1.19953900
H	3.06537400	3.67565900	-1.59707600
H	4.75917600	3.24912300	-1.96343300
H	4.29676200	3.79863600	-0.33358200
C	1.99682300	2.63068300	0.68573500
H	1.36905400	3.16085900	-0.05220700
H	1.34110500	2.21885400	1.46781700
H	2.65572300	3.37477900	1.16348800
C	3.76400600	-2.59883600	0.04533000
H	3.03484500	-2.84570100	0.83121500
H	3.51747900	-3.20455400	-0.84361000
H	4.76206400	-2.90258000	0.40274300
C	-1.83675900	2.49463500	0.40006900
H	-0.81938300	2.08222400	0.43348500
H	-1.83192100	3.34734500	-0.29907700
H	-2.07452500	2.87407000	1.40805200
C	-3.46803000	-2.69627900	-0.83569000
H	-2.40743900	-2.97026900	-0.73673500
H	-4.03788800	-3.27065700	-0.08607400
H	-3.80509500	-3.00939400	-1.83763000

Cartesian coordinates of the optimized geometry of Int-**7** at PBE0-D3BJ/def2-SVP level of theory:

Ga	-1.98639700	-0.83509800	0.00073800
Ga	1.98638700	-0.83508800	-0.00051100
P	-1.62303800	-3.07807400	0.00116300

P	1.62304400	-3.07806400	-0.00029400
O	-0.00000700	-0.95216500	0.00025800
N	-2.44925600	0.37132600	1.45030000
N	-2.44930300	0.36942700	-1.45049200
N	2.44946400	0.37083100	-1.45041100
N	2.44906200	0.36995200	1.45038100
C	-2.86768100	1.61690400	1.26409500
C	-3.06630800	2.19655800	-0.00128800
H	-3.41693500	3.22747400	-0.00196100
C	-2.86761400	1.61525600	-1.26593200
C	-3.14788400	2.47126700	2.46769400
H	-3.92949700	2.01670700	3.09652200
H	-3.47669300	3.47537200	2.17627500
H	-2.24955900	2.56575800	3.09772600
C	-3.14764500	2.46811300	-2.47064300
H	-3.92875900	2.01249500	-3.09931400
H	-2.24904600	2.56223900	-3.10035000
H	-3.47697600	3.47243000	-2.18054500
C	2.86781800	1.61650100	-1.26456000
C	3.06621500	2.19660100	0.00064500
H	3.41680500	3.22753000	0.00101900
C	2.86740700	1.61570600	1.26546600
C	3.14818900	2.47046600	-2.46840200
H	3.93003500	2.01579800	-3.09686200
H	3.47677200	3.47472800	-2.17726800
H	2.25001000	2.56458800	-3.09869800
C	3.14730900	2.46896000	2.46992600
H	3.92830600	2.01352200	3.09887100
H	2.24861700	2.56333800	3.09946500
H	3.47672900	3.47316300	2.17953500
C	-0.00000100	-2.35598200	0.00037800
C	2.23554200	-0.15877400	2.78211100
H	3.14259600	-0.10259100	3.40668700
H	1.42415000	0.37028700	3.31163600
H	1.94939800	-1.21803400	2.69639500
C	2.23704800	-0.15724400	-2.78259000
H	1.95096600	-1.21658000	-2.69766900
H	1.42601800	0.37196500	-3.31253300
H	3.14458300	-0.10063600	-3.40642700
C	-2.23590700	-0.15970700	-2.78208100
H	-3.14306200	-0.10383700	-3.40654300
H	-1.42465600	0.36926800	-3.31190500
H	-1.94964500	-1.21891000	-2.69605900
C	-2.23648900	-0.15625700	2.78261500
H	-1.95027700	-1.21558300	2.69801000
H	-1.42543500	0.37326500	3.31220900
H	-3.14390800	-0.09956400	3.40661400

Cartesian coordinates of the optimized geometry of TS-**1** at PBE0-D3BJ/def2-SVP level of theory:

Ga	1.86700700	0.29731700	0.52500800
Ga	-1.76848000	-0.25750000	-0.74705700
P	-0.10230400	-0.76149200	0.73535900
P	-0.62850900	-0.13455000	-2.80715400
O	1.77305600	0.17100700	-1.45292400
C	0.57537300	-0.16604100	-1.62763000
N	3.39608000	-0.88778800	0.88971400
N	2.79890800	1.95781700	0.88779000
N	-2.85045800	-1.92988200	-0.72944000
N	-3.33119600	0.95493100	-0.74438600
C	4.47888300	-0.47055100	1.53054800
C	4.67700800	0.86191400	1.94382100
H	5.58384200	1.04579500	2.51845600
C	3.94115100	1.99258900	1.58786700
C	5.61179500	-1.41396200	1.82863100
H	5.89877200	-1.32098500	2.88577400

H	6.49007300	-1.12569100	1.23189900
H	5.36754700	-2.45806400	1.60633300
C	4.48030600	3.32864600	2.01255200
H	5.43177700	3.21598700	2.54465700
H	3.76000700	3.83864400	2.66908600
H	4.62677200	3.98963200	1.14699200
C	3.31606900	-2.23458300	0.40571100
C	2.71350000	-3.22268700	1.21246800
C	2.68830300	-4.53528200	0.73420900
H	2.22670600	-5.31494300	1.34300200
C	3.23653600	-4.86380400	-0.49987200
H	3.21199800	-5.89794500	-0.85188500
C	3.79582000	-3.87112600	-1.29364800
H	4.20446200	-4.13032400	-2.27309700
C	3.83819100	-2.53997000	-0.86832500
C	2.15020600	-2.90983100	2.58614100
H	1.97494700	-1.82347200	2.62763300
C	3.14492700	-3.26938400	3.69213700
H	4.09683100	-2.73097800	3.59004600
H	3.37006100	-4.34813400	3.67856600
H	2.72517200	-3.02606000	4.68087900
C	0.80694400	-3.58728500	2.83000300
H	0.38171700	-3.24355500	3.78576500
H	0.89947000	-4.68298900	2.89258300
H	0.09478300	-3.34185700	2.03061900
C	4.46298400	-1.49210800	-1.77176500
H	4.26167700	-0.50725900	-1.32661700
C	5.98007500	-1.66705500	-1.86416400
H	6.42555100	-0.86756100	-2.47704300
H	6.23829100	-2.63032600	-2.33265200
H	6.45958600	-1.64422400	-0.87555900
C	3.83864800	-1.49455300	-3.16638200
H	4.24083100	-0.66112300	-3.76317100
H	2.74900900	-1.37624800	-3.11528000
H	4.06392700	-2.42628900	-3.70905700
C	2.25852000	3.19033700	0.40295800
C	1.36457400	3.92697000	1.20461500
C	0.89837100	5.15199000	0.71844800
H	0.19530900	5.73264600	1.31951000
C	1.32786800	5.64774200	-0.50660800
H	0.96784700	6.61686000	-0.86077000
C	2.20199500	4.90197100	-1.28912900
H	2.52025100	5.28868800	-2.26029000
C	2.66914300	3.65543700	-0.86479500
C	0.91464000	3.42784200	2.56324500
H	1.57030600	2.58787600	2.84048500
C	1.04961900	4.49400800	3.64997100
H	2.06396800	4.92113400	3.68873400
H	0.82443300	4.06177400	4.63703600
H	0.34571900	5.32592100	3.49195900
C	-0.51294300	2.89195700	2.49852500
H	-0.83227800	2.52543700	3.48630800
H	-0.59640900	2.05651200	1.78761000
H	-1.21680600	3.67477700	2.18052400
C	3.61311900	2.86252800	-1.75094700
H	3.75507500	1.87840100	-1.28274600
C	4.98583500	3.53002200	-1.84771000
H	4.91351000	4.52840900	-2.30829000
H	5.66543500	2.92461300	-2.46785500
H	5.45500900	3.65157700	-0.85994000
C	3.02059800	2.61724800	-3.13734600
H	2.86616500	3.55901000	-3.68776000
H	2.06045000	2.08880400	-3.06876300
H	3.70297400	1.99274100	-3.73467700
C	-3.99630700	-1.93303600	-1.39894900
C	-4.65027500	-0.76042000	-1.82798900
H	-5.55714700	-0.91487000	-2.41001400
C	-4.41694300	0.55854900	-1.41281900

C	-4.67647100	-3.23972600	-1.69672500
H	-4.82608200	-3.82201900	-0.77629600
H	-4.04358400	-3.85605000	-2.35059800
H	-5.64527800	-3.07841100	-2.18343500
C	-5.49843000	1.56196800	-1.69765900
H	-5.98117100	1.87499200	-0.75981600
H	-6.26234600	1.13700300	-2.35904000
H	-5.08557400	2.47292500	-2.15109000
C	-2.38587600	-3.15533000	-0.15715700
C	-2.72034800	-3.43506200	1.18637900
C	-2.37831400	-4.68423800	1.70898600
H	-2.63891100	-4.92395200	2.74094600
C	-1.68758500	-5.62036900	0.94692400
H	-1.42749700	-6.59137300	1.37537900
C	-1.29960300	-5.29917700	-0.34595100
H	-0.71697500	-6.01619900	-0.92946500
C	-1.63363700	-4.06869200	-0.92161700
C	-3.41833400	-2.40198500	2.05247400
H	-3.08997300	-1.41736200	1.68218800
C	-4.94104100	-2.46140600	1.92143200
H	-5.41519400	-1.72213400	2.58662000
H	-5.32072600	-3.45751300	2.20110700
H	-5.27642200	-2.24299400	0.89793200
C	-2.99731100	-2.49489400	3.51548200
H	-1.90300100	-2.45769600	3.61330600
H	-3.35724400	-3.41994300	3.99255500
H	-3.41841000	-1.65627000	4.08737400
C	-1.12275700	-3.74360200	-2.30975500
H	-1.52964600	-2.76224600	-2.59732300
C	-1.54986500	-4.76386000	-3.36399900
H	-1.19677600	-4.45151400	-4.35899000
H	-2.64318200	-4.87981700	-3.41848600
H	-1.12338000	-5.75931200	-3.16155900
C	0.39892600	-3.60841400	-2.27667900
H	0.71894500	-2.91924700	-1.48151400
H	0.77631400	-3.22303700	-3.23642100
H	0.87762400	-4.58118400	-2.08574200
C	-3.29895400	2.27596900	-0.20368500
C	-3.85763800	2.50697400	1.07122000
C	-3.85210100	3.80909000	1.57789400
H	-4.28422500	4.00035900	2.56339200
C	-3.29799700	4.86031000	0.85643700
H	-3.29769100	5.87140200	1.27105500
C	-2.74287200	4.61681800	-0.39367200
H	-2.30555500	5.44198900	-0.96097900
C	-2.74369400	3.33476900	-0.95228700
C	-4.45277300	1.38723600	1.90165300
H	-4.45085300	0.47813000	1.28283100
C	-5.90072300	1.66971200	2.30070600
H	-6.33200200	0.80050600	2.82138400
H	-6.53175800	1.88405700	1.42486300
H	-5.97547300	2.53183500	2.98216800
C	-3.58783300	1.10825500	3.12962600
H	-2.58182800	0.77075500	2.84023300
H	-4.04605800	0.32776900	3.75496100
H	-3.47724000	2.01117500	3.75080200
C	-2.17230000	3.12971200	-2.33991000
H	-2.30035400	2.06910100	-2.60108500
C	-2.90458100	3.95918900	-3.39506800
H	-2.77654300	5.03940200	-3.22031900
H	-3.98542700	3.75248200	-3.41058800
H	-2.50588200	3.73602100	-4.39675200
C	-0.67761000	3.42700200	-2.36445900
H	-0.48317800	4.49460200	-2.18659600
H	-0.24423000	3.15487400	-3.33823400
H	-0.13962400	2.85933100	-1.59184900

Cartesian coordinates of the optimized geometry of TS-**2** at PBE0-D3BJ/def2-SVP level of theory:

Ga	2.61071400	0.14123200	-0.12198200
Ga	-2.52804800	-0.05790400	-0.15659300
P	1.22321900	-0.50977300	-1.84323800
P	-1.29313800	1.38803100	-1.20735200
O	0.88366700	0.95756100	0.34412300
N	3.78157300	1.71376900	-0.32002700
N	3.54436100	-0.61085700	1.41259200
N	-3.10009600	-1.91210800	-0.26426500
N	-3.90556000	0.50617700	1.10820800
C	4.76326000	2.01644100	0.50866900
C	5.08349500	1.24366400	1.64629300
H	5.89555300	1.62842600	2.26205000
C	4.54141600	0.02303900	2.04717200
C	5.61041100	3.23503800	0.27605900
H	6.66886800	2.99593800	0.44831900
H	5.32972100	4.01802600	0.99697800
H	5.48327500	3.64024800	-0.73480700
C	5.15166900	-0.63687900	3.25176800
H	5.66628800	-1.56645300	2.96482600
H	4.37522000	-0.92630400	3.97407500
H	5.87251900	0.02888500	3.74029000
C	3.46338400	2.51367600	-1.45963900
C	3.85015400	2.03658000	-2.73093200
C	3.47934600	2.77564300	-3.85466900
H	3.76839800	2.42511800	-4.84714000
C	2.73407100	3.94341200	-3.72993800
H	2.44279200	4.50399400	-4.62128200
C	2.34971400	4.38809600	-2.47231800
H	1.75209300	5.29834600	-2.38234900
C	2.69977200	3.68872500	-1.31239200
C	4.68822900	0.78019000	-2.87337800
H	4.49362600	0.15652500	-1.98576000
C	6.18008200	1.11780500	-2.86459000
H	6.78937300	0.20177300	-2.92476700
H	6.46964200	1.65682300	-1.95045100
H	6.44049300	1.75632700	-3.72399900
C	4.31061500	-0.06080400	-4.08724300
H	4.57506400	0.43619000	-5.03406300
H	3.23073100	-0.27202000	-4.09175500
H	4.84964500	-1.02071000	-4.06444800
C	2.25589300	4.22481100	0.03660500
H	2.59889300	3.51922400	0.80831100
C	2.88341200	5.59268400	0.32009400
H	3.97818100	5.58315200	0.21724300
H	2.63672500	5.92601800	1.34049500
H	2.49686100	6.35389300	-0.37611500
C	0.73451600	4.30575600	0.14745900
H	0.26324000	3.32611900	-0.01013500
H	0.31484400	5.00921200	-0.58912700
H	0.44540500	4.66387400	1.14828500
C	3.22024900	-1.94265000	1.81051700
C	3.99617000	-3.00938800	1.30288100
C	3.70401700	-4.30541100	1.73201000
H	4.29187800	-5.14298300	1.35089000
C	2.67213400	-4.54774700	2.63398700
H	2.46099000	-5.56788700	2.96297400
C	1.90105600	-3.49093400	3.10012000
H	1.08412700	-3.68732100	3.79914200
C	2.15090500	-2.17415300	2.69590500
C	5.08785000	-2.76643200	0.27666900
H	5.38264800	-1.70848900	0.34824100
C	6.34183200	-3.60085200	0.52224400
H	6.16043600	-4.67537900	0.36368100
H	6.72238000	-3.47306600	1.54749900
H	7.13899200	-3.30253400	-0.17569000

C	4.54207300	-2.98781200	-1.13642500
H	3.66430300	-2.35398900	-1.33462500
H	4.22870700	-4.03499200	-1.27412400
H	5.30907900	-2.75706900	-1.89265500
C	1.27715700	-1.05185700	3.22249800
H	1.64964000	-0.10711700	2.80000100
C	1.34782500	-0.94519600	4.74595500
H	0.93949900	-1.84352800	5.23577700
H	0.76258600	-0.08120500	5.09780600
H	2.38244100	-0.81603900	5.09867800
C	-0.16493000	-1.21648200	2.74628700
H	-0.21675600	-1.12544900	1.65236800
H	-0.80250400	-0.43231800	3.17463100
H	-0.58393000	-2.19155600	3.03760600
C	-4.03909800	-2.43109800	0.53387900
C	-4.67779200	-1.72455300	1.56354500
H	-5.36592000	-2.30333400	2.17744500
C	-4.67054600	-0.33912500	1.79335500
C	-4.52105100	-3.83163200	0.28082500
H	-5.25988500	-3.81594000	-0.53563200
H	-5.01014900	-4.23867300	1.17450200
H	-3.71228600	-4.49891300	-0.03911800
C	-5.59790500	0.19946200	2.84320100
H	-6.20750300	1.02120600	2.44059300
H	-5.01983000	0.62328100	3.67747600
H	-6.25507100	-0.58700800	3.23208600
C	-2.71524900	-2.65194200	-1.43456400
C	-3.53411900	-2.56581800	-2.57822500
C	-3.19378400	-3.33161500	-3.69687200
H	-3.81603700	-3.27552600	-4.59326000
C	-2.07161300	-4.14734000	-3.68993000
H	-1.81917300	-4.74149800	-4.57130200
C	-1.24972500	-4.18248400	-2.56923400
H	-0.34851600	-4.79524800	-2.58885400
C	-1.54139700	-3.43409500	-1.42585400
C	-4.72264200	-1.62627100	-2.66469100
H	-4.89677800	-1.19878100	-1.66494700
C	-6.01145000	-2.33520400	-3.07788000
H	-5.94549000	-2.73343100	-4.10233100
H	-6.85977600	-1.63353200	-3.05312600
H	-6.25033800	-3.17734600	-2.41051400
C	-4.39903900	-0.46857600	-3.61168700
H	-3.50057600	0.07765800	-3.28234000
H	-5.24122900	0.23844900	-3.66290300
H	-4.21040800	-0.83857700	-4.63171900
C	-0.60409000	-3.45371300	-0.23263100
H	-0.51861900	-2.40662100	0.10339100
C	-1.13253900	-4.28821300	0.93611100
H	-1.36231300	-5.31592000	0.61055900
H	-2.03500500	-3.86037500	1.39259000
H	-0.36495600	-4.35194900	1.72124000
C	0.80538300	-3.90645600	-0.58924800
H	0.84686600	-4.98621900	-0.80830200
H	1.47694200	-3.72045600	0.25895400
H	1.19363900	-3.34720800	-1.45188300
C	-4.04316200	1.91878600	1.28450800
C	-4.77010700	2.64227900	0.31427500
C	-4.93566600	4.01585500	0.50332700
H	-5.50059400	4.59509700	-0.22810600
C	-4.37354600	4.66436900	1.59745900
H	-4.51462800	5.73987200	1.72746000
C	-3.60188300	3.94782600	2.50122900
H	-3.12048700	4.47006700	3.33130500
C	-3.40872800	2.57045700	2.35847000
C	-5.33481400	1.95519100	-0.91654300
H	-4.61540500	1.16633500	-1.19905000
C	-6.67142400	1.26709200	-0.63756500
H	-7.05206500	0.77776800	-1.54817400

H	-7.42493400	1.99983200	-0.30693200
H	-6.58465100	0.49459500	0.13950100
C	-5.43878200	2.88521600	-2.11951900
H	-4.48008100	3.38664400	-2.31717000
H	-6.21389000	3.65523500	-1.98076100
H	-5.71223500	2.31199600	-3.01716600
C	-2.46668200	1.85421600	3.30539000
H	-2.56414200	0.77319900	3.11930400
C	-2.79932500	2.10087100	4.77631800
H	-3.84753200	1.86069400	5.01364400
H	-2.63057600	3.15158700	5.05932000
H	-2.15538100	1.48349400	5.42192800
C	-1.02076000	2.25002800	2.99334900
H	-0.32424900	1.76252200	3.69426600
H	-0.88815800	3.33845700	3.09763700
H	-0.72838300	1.96907300	1.96999500
C	0.31954400	0.56276400	-0.78252100

Cartesian coordinates of the optimized geometry of TS-**3** at PBE0-D3BJ/def2-SVP level of theory:

Ga	1.86479300	-0.00685000	-0.22300900
Ga	-1.86482300	0.00687200	-0.22300900
P	1.49325600	-0.69835800	-2.37578800
P	-1.49338400	0.69855400	-2.37574500
O	-0.00001600	0.00011500	0.01351000
N	2.78400000	1.75251600	-0.14988000
N	2.79003700	-0.77069900	1.32331400
N	-2.78389300	-1.75257700	-0.14996600
N	-2.79014500	0.77062300	1.32329900
C	3.56355000	2.12269600	0.85530900
C	3.82419400	1.31665100	1.97992400
H	4.44193200	1.77465000	2.75142900
C	3.54531800	-0.04087300	2.15309500
C	4.29262700	3.43885100	0.82196900
H	5.37009500	3.25776000	0.69319100
H	4.16572100	3.95259800	1.78576500
H	3.95206000	4.09179300	0.01138100
C	4.16329100	-0.70795300	3.35074600
H	4.64910900	-1.65386000	3.07355600
H	3.39106700	-0.95906100	4.09188700
H	4.89903600	-0.04886900	3.82578800
C	2.82009700	2.47964000	-1.38432800
C	3.88683800	2.24680300	-2.27739500
C	3.90231100	2.94802000	-3.48649600
H	4.72080900	2.78052900	-4.19095300
C	2.88931600	3.83837500	-3.81261800
H	2.91335200	4.37232200	-4.76550100
C	1.83330400	4.03662300	-2.93020900
H	1.03149100	4.72339900	-3.20288400
C	1.77154900	3.36579300	-1.70648700
C	5.00434000	1.25872800	-1.99096000
H	4.79091800	0.77276600	-1.02659600
C	6.36187700	1.95349800	-1.86829400
H	7.14237800	1.23136200	-1.58075200
H	6.34888400	2.75826400	-1.11981300
H	6.66315600	2.40334300	-2.82757200
C	5.06611600	0.16116600	-3.05337100
H	5.33127900	0.57461200	-4.03899200
H	4.09810600	-0.34964800	-3.15809400
H	5.83340200	-0.58437400	-2.79221900
C	0.62608500	3.61710500	-0.74441100
H	0.37679600	2.64716400	-0.28199100
C	1.03064000	4.58992000	0.36532700
H	1.84028200	4.19891800	0.99313000
H	0.17029600	4.79098400	1.02028500
H	1.36536400	5.54801700	-0.06446200

C	-0.63635800	4.12760200	-1.42627600
H	-0.93569000	3.47159800	-2.25641100
H	-0.51164900	5.15593600	-1.80344900
H	-1.45882000	4.14594800	-0.69846000
C	2.71154900	-2.18241400	1.52692100
C	3.53141300	-3.02126200	0.73937500
C	3.50385800	-4.39585900	0.98921800
H	4.13486300	-5.06085900	0.39732500
C	2.67844000	-4.93301600	1.97114700
H	2.67158700	-6.01056800	2.15087200
C	1.84907500	-4.09740000	2.70849300
H	1.18673400	-4.52672100	3.46403200
C	1.84406500	-2.71426600	2.50297000
C	4.44113500	-2.44965100	-0.33337500
H	3.95463300	-1.53784100	-0.71448300
C	5.79612600	-2.03217500	0.24055200
H	6.31361100	-2.89351000	0.69284600
H	5.69416900	-1.25377900	1.01010200
H	6.44285100	-1.62770600	-0.55387800
C	4.61265900	-3.37983500	-1.52920900
H	3.63878000	-3.69604300	-1.93006500
H	5.19430900	-4.28042700	-1.27625800
H	5.15376000	-2.86029300	-2.33326200
C	0.90434400	-1.84174800	3.31354700
H	1.18551500	-0.79521000	3.13000500
C	0.99734400	-2.10400500	4.81657300
H	0.60463000	-3.09874800	5.07922400
H	0.39842600	-1.36318900	5.36925400
H	2.03133600	-2.05180800	5.19014700
C	-0.53516600	-2.00547700	2.83341900
H	-0.62785200	-1.71467900	1.77909200
H	-1.21767100	-1.37675900	3.42430400
H	-0.87407200	-3.04742700	2.93655300
C	-3.56350000	-2.12279700	0.85516600
C	-3.82426400	-1.31677700	1.97977300
H	-4.44201900	-1.77483100	2.75123300
C	-3.54542700	0.04074300	2.15303000
C	-4.29242100	-3.43904600	0.82186600
H	-5.37004300	-3.25808500	0.69422500
H	-4.16450500	-3.95321200	1.78531100
H	-3.95246700	-4.09157700	0.01069100
C	-4.16342900	0.70775400	3.35069900
H	-4.64947400	1.65354300	3.07349900
H	-3.39119100	0.95906500	4.09175100
H	-4.89899400	0.04854400	3.82584400
C	-2.81990600	-2.47962200	-1.38446600
C	-3.88659900	-2.24672000	-2.27757300
C	-3.90202200	-2.94786200	-3.48671800
H	-4.72049300	-2.78033000	-4.19119700
C	-2.88901200	-3.83819500	-3.81285300
H	-2.91300700	-4.37208500	-4.76576800
C	-1.83302900	-4.03648100	-2.93041800
H	-1.03119500	-4.72322500	-3.20311000
C	-1.77132600	-3.36572700	-1.70665100
C	-5.00413700	-1.25870100	-1.99109700
H	-4.79059600	-0.77258800	-1.02683600
C	-6.36157900	-1.95359800	-1.86808700
H	-6.66303700	-2.40349100	-2.82728800
H	-7.14208200	-1.23153500	-1.58036800
H	-6.34831400	-2.75834500	-1.11959500
C	-5.06619600	-0.16128300	-3.05363600
H	-4.09826600	0.34964900	-3.15853300
H	-5.83354800	0.58418500	-2.79247200
H	-5.33143800	-0.57487200	-4.03917500
C	-0.62587900	-3.61706400	-0.74456400
H	-0.37661100	-2.64713700	-0.28210300
C	-1.03045500	-4.58991900	0.36512700
H	-1.36507700	-5.54803400	-0.06470200

H	-1.84018300	-4.19897600	0.99285100
H	-0.17015700	-4.79092800	1.02016300
C	0.63658600	-4.12752200	-1.42641900
H	0.51187200	-5.15582100	-1.80368900
H	1.45900700	-4.14595700	-0.69856100
H	0.93597300	-3.47144900	-2.25647900
C	-2.71170500	2.18234400	1.52687900
C	-3.53156600	3.02113300	0.73926700
C	-3.50402100	4.39574800	0.98900300
H	-4.13503400	5.06069500	0.39705600
C	-2.67861000	4.93298600	1.97089200
H	-2.67175800	6.01055200	2.15053200
C	-1.84925900	4.09742600	2.70831800
H	-1.18693000	4.52681000	3.46383100
C	-1.84424500	2.71427400	2.50290800
C	-4.44128900	2.44946900	-0.33345300
H	-3.95496800	1.53744500	-0.71427300
C	-5.79644100	2.03244600	0.24041900
H	-6.44315400	1.62788900	-0.55397600
H	-6.31380500	2.89401100	0.69241200
H	-5.69473300	1.25424700	1.01020300
C	-4.61241300	3.37940800	-1.52953500
H	-3.63838900	3.69514600	-1.93041100
H	-5.19374300	4.28028100	-1.27685000
H	-5.15365400	2.85985900	-2.33348800
C	-0.90457700	1.84182000	3.31361800
H	-1.18572600	0.79526700	3.13012200
C	-0.99775200	2.10419500	4.81661800
H	-2.03179900	2.05210300	5.19006200
H	-0.60501300	3.09893700	5.07923500
H	-0.39894700	1.36339100	5.36943800
C	0.53498800	2.00553200	2.83364400
H	1.21744000	1.37691800	3.42470000
H	0.87384400	3.04750600	2.93668700
H	0.62780200	1.71459700	1.77936600
C	-0.00005000	0.00011500	-1.90362900

Cartesian coordinates of the optimized geometry of TS-**4** at PBE0-D3BJ/def2-SVP level of theory:

Ga	-1.79575200	0.45744000	0.18867900
Ga	0.81587900	-1.09292900	0.26205500
P	-0.49900900	1.16846200	-1.61487900
P	2.58292500	0.87722800	-0.60133700
O	-0.83828500	-0.88539000	0.96520200
N	-2.19079700	2.10199200	1.27053500
N	-3.65199800	-0.22348900	0.31411700
N	1.70139900	-2.00192400	1.80230300
N	1.24480500	-2.51896000	-1.05329000
N	2.94820100	2.06618600	-3.39977400
C	-3.22347800	2.17882200	2.09852800
C	-4.18935500	1.16603400	2.22467900
H	-4.93517300	1.31836400	3.00355200
C	-4.44273800	0.11379100	1.33573600
C	-3.47211600	3.42249600	2.90994100
H	-3.80245400	3.14577100	3.92098600
H	-4.28806500	4.00025800	2.44982000
H	-2.59252900	4.07199200	2.97546200
C	-5.73240200	-0.63349600	1.54125100
H	-5.53637200	-1.68624100	1.78182000
H	-6.33625500	-0.63589600	0.62281200
H	-6.31645600	-0.18649500	2.35383100
C	-1.43412800	3.27957600	0.98582600
C	-0.19838300	3.48943500	1.63172700
C	0.50360500	4.66303600	1.34683300
H	1.45464300	4.84920400	1.84641500
C	0.01814500	5.59524600	0.43590400

H	0.58349500	6.50819900	0.23142800
C	-1.18394500	5.35710600	-0.21628200
H	-1.55593600	6.08159300	-0.94519000
C	-1.92908100	4.20285900	0.04108200
C	0.34846000	2.49029000	2.63207600
H	0.04798800	1.48725400	2.28380600
C	-0.24323500	2.69712200	4.02813200
H	0.22456200	2.00075400	4.73954100
H	-1.32521200	2.51958400	4.05708300
H	-0.05382600	3.72461600	4.37903400
C	1.86765800	2.50290800	2.71681500
H	2.25012300	3.43723400	3.15862400
H	2.31804400	2.36641800	1.72276500
H	2.20821000	1.68051300	3.35682000
C	-3.22364700	3.98108200	-0.71879200
H	-3.63746800	3.01267400	-0.40038800
C	-4.26379400	5.05688400	-0.40349600
H	-4.45806400	5.13840000	0.67547800
H	-5.21836300	4.83208900	-0.90478800
H	-3.93074300	6.04698900	-0.75330400
C	-2.97357400	3.90418200	-2.22493200
H	-2.59023200	4.86053500	-2.61464100
H	-3.90886300	3.68191300	-2.76112300
H	-2.23447400	3.12626400	-2.46834400
C	-4.12535400	-1.22566300	-0.58509100
C	-3.98488100	-2.59374400	-0.26886300
C	-4.57352200	-3.53102100	-1.12519800
H	-4.48265200	-4.59477100	-0.89127700
C	-5.26513800	-3.13733800	-2.26300500
H	-5.72506700	-3.88558100	-2.91291000
C	-5.34681700	-1.78706500	-2.58930800
H	-5.85937500	-1.48921700	-3.50518300
C	-4.77208400	-0.81045600	-1.77192400
C	-3.20469000	-3.07989400	0.93977100
H	-2.85179200	-2.19573600	1.48939800
C	-4.03299600	-3.95730600	1.87896500
H	-3.43719100	-4.22683900	2.76528600
H	-4.33887300	-4.89730300	1.39226200
H	-4.94778100	-3.46056500	2.23436300
C	-1.95370300	-3.83424300	0.49550200
H	-1.34423300	-4.11588500	1.36728000
H	-1.33078100	-3.20347700	-0.14829700
H	-2.20974900	-4.75587500	-0.05265100
C	-4.83249700	0.66032300	-2.15312400
H	-3.87189600	1.10168500	-1.83678400
C	-5.94516600	1.41403600	-1.42025600
H	-5.79124300	1.43706300	-0.33345100
H	-6.92582700	0.95363300	-1.62189500
H	-5.98891500	2.45871700	-1.76605900
C	-4.96114100	0.88524300	-3.65672100
H	-4.19836200	0.33152800	-4.22182300
H	-4.83931400	1.95368000	-3.88706800
H	-5.95273800	0.58647000	-4.03221600
C	1.79707000	-3.32796400	1.80958100
C	1.55696800	-4.14955100	0.69666200
H	1.63523900	-5.22067900	0.88257900
C	1.40723400	-3.77936600	-0.64258400
C	2.18258200	-4.06439200	3.06655600
H	2.84910400	-4.90219700	2.82341500
H	1.27522200	-4.49021700	3.52119200
H	2.66312700	-3.42103300	3.81159300
C	1.43500400	-4.91750100	-1.63120600
H	2.24209800	-5.62278300	-1.39431500
H	1.54010900	-4.58147400	-2.66808500
H	0.48885200	-5.47321300	-1.53889000
C	2.01422400	-1.28752700	2.99916200
C	3.31862600	-0.76553500	3.14453900
C	3.66266500	-0.15901800	4.35498700

H	4.67092800	0.23714300	4.48977100
C	2.73590200	-0.03881000	5.38544500
H	3.01929100	0.44304600	6.32415900
C	1.44531300	-0.52279600	5.21104200
H	0.71898800	-0.42150000	6.02150000
C	1.05464400	-1.16149100	4.02813300
C	4.33887700	-0.89749000	2.03007700
H	3.77209200	-0.96995000	1.08878900
C	5.15975000	-2.18092000	2.16244800
H	5.71199100	-2.19921200	3.11594200
H	5.89307100	-2.25583500	1.34384600
H	4.52635400	-3.07762200	2.12159900
C	5.24858200	0.31990900	1.90743900
H	5.93798000	0.41328500	2.76145600
H	4.66418500	1.24875800	1.83243600
H	5.86766000	0.23422000	1.00138600
C	-0.34736400	-1.73853700	3.93049300
H	-0.45289800	-2.18942500	2.93437000
C	-0.59514900	-2.80450900	5.00064400
H	0.17860200	-3.58482200	5.00776000
H	-1.56885200	-3.29167800	4.83283400
H	-0.62040400	-2.36197700	6.00955400
C	-1.42063600	-0.65689100	4.02086300
H	-2.41990400	-1.10484600	3.90666200
H	-1.29990900	0.07581800	3.21605100
H	-1.39676300	-0.13544100	4.99136000
C	1.55631300	-2.20143600	-2.41457500
C	2.92007100	-2.07986400	-2.77481500
C	3.23310200	-1.83264400	-4.11323100
H	4.27951800	-1.73995300	-4.41056400
C	2.23711900	-1.68634100	-5.07031200
H	2.50186600	-1.48507000	-6.11086900
C	0.90410900	-1.77457700	-4.69413700
H	0.12604900	-1.64254800	-5.44734300
C	0.53384500	-2.03009900	-3.36933000
C	4.04821300	-2.22547700	-1.76582800
H	3.61163800	-2.05724800	-0.76958300
C	4.66832900	-3.62414000	-1.77744500
H	5.49015900	-3.68487600	-1.04641700
H	5.08189900	-3.85910200	-2.77150700
H	3.94197300	-4.40493200	-1.52400300
C	5.14580500	-1.18366500	-1.96783100
H	5.84874500	-1.20258800	-1.12027300
H	4.72296400	-0.17255300	-2.03595900
H	5.73377600	-1.37648600	-2.87895700
C	-0.93450500	-2.17552900	-3.01978300
H	-1.04910600	-1.88447000	-1.96271600
C	-1.41776000	-3.61918600	-3.16797700
H	-0.89787000	-4.30394300	-2.48940400
H	-1.25971500	-3.97687400	-4.19842000
H	-2.49268400	-3.67962900	-2.94271100
C	-1.83214500	-1.26009700	-3.84288400
H	-2.84831000	-1.28862600	-3.42929600
H	-1.90047500	-1.58807300	-4.89268400
H	-1.47410700	-0.22076000	-3.81684900
C	0.95880500	0.73281900	-0.81290900
C	2.86560600	2.68598200	-2.24308800
N	3.08005700	1.50045600	-4.36697200
Si	4.35826800	3.72691100	-1.70789600
C	4.69847300	5.02263800	-3.02815500
H	3.83815400	5.69927900	-3.14854800
H	5.57703600	5.63253100	-2.76222700
H	4.90016600	4.55397900	-4.00465100
C	3.89853400	4.52973200	-0.08462200
H	3.70530300	3.77032100	0.68671400
H	4.71414900	5.18359100	0.26233400
H	2.98843200	5.13793300	-0.19504800
C	5.83989600	2.59012800	-1.53500100

H	6.05627100	2.05874300	-2.47510300
H	6.73755200	3.16774000	-1.26244100
H	5.66330300	1.83868400	-0.75112800
H	1.83394700	3.01178300	-2.02760300

Cartesian coordinates of the optimized geometry of TS-**5** at PBE0-D3BJ/def2-SVP level of theory:

Ga	-1.75019600	0.26414500	0.29879300
Ga	0.88109500	-1.14283500	0.07232500
P	-0.25503800	1.61835900	-0.92148500
P	2.77957300	1.39002400	-0.61774500
O	-0.83217400	-1.26030400	0.67144000
N	-2.21195600	1.57449100	1.76474900
N	-3.60771600	-0.44376100	0.19591000
N	1.79264000	-2.39964200	1.30541800
N	1.41054200	-2.16297000	-1.56779800
N	1.97936600	3.01837700	-2.70467900
C	-3.24477000	1.39935200	2.57618400
C	-4.17811500	0.36210300	2.41452400
H	-4.92678600	0.27217100	3.20033900
C	-4.40114900	-0.41637000	1.27008300
C	-3.52442800	2.37377700	3.68865000
H	-3.87185200	1.83155500	4.57922800
H	-4.33474200	3.05443200	3.38581700
H	-2.65134000	2.98354000	3.94648800
C	-5.66634700	-1.23169600	1.26558000
H	-5.43351100	-2.30404300	1.24487100
H	-6.26458500	-1.02977700	0.36621700
H	-6.27039800	-1.01807500	2.15477600
C	-1.50865600	2.81772400	1.77772200
C	-0.24746700	2.90900300	2.40493300
C	0.41785600	4.13671600	2.37877100
H	1.39041900	4.22387300	2.86427200
C	-0.13079800	5.24644200	1.74434000
H	0.40626700	6.19856200	1.74119500
C	-1.36022400	5.13480100	1.11027600
H	-1.78200200	6.00078400	0.59386700
C	-2.06565900	3.92798800	1.10779100
C	0.37516200	1.71652600	3.09939400
H	0.09961300	0.82381700	2.51176400
C	-0.17422100	1.53229100	4.51527200
H	0.33291600	0.68916000	5.00697500
H	-1.25060300	1.32001600	4.52176000
H	0.00163000	2.43820800	5.11785500
C	1.89503900	1.77994200	3.14046300
H	2.25598500	2.57716400	3.81050100
H	2.31651400	1.94298800	2.13728300
H	2.29105800	0.83339000	3.52658400
C	-3.36612300	3.84676400	0.33126300
H	-3.75126300	2.82051300	0.42432200
C	-4.43046500	4.79475500	0.88372900
H	-4.61727100	4.62336100	1.95370200
H	-5.38323100	4.66275900	0.34732500
H	-4.12770600	5.84752100	0.76699600
C	-3.12204500	4.11596800	-1.15421900
H	-2.76292000	5.14430900	-1.31764700
H	-4.05318900	3.99711400	-1.72830100
H	-2.36378000	3.43299100	-1.56557000
C	-4.05412900	-1.19755500	-0.93262300
C	-3.87285600	-2.59717200	-0.98027500
C	-4.42988900	-3.29752500	-2.05662800
H	-4.30569400	-4.38230600	-2.10342700
C	-5.12730800	-2.64551100	-3.06392800
H	-5.55840300	-3.21380800	-3.89154700
C	-5.25405900	-1.26050300	-3.02872100
H	-5.77621600	-0.75179800	-3.84018500

C	-4.71899100	-0.51250700	-1.97695600
C	-3.08331700	-3.36642400	0.06399900
H	-2.74210400	-2.65070200	0.82496900
C	-3.89395800	-4.47512700	0.73666400
H	-3.29186500	-4.95474300	1.52445800
H	-4.18078400	-5.26161200	0.02015300
H	-4.81893300	-4.10836200	1.20554300
C	-1.81957800	-3.95688400	-0.55795800
H	-1.20510000	-4.44631400	0.21233100
H	-1.20718500	-3.16689700	-1.00644400
H	-2.05983600	-4.70678900	-1.32996600
C	-4.84734500	1.00261700	-1.96008000
H	-3.90392900	1.38962200	-1.53873400
C	-5.98289000	1.48752300	-1.05507600
H	-5.81462600	1.23638500	0.00020800
H	-6.94421000	1.04855800	-1.36712100
H	-6.07952100	2.58274700	-1.11889300
C	-5.00848000	1.60530100	-3.35274800
H	-4.23603900	1.24861000	-4.04886800
H	-4.93216900	2.70109700	-3.29926400
H	-5.99405400	1.37325500	-3.78647600
C	1.92772400	-3.68134400	0.97232700
C	1.70428800	-4.19516300	-0.31499700
H	1.79102300	-5.27675000	-0.41218000
C	1.55600800	-3.48598500	-1.51341600
C	2.34946300	-4.67962800	2.01610900
H	2.73325000	-5.59256100	1.54561600
H	1.48695800	-4.95170400	2.64111600
H	3.11181600	-4.26780700	2.69005500
C	1.55391200	-4.31315000	-2.77247100
H	2.27837100	-5.13537400	-2.71164000
H	1.75163400	-3.71652600	-3.67013200
H	0.55613500	-4.76895900	-2.87511300
C	2.09612900	-2.00729600	2.64258000
C	3.36646200	-1.44451600	2.89828200
C	3.70955800	-1.14866700	4.21952700
H	4.69181300	-0.72586500	4.43790600
C	2.80888800	-1.36256000	5.25835000
H	3.09070700	-1.12064400	6.28587500
C	1.54337900	-1.86490100	4.98204500
H	0.83307600	-2.01170800	5.79971200
C	1.15678400	-2.20157800	3.67931900
C	4.33929600	-1.18316700	1.76334400
H	3.73213300	-0.96027700	0.87054000
C	5.18010100	-2.42082800	1.44442200
H	5.74318800	-2.74944200	2.33277500
H	5.90627100	-2.19916800	0.64734500
H	4.56218100	-3.26195700	1.10115900
C	5.23226700	0.03000500	1.99943300
H	5.97699700	-0.15019500	2.79123800
H	4.64066700	0.91562000	2.27256700
H	5.78306400	0.26909400	1.07744400
C	-0.23401700	-2.76687400	3.44217400
H	-0.31956100	-3.00764800	2.37292100
C	-0.49790900	-4.02743900	4.26904400
H	0.27980900	-4.79478800	4.14243300
H	-1.46282800	-4.47373600	3.98116400
H	-0.55305200	-3.79810800	5.34530000
C	-1.31805500	-1.73050900	3.73149000
H	-2.31179800	-2.14794800	3.50706900
H	-1.18882900	-0.84732700	3.09715700
H	-1.31218600	-1.42178900	4.78932600
C	1.72930500	-1.46522100	-2.77350100
C	3.09412000	-1.23092700	-3.07307900
C	3.40807200	-0.56120500	-4.25901000
H	4.45492300	-0.37877800	-4.50840100
C	2.41408600	-0.11076700	-5.11894100
H	2.68038300	0.41383900	-6.03948600

C	1.08011100	-0.30980900	-4.78973800
H	0.30189300	0.06507500	-5.45654300
C	0.71110000	-0.97868100	-3.61801400
C	4.21734900	-1.68661400	-2.15450900
H	3.78950200	-1.75968700	-1.14222800
C	4.75813700	-3.06755000	-2.53305000
H	5.58444100	-3.35046200	-1.86205500
H	5.14669100	-3.06161900	-3.56430900
H	3.99624600	-3.85188800	-2.46521600
C	5.37622000	-0.69356900	-2.09986600
H	6.08124300	-0.98200800	-1.30505300
H	5.01418200	0.32111100	-1.88629100
H	5.94757400	-0.67795900	-3.04140100
C	-0.75723500	-1.19692800	-3.31568400
H	-0.85470200	-1.30812800	-2.22275200
C	-1.28558100	-2.47734500	-3.96358100
H	-0.74858000	-3.36749200	-3.61617800
H	-1.17966200	-2.42640500	-5.05942400
H	-2.35079100	-2.61215500	-3.72501600
C	-1.62764700	-0.01853500	-3.73528500
H	-2.64005200	-0.15897600	-3.33615400
H	-1.71906000	0.05477300	-4.83092300
H	-1.23029600	0.93351900	-3.35200600
C	1.16290500	0.76458400	-0.39574600
C	2.37994100	3.07090300	-1.21198600
N	2.26999200	2.86072100	-3.77548900
Si	3.82288500	4.32065500	-1.03196300
C	3.37289500	5.88302600	-1.97434100
H	2.43505900	6.31532700	-1.59113200
H	4.16463600	6.64105500	-1.86266700
H	3.24677500	5.69247100	-3.05161500
C	3.98140000	4.67600200	0.79974900
H	4.24305400	3.76052200	1.35067700
H	4.76031500	5.43372700	0.98202400
H	3.02898700	5.05490800	1.20017900
C	5.36022200	3.52439100	-1.74916700
H	5.19479900	3.20891400	-2.79191800
H	6.20857000	4.22703800	-1.73923300
H	5.64086400	2.63295100	-1.16781200
H	1.43782000	3.42614200	-0.74250800

Cartesian coordinates of the optimized geometry of TS-**6** at PBE0-D3BJ/def2-SVP level of theory:

Ga	-1.73501900	0.31579100	0.31004100
Ga	0.86087200	-1.15232600	0.07119600
P	-0.30884600	1.51886300	-1.09671100
P	2.75887500	1.31240200	-0.59685300
O	-0.82352500	-1.19500600	0.75287700
N	-2.11862600	1.74090700	1.69689900
N	-3.60581800	-0.35483100	0.32739200
N	1.78730400	-2.34484900	1.36318600
N	1.32858200	-2.28333300	-1.51159200
N	1.94967900	2.85356900	-2.64424200
C	-3.13985400	1.66562000	2.53667400
C	-4.10272700	0.64330200	2.48310900
H	-4.83482400	0.63959900	3.28950300
C	-4.37034400	-0.22318600	1.41434500
C	-3.37356600	2.73817300	3.56683200
H	-3.68383300	2.28034000	4.51672700
H	-4.19748100	3.38898900	3.23664200
H	-2.49069200	3.36547600	3.73295100
C	-5.65030500	-1.00954000	1.50616200
H	-5.43887900	-2.08570300	1.54871800
H	-6.27449500	-0.85440300	0.61501400
H	-6.22000500	-0.72462500	2.39808300
C	-1.36366700	2.94896200	1.59723900

C	-0.08486500	3.02949900	2.19046700
C	0.64696200	4.20775300	2.02596500
H	1.63393100	4.28621500	2.48257200
C	0.14447000	5.28020800	1.29512200
H	0.73431800	6.19429700	1.18540300
C	-1.11076300	5.18255900	0.71109500
H	-1.50108900	6.01948600	0.12653300
C	-1.88154300	4.02391400	0.84354400
C	0.48710600	1.87648300	2.98917700
H	0.20988300	0.95106900	2.45414400
C	-0.10805300	1.80115500	4.39702600
H	0.37630400	0.99155700	4.96268000
H	-1.18542300	1.59726600	4.38935800
H	0.06113700	2.74628400	4.93815400
C	2.00501900	1.90980900	3.08452800
H	2.35875500	2.74952300	3.70503000
H	2.46612600	1.98361900	2.08825400
H	2.36271600	0.98778200	3.55747000
C	-3.21447200	3.95087400	0.12299900
H	-3.64199200	2.95384700	0.30559500
C	-4.20856700	4.98718500	0.64799400
H	-4.35383000	4.90396700	1.73466800
H	-5.18967600	4.86378400	0.16289100
H	-3.86250000	6.01226700	0.44008000
C	-3.02706300	4.10245800	-1.38695200
H	-2.64050500	5.10208000	-1.64147400
H	-3.98722200	3.97894200	-1.91009900
H	-2.31267300	3.36248900	-1.77767900
C	-4.09997400	-1.17358100	-0.73331900
C	-3.94371500	-2.57616400	-0.69380000
C	-4.54626500	-3.33461900	-1.70432300
H	-4.44231900	-4.42238700	-1.68339300
C	-5.26468800	-2.73633900	-2.73017900
H	-5.73242200	-3.34922100	-3.50439700
C	-5.36460700	-1.34963200	-2.78398100
H	-5.90084900	-0.88505900	-3.61261700
C	-4.78106000	-0.54483100	-1.80232100
C	-3.13508200	-3.28879800	0.37580900
H	-2.75373300	-2.52877800	1.07199600
C	-3.94731500	-4.32624400	1.15208700
H	-3.32924300	-4.76645400	1.95049400
H	-4.27931900	-5.15213300	0.50251700
H	-4.84520600	-3.90240800	1.62554800
C	-1.90560800	-3.95174000	-0.24230400
H	-1.27720100	-4.40125900	0.54116900
H	-1.29108100	-3.21017100	-0.76466000
H	-2.18690500	-4.74785400	-0.95151200
C	-4.86737500	0.97101700	-1.88888700
H	-3.89999100	1.35704200	-1.52458800
C	-5.95989200	1.55385100	-0.98873600
H	-5.76884500	1.37017400	0.07654000
H	-6.94331400	1.12683700	-1.24341800
H	-6.02403300	2.64484500	-1.12561300
C	-5.05352700	1.47905300	-3.31573500
H	-4.31046500	1.05365700	-4.00503900
H	-4.94741000	2.57336800	-3.34138000
H	-6.05696500	1.24473900	-3.70516400
C	1.90153800	-3.64630300	1.10593100
C	1.64879100	-4.23557600	-0.14253100
H	1.73078100	-5.32132100	-0.17635200
C	1.47907700	-3.59995400	-1.37885300
C	2.33712600	-4.58344400	2.19977500
H	2.66069700	-5.54355200	1.78101400
H	1.50126100	-4.77083000	2.88806900
H	3.14986600	-4.15576500	2.80130300
C	1.46820500	-4.50575200	-2.58317100
H	2.23359400	-5.28799400	-2.49827700
H	1.60526600	-3.96186700	-3.52431200

H	0.49187700	-5.01464300	-2.61635000
C	2.12180800	-1.88730300	2.67252200
C	3.41098800	-1.34704600	2.87880100
C	3.78881200	-1.00326500	4.17878600
H	4.78616900	-0.59850000	4.35955300
C	2.90411300	-1.14564300	5.24327400
H	3.21304300	-0.86616400	6.25333700
C	1.62013800	-1.62367000	5.01350200
H	0.92271400	-1.71420100	5.85021000
C	1.19905800	-2.00999200	3.73534100
C	4.36699800	-1.15772100	1.71544800
H	3.74679800	-0.95313900	0.82710500
C	5.17255800	-2.42504800	1.42347000
H	5.74316400	-2.73956200	2.31220800
H	5.89027700	-2.24511500	0.60818000
H	4.52982600	-3.26105200	1.11539700
C	5.29348700	0.04091100	1.88807900
H	6.05437200	-0.13116600	2.66620600
H	4.72870100	0.94765800	2.14854800
H	5.82554800	0.23733900	0.94514400
C	-0.20851600	-2.55524000	3.55478500
H	-0.32342200	-2.84086900	2.49956000
C	-0.48172600	-3.77057100	4.44418700
H	0.27135600	-4.56444200	4.33341700
H	-1.46479000	-4.20333500	4.20036800
H	-0.50337800	-3.49255100	5.51013000
C	-1.26436400	-1.48433800	3.82148600
H	-2.27124300	-1.89065000	3.63901900
H	-1.13249900	-0.63342300	3.14526100
H	-1.22803600	-1.12892600	4.86394400
C	1.61272100	-1.66729500	-2.77007800
C	2.96736500	-1.44681600	-3.11807400
C	3.24825500	-0.87119200	-4.36017200
H	4.28780400	-0.70194800	-4.64769700
C	2.23011500	-0.50209700	-5.23069800
H	2.46960300	-0.05492000	-6.19871000
C	0.90568000	-0.68889200	-4.85739700
H	0.10902200	-0.37496600	-5.53353200
C	0.56929200	-1.26242500	-3.62740300
C	4.11485900	-1.82243100	-2.19366800
H	3.70674100	-1.84246300	-1.17101700
C	4.67831400	-3.21216200	-2.49924200
H	5.51938300	-3.44034100	-1.82575400
H	5.05163600	-3.25971900	-3.53505200
H	3.93208600	-4.00480700	-2.37471200
C	5.25047700	-0.80204900	-2.21944900
H	5.97621100	-1.02640200	-1.42238200
H	4.86438400	0.21324600	-2.05450600
H	5.80351600	-0.82619500	-3.17172400
C	-0.88894600	-1.46634200	-3.26972700
H	-0.95760300	-1.46373000	-2.16915500
C	-1.41762700	-2.81194900	-3.76796200
H	-0.87031200	-3.65758500	-3.33651000
H	-1.33209900	-2.87819700	-4.86482000
H	-2.47765600	-2.92511900	-3.49659400
C	-1.78536100	-0.34660600	-3.78360800
H	-2.78743900	-0.46325200	-3.35171300
H	-1.89902900	-0.38156200	-4.87921400
H	-1.39482300	0.64108900	-3.49815400
C	1.14160100	0.73409800	-0.51373700
C	2.44244800	3.05044200	-1.35122000
N	1.33313700	2.46882600	-3.49672300
Si	3.95248100	4.23012800	-1.25348100
C	3.44380300	5.88420700	-1.98123300
H	2.58322100	6.30153500	-1.43493500
H	4.26960600	6.61091700	-1.92084400
H	3.16039300	5.78869600	-3.04140400
C	4.35534900	4.37026300	0.56633600

H	4.58804900	3.38208900	0.99086600
H	5.22439900	5.02997300	0.71731500
H	3.50324900	4.78782500	1.12308100
C	5.34916600	3.45098700	-2.22982700
H	5.05701700	3.27321700	-3.27697200
H	6.23818900	4.10135100	-2.23352400
H	5.63210400	2.48272800	-1.78926400
H	1.53785500	3.44143000	-0.81694200

Cartesian coordinates of the optimized geometry of TS-**7** at PBE0-D3BJ/def2-SVP level of theory:

Ga	-1.77703700	0.02804000	0.20401000
Ga	1.10283300	-1.00644500	0.11407800
P	-0.40410900	1.42473500	-1.17546000
P	2.51300700	1.87835000	-0.52955200
O	-0.63583400	-1.33570300	0.55019000
N	-2.62446900	1.11046600	1.63547900
N	-3.42865800	-0.99684400	-0.20951800
N	2.02591800	-2.11642700	1.47072300
N	2.03369500	-1.91838200	-1.42482900
C	-3.67408500	0.64894900	2.31582200
C	-4.38868800	-0.50076400	1.95770200
H	-5.18684000	-0.79284900	2.63809900
C	-4.34630800	-1.19201600	0.73661700
C	-4.20849400	1.42604100	3.48713400
H	-4.71023000	0.74849600	4.19037400
H	-4.95225700	2.15922800	3.13993900
H	-3.42358000	1.98789200	4.00643800
C	-5.44634400	-2.19153300	0.50530000
H	-5.04585400	-3.21315100	0.54545400
H	-5.89322100	-2.07279100	-0.49088000
H	-6.22767400	-2.09246200	1.26750900
C	-2.24778400	2.47312300	1.85341000
C	-1.10252100	2.78323500	2.61033800
C	-0.80307900	4.12884800	2.83959000
H	0.08012200	4.38574700	3.42648700
C	-1.60033500	5.14399100	2.32687100
H	-1.34744600	6.18947200	2.51777100
C	-2.70337500	4.82262800	1.54739900
H	-3.30644100	5.62323800	1.11197400
C	-3.04059700	3.49322900	1.28590200
C	-0.21967800	1.70302900	3.19660800
H	-0.33244300	0.80364000	2.56854200
C	-0.66391300	1.34012900	4.61423800
H	0.00474400	0.57855100	5.03969800
H	-1.68537600	0.93658200	4.63601200
H	-0.63421300	2.22923900	5.26470700
C	1.25604300	2.07997800	3.18058400
H	1.48390700	2.88844100	3.89319900
H	1.58090500	2.39608300	2.17878800
H	1.85855700	1.21191200	3.47498700
C	-4.18384000	3.20325500	0.33101900
H	-4.35215700	2.11623200	0.31543200
C	-5.49508000	3.86375800	0.75434600
H	-5.78435500	3.58424100	1.77834700
H	-6.31241200	3.56400600	0.08004200
H	-5.42607900	4.96227200	0.71909400
C	-3.79718000	3.63045400	-1.08651200
H	-3.58102700	4.70926100	-1.12790300
H	-4.61583400	3.42470900	-1.79256300
H	-2.89674500	3.10497500	-1.43632100
C	-3.56693500	-1.72920200	-1.42824000
C	-3.14611700	-3.07508100	-1.50947600
C	-3.41135600	-3.77401500	-2.69270500
H	-3.09952900	-4.81847000	-2.77157900
C	-4.05993100	-3.17226900	-3.76292000

H	-4.26406700	-3.74202200	-4.67250600
C	-4.42970400	-1.83381300	-3.68111000
H	-4.91282400	-1.35852700	-4.53618700
C	-4.18176000	-1.08688400	-2.52672800
C	-2.41440400	-3.78699700	-0.38441000
H	-2.32546500	-3.08474500	0.45631900
C	-3.12871500	-5.05574000	0.08519200
H	-2.59937100	-5.48702600	0.94932100
H	-3.14681200	-5.82486200	-0.70344700
H	-4.17074200	-4.87894300	0.38913900
C	-0.98714900	-4.13304800	-0.80337800
H	-0.43609400	-4.57414200	0.04082100
H	-0.44515800	-3.23285900	-1.11128400
H	-0.97299000	-4.85857900	-1.63321900
C	-4.57686600	0.37787700	-2.45093300
H	-3.82724500	0.87307400	-1.81207000
C	-5.94121600	0.57041000	-1.78413700
H	-5.94370900	0.23885500	-0.73716600
H	-6.72097200	0.01000600	-2.32448700
H	-6.22649100	1.63387200	-1.79181500
C	-4.55118400	1.07648400	-3.80697200
H	-3.59744600	0.91814300	-4.32988500
H	-4.68604800	2.15980000	-3.67423900
H	-5.36475100	0.72953100	-4.46334500
C	2.43121800	-3.35253000	1.17626800
C	2.50739300	-3.87922000	-0.12085900
H	2.81500000	-4.92199200	-0.19082900
C	2.44163100	-3.18046000	-1.33339800
C	2.86208000	-4.27448800	2.28484800
H	3.42039700	-5.12780300	1.88265200
H	1.98151100	-4.65815100	2.81818300
H	3.47860800	-3.75485600	3.02982700
C	2.87853800	-3.94769800	-2.55293400
H	3.85403800	-4.42296100	-2.38422800
H	2.93027900	-3.32085600	-3.44991000
H	2.15346800	-4.75759400	-2.73036800
C	2.08794700	-1.70222600	2.83581300
C	3.20555400	-0.94307200	3.24769400
C	3.33369700	-0.62856500	4.60305800
H	4.19648000	-0.05177700	4.94096600
C	2.37516600	-1.03244600	5.52616300
H	2.49220400	-0.78095700	6.58293800
C	1.25931100	-1.74012500	5.09677100
H	0.49882400	-2.03654000	5.82359500
C	1.08401700	-2.08661500	3.75238900
C	4.26981200	-0.51732600	2.25218000
H	3.77628400	-0.42088300	1.27125400
C	5.35186400	-1.59171800	2.11884300
H	5.80941600	-1.80651200	3.09796400
H	6.15001700	-1.25590800	1.44029300
H	4.95099200	-2.53276000	1.71713200
C	4.88991200	0.83832900	2.57384100
H	5.50852200	0.80571900	3.48475800
H	4.12055800	1.61270900	2.70226300
H	5.54357300	1.15359600	1.74752600
C	-0.16355600	-2.85279900	3.34484300
H	-0.08744900	-3.06832400	2.26950900
C	-0.33176100	-4.16425100	4.11517300
H	0.55417700	-4.81352200	4.06198800
H	-1.18735900	-4.73041000	3.71456300
H	-0.53403200	-3.98069700	5.18245500
C	-1.41638400	-1.99760200	3.52361700
H	-2.30300500	-2.53534900	3.15422000
H	-1.33684600	-1.06818400	2.95066400
H	-1.58816300	-1.74987700	4.58359600
C	2.40090800	-1.12092900	-2.55309900
C	3.73620100	-0.66966500	-2.67628800
C	4.05001800	0.19970700	-3.72610500

H	5.07531400	0.56158100	-3.82949600
C	3.08360800	0.62992000	-4.62264400
H	3.34660800	1.32442100	-5.42409900
C	1.77824700	0.16910000	-4.49696500
H	1.02361300	0.50480700	-5.20870300
C	1.41432400	-0.71522400	-3.47911600
C	4.85601800	-1.09231400	-1.73783800
H	4.40507600	-1.61987800	-0.88332500
C	5.82379700	-2.05404600	-2.43406700
H	6.57874500	-2.42552700	-1.72318700
H	6.35762300	-1.54091600	-3.25001600
H	5.31227800	-2.92002900	-2.87278500
C	5.63888100	0.09835000	-1.18461000
H	6.41264800	-0.24830600	-0.48300100
H	4.98057700	0.80590200	-0.66188800
H	6.15577900	0.64876600	-1.98606700
C	0.00195000	-1.26231400	-3.41595300
H	-0.28466600	-1.32794100	-2.35036200
C	-0.06723300	-2.67795000	-3.99197900
H	0.56041600	-3.38183000	-3.43500800
H	0.26621800	-2.67973200	-5.04217000
H	-1.10121800	-3.05052400	-3.95549100
C	-1.02721800	-0.38690700	-4.11790300
H	-2.03204200	-0.77813100	-3.91514300
H	-0.89041300	-0.39731700	-5.21109900
H	-0.98343600	0.65603900	-3.77044500
C	1.10580300	0.87657500	-0.48906600
C	2.01458200	3.28068200	-1.44306400
Si	3.18352600	4.73675600	-1.62742000
C	2.26671000	6.16011500	-2.44638700
H	1.38306000	6.43631000	-1.85060300
H	2.90991000	7.04922600	-2.54341300
H	1.92292000	5.87915900	-3.45463700
C	3.76059300	5.22228100	0.09494400
H	4.27398500	4.37814100	0.58241700
H	4.45229100	6.07916700	0.07006700
H	2.89302600	5.49296400	0.71727300
C	4.65096100	4.21025200	-2.68730500
H	4.31138500	3.85901000	-3.67456900
H	5.36017600	5.03962900	-2.84113400
H	5.18928400	3.37864800	-2.20560800
N	0.43344200	4.04779400	-0.47930200
N	-0.45912800	3.32573100	-0.39658900
H	1.32359400	3.05988600	-2.27759600

Cartesian coordinates of the optimized geometry of TS-**8** at PBE0-D3BJ/def2-SVP level of theory:

Ga	-1.55794800	-0.26203800	0.34108600
Ga	1.46962200	-0.64033100	0.03720000
P	-0.70749000	1.16186700	-1.39971300
P	1.72173200	2.52853100	-1.32699400
O	-0.08103400	-1.22942900	0.78868000
N	-2.46837700	0.95029200	1.64413100
N	-2.93309900	-1.70594600	0.53847700
N	2.78979000	-1.13875700	1.42397500
N	2.38871100	-1.67596000	-1.39856700
C	-3.28712900	0.49766400	2.59385200
C	-3.74961500	-0.82086100	2.64543300
H	-4.37306700	-1.07260000	3.50118400
C	-3.66241700	-1.80822600	1.64693100
C	-3.78515700	1.44413300	3.64897300
H	-4.47951600	0.94245000	4.33286800
H	-4.29038400	2.30882100	3.19501100
H	-2.94118800	1.84635800	4.22751800
C	-4.50440700	-3.03624800	1.86644800
H	-3.86996400	-3.91975700	2.01141600

H	-5.12861700	-3.24885600	0.98774700
H	-5.14789000	-2.91519200	2.74530500
C	-2.38629700	2.36321300	1.44207700
C	-1.44737100	3.15032000	2.13796800
C	-1.44341300	4.53271600	1.92448300
H	-0.71562000	5.15030100	2.45595000
C	-2.34440900	5.13197600	1.05392800
H	-2.33510400	6.21493400	0.91094400
C	-3.25897600	4.34409400	0.36534800
H	-3.97081100	4.81584100	-0.31637200
C	-3.29711800	2.95854200	0.53957700
C	-0.43379800	2.54581700	3.08328700
H	-0.67877800	1.48041000	3.19676200
C	-0.45729000	3.18199300	4.47234800
H	0.23101500	2.64301500	5.14153100
H	-1.46059200	3.16381500	4.92513200
H	-0.12887500	4.23289600	4.44207100
C	0.96225700	2.63162600	2.47891200
H	1.25296400	3.67911900	2.29755900
H	1.01150500	2.10752900	1.51477900
H	1.70091500	2.18411600	3.15416300
C	-4.34521200	2.14889500	-0.19876200
H	-4.12344300	1.08595500	-0.02431100
C	-5.74024800	2.42085200	0.36664200
H	-5.79793000	2.17544400	1.43741900
H	-6.49828600	1.81766100	-0.15587000
H	-6.01545400	3.48123200	0.25007900
C	-4.29988200	2.38791700	-1.70584100
H	-4.54941600	3.42865000	-1.96445400
H	-5.02848800	1.74188000	-2.21831900
H	-3.30106000	2.17299400	-2.11491800
C	-3.01008700	-2.78601900	-0.39496800
C	-2.26125400	-3.96588000	-0.18818500
C	-2.47165900	-5.03881000	-1.06221100
H	-1.90702300	-5.96228600	-0.91137000
C	-3.37466900	-4.95436400	-2.11299300
H	-3.52807100	-5.80858800	-2.77664000
C	-4.06482300	-3.76709000	-2.33400300
H	-4.74643700	-3.69541800	-3.18265000
C	-3.89023700	-2.66347700	-1.49509100
C	-1.22996800	-4.11508000	0.91755800
H	-1.20367400	-3.17273800	1.48170800
C	-1.53035200	-5.27269000	1.87093600
H	-0.77668400	-5.30212200	2.67363600
H	-1.49503900	-6.24496100	1.35364100
H	-2.51692600	-5.19331300	2.35029200
C	0.16599000	-4.29134900	0.32250700
H	0.92322400	-4.31090800	1.12075500
H	0.41165900	-3.45399600	-0.33945000
H	0.25085700	-5.23197500	-0.24628100
C	-4.62790600	-1.36195800	-1.76517500
H	-3.93009800	-0.54882800	-1.50051500
C	-5.87409700	-1.20293000	-0.89093100
H	-5.63113300	-1.11648400	0.17629700
H	-6.55379100	-2.05984900	-1.02405800
H	-6.42533700	-0.29368700	-1.17573100
C	-5.00377000	-1.17653500	-3.23256300
H	-4.14435700	-1.32537600	-3.90131700
H	-5.38370100	-0.15725400	-3.39573000
H	-5.80278000	-1.86925700	-3.54074000
C	3.47334400	-2.27996800	1.33035300
C	3.53518200	-3.07882800	0.17904700
H	4.10007600	-4.00535500	0.27528700
C	3.12965200	-2.74396600	-1.12070000
C	4.25767100	-2.76580200	2.51849100
H	4.92698300	-3.58585600	2.23408600
H	3.57584800	-3.12673200	3.30079700
H	4.84684300	-1.95569700	2.96911200

C	3.59308600	-3.66931400	-2.21403500
H	4.67054300	-3.86465000	-2.12975500
H	3.37140300	-3.28300400	-3.21497600
H	3.08032600	-4.63613800	-2.08886400
C	2.89478400	-0.38663700	2.63276200
C	3.81086000	0.68735500	2.66944200
C	3.99879000	1.36308600	3.87743400
H	4.71538800	2.18495500	3.92723600
C	3.26859300	1.02103800	5.01107700
H	3.42326000	1.56304900	5.94707600
C	2.32488700	0.00368900	4.94093800
H	1.73500300	-0.24390900	5.82710500
C	2.11817900	-0.72358500	3.76283600
C	4.58715400	1.09446100	1.43090400
H	3.99056600	0.77751500	0.56054800
C	5.92919000	0.36431000	1.35320100
H	6.53827500	0.57099900	2.24792800
H	6.50142600	0.69267000	0.47230200
H	5.79837900	-0.72442600	1.27549000
C	4.77248000	2.60456100	1.31927700
H	5.45177800	2.99771600	2.09211900
H	3.81049200	3.13121000	1.40302200
H	5.20750800	2.85899300	0.34201100
C	1.07615400	-1.82893700	3.75348100
H	1.10050200	-2.30314100	2.76174300
C	1.33259600	-2.88923600	4.82629800
H	2.34707500	-3.31181000	4.77801600
H	0.61765600	-3.71967900	4.71563400
H	1.20077300	-2.47804200	5.83981700
C	-0.32953200	-1.25562800	3.91876800
H	-1.08073700	-2.05970100	3.88841100
H	-0.55398200	-0.56972600	3.09500600
H	-0.44222600	-0.72334300	4.87750600
C	2.37185200	-1.13295900	-2.72002900
C	3.49906100	-0.41626400	-3.18066600
C	3.45336300	0.14150100	-4.46200500
H	4.31845600	0.69578000	-4.83310600
C	2.32552500	0.02166000	-5.26122600
H	2.30592700	0.47306700	-6.25599300
C	1.21524200	-0.66526000	-4.78411000
H	0.32592600	-0.74399300	-5.41053500
C	1.21426600	-1.25222800	-3.51704400
C	4.75426700	-0.22267200	-2.34668500
H	4.53729000	-0.57078100	-1.32535200
C	5.92236900	-1.05177100	-2.88525600
H	6.80315000	-0.95002300	-2.23163000
H	6.21138900	-0.70939100	-3.89193200
H	5.67555500	-2.11891200	-2.95979200
C	5.15774500	1.24860400	-2.25771600
H	6.02345300	1.36745100	-1.58856000
H	4.33417500	1.87311800	-1.88334000
H	5.45394900	1.64535800	-3.24123300
C	0.00764800	-2.03157600	-3.03735900
H	-0.06734100	-1.88866300	-1.94455400
C	0.17713200	-3.53068700	-3.28821400
H	1.05362800	-3.93752600	-2.76989700
H	0.29801400	-3.72789300	-4.36563500
H	-0.70774800	-4.07825400	-2.93211300
C	-1.30163100	-1.55189200	-3.64792800
H	-2.13685600	-2.05754300	-3.14854600
H	-1.36899100	-1.79716000	-4.71997100
H	-1.42934500	-0.46473300	-3.53084300
C	0.93964200	1.05777400	-0.81787700
C	0.25459100	3.48864500	-1.30747200
H	-0.36324600	3.40741000	-0.40218900
Si	0.00522500	4.98506100	-2.41272100
C	1.25349100	4.89672700	-3.81859700
H	2.28559500	4.90993400	-3.43333300

H	1.12617000	3.96417000	-4.39103000
H	1.13679500	5.74639000	-4.51028500
C	0.26887900	6.57440900	-1.43011300
H	1.29475100	6.61787500	-1.03212800
H	0.10771300	7.46386800	-2.06069400
H	-0.42567500	6.62606900	-0.57817500
C	-1.74265100	4.95261400	-3.10911900
H	-1.91200700	4.02376000	-3.67496000
H	-2.48261500	4.99212300	-2.29648400
H	-1.91672200	5.80845100	-3.78096600

Cartesian coordinates of the optimized geometry of TS-**9** at PBE0-D3BJ/def2-SVP level of theory:

Ga	1.74381300	-0.17841800	-0.39853000
Ga	-1.79204100	-0.14775400	0.30530300
P	-0.09253000	-1.33595900	-0.85175600
P	-0.72046300	-0.60066700	2.29296000
O	1.42313200	-2.14284600	1.50094200
C	0.41396900	-1.51237200	1.24689800
N	2.44090100	1.38082900	0.46458500
N	3.42400000	-0.98888000	-0.88533300
N	-2.43600300	1.53123100	-0.47861600
N	-3.49666100	-1.11272800	0.07181400
C	3.74221400	1.69103800	0.42695500
C	4.73225500	0.89313900	-0.15672300
H	5.74697000	1.28450600	-0.10816100
C	4.59481000	-0.39053700	-0.71476700
C	4.18998600	2.96862100	1.07653800
H	5.25456800	3.14989700	0.89056100
H	4.03296800	2.92598600	2.16591000
H	3.61343300	3.82873500	0.70425800
C	5.84611000	-1.11246200	-1.11860000
H	6.73507900	-0.49620800	-0.94280100
H	5.81424500	-1.39140800	-2.18321900
H	5.95014600	-2.04825900	-0.54756300
C	-3.71922500	1.77473800	-0.73609100
C	-4.74467000	0.82855000	-0.61237800
H	-5.74769500	1.17810700	-0.85198800
C	-4.63085200	-0.53781800	-0.27864700
C	-4.11429000	3.14537900	-1.21592500
H	-3.68831200	3.35270500	-2.21082200
H	-3.74235300	3.92788800	-0.53676200
H	-5.20423700	3.23527100	-1.28924600
C	-5.88467900	-1.36539700	-0.33525400
H	-5.76541600	-2.20767700	-1.03442600
H	-6.74384400	-0.76492200	-0.65585500
H	-6.11113100	-1.80049700	0.65099900
C	1.52365700	2.22337300	1.21645000
H	1.90061300	2.41986800	2.23110400
H	1.34651100	3.19027800	0.71595500
H	0.57041100	1.68997500	1.34421600
C	3.35220600	-2.35031500	-1.38493100
H	2.29485100	-2.62528400	-1.50649200
H	3.85278600	-2.45602400	-2.36077500
H	3.80354300	-3.06050600	-0.67386400
C	-1.43657500	2.53864000	-0.74295800
H	-1.38996900	3.31010800	0.04720800
H	-0.45203900	2.05197000	-0.79391600
H	-1.59229800	3.04812300	-1.70685500
C	-3.44193800	-2.52560700	0.37916500
H	-4.20188100	-2.82274500	1.12017800
H	-3.57748400	-3.14816600	-0.52233700
H	-2.45808100	-2.75520100	0.81357600

Cartesian coordinates of the optimized geometry of TS-**10** at PBE0-D3BJ/def2-SVP level of theory:

Ga	2.46764500	-0.16666800	0.56490600
Ga	-2.62494000	-0.61962400	-0.04744200
P	0.85305200	-0.92729800	2.01343000
P	-1.20372800	-2.24529000	-0.03827500
O	1.00910300	-0.76364100	-0.64382100
N	4.01124400	-1.16364500	-0.06132200
N	3.11630200	1.59794100	0.08805200
N	-2.70590600	1.28160000	0.25325100
N	-4.50073200	-0.88566200	-0.44535700
C	5.00453900	-0.61289800	-0.74097700
C	5.10315300	0.76788200	-1.00519600
H	5.97499900	1.07942500	-1.57827600
C	4.23273200	1.79394600	-0.60978400
C	6.09896900	-1.49694300	-1.26708100
H	6.85000000	-0.91803800	-1.81666400
H	5.68754200	-2.26632900	-1.93893300
H	6.60136300	-2.02925600	-0.44416700
C	4.60071100	3.19836000	-0.99765900
H	4.75786300	3.82358400	-0.10441400
H	3.79295300	3.66939500	-1.57920100
H	5.51826100	3.21680600	-1.59676700
C	-3.80901700	1.99916900	0.05627000
C	-5.04815800	1.45613600	-0.31939000
H	-5.86215300	2.16894700	-0.44044200
C	-5.37696500	0.10624100	-0.53563900
C	-3.74432400	3.48713000	0.25274100
H	-2.94960500	3.93172500	-0.36564700
H	-3.51012200	3.73367500	1.30044800
H	-4.69795500	3.96036400	-0.00724100
C	-6.80090500	-0.21423100	-0.88747600
H	-7.24334700	-0.89470000	-0.14312700
H	-6.85846600	-0.72682800	-1.86043500
H	-7.41248600	0.69391000	-0.93489700
C	0.28674900	-1.19847500	0.35016000
C	-1.46809900	1.92107100	0.66411900
H	-0.99780600	2.47052000	-0.16981600
H	-1.62494900	2.62341200	1.49732800
H	-0.76079700	1.15300000	1.01340100
C	-4.90618200	-2.25814100	-0.67787100
H	-4.03470100	-2.91576400	-0.55162400
H	-5.68098600	-2.58532300	0.03509000
H	-5.29411700	-2.40648200	-1.69930100
C	3.95362100	-2.59518800	0.15819900
H	3.09492700	-2.81604700	0.80945900
H	3.81326300	-3.14887800	-0.78527300
H	4.86199100	-2.97815300	0.65196700
C	2.28285300	2.71274800	0.48343900
H	2.84647100	3.48950000	1.02626400
H	1.78827100	3.19230400	-0.37970300
H	1.49864800	2.33976500	1.15832700

Cartesian coordinates of the optimized geometry of TS-**11** at PBE0-D3BJ/def2-SVP level of theory:

Ga	-1.82637000	-0.32167300	-0.56778400
Ga	1.82646500	-0.33232900	-0.56233900
P	-1.64859000	-1.34548900	-2.60657600
P	1.64972700	-1.37836700	-2.58996400
O	-0.00022700	-0.31488000	-0.35773700
N	-2.70081600	-1.13186300	0.96365400
N	-2.55885300	1.46393700	-0.35073000
N	2.56315700	1.45350200	-0.36191000
N	2.69608800	-1.12936400	0.97864900
C	-3.44086700	-0.44317300	1.82214700
C	-3.72329100	0.92889400	1.69399300

H	-4.34471900	1.35957300	2.47762400
C	-3.31661700	1.81446200	0.67973000
C	-4.02263800	-1.15879900	3.00862900
H	-4.69539700	-1.96870700	2.68502300
H	-4.58951700	-0.47303200	3.64863800
H	-3.22903700	-1.62667600	3.61187900
C	-3.77935000	3.24052900	0.78301800
H	-4.39346500	3.51613400	-0.08887500
H	-2.92146600	3.93077900	0.79877500
H	-4.37375900	3.40165900	1.68964000
C	3.32020500	1.81245300	0.66605800
C	3.72301200	0.93598500	1.68981500
H	4.34439500	1.37285800	2.47004700
C	3.43670600	-0.43389800	1.83131100
C	3.78643800	3.23829500	0.75578300
H	4.40256300	3.50357100	-0.11789400
H	4.37988200	3.40699900	1.66166200
H	2.93030000	3.93084500	0.76327700
C	4.01475700	-1.13908500	3.02583900
H	4.68583100	-1.95401500	2.71147100
H	3.21899500	-1.59871000	3.63257800
H	4.58251100	-0.44840500	3.65975900
C	0.00038100	-1.17324600	-2.18318700
C	2.17749900	2.40760400	-1.37932000
H	1.65698300	1.87198500	-2.18684800
H	1.49222800	3.17885300	-0.98584900
H	3.04565600	2.91962800	-1.82627300
C	2.43599900	-2.53800200	1.18665800
H	3.36321500	-3.12328000	1.30263000
H	1.80614500	-2.71621300	2.07559700
H	1.90241400	-2.93205900	0.30938300
C	-2.44431200	-2.54313600	1.15779400
H	-1.91089300	-2.92971500	0.27710400
H	-1.81562700	-2.73166700	2.04542300
H	-3.37303800	-3.12727800	1.26716700
C	-2.16970600	2.42728900	-1.35802800
H	-3.03612700	2.94547400	-1.80124900
H	-1.48350200	3.19326300	-0.95594700
H	-1.64908400	1.89856000	-2.17002500

7. References

[1] M. K. Sharma, C. Wölper, G. Haberhauer, S. Schulz, *Angew. Chem., Int. Ed.* 2021, **60**, 6784; *Angew. Chem.* 2021, **133**, 6859.
[2] M. K. Sharma, P. Dhawan, C. Helling, C. Wölper, S. Schulz, *Chem. Eur. J.* 2022, **28**, e202200444.
[3] A. J. Arduengo III, H. V. R. Dias, R. L. Harlow, M. Kline, *J. Am. Chem. Soc.* 1992, **114**, 5530.
[4] V. Lavallo, Y. Canac, C. Präsang, B. Donnadieu, G. Bertrand, *Angew. Chem., Int. Ed.* **2005**, *44*, 5705; *Angew. Chem.* 2005, **117**, 5851.
[5] G. R. Fulmer, A. J. M. Miller, N. H. Sherden, H. E. Gottlieb, A. Nudelman, B. M. Stoltz, J. E. Bercaw, K. I. Goldberg, *Organometallics* 2010, **29**, 2176.
[6] G. M. Sheldrick, *Acta Crystallogr., Sect. A.* 1990, **A46**, 467.
[7] G. M. Sheldrick, SHELX 2014, Program for the Refinement of Crystal Structures, University of Göttingen: Göttingen, Germany, 2014.
[8] G. M. Sheldrick, *Acta Crystallogr., Sect. A* 2008, **A64**, 112.
[9] C. B. Hübschle, G. M. Sheldrick, B. Dittrich, *J. Appl. Crystallogr.* 2011, **44**, 1281−1284.
[10] M. J. Frisch, G. W. Trucks, H. B. Schlegel, G. E. Scuseria, M. A. Robb, J. R. Cheeseman, G. Scalmani, V. Barone, G. A. Petersson, H. Nakatsuji, X. Li, M. Caricato, A. V. Marenich, J. Bloino, B. G. Janesko, R. Gomperts, B. Mennucci, H. P. Hratchian, J. V. Ortiz, A. F. Izmaylov, J. L. Sonnenberg, D. Williams-Young, F. Ding, F. Lipparini, F. Egidi, J. Goings, B. Peng, A. Petrone, T. Henderson, D. Ranasinghe, V. G. Zakrzewski, J. Gao, N. Rega, G. Zheng, W. Liang, M. Hada, M. Ehara, K. Toyota, R. Fukuda, J. Hasegawa, M. Ishida, T. Nakajima, Y. Honda, O. Kitao, H. Nakai, T. Vreven, K. Throssell, J. A. Montgomery, Jr.; , J. E. Peralta, F. Ogliaro, M. J. Bearpark, J. J. Heyd, E. N. Brothers, K. N. Kudin, V. N. Staroverov, T. A. Keith, R. Kobayashi, J. Normand, K. Raghavachari, A. P. Rendell, J. C. Burant, S. S. Iyengar, J. Tomasi, M. Cossi, J. M. Millam, M. Klene, C. Adamo, R. Cammi, J. W. Ochterski, R. L. Martin, K. Morokuma, O. Farkas, J. B. Foresman, D. J. Fox, *Gaussian 16, Revision A.03*, Gaussian, Inc., Wallingford CT, 2016.
[11] a) J. P. Perdew, K. Burke, M. Ernzerhof, *Phys. Rev. Lett.* 1996, **77**, 3865. b) J. P. Perdew, K. Burke, M. Ernzerhof, *Phys. Rev. Lett.* 1997, **78**, 1396.
[12] C. Adamo, V. Barone, *J. Chem. Phys.* 1999, **110**, 6158.
[13] S. Grimme, S. Ehrlich, L. Goerigk, *J. Comp. Chem.* 2011, **32**, 1456
[14] F. Weigend, R. Ahlrichs, *Phys. Chem. Chem. Phys.* 2005, **7**, 3297.
[15] A. V. Marenich, C. J. Cramer, D. G. Truhlar, *J. Phys. Chem. B* 2009, **113**, 6378.
